# Surveillance for Certain Health Behaviors and Conditions Among States and Selected Local Areas — Behavioral Risk Factor Surveillance System, United States, 2013 and 2014

**DOI:** 10.15585/mmwr.ss6616a1

**Published:** 2017-09-15

**Authors:** Sonya Gamble, Tebitha Mawokomatanda, Fang Xu, Pranesh P. Chowdhury, Carol Pierannunzi, David Flegel, William Garvin, Machell Town

**Affiliations:** 1 Division of Population Health, National Center for Chronic Disease Prevention and Health Promotion, CDC; 2 Northrop Grumman Corporation, Atlanta, Georgia

## Abstract

**Problem:**

Chronic diseases and conditions (e.g., heart diseases, stroke, arthritis, and diabetes) are the leading causes of morbidity and mortality in the United States. These conditions are costly to the U.S. economy, yet they are often preventable or controllable. Behavioral risk factors (e.g., excessive alcohol consumption, tobacco use, poor diet, frequent mental distress, and insufficient sleep) are linked to the leading causes of morbidity and mortality. Adopting positive health behaviors (e.g., staying physically active, quitting tobacco use, obtaining routine physical checkups, and checking blood pressure and cholesterol levels) can reduce morbidity and mortality from chronic diseases and conditions. Monitoring the health risk behaviors, chronic diseases and conditions, access to health care, and use of preventive health services at multilevel public health points (states, territories, and metropolitan and micropolitan statistical areas [MMSA]) can provide important information for development and evaluation of health intervention programs.

**Reporting Period:**

2013 and 2014.

**Description of the System:**

The Behavioral Risk Factor Surveillance System (BRFSS) is an ongoing, state-based, random-digit–dialed telephone survey of noninstitutionalized adults aged ≥18 years residing in the United States. BRFSS collects data on health risk behaviors, chronic diseases and conditions, access to health care, and use of preventive health services and practices related to the leading causes of death and disability in the United States and participating territories. This is the first BRFSS report to include age-adjusted prevalence estimates. For 2013 and 2014, these age-adjusted prevalence estimates are presented for all 50 states, the District of Columbia, the Commonwealth of Puerto Rico, Guam, and selected MMSA.

**Results:**

Age-adjusted prevalence estimates of health status indicators, health care access and preventive practices, health risk behaviors, chronic diseases and conditions, and cardiovascular conditions vary by state, territory, and MMSA. Each set of proportions presented refers to the range of age-adjusted prevalence estimates of selected BRFSS measures as reported by survey respondents.

The following are estimates for 2013. Adults reporting frequent mental distress: 7.7%–15.2% in states and territories and 6.3%–19.4% in MMSA. Adults with inadequate sleep: 27.6%–49.2% in states and territories and 26.5%–44.4% in MMSA. Adults aged 18–64 years having health care coverage: 66.9%–92.4% in states and territories and 60.5%–97.6% in MMSA. Adults identifying as current cigarette smokers: 10.1%–28.8% in states and territories and 6.1%–33.6% in MMSA. Adults reporting binge drinking during the past month: 10.5%–25.2% in states and territories and 7.2%–25.3% in MMSA. Adults with obesity: 21.0%–35.2% in states and territories and 12.1%–37.1% in MMSA. Adults aged ≥45 years with some form of arthritis: 30.6%–51.0% in states and territories and 27.6%–52.4% in MMSA. Adults aged ≥45 years who have had coronary heart disease: 7.4%–17.5% in states and territories and 6.2%–20.9% in MMSA. Adults aged ≥45 years who have had a stroke: 3.1%–7.5% in states and territories and 2.3%–9.4% in MMSA. Adults with high blood pressure: 25.2%–40.1% in states and territories and 22.2%–42.2% in MMSA. Adults with high blood cholesterol: 28.8%–38.4% in states and territories and 26.3%–39.6% in MMSA.

The following are estimates for 2014. Adults reporting frequent physical distress: 7.8%–16.0% in states and territories and 6.2%–18.5% in MMSA. Women aged 21–65 years who had a Papanicolaou test during the past 3 years: 67.7%–87.8% in states and territories and 68.0%–94.3% in MMSA. Adults aged 50–75 years who received colorectal cancer screening on the basis of the 2008 U.S. Preventive Services Task Force recommendation: 42.8%–76.7% in states and territories and 49.1%–79.6% in MMSA. Adults with inadequate sleep: 28.4%–48.6% in states and territories and 25.4%–45.3% in MMSA. Adults reporting binge drinking during the past month: 10.7%–25.1% in states and territories and 6.7%–26.3% in MMSA. Adults aged ≥45 years who have had coronary heart disease: 8.0%–17.1% in states and territories and 7.6%–19.2% in MMSA. Adults aged ≥45 years with some form of arthritis: 31.2%–54.7% in states and territories and 28.4%–54.7% in MMSA. Adults with obesity: 21.0%–35.9% in states and territories and 19.7%–42.5% in MMSA.

**Interpretation:**

Prevalence of certain chronic diseases and conditions, health risk behaviors, and use of preventive health services varies among states, territories, and MMSA. The findings of this report highlight the need for continued monitoring of health status, health care access, health behaviors, and chronic diseases and conditions at state and local levels.

**Public Health Action:**

State and local health departments and agencies can continue to use BRFSS data to identify populations at risk for certain unhealthy behaviors and chronic diseases and conditions. Data also can be used to design, monitor, and evaluate public health programs at state and local levels.

## Introduction

Chronic diseases and conditions (e.g., cardiovascular disease, cancer, chronic lower respiratory diseases, and diabetes) are among the top 10 leading causes of death in the United States (*1*). Practicing healthy behaviors (e.g., quitting smoking, being more physically active, limiting alcohol intake, eating a nutritious diet, and maintaining a healthy weight) and using preventive health services (e.g., regular checks for high blood pressure and high blood cholesterol, screening for cancer on recommended schedules, and obtaining regular physical checkups) can reduce morbidity and premature mortality from chronic diseases and conditions (*2*).

BRFSS is an ongoing, state-based, random-digit–dialed cellular and landline telephone survey of noninstitutionalized adults aged ≥18 years in each U.S. state and participating territory. Since 1984, CDC has assisted state and territorial health departments in conducting the BRFSS survey each year. The survey is one of the main data sources that public health officials and practitioners use to track chronic diseases and conditions, health risk behaviors, use of preventive health services, and emerging health problems at state and local levels. The data are frequently used to set health goals as well as to monitor progress and success of public health programs and policy implementation at national, state, and local levels. BRFSS data collection is conducted by state health departments with assistance from CDC. The estimates in this report are calculated from BRFSS data sets, which are aggregates of the combined landline and cellular telephone data submitted during 2013 and 2014. Beginning in 2002, BRFSS data have been used to generate prevalence estimates from metropolitan and micropolitan statistical areas (MMSA) that meet the system’s inclusion criteria. This report includes BRFSS findings related to selected chronic diseases and conditions, health risk behaviors, health care access, and use of preventive health services. 

## Methods

BRFSS is conducted in all 50 states, the District of Columbia, the Commonwealth of Puerto Rico, and Guam. BRFSS uses a multistage sampling design and random-digit–dialing methods to select a representative sample from the noninstitutionalized adult population aged ≥18 years in each state and participating territory (*3*,*4*). Details on methodology, random sampling procedures, design (*5*), and reliability and validity of measures (*6*) used in BRFSS have been described in previous publications. Estimates are from 53 states and territories for both years, 145 MMSA for 2013, and 132 MMSA for 2014. A list of MMSA for each year is available on the BRFSS SMART website (https://www.cdc.gov/brfss/smart/smart_data.htm). The 2013 and 2014 questionnaires and all related supporting documents are available at the BRFSS website (https://www.cdc.gov/brfss/).

MMSA are defined by the Office of Management and Budget; respondents are assigned MMSA according to their county Federal Information Processing Standards code. MMSA were included in the data set if they met the selection criterion of ≥500 participants. Data are submitted monthly to CDC by the states or their designees. Data cleaning and weighting are conducted by CDC. Complete documentation of the BRFSS methodology is available at https://www.cdc.gov/brfss.

### Questionnaire

The BRFSS questionnaire is designed to collect uniform, state-specific, self-reported data on a range of health behaviors and conditions (*3*,*4*). All questions undergo cognitive and field testing. The standard questionnaire consists of three parts: 1) core questions, 2) optional BRFSS modules, and 3) state-added questions. The core consists of a set of demographic and standard health-related questions used by all participating states and includes some topics that appear biennially. Topics and number of optional modules vary by year and are adopted by states depending upon their programmatic needs. State-added questions are developed, added, and used by the authoring state, specifically for their own residents; CDC does not develop, track, or record state-added questions. All BRFSS questionnaires are available at https://www.cdc.gov/brfss/questionnaires/index.htm.

### Data Collection and Processing

Data collection for BRFSS is conducted using a computer-assisted telephone interviewing system. Data are collected monthly by each state and territory according to BRFSS standard protocol. After the monthly interviewing cycle concludes, data are submitted to CDC to be edited, processed, weighted, checked for reliability, and prepared for analysis. At the end of the survey year, CDC processes and aggregates the monthly data files to create a year-end data file for each state and territory.

#### Sampling

In 2013, BRFSS used a partially overlapping sample that, in addition to other eligibility requirements, screened out cellular telephone respondents who received more than 10% of all incoming calls on a landline telephone. In 2014, BRFSS adopted the use of a fully overlapping sample of landline and cellular telephone respondents aged ≥18 years. No minor children are included in the BRFSS sample. States designed samples using substate regions (e.g., public health districts or other jurisdictions) to ensure geographic representation within the sample. CDC assisted states with sample design and set minimum sample sizes for substate regions, split-sample versions of the questionnaire, and oversampling of populations that are hard to reach.

#### Data Weighting

BRFSS data were used to create direct estimates for each geographic area (i.e., state or MMSA). Data were weighted using a raking method. Raking (iterative proportional fitting) was applied using each demographic factor individually in an iterative process until demographic estimates matched control totals based on U.S. Census estimates for that year. Raking has improved the precision with which the BRFSS sample reflects the sociodemographic profile at the state level. Details of the BRFSS raking method are provided in the BRFSS weighting documents for 2013 and 2014 (*7*,*8*). The 2014 sampling overlap also prompted an adjustment to the BRFSS design weights. To account for overlap of the two samples, a composite factor was multiplied by the design weight for each dual user to create an adjustment that addressed and corrected for the respondent’s probability of being selected in both frames. More information about the composite factor calculation is available at https://www.cdc.gov/brfss/annual_data/2014/pdf/compare_2014.pdf.

### Statistical Analysis

The analysis was conducted using statistical software, SAS-Callable SUDAAN release 11.0 (Research Triangle Institute, Cary, North Carolina), to account for the complex sampling design and calculate age-adjusted prevalence estimates, standard errors, and 95% confidence intervals. Sample sizes are unweighted in this report. Data with sample sizes <50 or having a relative standard error >30% were deemed unstable and less reliable and were suppressed in the tables, as noted by N/A (not available). Responses coded as do not know or refused were excluded from the analysis. Several chronic diseases and conditions (i.e., diabetes, arthritis, coronary heart disease, and stroke) were limited to participants aged ≥45 years (*9*).

This is the first BRFSS report to include age-adjusted prevalence estimates. Age adjustment is a standard analytical technique used to compare estimates between populations with different age distributions (e.g., states) and over time. In this report, the estimates were age adjusted so that data could be compared across states, MMSA, and time, each having different age distributions. BRFSS age-adjusted estimates were standardized to the 2000 U.S. population using distribution No. 8, consistent with the current National Center for Health Statistics recommendations and practice (*10*).

Crude prevalence estimates for each individual state and MMSA are provided at https://www.cdc.gov/brfss/brfssprevalence/index.html. Results should not be compared with those in previous BRFSS reports, which provided nonage-adjusted estimates by state, territory, and MMSA. The age-adjusted prevalence estimates were calculated from results of BRFSS and might differ from those derived by other methods. 

## About This Report

This report presents age-adjusted prevalence estimates and discussion of five topics. The topics are 1) health status indicators (self-rated general health status, frequent mental distress, and frequent physical distress); 2) health care access and preventive practices (health care coverage, recent routine physical checkup, Papanicolaou [Pap] test, colorectal cancer screening, and blood cholesterol check); 3) health risk behaviors (no leisure-time physical activity, inadequate sleep, current cigarette smoking, and binge drinking); 4) chronic diseases and conditions (obesity, diabetes, arthritis, and depression); and 5) cardiovascular conditions (coronary heart disease and stroke for adults aged ≥45 years and high blood pressure and high blood cholesterol for adults aged ≥18 years).

## Results

In 2013, a total of 491,773 adults completed BRFSS interviews on landline and cellular telephones. Results were from 53 states and territories and 145 MMSA with sufficient sample sizes. A total of 360,079 respondents completed the interview by landline telephone (range: 1,461 in Guam to 27,763 in Florida; median: 5,668). A total of 131,694 respondents completed the interview by cellular telephone (range: 445 in Guam to 7,620 in Kansas; median: 2,291). In 2014, a total of 464,662 adults completed the interview on landline and cellular telephones. Results were from 53 states and territories and 132 MMSA with sufficient sample size. A total of 298,568 respondents completed the interview by landline telephone (range: 1,852 in Guam to 12,962 in Nebraska; median: 4,973). A total of 166,094 respondents completed the interview by cellular telephone (range: 686 in Guam to 9,952 in Nebraska; median: 2,868).

BRFSS uses the American Association of Public Opinion Research Response Rate 4 (defined as the number of complete and partial interviews divided by the number of contacted and eligible respondents) (*11*) as a method for calculating response. In 2013, landline response rates ranged from 28.0% in Alabama to 63.7% in Puerto Rico (median: 49.6%) and cellular response rates ranged from 19.1% in Washington to 62.6% in Alaska (median: 37.8%). The combined (landline and cellular) response rates ranged from 29.0% in Alabama to 60.3% in Puerto Rico (median: 46.4%). In 2014, landline response rates ranged from 26.7% in California to 61.6% in Kentucky (median: 48.7%) and cellular response rates ranged from 22.2% in California to 60.0% in Alaska (median: 40.5%). Overall, the combined response rates ranged from 25.1% in California to 60.1% in South Dakota (median: 47.0%). BRFSS Summary Data Quality Reports for 2013 (*12*) and 2014 (*13*) have detailed information on response, cooperation, and refusal rates.

Increasing use of cellular telephones (*14*) prompted BRFSS to move to an overlapping sample in 2014. This was a change from the 2013 screening process, which restricted the eligibility of cellular telephone respondents who also used landline telephones. Effects of the 2014 sample change include larger proportions of completed interviews among persons aged 18–44 years, men, and Hispanics. Moving to an overlapping sample increased the proportion of cellular telephone respondents eligible to participate in the survey (*15*).

### Health Status Indicators

#### Health Status

All respondents were asked if their general health was excellent, very good, good, fair, or poor. Respondents were then divided into two groups: those reporting their health was excellent, very good, or good and those reporting their health was fair or poor. In 2013, age-adjusted prevalence estimates for good or better health ranged from 66.4% in Puerto Rico to 88.7% in Vermont (median: 84.0%) (Table 1). Among selected MMSA, estimated age-adjusted prevalence ranged from 66.7% in San Juan-Carolina-Caguas, Puerto Rico, to 91.2% in Sioux Falls, South Dakota (median: 83.8%) (Table 2). In 2014, age-adjusted prevalence estimates of persons with good or better health ranged from 66.9% in Puerto Rico to 89.1% in Vermont (median: 84.1%) (Table 3). Among selected MMSA, age-adjusted prevalence estimates ranged from 63.3% in Ponce, Puerto Rico, to 92.2% in Logan, Utah-Idaho (median: 83.7%) (Table 4).

**TABLE 1:** Age-adjusted* prevalence estimates of adults aged ≥18 years who reported good or better health,^†^ by state/territory — Behavioral Risk Factor Surveillance System, United States, 2013

State/Territory	Sample size	%	SE	95% CI
Alabama	6,476	78.6	0.7	(77.3–79.9)
Alaska	4,561	85.1	0.7	(83.6–86.5)
Arizona	4,242	84.1	1.0	(82.1–86.1)
Arkansas	5,243	77.1	0.8	(75.5–78.8)
California	11,508	81.4	0.5	(80.4–82.4)
Colorado	13,617	87.0	0.4	(86.3–87.8)
Connecticut	7,691	87.6	0.5	(86.6–88.7)
Delaware	5,200	83.5	0.7	(82.0–84.9)
District of Columbia	4,920	86.6	0.7	(85.2–88.0)
Florida	33,822	81.7	0.5	(80.7–82.7)
Georgia	8,113	81.2	0.6	(80.0–82.4)
Hawaii	7,850	86.9	0.5	(85.9–88.0)
Idaho	5,598	86.2	0.7	(84.9–87.6)
Illinois	5,605	83.6	0.7	(82.2–85.0)
Indiana	10,206	82.6	0.5	(81.7–83.6)
Iowa	8,145	86.4	0.5	(85.4–87.5)
Kansas	23,228	85.2	0.3	(84.6–85.8)
Kentucky	10,995	77.9	0.6	(76.7–79.1)
Louisiana	5,241	78.1	0.9	(76.4–79.8)
Maine	8,033	86.5	0.5	(85.5–87.5)
Maryland	12,972	85.4	0.5	(84.4–86.5)
Massachusetts	15,042	86.8	0.5	(85.9–87.7)
Michigan	12,745	83.2	0.5	(82.3–84.2)
Minnesota	14,299	88.1	0.5	(87.1–89.1)
Mississippi	7,424	76.5	0.7	(75.2–77.9)
Missouri	7,103	82.4	0.7	(81.1–83.8)
Montana	9,674	85.8	0.5	(84.9–86.8)
Nebraska	17,106	86.7	0.4	(85.9–87.5)
Nevada	5,087	83.1	1.0	(81.2–85.1)
New Hampshire	6,449	88.0	0.6	(86.9–89.1)
New Jersey	13,351	84.0	0.5	(83.0–85.0)
New Mexico	9,295	80.1	0.6	(78.9–81.3)
New York	8,886	82.8	0.5	(81.7–83.8)
North Carolina	8,827	81.5	0.5	(80.5–82.6)
North Dakota	7,775	86.1	0.6	(85.0–87.2)
Ohio	11,940	82.8	0.5	(81.8–83.9)
Oklahoma	8,204	80.1	0.6	(78.9–81.2)
Oregon	5,934	83.2	0.7	(81.7–84.6)
Pennsylvania	11,352	84.2	0.5	(83.2–85.1)
Rhode Island	6,508	84.3	0.6	(83.1–85.6)
South Carolina	10,667	81.4	0.5	(80.4–82.4)
South Dakota	6,887	88.2	0.6	(87.1–89.3)
Tennessee	5,785	78.1	0.8	(76.6–79.6)
Texas	10,708	80.8	0.6	(79.7–82.0)
Utah	12,737	87.1	0.4	(86.3–87.8)
Vermont	6,383	88.7	0.5	(87.7–89.8)
Virginia	8,431	84.7	0.5	(83.7–85.6)
Washington	11,129	84.7	0.5	(83.7–85.6)
West Virginia	5,885	76.4	0.6	(75.1–77.6)
Wisconsin	6,582	85.2	0.7	(83.8–86.7)
Wyoming	6,433	85.4	0.6	(84.2–86.6)
Guam	1,896	79.0	1.3	(76.6–81.5)
Puerto Rico	6,003	66.4	0.8	(64.9–67.9)
*Median*		*84.0*		
*Range*		*66.4–88.7*		

**Abbreviations:** CI = confidence interval; SE = standard error.

* Age adjusted to the 2000 U.S. standard population.

^† ^Respondents were asked to rate their general health as poor, fair, good, very good, or excellent. Respondents were classified into two groups: those who reported poor or fair health and those who reported good, very good, or excellent health.

**TABLE 2:** Age-adjusted* prevalence estimates of adults aged ≥18 years who reported good or better health,^†^ by metropolitan and micropolitan statistical area — Behavioral Risk Factor Surveillance System, United States, 2013

MMSA	Sample size	%	SE	95% CI
Aguadilla-Isabela, Puerto Rico	593	67.8	2.5	(62.9–72.7)
Akron, Ohio	687	79.8	2.6	(74.8–84.8)
Albuquerque, New Mexico	2,081	81.4	1.2	(79.1–83.7)
Allentown-Bethlehem-Easton, Pennsylvania-New Jersey	1,027	80.1	2.0	(76.1–84.1)
Anchorage, Alaska	1,523	86.1	1.1	(83.9–88.3)
Atlanta-Sandy Springs-Roswell, Georgia	3,508	83.6	0.8	(82.0–85.3)
Augusta-Richmond County, Georgia-South Carolina	913	79.4	2.0	(75.5–83.4)
Austin-Round Rock, Texas	924	84.9	1.7	(81.6–88.2)
Baltimore-Columbia-Towson, Maryland	4,757	85.0	0.8	(83.4–86.6)
Baton Rouge, Louisiana	928	80.8	1.9	(77.1–84.4)
Billings, Montana	816	83.6	1.5	(80.7–86.4)
Birmingham-Hoover, Alabama	1,349	80.8	1.3	(78.3–83.3)
Bismarck, North Dakota	1,032	86.1	1.4	(83.4–88.9)
Boise City, Idaho	1,485	88.5	1.1	(86.3–90.7)
Boston, Massachusetts^§^	4,067	87.3	0.8	(85.6–88.9)
Buffalo-Cheektowaga-Niagara Falls, New York	505	84.9	2.5	(80.0–89.7)
Burlington-South Burlington, Vermont	1,632	89.7	0.9	(87.8–91.5)
Cambridge-Newton-Framingham, Massachusetts^§^	4,888	88.0	0.7	(86.6–89.4)
Camden, New Jersey^§^	1,866	83.2	1.3	(80.8–85.7)
Cedar Rapids, Iowa	648	85.1	2.1	(80.9–89.2)
Charleston, West Virginia	819	77.6	1.7	(74.2–81.0)
Charleston-North Charleston, South Carolina	1,547	84.1	1.2	(81.7–86.5)
Charlotte-Concord-Gastonia, North Carolina-South Carolina	1,948	82.4	1.1	(80.3–84.5)
Chattanooga, Tennessee-Georgia	577	78.9	2.5	(74.0–83.9)
Chicago-Naperville-Elgin, Illinois-Indiana-Wisconsin	3,330	83.2	0.9	(81.4–85.1)
Cincinnati, Ohio-Kentucky-Indiana	2,601	84.6	1.0	(82.5–86.6)
Claremont-Lebanon, New Hampshire-Vermont	1,688	86.7	1.5	(83.8–89.6)
Cleveland-Elyria, Ohio	1,105	81.6	1.8	(78.2–85.1)
Colorado Springs, Colorado	1,376	87.8	1.0	(85.7–89.8)
Columbia, South Carolina	1,442	85.1	1.1	(82.9–87.4)
Columbus, Ohio	1,861	84.2	1.1	(82.1–86.4)
Crestview-Fort Walton Beach-Destin, Florida	1,069	82.1	1.7	(78.7–85.5)
Dallas-Plano-Irving, Texas^§^	887	83.8	1.4	(81.0–86.5)
Davenport-Moline-Rock Island, Iowa-Illinois	672	83.0	2.3	(78.5–87.4)
Dayton, Ohio	837	87.6	1.4	(85.0–90.3)
Deltona-Daytona Beach-Ormond Beach, Florida	1,103	85.1	1.6	(81.9–88.3)
Denver-Aurora-Lakewood, Colorado	5,702	87.0	0.6	(85.9–88.1)
Des Moines-West Des Moines, Iowa	1,343	88.8	1.0	(86.8–90.9)
Duluth, Minnesota-Wisconsin	699	87.5	1.7	(84.1–90.9)
Durham-Chapel Hill, North Carolina	617	84.0	1.9	(80.2–87.8)
El Paso, Texas	743	79.5	1.7	(76.1–82.9)
Evansville, Indiana-Kentucky	571	80.4	2.5	(75.6–85.3)
Fargo, North Dakota-Minnesota	1,179	86.1	1.4	(83.3–88.9)
Fayetteville-Springdale-Rogers, Arkansas-Missouri	820	79.4	2.0	(75.5–83.3)
Fort Smith, Arkansas-Oklahoma	497	69.7	3.3	(63.2–76.2)
Fort Wayne, Indiana	772	86.1	1.7	(82.8–89.4)
Fort Worth-Arlington, Texas^§^	802	85.7	1.6	(82.6–88.9)
Gainesville, Florida	1,018	87.0	1.6	(83.8–90.2)
Grand Forks, North Dakota-Minnesota	503	86.0	2.4	(81.2–90.7)
Grand Island, Nebraska	798	82.6	1.9	(78.9–86.3)
Grand Rapids-Wyoming, Michigan	1,346	86.8	1.4	(84.1–89.4)
Greensboro-High Point, North Carolina	662	82.0	1.9	(78.2–85.7)
Greenville-Anderson-Mauldin, South Carolina	1,339	83.1	1.4	(80.3–85.9)
Gulfport-Biloxi-Pascagoula, Mississippi	771	75.6	2.0	(71.6–79.6)
Hagerstown-Martinsburg, Maryland-West Virginia	766	82.2	1.9	(78.4–86.0)
Hartford-West Hartford-East Hartford, Connecticut	2,831	88.2	0.8	(86.6–89.9)
Hilton Head Island-Bluffton-Beaufort, South Carolina	825	89.5	1.5	(86.7–92.4)
Houston-The Woodlands-Sugar Land, Texas	1,363	81.9	1.5	(78.9–84.9)
Huntington-Ashland, West Virginia-Kentucky-Ohio	1,175	75.6	1.5	(72.6–78.5)
Idaho Falls, Idaho	506	80.0	2.6	(74.9–85.0)
Indianapolis-Carmel-Anderson, Indiana	2,516	82.9	1.0	(80.9–84.9)
Jackson, Mississippi	802	81.8	1.7	(78.5–85.2)
Jacksonville, Florida	2,852	82.4	1.0	(80.3–84.4)
Kansas City, Missouri-Kansas	7,411	85.2	0.9	(83.5–86.9)
Kingsport-Bristol-Bristol, Tennessee-Virginia	536	73.7	3.0	(67.8–79.7)
Knoxville, Tennessee	651	78.2	2.1	(74.1–82.2)
Lansing-East Lansing, Michigan	687	86.3	1.5	(83.3–89.2)
Lexington-Fayette, Kentucky	637	80.8	2.0	(76.8–84.8)
Lincoln, Nebraska	1,875	88.8	0.8	(87.2–90.5)
Little Rock-North Little Rock-Conway, Arkansas	1,140	79.0	1.6	(75.8–82.1)
Logan, Utah-Idaho	641	86.5	1.8	(83.0–89.9)
Los Angeles-Long Beach-Anaheim, California	3,041	80.6	0.9	(78.8–82.4)
Louisville/Jefferson County, Kentucky-Indiana	2,142	81.7	1.3	(79.2–84.2)
Lubbock, Texas	525	80.0	2.6	(74.9–85.0)
Manhattan, Kansas	663	90.2	1.2	(87.8–92.5)
Memphis, Tennessee-Mississippi-Arkansas	1,206	81.4	1.5	(78.6–84.3)
Miami-Fort Lauderdale-West Palm Beach, Florida	2,193	82.1	1.3	(79.5–84.7)
Milwaukee-Waukesha-West Allis, Wisconsin	1,271	81.2	1.9	(77.6–84.9)
Minneapolis-St. Paul-Bloomington, Minnesota-Wisconsin	9,113	88.7	0.6	(87.4–89.9)
Minot, North Dakota	650	87.8	1.4	(85.0–90.6)
Montgomery County-Bucks County-Chester County, Pennsylvania^§^	963	88.5	1.2	(86.1–90.9)
Myrtle Beach-Conway-North Myrtle Beach, South Carolina-North Carolina	775	81.8	1.7	(78.4–85.1)
Nashville-Davidson County-Murfreesboro-Franklin, Tennessee	1,055	84.5	1.4	(81.8–87.2)
Nassau County-Suffolk County, New York^§^	936	85.5	1.4	(82.7–88.3)
Newark, New Jersey-Pennsylvania^§^	4,113	85.3	0.8	(83.7–86.8)
New Orleans-Metairie, Louisiana	1,284	79.7	1.8	(76.2–83.2)
New York-Jersey City-White Plains, New York-New Jersey^§^	8,889	80.7	0.6	(79.5–81.9)
Norfolk, Nebraska	673	81.5	1.9	(77.8–85.1)
North Platte, Nebraska	718	85.4	1.8	(81.8–89.0)
North Port-Sarasota-Bradenton, Florida	1,082	81.2	2.0	(77.4–85.1)
Oakland-Hayward-Berkeley, California^§^	700	87.0	1.5	(84.1–89.8)
Ogden-Clearfield, Utah	2,454	88.3	0.8	(86.8–89.8)
Oklahoma City, Oklahoma	2,634	83.0	0.8	(81.4–84.7)
Omaha-Council Bluffs, Nebraska-Iowa	3,123	86.8	0.8	(85.2–88.4)
Orlando-Kissimmee-Sanford, Florida	2,264	80.0	1.3	(77.5–82.5)
Panama City, Florida	1,021	80.8	1.9	(77.1–84.5)
Pensacola-Ferry Pass-Brent, Florida	1,307	82.7	1.3	(80.1–85.2)
Philadelphia, Pennsylvania^§^	1,765	81.5	1.2	(79.2–83.9)
Phoenix-Mesa-Scottsdale, Arizona	1,545	85.2	1.4	(82.4–88.0)
Pittsburgh, Pennsylvania	2,344	85.5	0.9	(83.7–87.3)
Ponce, Puerto Rico	530	68.3	2.1	(64.3–72.4)
Portland-South Portland, Maine	2,618	90.0	0.7	(88.5–91.4)
Portland-Vancouver-Hillsboro, Oregon-Washington	3,239	84.6	0.9	(82.8–86.5)
Port St. Lucie, Florida	1,020	82.6	2.3	(78.1–87.1)
Providence-Warwick, Rhode Island-Massachusetts	8,282	84.0	0.7	(82.5–85.4)
Provo-Orem, Utah	1,854	88.2	0.9	(86.5–90.0)
Raleigh, North Carolina	673	84.8	1.6	(81.7–87.9)
Rapid City, South Dakota	871	87.1	1.3	(84.5–89.7)
Reno, Nevada	1,818	82.5	1.3	(80.0–85.0)
Richmond, Virginia	1,308	85.1	1.2	(82.8–87.4)
Riverside-San Bernardino-Ontario, California	1,370	78.6	1.4	(75.9–81.3)
Rochester, New York	504	88.5	1.5	(85.5–91.5)
Rockingham County-Strafford County, New Hampshire^§^	1,660	88.8	1.0	(86.7–90.8)
Sacramento-Roseville-Arden-Arcade, California	888	83.5	1.9	(79.8–87.3)
St. Louis, Missouri-Illinois	2,058	83.7	1.2	(81.3–86.0)
Salem, Oregon	527	83.5	2.5	(78.6–88.5)
Salina, Kansas	523	83.1	2.2	(78.8–87.4)
Salisbury, Maryland-Delaware	2,060	84.4	1.2	(82.1–86.8)
Salt Lake City, Utah	4,662	86.5	0.6	(85.2–87.7)
San Antonio-New Braunfels, Texas	928	76.7	1.8	(73.2–80.3)
San Francisco-Redwood City-South San Francisco, California^§^	539	84.4	2.0	(80.4–88.4)
San Jose-Sunnyvale-Santa Clara, California	627	85.4	2.1	(81.3–89.5)
San Juan-Carolina-Caguas, Puerto Rico	3,652	66.7	1.0	(64.8–68.6)
Scottsbluff, Nebraska	712	82.0	2.0	(78.0–86.0)
Scranton-Wilkes-Barre-Hazleton, Pennsylvania	564	84.7	2.1	(80.5–88.8)
Seattle-Bellevue-Everett, Washington^§^	3,762	87.2	0.7	(85.7–88.6)
Shreveport-Bossier City, Louisiana	571	79.2	2.5	(74.3–84.0)
Silver Spring-Frederick-Rockville, Maryland^§^	2,417	88.4	1.1	(86.2–90.6)
Sioux City, Iowa-Nebraska-South Dakota	1,055	85.4	2.2	(81.0–89.8)
Sioux Falls, South Dakota	1,007	91.2	1.1	(89.0–93.4)
Spartanburg, South Carolina	592	79.2	2.6	(74.2–84.2)
Spokane-Spokane Valley, Washington	857	84.7	1.8	(81.3–88.2)
Springfield, Massachusetts	1,573	83.1	1.5	(80.1–86.1)
Tallahassee, Florida	1,840	85.7	1.3	(83.1–88.2)
Tampa-St. Petersburg-Clearwater, Florida	2,193	82.1	1.1	(79.9–84.4)
Toledo, Ohio	998	83.2	1.8	(79.7–86.7)
Topeka, Kansas	2,399	83.0	1.0	(81.1–84.9)
Tulsa, Oklahoma	1,988	81.1	1.2	(78.8–83.4)
Virginia Beach-Norfolk-Newport News, Virginia-North Carolina	1,679	84.8	1.1	(82.7–87.0)
Warren-Troy-Farmington Hills, Michigan^§^	2,259	85.6	0.9	(83.7–87.4)
Washington-Arlington-Alexandria, District of Columbia-Virginia-Maryland-West Virginia^§^	9,005	87.4	0.6	(86.2–88.7)
Wichita, Kansas	4,921	84.5	0.6	(83.3–85.8)
Wilmington, Delaware-Maryland-New Jersey^§^	3,276	83.2	0.9	(81.4–85.1)
Winston-Salem, North Carolina	694	82.6	2.0	(78.7–86.6)
Worcester, Massachusetts-Connecticut	2,760	85.3	1.1	(83.0–87.5)
*Median*		*83.8*		
*Range*		*66.7–91.2*		

**Abbreviations:** CI = confidence interval; MMSA = metropolitan and micropolitan statistical areas; SE = standard error.

* Age adjusted to the 2000 U.S. standard population.

^†^ Respondents were asked to rate their general health as poor, fair, good, very good, or excellent. Respondents were classified into two groups: those who reported poor or fair health and those who reported good, very good, or excellent health.

^§^ Metropolitan division.

**TABLE 3:** Age-adjusted* prevalence estimates of adults aged ≥18 years who reported good or better health,^†^ by state/territory — Behavioral Risk Factor Surveillance System, United States, 2014

State/Territory	Sample size	%	SE	95% CI
Alabama	8,624	78.5	0.6	(77.3–79.6)
Alaska	4,371	86.6	0.7	(85.2–87.9)
Arizona	14,832	81.6	0.6	(80.5–82.7)
Arkansas	5,238	77.7	0.9	(75.9–79.5)
California	8,819	82.3	0.5	(81.2–83.3)
Colorado	13,352	87.1	0.4	(86.3–87.8)
Connecticut	7,935	86.2	0.6	(85.1–87.4)
Delaware	4,285	85.3	0.8	(83.8–86.8)
District of Columbia	4,067	86.5	0.9	(84.8–88.2)
Florida	9,749	82.1	0.6	(81.0–83.2)
Georgia	6,332	81.7	0.7	(80.4–83.0)
Hawaii	7,244	86.0	0.6	(84.9–87.1)
Idaho	5,468	87.2	0.7	(85.9–88.5)
Illinois	5,051	83.1	0.8	(81.6–84.6)
Indiana	11,431	81.8	0.5	(80.9–82.8)
Iowa	8,114	87.3	0.5	(86.3–88.2)
Kansas	13,705	85.2	0.4	(84.5–85.9)
Kentucky	11,179	76.8	0.7	(75.4–78.2)
Louisiana	6,765	79.1	0.6	(77.9–80.3)
Maine	9,101	85.9	0.6	(84.8–87.0)
Maryland	12,543	86.2	0.6	(84.9–87.4)
Massachusetts	15,614	86.2	0.5	(85.3–87.1)
Michigan	8,454	84.1	0.5	(83.1–85.2)
Minnesota	16,375	88.4	0.3	(87.8–89.1)
Mississippi	4,183	79.0	0.8	(77.4–80.6)
Missouri	7,065	84.2	0.6	(83.0–85.4)
Montana	7,474	85.7	0.6	(84.4–86.9)
Nebraska	22,370	87.4	0.4	(86.7–88.1)
Nevada	3,756	81.9	1.0	(79.9–83.9)
New Hampshire	6,171	87.1	0.7	(85.6–88.5)
New Jersey	13,000	83.9	0.5	(82.9–84.9)
New Mexico	8,915	80.2	0.7	(78.8–81.5)
New York	6,802	83.5	0.6	(82.3–84.7)
North Carolina	7,256	81.8	0.6	(80.7–82.9)
North Dakota	7,734	86.5	0.6	(85.3–87.7)
Ohio	10,907	83.1	0.6	(82.0–84.3)
Oklahoma	8,431	80.4	0.5	(79.3–81.4)
Oregon	5,206	85.1	0.7	(83.8–86.4)
Pennsylvania	10,923	84.0	0.5	(83.0–85.0)
Rhode Island	6,435	85.6	0.6	(84.4–86.8)
South Carolina	10,990	81.3	0.5	(80.3–82.3)
South Dakota	7,391	86.8	0.7	(85.4–88.2)
Tennessee	5,128	77.4	0.8	(75.8–79.1)
Texas	15,122	80.7	0.5	(79.6–81.7)
Utah	14,977	87.5	0.3	(86.9–88.1)
Vermont	6,458	89.1	0.5	(88.1–90.0)
Virginia	9,444	83.6	0.5	(82.6–84.7)
Washington	10,073	84.3	0.5	(83.3–85.4)
West Virginia	6,186	6.5	0.7	(75.2–77.8)
Wisconsin	7,035	85.2	0.7	(83.9–86.5)
Wyoming	6,393	86.7	0.7	(85.3–88.2)
Guam	2,516	78.8	1.1	(76.6–81.0)
Puerto Rico	5,983	66.9	0.7	(65.5–68.2)
*Median*		*84.1*		
*Range*		*66.9–89.1*		

**Abbreviations:** CI = confidence interval; SE = standard error.

* Age adjusted to the 2000 U.S. standard population.

^†^ Respondents were asked to rate their general health as poor, fair, good, very good, or excellent. Respondents were classified into two groups: those who reported fair or poor health and those who reported good, very good, or excellent health.

**TABLE 4:** Age-adjusted* prevalence estimates of adults aged ≥18 years who reported good or better health,^†^ by metropolitan and micropolitan statistical area — Behavioral Risk Factor Surveillance System, United States, 2014

MMSA	Sample size	%	SE	95% CI
Aberdeen, South Dakota	620	85.2	2.1	(81.1–89.2)
Aguadilla-Isabela, Puerto Rico	544	69.9	2.1	(65.8–73.9)
Albuquerque, New Mexico	1,786	81.7	1.3	(79.3–84.2)
Allentown-Bethlehem-Easton, Pennsylvania-New Jersey	1,088	85.3	1.8	(81.8–88.8)
Anchorage, Alaska	1,781	86.7	1.0	(84.7–88.7)
Atlanta-Sandy Springs-Roswell, Georgia	2,768	84.8	0.9	(83.0–86.6)
Augusta-Richmond County, Georgia-South Carolina	887	80.0	2.7	(74.7–85.2)
Austin-Round Rock, Texas	2,221	85.3	0.9	(83.5–87.2)
Baltimore-Columbia-Towson, Maryland	4,608	85.8	1.0	(83.9–87.8)
Baton Rouge, Louisiana	923	80.0	1.6	(76.8–83.2)
Berlin, New Hampshire-Vermont	538	87.8	1.4	(85.0–90.6)
Billings, Montana	804	83.6	1.7	(80.4–86.9)
Birmingham-Hoover, Alabama	1,568	80.9	1.3	(78.4–83.4)
Bismarck, North Dakota	1,032	86.5	1.7	(83.2–89.9)
Boise City, Idaho	1,353	87.8	1.3	(85.3–90.3)
Boston, Massachusetts^§^	4,539	87.0	0.9	(85.2–88.7)
Burlington-South Burlington, Vermont	1,982	90.5	0.8	(88.9–92.2)
Cambridge-Newton-Framingham, Massachusetts^§^	5,167	87.0	0.8	(85.5–88.5)
Camden, New Jersey^§^	1,714	84.8	1.4	(82.0–87.5)
Cedar Rapids, Iowa	638	87.9	1.7	(84.5–91.3)
Charleston, West Virginia	875	77.5	1.8	(74.0–81.0)
Charleston-North Charleston, South Carolina	1,402	83.7	1.4	(81.0–86.5)
Charlotte-Concord-Gastonia, North Carolina-South Carolina	2,152	82.8	1.0	(80.8–84.9)
Chicago-Naperville-Elgin, Illinois-Indiana-Wisconsin	4,123	82.8	0.9	(80.9–84.6)
Cincinnati, Ohio-Kentucky-Indiana	2,041	83.2	1.4	(80.6–85.9)
Claremont-Lebanon, New Hampshire-Vermont	1,683	87.5	1.5	(84.6–90.3)
Cleveland-Elyria, Ohio	962	84.8	1.7	(81.4–88.1)
College Station-Bryan, Texas	565	83.4	2.6	(78.4–88.4)
Colorado Springs, Colorado	1,298	88.4	1.0	(86.3–90.4)
Columbia, South Carolina	1,206	82.2	1.4	(79.5–84.9)
Columbus, Ohio	1,651	83.4	1.4	(80.7–86.2)
Corpus Christi, Texas	606	75.7	3.5	(68.7–82.6)
Dallas-Plano-Irving, Texas^§^	1,269	84.3	1.5	(81.3–87.3)
Dayton, Ohio	564	81.6	2.3	(77.1–86.0)
Denver-Aurora-Lakewood, Colorado	5,782	87.2	0.5	(86.1–88.2)
Des Moines-West Des Moines, Iowa	1,357	87.9	1.2	(85.6–90.2)
Duluth, Minnesota-Wisconsin	945	85.4	1.5	(82.4–88.4)
El Paso, Texas	695	74.3	2.1	(70.2–78.5)
Evansville, Indiana-Kentucky	652	83.4	2.1	(79.2–87.5)
Fargo, North Dakota-Minnesota	1,150	87.7	1.2	(85.2–90.1)
Fayetteville-Springdale-Rogers, Arkansas-Missouri	810	80.7	2.1	(76.7–84.8)
Fort Wayne, Indiana	860	83.3	2.1	(79.2–87.4)
Fort Worth-Arlington, Texas^§^	742	80.4	2.2	(76.0–84.8)
Grand Island, Nebraska	1,058	80.3	2.0	(76.3–84.3)
Grand Rapids-Wyoming, Michigan	897	87.9	1.7	(84.6–91.3)
Greensboro-High Point, North Carolina	519	81.1	2.1	(77.0–85.3)
Greenville-Anderson-Mauldin, South Carolina	1,490	83.3	1.3	(80.8–85.7)
Hagerstown-Martinsburg, Maryland-West Virginia	780	82.3	2.2	(78.0–86.6)
Hartford-West Hartford-East Hartford, Connecticut	2,639	87.0	0.9	(85.2–88.7)
Hilton Head Island-Bluffton-Beaufort, South Carolina	550	85.1	2.6	(80.1–90.1)
Houston-The Woodlands-Sugar Land, Texas	2,115	81.2	1.4	(78.6–83.9)
Huntington-Ashland, West Virginia-Kentucky-Ohio	1,246	77.6	1.4	(74.8–80.4)
Idaho Falls, Idaho	516	86.4	1.7	(83.0–89.8)
Indianapolis-Carmel-Anderson, Indiana	3,576	82.8	0.9	(81.1–84.5)
Jacksonville, Florida	670	83.8	1.9	(80.0–87.6)
Kansas City, Missouri-Kansas	4,873	85.1	0.9	(83.4–86.9)
Kingsport-Bristol-Bristol, Tennessee-Virginia	505	76.7	2.8	(71.1–82.2)
Knoxville, Tennessee	564	73.0	3.1	(66.9–79.1)
Lafayette, Louisiana	561	79.8	2.0	(75.8–83.7)
Lexington-Fayette, Kentucky	624	82.5	2.0	(78.6–86.4)
Lincoln, Nebraska	2,007	90.8	0.8	(89.2–92.3)
Little Rock-North Little Rock-Conway, Arkansas	1,179	81.5	1.7	(78.2–84.8)
Logan, Utah-Idaho	624	92.2	1.1	(90.0–94.3)
Los Angeles-Long Beach-Anaheim, California	2,449	80.1	1.0	(78.2–82.1)
Louisville-Jefferson County, Kentucky-Indiana	2,454	79.6	1.6	(76.5–82.7)
Madison, Wisconsin	549	90.7	1.5	(87.8–93.6)
Memphis, Tennessee-Mississippi-Arkansas	881	82.0	2.0	(78.0–86.0)
Miami-Fort Lauderdale-West Palm Beach, Florida	2,212	81.3	1.1	(79.1–83.6)
Milwaukee-Waukesha-West Allis, Wisconsin	1,353	80.9	1.8	(77.3–84.5)
Minneapolis-St. Paul-Bloomington, Minnesota-Wisconsin	8,745	89.1	0.4	(88.3–89.9)
Minot, North Dakota	591	86.9	1.9	(83.2–90.6)
Montgomery, Alabama	513	78.1	2.3	(73.6–82.6)
Montgomery County-Bucks County-Chester County, Pennsylvania^§^	796	90.9	1.2	(88.5–93.3)
Myrtle Beach-Conway-North Myrtle Beach, South Carolina-North Carolina	995	81.2	1.4	(78.4–84.0)
Nashville-Davidson County-Murfreesboro-Franklin, Tennessee	803	81.3	1.7	(77.9–84.6)
Nassau County-Suffolk County, New York^§^	765	84.8	1.8	(81.2–88.4)
Newark, New Jersey-Pennsylvania^§^	4,141	85.1	0.9	(83.4–86.8)
New Orleans-Metairie, Louisiana	1,914	82.5	1.0	(80.6–84.4)
New York-Jersey City-White Plains, New York-New Jersey^§^	7,486	81.5	0.7	(80.1–82.8)
Norfolk, Nebraska	995	84.0	1.4	(81.3–86.8)
North Platte, Nebraska	964	84.7	1.4	(81.9–87.4)
North Port-Sarasota-Bradenton, Florida	506	83.6	3.0	(77.8–89.5)
Oakland-Hayward-Berkeley, California^§^	699	86.8	1.5	(83.9–89.7)
Ogden-Clearfield, Utah	2,922	87.6	0.7	(86.2–89.0)
Oklahoma City, Oklahoma	2,432	83.3	0.9	(81.6–85.1)
Omaha-Council Bluffs, Nebraska-Iowa	4,861	87.6	0.7	(86.3–88.9)
Orlando-Kissimmee-Sanford, Florida	952	81.6	1.8	(78.1–85.1)
Philadelphia, Pennsylvania^§^	1,509	78.4	1.5	(75.5–81.4)
Phoenix-Mesa-Scottsdale, Arizona	9,380	82.3	0.6	(81.1–83.6)
Pittsburgh, Pennsylvania	2,402	84.4	1.0	(82.5–86.3)
Ponce, Puerto Rico	530	63.3	2.4	(58.6–68.1)
Portland-South Portland, Maine	2,746	87.9	1.0	(86.0–89.8)
Portland-Vancouver-Hillsboro, Oregon-Washington	2,840	87.1	0.8	(85.5–88.7)
Providence-Warwick, Rhode Island-Massachusetts	8,092	84.8	0.7	(83.4–86.2)
Provo-Orem, Utah	2,146	88.2	0.8	(86.7–89.8)
Raleigh, North Carolina	720	85.6	1.5	(82.7–88.6)
Rapid City, South Dakota	1,420	85.7	1.4	(82.9–88.5)
Reno, Nevada	1,201	83.4	1.5	(80.4–86.3)
Richmond, Virginia	1,462	85.9	1.1	(83.7–88.2)
Riverside-San Bernardino-Ontario, California	940	80.5	1.6	(77.4–83.7)
Roanoke, Virginia	531	86.6	1.7	(83.2–89.9)
Rochester, Minnesota	699	90.6	1.3	(88.0–93.2)
Rockingham County-Strafford County, New Hampshire^§^	1,441	87.2	1.3	(84.7–89.7)
Sacramento-Roseville-Arden-Arcade, California	642	84.2	1.7	(80.8–87.6)
St. Cloud, Minnesota	559	90.3	1.5	(87.4–93.3)
St. Louis, Missouri-Illinois	1,924	85.4	1.1	(83.1–87.6)
Salisbury, Maryland-Delaware	1,954	82.9	1.4	(80.1–85.7)
Salt Lake City, Utah	5,406	86.8	0.5	(85.7–87.8)
San Antonio-New Braunfels, Texas	2,240	81.4	1.0	(79.4–83.5)
San Juan-Carolina-Caguas, Puerto Rico	3,745	67.2	0.9	(65.4–68.9)
Scottsbluff, Nebraska	903	80.6	1.9	(76.9–84.3)
Seattle-Bellevue-Everett, Washington^§^	3,689	86.5	0.8	(84.8–88.1)
Shreveport-Bossier City, Louisiana	549	75.1	2.3	(70.6–79.7)
Silver Spring-Frederick-Rockville, Maryland^§^	2,379	86.8	1.6	(83.7–89.8)
Sioux City, Iowa-Nebraska-South Dakota	1,142	83.3	2.4	(78.6–88.0)
Sioux Falls, South Dakota	1,344	87.0	1.5	(84.0–89.9)
Spartanburg, South Carolina	561	79.8	2.5	(74.9–84.7)
Spokane-Spokane Valley, Washington	726	83.2	2.0	(79.3–87.2)
Springfield, Massachusetts	1,105	81.3	1.7	(78.0–84.6)
Tampa-St. Petersburg-Clearwater, Florida	1,571	82.1	1.5	(79.2–84.9)
Toledo, Ohio	649	76.6	2.6	(71.6–81.7)
Topeka, Kansas	1,442	82.8	1.3	(80.2–85.3)
Tulsa, Oklahoma	2,028	82.0	1.1	(79.8–84.1)
Tuscaloosa, Alabama	717	78.5	1.9	(74.7–82.2)
Virginia Beach-Norfolk-Newport News, Virginia-North Carolina	1,877	83.0	1.3	(80.5–85.5)
Warren-Troy-Farmington Hills, Michigan^§^	2,115	85.1	1.1	(83.0–87.2)
Washington-Arlington-Alexandria, District of Columbia-Virginia-Maryland-West Virginia^§^	8,287	88.1	0.7	(86.8–89.4)
Wichita, Kansas	2,732	84.5	0.8	(82.9–86.1)
Wichita Falls, Texas	539	82.1	2.7	(76.8–87.3)
Wilmington, Delaware-Maryland-New Jersey^§^	2,760	85.2	1.0	(83.3–87.1)
Worcester, Massachusetts-Connecticut	2,461	86.0	1.1	(83.9–88.2)
Youngstown-Warren-Boardman, Ohio-Pennsylvania	525	84.6	2.0	(80.7–88.5)
*Median*		*83.7*		
*Range*		*63.3–92.2*		

**Abbreviations:** CI = confidence interval; MMSA = metropolitan and micropolitan statistical areas; SE = standard error.

* Age adjusted to the 2000 U.S. standard population.

^†^ Respondents were asked to rate their general health as poor, fair, good, very good, or excellent. Respondents were classified into two groups: those who reported fair or poor health and those who reported good, very good, or excellent health.

^§^ Metropolitan division.

#### Frequent Mental Distress 

All respondents were asked to determine how many days during the past 30 days their mental health status (e.g., stress, depression, and problems with emotions) was not good. The respondents were divided into two groups: those who reported frequent mental distress (≥14 mentally unhealthy days during the past 30 days) and those who reported no frequent mental distress (<14 mentally unhealthy days during the past 30 days). In 2013, age-adjusted prevalence estimates of frequent mental distress ranged from 7.7% in North Dakota to 15.2% in West Virginia (median: 11.3%) (Table 5). Among selected MMSA, estimated age-adjusted prevalence ranged from 6.3% in Grand Forks, North Dakota-Minnesota, and Minot, North Dakota, to 19.4% in Akron, Ohio (median: 10.9%) (Table 6).

**TABLE 5:** Age-adjusted* prevalence estimates of adults aged ≥18 years who reported ≥14 days of frequent mental distress during the past 30 days,^†^ by state/territory — Behavioral Risk Factor Surveillance System, United States, 2013

State/Territory	Sample size	%	SE	95% CI
Alabama	6,334	14.4	0.7	(13.1–15.7)
Alaska	4,505	9.1	0.6	(7.8–10.3)
Arizona	4,180	12.3	1.1	(10.2–14.4)
Arkansas	5,139	14.9	0.8	(13.3–16.4)
California	11,410	11.6	0.4	(10.8–12.4)
Colorado	13,453	9.6	0.4	(8.9–10.3)
Connecticut	7,592	10.4	0.5	(9.4–11.5)
Delaware	5,134	11.2	0.7	(9.9–12.5)
District of Columbia	4,832	10.2	0.8	(8.8–11.7)
Florida	33,227	12.8	0.5	(11.9–13.7)
Georgia	7,982	10.5	0.5	(9.6–11.5)
Hawaii	7,783	8.3	0.4	(7.4–9.2)
Idaho	5,523	11.4	0.7	(10.1–12.7)
Illinois	5,564	11.0	0.7	(9.7–12.3)
Indiana	10,088	12.5	0.5	(11.5–13.5)
Iowa	8,058	9.0	0.5	(8.1–10.0)
Kansas	22,972	9.8	0.3	(9.3–10.3)
Kentucky	10,851	14.3	0.6	(13.3–15.4)
Louisiana	5,147	13.7	0.9	(12.0–15.5)
Maine	7,950	12.7	0.6	(11.5–13.9)
Maryland	12,779	10.0	0.5	(9.1–10.9)
Massachusetts	14,792	12.0	0.5	(11.1–13.0)
Michigan	12,601	12.0	0.4	(11.1–12.9)
Minnesota	14,158	8.5	0.4	(7.6–9.3)
Mississippi	7,298	14.2	0.6	(13.0–15.5)
Missouri	7,028	12.3	0.6	(11.1–13.5)
Montana	9,566	10.2	0.4	(9.3–11.0)
Nebraska	16,889	9.0	0.4	(8.2–9.8)
Nevada	5,003	11.0	0.9	(9.3–12.6)
New Hampshire	6,378	11.1	0.6	(9.9–12.2)
New Jersey	12,996	9.7	0.4	(8.9–10.5)
New Mexico	9,164	12.2	0.5	(11.2–13.3)
New York	8,730	11.3	0.5	(10.3–12.3)
North Carolina	8,702	11.7	0.5	(10.7–12.7)
North Dakota	7,617	7.7	0.5	(6.7–8.7)
Ohio	11,751	12.2	0.5	(11.3–13.2)
Oklahoma	8,114	14.3	0.6	(13.2–15.4)
Oregon	5,861	13.5	0.7	(12.1–14.8)
Pennsylvania	11,193	12.8	0.5	(11.8–13.7)
Rhode Island	6,428	11.6	0.6	(10.4–12.9)
South Carolina	10,501	11.7	0.5	(10.7–12.6)
South Dakota	6,818	7.9	0.6	(6.7–9.0)
Tennessee	5,703	12.1	0.6	(10.8–13.3)
Texas	10,604	10.0	0.5	(9.1–10.9)
Utah	12,591	10.4	0.4	(9.7–11.1)
Vermont	6,303	9.7	0.6	(8.7–10.8)
Virginia	8,311	10.5	0.5	(9.5–11.4)
Washington	11,018	11.7	0.5	(10.8–12.6)
West Virginia	5,798	15.2	0.6	(14.0–16.4)
Wisconsin	6,525	10.4	0.7	(9.0–11.7)
Wyoming	6,356	9.8	0.6	(8.6–11.0)
Guam	1,879	10.1	0.9	(8.4–11.8)
Puerto Rico	5,959	13.8	0.6	(12.6–15.0)
*Median*		*11.3*		
*Range *		*7.7–15.2*		

**Abbreviations:** CI = confidence interval; SE = standard error.

* Age adjusted to the 2000 U.S. standard population.

^†^ Respondents were asked “How many days in the past month was your mental health (including stress, depression, and problems with emotions) not good?”

**TABLE 6:** Age-adjusted* prevalence estimates of adults aged ≥18 years who reported ≥14 days of frequent mental distress during the past 30 days,^†^ by metropolitan and micropolitan statistical area — Behavioral Risk Factor Surveillance System, United States, 2013

MMSA	Sample size	%	SE	95% CI
Aguadilla-Isabela, Puerto Rico	586	12.6	1.8	(9.1–16.0)
Akron, Ohio	680	19.4	2.9	(13.7–25.1)
Albuquerque, New Mexico	2,055	12.4	1.0	(10.4–14.4)
Allentown-Bethlehem-Easton, Pennsylvania-New Jersey	1,011	12.9	1.8	(9.4–16.4)
Anchorage, Alaska	1,501	8.9	1.0	(6.9–10.8)
Atlanta-Sandy Springs-Roswell, Georgia	3,451	9.2	0.7	(7.9–10.5)
Augusta-Richmond County, Georgia-South Carolina	893	16.3	2.4	(11.6–21.0)
Austin-Round Rock, Texas	915	11.7	1.5	(8.8–14.6)
Baltimore-Columbia-Towson, Maryland	4,679	11.0	0.8	(9.4–12.5)
Baton Rouge, Louisiana	914	8.8	1.3	(6.2–11.4)
Billings, Montana	813	10.2	1.2	(7.9–12.6)
Birmingham-Hoover, Alabama	1,335	14.0	1.2	(11.5–16.4)
Bismarck, North Dakota	1,011	7.5	1.2	(5.1–9.8)
Boise City, Idaho	1,470	10.8	1.1	(8.6–13.1)
Boston, Massachusetts^§^	4,003	11.9	0.9	(10.2–13.7)
Buffalo-Cheektowaga-Niagara Falls, New York	497	11.4	2.2	(7.1–15.8)
Burlington-South Burlington, Vermont	1,617	8.7	0.9	(7.0–10.4)
Cambridge-Newton-Framingham, Massachusetts^§^	4,821	10.6	0.8	(9.1–12.2)
Camden, New Jersey^§^	1,823	11.0	1.0	(9.0–13.0)
Cedar Rapids, Iowa	641	10.0	1.9	(6.2–13.8)
Charleston, West Virginia	810	15.2	1.6	(12.1–18.4)
Charleston-North Charleston, South Carolina	1,527	10.6	1.1	(8.4–12.8)
Charlotte-Concord-Gastonia, North Carolina-South Carolina	1,918	12.0	1.1	(9.9–14.1)
Chattanooga, Tennessee-Georgia	572	11.1	2.1	(7.0–15.2)
Chicago-Naperville-Elgin, Illinois-Indiana-Wisconsin	3,308	12.4	0.9	(10.7–14.1)
Cincinnati, Ohio-Kentucky-Indiana	2,566	10.8	1.0	(8.8–12.8)
Claremont-Lebanon, New Hampshire-Vermont	1,656	10.0	1.2	(7.7–12.3)
Cleveland-Elyria, Ohio	1,099	9.4	1.2	(7.1–11.7)
Colorado Springs, Colorado	1,361	11.5	1.2	(9.2–13.8)
Columbia, South Carolina	1,411	9.6	1.1	(7.4–11.7)
Columbus, Ohio	1,834	13.5	1.1	(11.4–15.6)
Crestview-Fort Walton Beach-Destin, Florida	1,052	10.8	1.3	(8.2–13.5)
Dallas-Plano-Irving, Texas^§^	877	9.7	1.3	(7.1–12.2)
Davenport-Moline-Rock Island, Iowa-Illinois	665	10.1	1.9	(6.5–13.8)
Dayton, Ohio	829	12.4	1.7	(9.0–15.8)
Deltona-Daytona Beach-Ormond Beach, Florida	1,082	15.4	2.1	(11.2–19.5)
Denver-Aurora-Lakewood, Colorado	5,633	9.0	0.5	(8.1–10.0)
Des Moines-West Des Moines, Iowa	1,332	10.4	1.2	(8.0–12.8)
Duluth, Minnesota-Wisconsin	694	11.2	2.2	(6.9–15.5)
Durham-Chapel Hill, North Carolina	606	7.3	1.7	(4.0–10.6)
El Paso, Texas	730	9.4	1.3	(6.8–11.9)
Evansville, Indiana-Kentucky	564	14.7	2.2	(10.3–19.1)
Fargo, North Dakota-Minnesota	1,164	10.6	1.8	(7.1–14.1)
Fayetteville-Springdale-Rogers, Arkansas-Missouri	808	15.8	2.2	(11.4–20.2)
Fort Smith, Arkansas-Oklahoma	490	17.6	2.5	(12.8–22.4)
Fort Wayne, Indiana	766	10.9	1.7	(7.5–14.2)
Fort Worth-Arlington, Texas^§^	793	10.6	1.6	(7.5–13.8)
Gainesville, Florida	992	11.5	1.7	(8.3–14.7)
Grand Forks, North Dakota-Minnesota	495	6.3	1.5	(3.4–9.2)
Grand Island, Nebraska	785	12.2	1.9	(8.5–15.9)
Grand Rapids-Wyoming, Michigan	1,334	10.7	1.3	(8.2–13.2)
Greensboro-High Point, North Carolina	655	10.5	1.5	(7.4–13.5)
Greenville-Anderson-Mauldin, South Carolina	1,327	13.5	1.4	(10.7–16.2)
Gulfport-Biloxi-Pascagoula, Mississippi	756	16.6	1.9	(12.8–20.3)
Hagerstown-Martinsburg, Maryland-West Virginia	754	11.2	1.5	(8.2–14.2)
Hartford-West Hartford-East Hartford, Connecticut	2,811	10.2	0.9	(8.5–11.9)
Hilton Head Island-Bluffton-Beaufort, South Carolina	817	10.4	2.1	(6.4–14.5)
Houston-The Woodlands-Sugar Land, Texas	1,359	8.9	1.1	(6.8–11.0)
Huntington-Ashland, West Virginia-Kentucky-Ohio	1,155	16.2	1.5	(13.3–19.1)
Idaho Falls, Idaho	499	13.5	2.2	(9.2–17.7)
Indianapolis-Carmel-Anderson, Indiana	2,490	11.7	0.9	(10.0–13.4)
Jackson, Mississippi	795	11.5	1.5	(8.5–14.4)
Jacksonville, Florida	2,820	13.7	1.0	(11.7–15.7)
Kansas City, Missouri-Kansas	7,350	10.4	0.8	(8.8–11.9)
Kingsport-Bristol-Bristol, Tennessee-Virginia	524	18.0	3.0	(12.1–23.9)
Knoxville, Tennessee	643	14.6	1.9	(11.0–18.3)
Lansing-East Lansing, Michigan	680	11.3	1.6	(8.3–14.4)
Lexington-Fayette, Kentucky	633	12.3	1.6	(9.1–15.5)
Lincoln, Nebraska	1,851	9.3	0.9	(7.6–11.0)
Little Rock-North Little Rock-Conway, Arkansas	1,120	13.5	1.5	(10.6–16.5)
Logan, Utah-Idaho	628	9.9	1.6	(6.7–13.1)
Los Angeles-Long Beach-Anaheim, California	3,011	10.6	0.8	(9.1–12.1)
Louisville/Jefferson County, Kentucky-Indiana	2,120	14.3	1.3	(11.7–16.8)
Lubbock, Texas	520	9.0	2.0	(5.1–13.0)
Manhattan, Kansas	651	9.9	1.4	(7.2–12.6)
Memphis, Tennessee-Mississippi-Arkansas	1,185	8.5	1.1	(6.4–10.6)
Miami-Fort Lauderdale-West Palm Beach, Florida	2,146	13.0	1.2	(10.6–15.4)
Milwaukee-Waukesha-West Allis, Wisconsin	1,251	14.0	1.9	(10.3–17.7)
Minneapolis-St. Paul-Bloomington, Minnesota-Wisconsin	9,016	8.1	0.5	(7.1–9.2)
Minot, North Dakota	638	6.3	1.5	(3.4–9.2)
Montgomery County-Bucks County-Chester County, Pennsylvania^§^	957	11.7	1.4	(8.9–14.4)
Myrtle Beach-Conway-North Myrtle Beach, South Carolina-North Carolina	762	12.0	1.8	(8.4–15.6)
Nashville-Davidson County-Murfreesboro-Franklin, Tennessee	1,043	9.5	1.3	(7.0–12.0)
Nassau County-Suffolk County, New York^§^	916	8.8	1.1	(6.7–11.0)
Newark, New Jersey-Pennsylvania^§^	4,005	10.1	0.8	(8.6–11.6)
New Orleans-Metairie, Louisiana	1,267	16.1	2.0	(12.2–20.1)
New York-Jersey City-White Plains, New York-New Jersey^§^	8,688	11.0	0.6	(9.9–12.1)
Norfolk, Nebraska	664	9.3	1.4	(6.6–11.9)
North Platte, Nebraska	704	8.9	1.9	(5.2–12.5)
North Port-Sarasota-Bradenton, Florida	1,061	13.8	1.7	(10.5–17.1)
Oakland-Hayward-Berkeley, California^§^	694	9.9	1.5	(6.8–12.9)
Ogden-Clearfield, Utah	2,430	10.0	0.7	(8.5–11.5)
Oklahoma City, Oklahoma	2,612	11.8	0.9	(10.1–13.4)
Omaha-Council Bluffs, Nebraska-Iowa	3,090	8.9	0.7	(7.6–10.3)
Orlando-Kissimmee-Sanford, Florida	2,231	11.1	1.0	(9.0–13.1)
Panama City, Florida	1,001	15.3	1.9	(11.7–19.0)
Pensacola-Ferry Pass-Brent, Florida	1,291	10.5	1.1	(8.4–12.6)
Philadelphia, Pennsylvania^§^	1,733	14.1	1.3	(11.7–16.6)
Phoenix-Mesa-Scottsdale, Arizona	1,521	13.0	1.5	(10.1–15.9)
Pittsburgh, Pennsylvania	2,324	12.3	1.0	(10.3–14.3)
Ponce, Puerto Rico	528	12.8	2.2	(8.5–17.1)
Portland-South Portland, Maine	2,597	13.5	1.1	(11.4–15.5)
Portland-Vancouver-Hillsboro, Oregon-Washington	3,205	12.0	0.8	(10.4–13.6)
Port St. Lucie, Florida	996	12.3	1.9	(8.6–16.0)
Providence-Warwick, Rhode Island-Massachusetts	8,165	12.9	0.8	(11.4–14.4)
Provo-Orem, Utah	1,832	8.6	0.8	(7.1–10.1)
Raleigh, North Carolina	667	9.2	1.2	(6.8–11.6)
Rapid City, South Dakota	865	9.6	1.4	(6.8–12.4)
Reno, Nevada	1,783	11.9	1.2	(9.5–14.3)
Richmond, Virginia	1,293	11.5	1.3	(9.0–14.0)
Riverside-San Bernardino-Ontario, California	1,355	13.4	1.2	(10.9–15.8)
Rochester, New York	498	10.7	1.8	(7.3–14.1)
Rockingham County-Strafford County, New Hampshire^§^	1,641	10.7	1.1	(8.5–12.9)
Sacramento-Roseville-Arden-Arcade, California	885	14.6	1.9	(11.0–18.3)
St. Louis, Missouri-Illinois	2,038	10.7	1.0	(8.7–12.6)
Salem, Oregon	520	12.1	2.5	(7.2–17.1)
Salina, Kansas	519	9.2	1.7	(5.7–12.6)
Salisbury, Maryland-Delaware	2,029	9.9	1.2	(7.5–12.3)
Salt Lake City, Utah	4,626	10.6	0.6	(9.5–11.7)
San Antonio-New Braunfels, Texas	918	10.6	1.4	(7.8–13.3)
San Francisco-Redwood City-South San Francisco, California	532	10.0	1.7	(6.7–13.3)
San Jose-Sunnyvale-Santa Clara, California	622	8.2	1.4	(5.4–11.0)
San Juan-Carolina-Caguas, Puerto Rico	3,626	14.3	0.8	(12.8–15.9)
Scottsbluff, Nebraska	703	8.7	1.4	(5.9–11.5)
Scranton-Wilkes-Barre-Hazleton, Pennsylvania	554	12.8	2.1	(8.6–17.0)
Seattle-Bellevue-Everett, Washington^§^	3,734	11.1	0.7	(9.7–12.6)
Shreveport-Bossier City, Louisiana	561	10.7	1.9	(7.0–14.5)
Silver Spring-Frederick-Rockville, Maryland^§^	2,385	8.1	1.0	(6.2–10.1)
Sioux City, Iowa-Nebraska-South Dakota	1,041	7.1	1.5	(4.1–10.0)
Sioux Falls, South Dakota	1,003	6.5	1.3	(4.1–9.0)
Spartanburg, South Carolina	580	8.7	1.7	(5.5–12.0)
Spokane-Spokane Valley, Washington	851	10.7	1.6	(7.6–13.9)
Springfield, Massachusetts	1,541	13.1	1.6	(10.0–16.2)
Tallahassee, Florida	1,805	9.6	1.2	(7.3–11.9)
Tampa-St. Petersburg-Clearwater, Florida	2,163	13.9	1.2	(11.6–16.1)
Toledo, Ohio	986	12.7	1.8	(9.2–16.2)
Topeka, Kansas	2,378	10.4	0.9	(8.7–12.1)
Tulsa, Oklahoma	1,973	14.6	1.2	(12.4–16.9)
Virginia Beach-Norfolk-Newport News, Virginia-North Carolina	1,657	9.5	1.0	(7.5–11.5)
Warren-Troy-Farmington Hills, Michigan^§^	2,237	9.7	0.9	(8.1–11.4)
Washington-Arlington-Alexandria, District of Columbia-Virginia-Maryland-West Virginia^§^	8,870	8.7	0.6	(7.5–10.0)
Wichita, Kansas	4,867	10.5	0.6	(9.3–11.7)
Wilmington, Delaware-Maryland-New Jersey^§^	3,233	11.7	0.8	(10.1–13.2)
Winston-Salem, North Carolina	684	16.8	2.4	(12.1–21.4)
Worcester, Massachusetts-Connecticut	2,712	12.0	1.1	(9.7–14.2)
*Median*		*10.9*		
*Range*		*6.3–19.4*		

**Abbreviations:** CI = confidence interval; MMSA = metropolitan and micropolitan statistical areas; SE = standard error.

* Age adjusted to the 2000 U.S. standard population.

^†^ Respondents were asked “How many days in the past month was your mental health (including stress, depression, and problems with emotions) not good?”

^§^ Metropolitan division.

#### Frequent Physical Distress 

Frequent physical distress included respondents who reported ≥14 days of poor physical health (e.g., physical illness or injury) during the past 30 days. In 2014, age-adjusted prevalence estimates of frequent physical distress ranged from 7.8% in North Dakota to 16.0% in Kentucky (median: 10.9%) (Table 7). Among selected MMSA, age-adjusted prevalence estimates ranged from 6.2% in Logan, Utah-Idaho, to 18.5% in Aguadilla-Isabela, Puerto Rico (median: 11.1%) (Table 8).

**TABLE 7:** Age-adjusted* prevalence estimates of adults aged ≥18 years who reported ≥14 days of frequent physical distress^†^ during the past 30 days, by state/territory — Behavioral Risk Factor Surveillance System, United States, 2014

State/Territory	Sample size	%	SE	95% CI
Alabama	8,394	14.6	0.5	(13.6–15.6)
Alaska	4,248	10.1	0.6	(8.8–11.3)
Arizona	14,540	11.9	0.4	(11.1–12.8)
Arkansas	5,088	14.6	0.7	(13.1–16.0)
California	8,755	12.5	0.5	(11.6–13.4)
Colorado	13,153	9.5	0.3	(8.9–10.1)
Connecticut	7,835	9.2	0.5	(8.2–10.1)
Delaware	4,178	10.5	0.6	(9.3–11.8)
District of Columbia	3,956	9.3	0.8	(7.8–10.8)
Florida	9,430	11.7	0.5	(10.8–12.6)
Georgia	6,211	11.9	0.5	(10.9–12.9)
Hawaii	7,196	8.4	0.5	(7.4–9.3)
Idaho	5,327	9.3	0.5	(8.3–10.3)
Illinois	5,018	11.1	0.6	(9.9–12.3)
Indiana	11,152	12.6	0.4	(11.8–13.4)
Iowa	7,993	9.5	0.4	(8.7–10.4)
Kansas	13,389	9.6	0.3	(9.0–10.1)
Kentucky	10,967	16.0	0.6	(14.8–17.3)
Louisiana	6,614	13.5	0.5	(12.5–14.5)
Maine	8,885	10.9	0.5	(10.0–11.8)
Maryland	12,278	9.9	0.5	(8.9–11.0)
Massachusetts	15,234	10.2	0.4	(9.4–11.0)
Michigan	8,340	11.8	0.5	(10.9–12.8)
Minnesota	16,135	8.2	0.3	(7.7–8.7)
Mississippi	4,098	12.9	0.7	(11.5–14.2)
Missouri	6,976	12.4	0.5	(11.4–13.5)
Montana	7,337	11.7	0.6	(10.6–12.8)
Nebraska	22,024	8.6	0.3	(8.0–9.1)
Nevada	3,675	10.9	0.8	(9.4–12.4)
New Hampshire	6,073	8.9	0.5	(7.9–9.9)
New Jersey	12,733	9.9	0.4	(9.1–10.6)
New Mexico	8,775	13.5	0.6	(12.4–14.6)
New York	6,631	10.8	0.5	(9.8–11.7)
North Carolina	7,134	11.8	0.5	(10.9–12.7)
North Dakota	7,560	7.8	0.5	(6.9–8.7)
Ohio	10,661	11.9	0.5	(10.9–12.9)
Oklahoma	8,291	13.8	0.5	(12.9–14.7)
Oregon	5,093	13.3	0.6	(12.0–14.5)
Pennsylvania	10,668	11.3	0.4	(10.5–12.1)
Rhode Island	6,316	10.9	0.6	(9.8–12.0)
South Carolina	10,693	12.3	0.4	(11.5–13.1)
South Dakota	7,299	9.1	0.6	(7.9–10.3)
Tennessee	4,965	14.2	0.7	(12.9–15.5)
Texas	14,846	10.8	0.4	(10.0–11.6)
Utah	14,816	9.9	0.3	(9.4–10.5)
Vermont	6,377	10.0	0.5	(9.1–10.9)
Virginia	9,239	10.4	0.4	(9.6–11.2)
Washington	9,928	11.5	0.5	(10.6–12.4)
West Virginia	6,108	15.6	0.5	(14.6–16.7)
Wisconsin	6,985	11.1	0.6	(9.9–12.2)
Wyoming	6,292	9.8	0.6	(8.7–11.0)
Guam	2,493	9.1	0.8	(7.5–10.8)
Puerto Rico	5,959	15.2	0.6	(14.1–16.3)
*Median*		*10.9*		
*Range*		*7.8–16.0*		

**Abbreviations**: CI = confidence interval; SE = standard error.

* Age adjusted to the 2000 U.S. standard population.

^†^ Respondents were asked “How many days in the past month was your physical health (including physical illness and injury) not good?”

**TABLE 8:** Age-adjusted* prevalence estimates of adults aged ≥18 years who reported ≥14 days of frequent physical distress^†^ during the past 30 days, by metropolitan and micropolitan statistical area — Behavioral Risk Factor Surveillance System, United States, 2014

MMSA	Sample size	%	SE	95% CI
Aberdeen, South Dakota	614	8.7	1.5	(5.8–11.5)
Aguadilla-Isabela, Puerto Rico	544	18.5	2.0	(14.5–22.5)
Albuquerque, New Mexico	1,771	12.8	1.1	(10.7–14.9)
Allentown-Bethlehem-Easton, Pennsylvania-New Jersey	1,060	10.1	1.3	(7.5–12.7)
Anchorage, Alaska	1,737	9.7	0.9	(7.9–11.5)
Atlanta-Sandy Springs-Roswell, Georgia	2,725	10.1	0.7	(8.7–11.4)
Augusta-Richmond County, Georgia-South Carolina	863	14.7	2.6	(9.6–19.7)
Austin-Round Rock, Texas	2,195	8.8	0.7	(7.4–10.2)
Baltimore-Columbia-Towson, Maryland	4,509	10.1	0.9	(8.4–11.9)
Baton Rouge, Louisiana	900	11.3	1.2	(9.0–13.6)
Berlin, New Hampshire-Vermont	534	12.6	2.8	(7.1–18.1)
Billings, Montana	796	13.4	1.7	(10.2–16.7)
Birmingham-Hoover, Alabama	1,543	11.9	0.9	(10.0–13.7)
Bismarck, North Dakota	1,009	6.9	0.9	(5.1–8.7)
Boise City, Idaho	1,315	7.7	0.9	(5.9–9.4)
Boston, Massachusetts^§^	4,452	8.8	0.7	(7.5–10.2)
Burlington-South Burlington, Vermont	1,960	8.3	0.7	(6.9–9.8)
Cambridge-Newton-Framingham, Massachusetts^§^	5,026	9.6	0.6	(8.3–10.8)
Camden, New Jersey^§^	1,684	10.5	1.1	(8.4–12.7)
Cedar Rapids, Iowa	624	11.0	1.6	(7.8–14.2)
Charleston, West Virginia	865	16.7	1.5	(13.7–19.6)
Charleston-North Charleston, South Carolina	1,370	10.3	1.2	(8.0–12.6)
Charlotte-Concord-Gastonia, North Carolina-South Carolina	2,116	11.7	0.9	(10.0–13.5)
Chicago-Naperville-Elgin, Illinois-Indiana-Wisconsin	4,080	11.2	0.7	(9.8–12.7)
Cincinnati, Ohio-Kentucky-Indiana	2,017	12.4	1.1	(10.1–14.6)
Claremont-Lebanon, New Hampshire-Vermont	1,654	9.7	1.1	(7.6–11.8)
Cleveland-Elyria, Ohio	949	11.3	1.6	(8.2–14.4)
College Station-Bryan, Texas	556	12.3	2.7	(7.1–17.5)
Colorado Springs, Colorado	1,275	10.4	1.1	(8.2–12.6)
Columbia, South Carolina	1,184	11.1	1.0	(9.1–13.1)
Columbus, Ohio	1,626	10.9	1.0	(8.8–12.9)
Corpus Christi, Texas	588	12.1	2.0	(8.1–16.1)
Dallas-Plano-Irving, Texas^§^	1,255	8.9	1.1	(6.7–11.1)
Dayton, Ohio	552	13.9	2.1	(9.9–18.0)
Denver-Aurora-Lakewood, Colorado	5,724	9.0	0.4	(8.1–9.8)
Des Moines-West Des Moines, Iowa	1,343	9.7	1.1	(7.6–11.9)
Duluth, Minnesota-Wisconsin	931	13.2	1.6	(10.2–16.3)
El Paso, Texas	679	11.0	1.3	(8.4–13.6)
Evansville, Indiana-Kentucky	635	16.7	2.9	(11.0–22.3)
Fargo, North Dakota-Minnesota	1,131	7.4	0.9	(5.6–9.1)
Fayetteville-Springdale-Rogers, Arkansas-Missouri	796	12.7	1.7	(9.4–16.0)
Fort Wayne, Indiana	843	11.7	1.8	(8.1–15.3)
Fort Worth-Arlington, Texas^§^	734	10.5	1.7	(7.2–13.8)
Grand Island, Nebraska	1,038	9.5	1.4	(6.8–12.3)
Grand Rapids-Wyoming, Michigan	883	10.7	1.4	(8.0–13.3)
Greensboro-High Point, North Carolina	506	12.7	2.4	(8.0–17.5)
Greenville-Anderson-Mauldin, South Carolina	1,459	11.6	1.0	(9.6–13.6)
Hagerstown-Martinsburg, Maryland-West Virginia	766	17.2	2.9	(11.6–22.9)
Hartford-West Hartford-East Hartford, Connecticut	2,600	9.6	0.8	(8.0–11.2)
Hilton Head Island-Bluffton-Beaufort, South Carolina	542	11.6	2.0	(7.7–15.6)
Houston-The Woodlands-Sugar Land, Texas	2,080	8.9	1.0	(7.0–10.8)
Huntington-Ashland, West Virginia-Kentucky-Ohio	1,227	15.3	1.2	(12.8–17.7)
Idaho Falls, Idaho	507	11.4	1.7	(8.0–14.8)
Indianapolis-Carmel-Anderson, Indiana	3,505	12.0	0.7	(10.5–13.4)
Jacksonville, Florida	650	10.5	1.5	(7.5–13.5)
Kansas City, Missouri-Kansas	4,780	10.8	0.8	(9.3–12.3)
Kingsport-Bristol-Bristol, Tennessee-Virginia	488	16.9	2.3	(12.3–21.5)
Knoxville, Tennessee	546	15.4	2.1	(11.3–19.6)
Lafayette, Louisiana	545	14.0	1.8	(10.4–17.6)
Lexington-Fayette, Kentucky	616	11.1	1.7	(7.9–14.4)
Lincoln, Nebraska	1,987	7.7	0.7	(6.3–9.2)
Little Rock-North Little Rock-Conway, Arkansas	1,153	11.4	1.3	(8.8–13.9)
Logan, Utah-Idaho	620	6.2	1.0	(4.2–8.1)
Los Angeles-Long Beach-Anaheim, California	2,421	12.5	0.8	(10.8–14.1)
Louisville-Jefferson County, Kentucky-Indiana	2,404	13.4	1.3	(10.8–15.9)
Madison, Wisconsin	549	9.0	1.5	(6.0–12.0)
Memphis, Tennessee-Mississippi-Arkansas	862	10.6	1.8	(7.1–14.0)
Miami-Fort Lauderdale-West Palm Beach, Florida	2,118	11.0	0.9	(9.2–12.7)
Milwaukee-Waukesha-West Allis, Wisconsin	1,346	12.2	1.3	(9.6–14.8)
Minneapolis-St. Paul-Bloomington, Minnesota-Wisconsin	8,631	7.7	0.4	(7.0–8.4)
Minot, North Dakota	585	7.5	1.3	(5.0–10.0)
Montgomery, Alabama	497	14.6	1.9	(10.9–18.3)
Montgomery County-Bucks County-Chester County, Pennsylvania^§^	791	7.7	1.2	(5.4–10.0)
Myrtle Beach-Conway-North Myrtle Beach, South Carolina-North Carolina	979	16.6	1.6	(13.4–19.8)
Nashville-Davidson County-Murfreesboro-Franklin, Tennessee	786	14.1	1.5	(11.1–17.0)
Nassau County-Suffolk County, New York^§^	752	9.4	1.6	(6.2–12.6)
Newark, New Jersey-Pennsylvania^§^	4,060	9.1	0.7	(7.8–10.4)
New Orleans-Metairie, Louisiana	1,873	12.1	0.9	(10.4–13.9)
New York-Jersey City-White Plains, New York-New Jersey^§^	7,307	11.2	0.5	(10.2–12.3)
Norfolk, Nebraska	980	9.0	1.1	(6.9–11.1)
North Platte, Nebraska	945	11.7	1.3	(9.1–14.2)
North Port-Sarasota-Bradenton, Florida	497	13.0	2.9	(7.3–18.7)
Oakland-Hayward-Berkeley, California^§^	698	12.2	1.5	(9.2–15.2)
Ogden-Clearfield, Utah	2,890	10.3	0.7	(9.0–11.7)
Oklahoma City, Oklahoma	2,403	13.4	0.8	(11.7–15.0)
Omaha-Council Bluffs, Nebraska-Iowa	4,803	8.3	0.5	(7.3–9.4)
Orlando-Kissimmee-Sanford, Florida	923	12.8	1.4	(10.1–15.5)
Philadelphia, Pennsylvania^§^	1,462	14.4	1.3	(11.9–16.8)
Phoenix-Mesa-Scottsdale, Arizona	9,218	11.7	0.5	(10.7–12.7)
Pittsburgh, Pennsylvania	2,335	11.9	0.8	(10.3–13.5)
Ponce, Puerto Rico	525	15.3	2.1	(11.2–19.4)
Portland-South Portland, Maine	2,694	8.7	0.8	(7.2–10.2)
Portland-Vancouver-Hillsboro, Oregon-Washington	2,786	11.6	0.8	(10.1–13.2)
Providence-Warwick, Rhode Island-Massachusetts	7,914	11.6	0.6	(10.4–12.8)
Provo-Orem, Utah	2,127	8.5	0.7	(7.2–9.8)
Raleigh, North Carolina	712	10.0	1.3	(7.5–12.6)
Rapid City, South Dakota	1,408	10.6	1.3	(8.1–13.2)
Reno, Nevada	1,164	10.6	1.1	(8.4–12.9)
Richmond, Virginia	1,437	10.2	1.0	(8.1–12.2)
Riverside-San Bernardino-Ontario, California	932	15.6	1.5	(12.6–18.6)
Roanoke, Virginia	522	9.1	1.5	(6.2–12.1)
Rochester, Minnesota	691	7.4	1.3	(4.8–9.9)
Rockingham County-Strafford County, New Hampshire^§^	1,424	8.5	0.8	(6.9–10.2)
Sacramento-Roseville-Arden-Arcade, California	641	12.8	1.6	(9.7–15.9)
St. Cloud, Minnesota	556	7.5	1.3	(5.0–10.1)
St. Louis, Missouri-Illinois	1,906	11.2	1.0	(9.2–13.2)
Salisbury, Maryland-Delaware	1,893	10.8	1.3	(8.3–13.3)
Salt Lake City, Utah	5,340	10.2	0.5	(9.3–11.1)
San Antonio-New Braunfels, Texas	2,194	10.8	0.9	(9.1–12.5)
San Juan-Carolina-Caguas, Puerto Rico	3,729	14.8	0.7	(13.5–16.2)
Scottsbluff, Nebraska	892	13.9	1.7	(10.7–17.2)
Seattle-Bellevue-Everett, Washington^§^	3,646	10.0	0.6	(8.8–11.3)
Shreveport-Bossier City, Louisiana	535	16.2	2.1	(12.2–20.3)
Silver Spring-Frederick-Rockville, Maryland^§^	2,331	7.7	1.0	(5.7–9.6)
Sioux City, Iowa-Nebraska-South Dakota	1,111	12.8	2.1	(8.6–17.0)
Sioux Falls, South Dakota	1,318	7.3	1.2	(5.0–9.7)
Spartanburg, South Carolina	537	11.0	1.5	(8.1–13.9)
Spokane-Spokane Valley, Washington	716	11.3	1.5	(8.4–14.2)
Springfield, Massachusetts	1,080	17.3	2.0	(13.5–21.2)
Tampa-St. Petersburg-Clearwater, Florida	1,523	11.8	1.1	(9.7–13.9)
Toledo, Ohio	631	14.7	2.1	(10.6–18.7)
Topeka, Kansas	1,408	9.0	1.0	(7.1–10.9)
Tulsa, Oklahoma	2,006	12.8	0.9	(11.0–14.7)
Tuscaloosa, Alabama	704	14.9	1.6	(11.7–18.1)
Virginia Beach-Norfolk-Newport News, Virginia-North Carolina	1,839	10.0	0.9	(8.2–11.9)
Warren-Troy-Farmington Hills, Michigan^§^	2,094	9.7	0.7	(8.2–11.1)
Washington-Arlington-Alexandria, District of Columbia-Virginia-Maryland-West Virginia^§^	8,109	8.4	0.6	(7.3–9.5)
Wichita, Kansas	2,675	10.3	0.7	(9.0–11.6)
Wichita Falls, Texas	521	N/A^¶^	N/A^¶^	(N/A–N/A)^¶^
Wilmington, Delaware-Maryland-New Jersey^§^	2,705	11.5	0.9	(9.8–13.2)
Worcester, Massachusetts-Connecticut	2,406	10.5	0.9	(8.6–12.3)
Youngstown-Warren-Boardman, Ohio-Pennsylvania	512	13.2	2.0	(9.3–17.1)
*Median*		*11.1*		
*Range*		*6.2–18.5*		

**Abbreviations**: CI = confidence interval; MMSA = metropolitan and micropolitan statistical areas; N/A = not available; SE = standard error.

* Age adjusted to the 2000 U.S. standard population.

^†^ Respondents were asked “How many days in the past month was your physical health (including physical illness and injury) not good?”

^§^ Metropolitan division.

^¶^ Estimate not available if the unweighted sample size for the denominator was <50 or if the relative standard error is >30%.

### Health Care Access and Preventive Practices

#### Health Care Coverage

Health care coverage was defined as respondents aged 18–64 years having any form of coverage, including private health insurance, prepaid plans (e.g., health maintenance organizations), or a government plan (e.g., Medicare or Medicaid) at the time of the interview. In 2013, age-adjusted prevalence estimates of health care coverage ranged from 66.9% in Texas to 92.4% in Massachusetts (median: 79.6%) (Table 9). Among selected MMSA, estimated age-adjusted prevalence ranged from 60.5% in El Paso, Texas, to 97.6% in Aguadilla-Isabela, Puerto Rico (median: 80.6%) (Table 10). In 2014, age-adjusted prevalence estimates ranged from 70.5% in Texas to 94.5% in Massachusetts (median: 84.2%) (Table 11). Among selected MMSA, age-adjusted prevalence estimates ranged from 64.7% in El Paso, Texas, to 96.6% in Ponce, Puerto Rico (median: 84.3%) (Table 12).

**TABLE 9:** Age-adjusted* prevalence estimates of adults aged 18–64 years who have health care coverage,^†^ by state/territory — Behavioral Risk Factor Surveillance System, United States, 2013

State/Territory	Sample size	%	SE	95% CI
Alabama	4,031	77.9	1.0	(75.8–79.9)
Alaska	3,643	79.5	1.0	(77.6–81.5)
Arizona	2,615	74.6	1.6	(71.4–77.7)
Arkansas	3,127	70.8	1.2	(68.5–73.2)
California	8,376	79.4	0.6	(78.2–80.6)
Colorado	9,377	79.6	0.6	(78.5–80.8)
Connecticut	5,240	87.5	0.7	(86.0–88.9)
Delaware	3,351	85.0	0.9	(83.3–86.7)
District of Columbia	3,150	90.2	0.9	(88.4–92.0)
Florida	19,195	70.1	0.7	(68.6–71.5)
Georgia	5,729	72.2	0.8	(70.5–73.8)
Hawaii	5,519	89.7	0.6	(88.5–90.8)
Idaho	3,607	75.6	1.1	(73.5–77.7)
Illinois	3,744	80.0	1.1	(77.9–82.1)
Indiana	6,704	78.5	0.7	(77.1–79.9)
Iowa	5,148	86.7	0.7	(85.3–88.1)
Kansas	15,459	78.5	0.4	(77.7–79.4)
Kentucky	7,462	77.1	0.8	(75.6–78.7)
Louisiana	3,316	73.4	1.4	(70.8–76.1)
Maine	5,220	84.2	0.8	(82.7–85.7)
Maryland	8,531	83.7	0.7	(82.3–85.1)
Massachusetts	10,292	92.4	0.4	(91.6–93.3)
Michigan	8,411	81.7	0.6	(80.5–83.0)
Minnesota	10,056	86.8	0.6	(85.6–88.0)
Mississippi	4,645	71.4	1.0	(69.4–73.4)
Missouri	4,387	80.5	0.9	(78.8–82.2)
Montana	6,437	78.0	0.7	(76.6–79.5)
Nebraska	10,939	81.8	0.7	(80.5–83.1)
Nevada	3,431	71.9	1.5	(69.1–74.8)
New Hampshire	4,221	83.7	0.9	(82.0–85.4)
New Jersey	9,674	80.0	0.7	(78.7–81.3)
New Mexico	6,334	73.0	0.8	(71.4–74.7)
New York	6,242	82.3	0.7	(80.9–83.6)
North Carolina	5,818	75.2	0.8	(73.7–76.7)
North Dakota	5,271	87.4	0.7	(86.0–88.8)
Ohio	8,077	82.8	0.7	(81.5–84.2)
Oklahoma	5,239	76.9	0.8	(75.3–78.4)
Oregon	3,847	74.3	1.1	(72.2–76.4)
Pennsylvania	7,473	84.1	0.6	(82.9–85.3)
Rhode Island	4,367	80.7	0.9	(78.9–82.5)
South Carolina	6,745	75.4	0.8	(73.9–76.9)
South Dakota	4,673	84.6	0.9	(82.7–86.4)
Tennessee	3,788	78.2	1.0	(76.2–80.2)
Texas	7,305	66.9	0.8	(65.3–68.5)
Utah	9,368	81.0	0.6	(79.9–82.1)
Vermont	4,232	87.8	0.7	(86.4–89.3)
Virginia	5,907	81.0	0.7	(79.6–82.5)
Washington	7,273	78.7	0.7	(77.4–80.1)
West Virginia	4,068	75.0	0.9	(73.2–76.8)
Wisconsin	4,496	85.2	0.9	(83.5–87.0)
Wyoming	3,845	75.7	1.0	(73.7–77.8)
Guam	1,667	72.3	1.4	(69.5–75.1)
Puerto Rico	3,974	91.5	0.6	(90.3–92.6)
*Median*		*79.6*		
*Range*		*66.9–92.4*		

**Abbreviations**: CI = confidence interval; SE = standard error.

* Age adjusted to the 2000 U.S. standard population.

^†^ Including health insurance, prepaid plans (e.g., health maintenance organizations), or government plans (e.g., Medicare).

**TABLE 10:** Age-adjusted* prevalence estimates of adults aged 18–64 years who have health care coverage,^†^ by metropolitan and micropolitan statistical area — Behavioral Risk Factor Surveillance System, United States, 2013

MMSA	Sample size	%	SE	95% CI
Aguadilla-Isabela, Puerto Rico	388	97.6	0.9	(95.8–99.4)
Akron, Ohio	426	87.7	2.1	(83.5–91.9)
Albuquerque, New Mexico	1,511	75.4	1.6	(72.4–78.5)
Allentown-Bethlehem-Easton, Pennsylvania-New Jersey	732	83.8	2.3	(79.2–88.4)
Anchorage, Alaska	1,216	80.9	1.5	(77.9–83.9)
Atlanta-Sandy Springs-Roswell, Georgia	2,631	73.9	1.2	(71.4–76.3)
Augusta-Richmond County, Georgia-South Carolina	584	81.8	2.4	(77.1–86.6)
Austin-Round Rock, Texas	672	74.1	2.3	(69.6–78.6)
Baltimore-Columbia-Towson, Maryland	3,166	85.5	1.1	(83.4–87.7)
Baton Rouge, Louisiana	614	76.3	2.9	(70.6–81.9)
Billings, Montana	604	80.6	1.9	(76.8–84.4)
Birmingham-Hoover, Alabama	898	80.4	2.0	(76.5–84.3)
Bismarck, North Dakota	710	88.1	1.6	(84.9–91.2)
Boise City, Idaho	1,003	76.6	1.9	(73.0–80.3)
Boston, Massachusetts^§^	2,790	91.8	0.9	(90.0–93.5)
Buffalo-Cheektowaga-Niagara Falls, New York	316	89.7	2.1	(85.5–93.9)
Burlington-South Burlington, Vermont	1,209	90.8	1.2	(88.5–93.2)
Cambridge-Newton-Framingham, Massachusetts^§^	3,453	93.5	0.7	(92.1–95.0)
Camden, New Jersey^§^	1,345	83.5	1.6	(80.3–86.7)
Cedar Rapids, Iowa	419	85.1	2.3	(80.5–89.6)
Charleston, West Virginia	566	72.8	2.6	(67.8–77.9)
Charleston-North Charleston, South Carolina	1,017	76.6	1.8	(73.0–80.2)
Charlotte-Concord-Gastonia, North Carolina-South Carolina	1,334	76.5	1.5	(73.5–79.5)
Chattanooga, Tennessee-Georgia	354	77.2	3.8	(69.8–84.6)
Chicago-Naperville-Elgin, Illinois-Indiana-Wisconsin	2,360	78.1	1.4	(75.4–80.8)
Cincinnati, Ohio-Kentucky-Indiana	1,853	81.0	1.5	(78.1–84.0)
Claremont-Lebanon, New Hampshire-Vermont	1,079	82.8	1.8	(79.2–86.4)
Cleveland-Elyria, Ohio	756	85.6	1.9	(82.0–89.3)
Colorado Springs, Colorado	973	82.5	1.6	(79.4–85.7)
Columbia, South Carolina	990	77.8	1.9	(74.1–81.4)
Columbus, Ohio	1,344	83.3	1.4	(80.7–86.0)
Crestview-Fort Walton Beach-Destin, Florida	685	74.2	2.5	(69.3–79.0)
Dallas-Plano-Irving, Texas^§^	665	71.0	2.2	(66.6–75.3)
Davenport-Moline-Rock Island, Iowa-Illinois	413	86.9	2.5	(82.0–91.9)
Dayton, Ohio	526	82.4	2.7	(77.1–87.8)
Deltona-Daytona Beach-Ormond Beach, Florida	534	74.0	2.9	(68.3–79.8)
Denver-Aurora-Lakewood, Colorado	4,164	80.4	0.8	(78.8–81.9)
Des Moines-West Des Moines, Iowa	882	89.5	1.6	(86.4–92.6)
Duluth, Minnesota-Wisconsin	481	88.0	2.9	(82.3–93.8)
Durham-Chapel Hill, North Carolina	419	74.2	2.8	(68.7–79.8)
El Paso, Texas	501	60.5	2.8	(55.1–66.0)
Evansville, Indiana-Kentucky	360	84.6	2.6	(79.6–89.6)
Fargo, North Dakota-Minnesota	867	89.0	1.4	(86.3–91.8)
Fayetteville-Springdale-Rogers, Arkansas-Missouri	502	69.6	3.0	(63.8–75.4)
Fort Smith, Arkansas-Oklahoma	311	73.3	3.5	(66.4–80.2)
Fort Wayne, Indiana	503	76.6	2.6	(71.6–81.7)
Fort Worth-Arlington, Texas^§^	505	69.9	3.0	(64.1–75.7)
Gainesville, Florida	626	77.5	2.7	(72.3–82.7)
Grand Forks, North Dakota-Minnesota	345	88.3	2.7	(83.1–93.6)
Grand Island, Nebraska	467	76.9	3.2	(70.7–83.1)
Grand Rapids-Wyoming, Michigan	938	81.5	2.0	(77.5–85.4)
Greensboro-High Point, North Carolina	448	75.1	2.8	(69.6–80.6)
Greenville-Anderson-Mauldin, South Carolina	865	76.3	2.0	(72.4–80.2)
Gulfport-Biloxi-Pascagoula, Mississippi	524	70.0	2.7	(64.8–75.2)
Hagerstown-Martinsburg, Maryland-West Virginia	492	80.4	2.8	(75.0–85.8)
Hartford-West Hartford-East Hartford, Connecticut	1,917	88.3	1.1	(86.0–90.5)
Hilton Head Island-Bluffton-Beaufort, South Carolina	406	71.8	3.7	(64.6–79.0)
Houston-The Woodlands-Sugar Land, Texas	1,062	66.4	2.0	(62.4–70.3)
Huntington-Ashland, West Virginia-Kentucky-Ohio	798	75.8	2.0	(71.8–79.7)
Idaho Falls, Idaho	343	76.4	3.1	(70.3–82.4)
Indianapolis-Carmel-Anderson, Indiana	1,738	77.5	1.4	(74.8–80.2)
Jackson, Mississippi	542	71.2	2.7	(65.9–76.5)
Jacksonville, Florida	1,863	78.1	1.5	(75.2–81.0)
Kansas City, Missouri-Kansas	4,987	80.5	1.1	(78.2–82.7)
Kingsport-Bristol-Bristol, Tennessee-Virginia	322	81.9	3.3	(75.5–88.4)
Knoxville, Tennessee	442	76.7	2.8	(71.3–82.1)
Lansing-East Lansing, Michigan	465	85.1	2.1	(81.1–89.2)
Lexington-Fayette, Kentucky	478	78.9	2.4	(74.1–83.7)
Lincoln, Nebraska	1,402	82.2	1.3	(79.7–84.8)
Little Rock-North Little Rock-Conway, Arkansas	716	77.7	2.2	(73.4–82.1)
Logan, Utah-Idaho	461	76.6	2.5	(71.7–81.6)
Los Angeles-Long Beach-Anaheim, California	2,283	75.8	1.2	(73.4–78.1)
Louisville-Jefferson County, Kentucky-Indiana	1,416	81.0	1.7	(77.8–84.3)
Lubbock, Texas	315	72.2	3.5	(65.2–79.1)
Manhattan, Kansas	487	85.5	1.9	(81.7–89.3)
Memphis, Tennessee-Mississippi-Arkansas	821	79.5	2.1	(75.4–83.6)
Miami-Fort Lauderdale-West Palm Beach, Florida	1,290	66.8	2.0	(62.9–70.6)
Milwaukee-Waukesha-West Allis, Wisconsin	889	82.4	2.1	(78.4–86.5)
Minneapolis-St. Paul-Bloomington, Minnesota-Wisconsin	6,521	87.4	0.8	(85.9–89.0)
Minot, North Dakota	468	90.1	1.8	(86.5–93.7)
Montgomery County-Bucks County-Chester County, Pennsylvania^§^	683	87.5	1.6	(84.4–90.6)
Myrtle Beach-Conway-North Myrtle Beach, South Carolina-North Carolina	478	68.3	3.0	(62.5–74.1)
Nashville-Davidson County-Murfreesboro-Franklin, Tennessee	758	82.0	1.9	(78.3–85.7)
Nassau County-Suffolk County, New York^§^	675	81.5	2.0	(77.6–85.4)
Newark, New Jersey-Pennsylvania^§^	3,023	80.6	1.1	(78.4–82.7)
New Orleans-Metairie, Louisiana	790	73.5	2.8	(68.0–78.9)
New York-Jersey City-White Plains, New York-New Jersey^§^	6,451	79.6	0.8	(78.1–81.1)
Norfolk, Nebraska	480	83.0	2.2	(78.8–87.3)
North Platte, Nebraska	443	79.6	3.1	(73.6–85.6)
North Port-Sarasota-Bradenton, Florida	554	66.1	2.8	(60.7–71.5)
Oakland-Hayward-Berkeley, California^§^	528	87.1	1.8	(83.7–90.6)
Ogden-Clearfield, Utah	1,826	85.3	1.1	(83.2–87.5)
Oklahoma City, Oklahoma	1,759	75.6	1.4	(72.9–78.3)
Omaha-Council Bluffs, Nebraska-Iowa	2,255	83.3	1.1	(81.0–85.5)
Orlando-Kissimmee-Sanford, Florida	1,411	69.1	1.8	(65.6–72.6)
Panama City, Florida	599	69.8	2.8	(64.3–75.3)
Pensacola-Ferry Pass-Brent, Florida	885	73.4	2.0	(69.5–77.3)
Philadelphia, Pennsylvania^§^	1,198	81.4	1.6	(78.3–84.4)
Phoenix-Mesa-Scottsdale, Arizona	1,026	73.7	2.2	(69.3–78.0)
Pittsburgh, Pennsylvania	1,486	86.7	1.2	(84.2–89.1)
Ponce, Puerto Rico	365	94.0	1.5	(91.0–97.0)
Portland-South Portland, Maine	1,716	86.5	1.2	(84.1–88.9)
Portland-Vancouver-Hillsboro, Oregon-Washington	2,180	79.1	1.3	(76.6–81.7)
Port St. Lucie, Florida	493	67.5	3.4	(60.9–74.1)
Providence-Warwick, Rhode Island-Massachusetts	5,539	84.5	0.8	(82.9–86.2)
Provo-Orem, Utah	1,485	79.8	1.3	(77.2–82.4)
Raleigh, North Carolina	551	81.6	1.8	(78.0–85.1)
Rapid City, South Dakota	579	87.6	1.8	(84.2–91.1)
Reno, Nevada	1,249	76.7	1.6	(73.5–79.8)
Richmond, Virginia	949	83.1	1.7	(79.9–86.4)
Riverside-San Bernardino-Ontario, California	1,027	78.9	1.7	(75.6–82.1)
Rochester, New York	349	87.9	2.3	(83.3–92.5)
Rockingham County-Strafford County, New Hampshire^§^	1,147	85.5	1.5	(82.5–88.4)
Sacramento-Roseville-Arden-Arcade, California	625	81.1	2.1	(77.1–85.2)
St. Louis, Missouri-Illinois	1,314	83.7	1.4	(80.9–86.5)
Salem, Oregon	327	73.5	3.9	(65.8–81.2)
Salina, Kansas	353	74.2	3.1	(68.1–80.2)
Salisbury, Maryland-Delaware	1,154	82.1	2.0	(78.2–86.1)
Salt Lake City, Utah	3,508	80.9	0.9	(79.2–82.7)
San Antonio-New Braunfels, Texas	623	65.7	2.5	(60.9–70.6)
San Francisco-Redwood City-South San Francisco, California^§^	405	92.6	1.5	(89.8–95.5)
San Jose-Sunnyvale-Santa Clara, California	487	81.3	2.6	(76.3–86.3)
San Juan-Carolina-Caguas, Puerto Rico	2,442	89.7	0.8	(88.2–91.3)
Scottsbluff, Nebraska	424	76.5	2.9	(70.9–82.2)
Scranton-Wilkes-Barre-Hazleton, Pennsylvania	347	85.8	2.7	(80.5–91.0)
Seattle-Bellevue-Everett, Washington^§^	2,665	83.0	1.0	(81.0–85.1)
Shreveport-Bossier City, Louisiana	353	68.9	4.0	(61.0–76.8)
Silver Spring-Frederick-Rockville, Maryland^§^	1,683	84.6	1.5	(81.7–87.5)
Sioux City, Iowa-Nebraska-South Dakota	653	81.5	3.2	(75.2–87.9)
Sioux Falls, South Dakota	752	84.1	2.0	(80.2–88.0)
Spartanburg, South Carolina	370	76.3	3.2	(70.0–82.5)
Spokane-Spokane Valley, Washington	555	77.1	2.6	(72.1–82.2)
Springfield, Massachusetts	1,055	91.6	1.6	(88.4–94.8)
Tallahassee, Florida	1,160	79.1	2.4	(74.3–83.9)
Tampa-St. Petersburg-Clearwater, Florida	1,313	75.4	1.7	(72.1–78.7)
Toledo, Ohio	681	77.8	3.1	(71.7–83.8)
Topeka, Kansas	1,610	81.7	1.3	(79.1–84.3)
Tulsa, Oklahoma	1,267	79.1	1.5	(76.1–82.1)
Virginia Beach-Norfolk-Newport News, Virginia-North Carolina	1,170	85.0	1.5	(82.0–87.9)
Warren-Troy-Farmington Hills, Michigan^§^	1,520	83.0	1.5	(80.1–85.9)
Washington-Arlington-Alexandria, District of Columbia-Virginia-Maryland-West Virginia^§^	6,219	82.5	1.0	(80.5–84.5)
Wichita, Kansas	3,314	76.2	1.0	(74.2–78.2)
Wilmington, Delaware-Maryland-New Jersey^§^	2,267	85.7	1.1	(83.6–87.9)
Winston-Salem, North Carolina	416	70.3	3.3	(63.9–76.7)
Worcester, Massachusetts-Connecticut	1,923	91.4	1.1	(89.2–93.7)
*Median*		*80.6*		
*Range*		*60.5–97.6*		

**Abbreviations**: CI = confidence interval; MMSA = metropolitan and micropolitan statistical areas; SE = standard error.

* Age adjusted to the 2000 U.S. standard population.

^†^ Including health insurance, prepaid plans (e.g., health maintenance organizations), or government plans (e.g., Medicare).

^§^ Metropolitan division.

**TABLE 11:** Age-adjusted* prevalence estimates of adults aged 18–64 years who have health care coverage,† by state/territory — Behavioral Risk Factor Surveillance System, United States, 2014

State/Territory	Sample size	%	SE	95% CI
Alabama	5,440	81.9	0.8	(80.3–83.4)
Alaska	3,312	82.5	1.0	(80.6–84.5)
Arizona	8,036	81.8	0.7	(80.3–83.2)
Arkansas	2,778	79.2	1.2	(76.8–81.6)
California	6,447	82.0	0.6	(80.7–83.2)
Colorado	8,874	84.3	0.5	(83.2–85.3)
Connecticut	5,132	88.7	0.7	(87.3–90.1)
Delaware	2,665	88.6	0.9	(86.8–90.4)
District of Columbia	2,421	90.6	1.1	(88.4–92.8)
Florida	5,408	76.6	0.8	(74.9–78.2)
Georgia	4,064	74.4	1.0	(72.5–76.4)
Hawaii	5,003	89.9	0.7	(88.6–91.2)
Idaho	3,490	79.4	1.0	(77.4–81.4)
Illinois	3,384	85.1	0.9	(83.3–86.9)
Indiana	7,183	81.3	0.7	(80.0–82.6)
Iowa	5,091	90.0	0.6	(88.8–91.2)
Kansas	9,155	81.8	0.6	(80.7–82.9)
Kentucky	7,110	87.3	0.8	(85.8–88.8)
Louisiana	4,616	76.8	0.8	(75.1–78.4)
Maine	5,696	85.5	0.7	(84.1–86.9)
Maryland	7,663	88.2	0.8	(86.5–89.8)
Massachusetts	9,615	94.5	0.4	(93.7–95.3)
Michigan	5,447	86.8	0.6	(85.6–88.1)
Minnesota	11,656	90.8	0.4	(90.0–91.5)
Mississippi	2,598	76.6	1.2	(74.2–79.0)
Missouri	4,219	83.1	0.9	(81.3–84.9)
Montana	4,590	83.5	0.8	(81.9–85.2)
Nebraska	14,323	84.1	0.6	(83.0–85.2)
Nevada	2,408	78.7	1.4	(76.0–81.4)
New Hampshire	3,868	85.0	0.9	(83.1–86.8)
New Jersey	8,831	84.2	0.6	(83.0–85.5)
New Mexico	5,853	80.4	0.9	(78.7–82.1)
New York	4,651	84.8	0.7	(83.4–86.2)
North Carolina	4,831	79.3	0.7	(77.9–80.7)
North Dakota	4,897	89.3	0.8	(87.8–90.9)
Ohio	6,818	87.1	0.7	(85.6–88.5)
Oklahoma	5,356	82.5	0.7	(81.1–83.9)
Oregon	3,138	85.9	0.9	(84.1–87.7)
Pennsylvania	6,807	86.7	0.7	(85.4–88.1)
Rhode Island	4,154	89.7	0.8	(88.1–91.4)
South Carolina	6,896	78.1	0.7	(76.7–79.5)
South Dakota	4,782	87.6	1.0	(85.6–89.5)
Tennessee	3,064	81.7	1.1	(79.5–83.8)
Texas	9,879	70.5	0.7	(69.1–72.0)
Utah	11,236	84.0	0.4	(83.2–84.9)
Vermont	4,574	91.2	0.6	(90.0–92.3)
Virginia	6,444	83.7	0.7	(82.3–85.1)
Washington	6,294	86.6	0.7	(85.3–87.9)
West Virginia	4,109	86.3	0.7	(84.8–87.7)
Wisconsin	4,703	89.0	0.8	(87.5–90.5)
Wyoming	3,495	81.0	1.2	(78.6–83.3)
Guam	2,185	73.3	1.5	(70.4–76.1)
Puerto Rico	3,923	92.6	0.5	(91.5–93.6)
*Median*		*84.2*		
*Range*		*70.5–94.5*		

**Abbreviations:** CI = confidence interval; SE = standard error.

* Age adjusted to the 2000 U.S. standard population.

^†^ Including health insurance, prepaid plans (e.g., health maintenance organizations), or government plans (e.g., Medicare).

**TABLE 12:** Age-adjusted* prevalence estimates of adults aged 18–64 years who have health care coverage,^†^ by metropolitan and micropolitan statistical area — Behavioral Risk Factor Surveillance System, United States, 2014

MMSA	Sample size	%	SE	95% CI
Aberdeen, South Dakota	379	90.3	2.9	(84.5–96.0)
Aguadilla-Isabela, Puerto Rico	361	89.0	2.1	(84.9–93.1)
Albuquerque, New Mexico	1,213	82.8	1.6	(79.7–86.0)
Allentown-Bethlehem-Easton, Pennsylvania-New Jersey	651	84.6	2.4	(79.8–89.4)
Anchorage, Alaska	1,362	83.0	1.4	(80.3–85.7)
Atlanta-Sandy Springs-Roswell, Georgia	1,878	77.4	1.3	(74.8–80.0)
Augusta-Richmond County, Georgia-South Carolina	578	76.5	3.5	(69.6–83.3)
Austin-Round Rock, Texas	1,507	79.1	1.5	(76.2–81.9)
Baltimore-Columbia-Towson, Maryland	2,900	90.8	1.1	(88.6–92.9)
Baton Rouge, Louisiana	656	78.7	2.1	(74.5–82.9)
Berlin, New Hampshire-Vermont	325	79.0	5.2	(68.9–89.1)
Billings, Montana	521	82.9	2.2	(78.6–87.3)
Birmingham-Hoover, Alabama	1,053	83.0	1.7	(79.7–86.3)
Bismarck, North Dakota	624	88.8	2.1	(84.7–93.0)
Boise City, Idaho	845	80.1	1.9	(76.5–83.8)
Boston, Massachusetts^§^	2,810	94.1	0.8	(92.6–95.6)
Burlington-South Burlington, Vermont	1,493	93.2	0.8	(91.6–94.9)
Cambridge-Newton-Framingham, Massachusetts^§^	3,183	94.5	0.7	(93.1–95.9)
Camden, New Jersey^§^	1,126	92.8	1.3	(90.2–95.4)
Cedar Rapids, Iowa	401	89.3	2.4	(84.7–94.0)
Charleston, West Virginia	588	88.8	1.8	(85.3–92.4)
Charleston-North Charleston, South Carolina	918	78.2	2.0	(74.3–82.1)
Charlotte-Concord-Gastonia, North Carolina-South Carolina	1,421	78.7	1.4	(75.9–81.5)
Chicago-Naperville-Elgin, Illinois-Indiana-Wisconsin	2,859	83.3	1.1	(81.1–85.4)
Cincinnati, Ohio-Kentucky-Indiana	1,347	85.2	1.9	(81.5–88.9)
Claremont-Lebanon, New Hampshire-Vermont	1,077	86.8	2.0	(82.9–90.7)
Cleveland-Elyria, Ohio	618	88.5	2.0	(84.6–92.3)
College Station-Bryan, Texas	308	82.0	3.8	(74.5–89.5)
Colorado Springs, Colorado	885	85.4	1.6	(82.2–88.5)
Columbia, South Carolina	851	81.4	1.9	(77.8–85.1)
Columbus, Ohio	1,160	90.6	1.4	(87.9–93.3)
Corpus Christi, Texas	307	83.0	2.9	(77.3–88.6)
Dallas-Plano-Irving, Texas^§^	825	74.2	2.2	(69.9–78.5)
Dayton, Ohio	340	88.3	2.2	(83.9–92.7)
Denver-Aurora-Lakewood, Colorado	4,051	84.1	0.7	(82.6–85.5)
Des Moines-West Des Moines, Iowa	861	90.6	1.5	(87.7–93.5)
Duluth, Minnesota-Wisconsin	634	89.0	1.8	(85.6–92.5)
El Paso, Texas	439	64.7	2.9	(59.1–70.4)
Evansville, Indiana-Kentucky	376	89.4	2.6	(84.3–94.6)
Fargo, North Dakota-Minnesota	739	90.9	1.6	(87.7–94.0)
Fayetteville-Springdale-Rogers, Arkansas-Missouri	442	81.0	3.2	(74.8–87.2)
Fort Wayne, Indiana	510	80.3	2.7	(75.0–85.6)
Fort Worth-Arlington, Texas^§^	480	71.9	3.0	(66.0–77.7)
Grand Island, Nebraska	639	81.4	2.6	(76.4–86.5)
Grand Rapids-Wyoming, Michigan	598	86.9	2.1	(82.8–91.0)
Greensboro-High Point, North Carolina	344	79.0	2.8	(73.5–84.6)
Greenville-Anderson-Mauldin, South Carolina	951	80.4	1.7	(77.0–83.8)
Hagerstown-Martinsburg, Maryland-West Virginia	443	88.0	3.0	(82.2–93.8)
Hartford-West Hartford-East Hartford, Connecticut	1,692	91.0	1.1	(88.9–93.1)
Hilton Head Island-Bluffton-Beaufort, South Carolina	264	78.5	3.5	(71.6–85.4)
Houston-The Woodlands-Sugar Land, Texas	1,428	69.4	1.9	(65.7–73.0)
Huntington-Ashland, West Virginia-Kentucky-Ohio	833	85.9	2.1	(81.8–90.0)
Idaho Falls, Idaho	355	84.4	2.4	(79.7–89.2)
Indianapolis-Carmel-Anderson, Indiana	2,304	83.6	1.1	(81.4–85.9)
Jacksonville, Florida	404	80.9	3.0	(75.1–86.7)
Kansas City, Missouri-Kansas	3,192	83.0	1.3	(80.5–85.4)
Kingsport-Bristol-Bristol, Tennessee-Virginia	276	80.8	4.2	(72.6–89.1)
Knoxville, Tennessee	316	83.8	3.3	(77.4–90.2)
Lafayette, Louisiana	399	79.8	2.5	(74.9–84.7)
Lexington-Fayette, Kentucky	452	83.5	2.6	(78.4–88.5)
Lincoln, Nebraska	1,423	85.1	1.4	(82.4–87.9)
Little Rock-North Little Rock-Conway, Arkansas	685	80.0	2.4	(75.2–84.7)
Logan, Utah-Idaho	463	87.0	1.8	(83.4–90.5)
Los Angeles-Long Beach-Anaheim, California	1,871	78.2	1.2	(75.8–80.6)
Louisville-Jefferson County, Kentucky-Indiana	1,492	87.6	1.6	(84.5–90.6)
Madison, Wisconsin	400	90.2	2.2	(85.9–94.5)
Memphis, Tennessee-Mississippi-Arkansas	540	79.1	3.0	(73.3–84.9)
Miami-Fort Lauderdale-West Palm Beach, Florida	1,291	74.8	1.7	(71.5–78.1)
Milwaukee-Waukesha-West Allis, Wisconsin	888	89.3	1.7	(85.9–92.6)
Minneapolis-St. Paul-Bloomington, Minnesota-Wisconsin	6,385	91.3	0.5	(90.4–92.3)
Minot, North Dakota	380	88.9	2.1	(84.7–93.1)
Montgomery, Alabama	331	90.3	2.1	(86.2–94.3)
Montgomery County-Bucks County-Chester County, Pennsylvania^§^	531	91.5	1.9	(87.7–95.2)
Myrtle Beach-Conway-North Myrtle Beach, South Carolina-North Carolina	591	74.3	2.4	(69.6–79.0)
Nashville-Davidson County-Murfreesboro-Franklin, Tennessee	547	87.0	2.0	(83.0–91.0)
Nassau County-Suffolk County, New York^§^	519	86.7	2.2	(82.3–91.0)
Newark, New Jersey-Pennsylvania^§^	2,845	82.2	1.1	(80.0–84.5)
New Orleans-Metairie, Louisiana	1,287	80.0	1.5	(77.1–82.9)
New York-Jersey City-White Plains, New York-New Jersey^§^	5,373	81.7	0.8	(80.1–83.3)
Norfolk, Nebraska	687	85.6	1.8	(82.0–89.2)
North Platte, Nebraska	612	81.4	2.2	(77.2–85.7)
North Port-Sarasota-Bradenton, Florida	216	76.0	4.6	(67.0–84.9)
Oakland-Hayward-Berkeley, California^§^	523	86.8	1.9	(83.2–90.5)
Ogden-Clearfield, Utah	2,211	85.4	0.9	(83.6–87.2)
Oklahoma City, Oklahoma	1,602	81.4	1.3	(79.0–83.9)
Omaha-Council Bluffs, Nebraska-Iowa	3,305	85.7	0.9	(83.8–87.5)
Orlando-Kissimmee-Sanford, Florida	597	79.7	2.3	(75.1–84.2)
Philadelphia, Pennsylvania^§^	945	81.9	1.8	(78.3–85.5)
Phoenix-Mesa-Scottsdale, Arizona	5,289	81.7	0.9	(79.9–83.4)
Pittsburgh, Pennsylvania	1,498	89.9	1.2	(87.4–92.3)
Ponce, Puerto Rico	357	96.6	1.2	(94.2–99.0)
Portland-South Portland, Maine	1,672	88.7	1.3	(86.3–91.2)
Portland-Vancouver-Hillsboro, Oregon-Washington	1,829	89.6	1.0	(87.5–91.6)
Providence-Warwick, Rhode Island-Massachusetts	5,164	92.0	0.7	(90.6–93.4)
Provo-Orem, Utah	1,760	84.4	1.0	(82.4–86.3)
Raleigh, North Carolina	553	83.7	1.8	(80.2–87.2)
Rapid City, South Dakota	868	85.9	1.8	(82.4–89.4)
Reno, Nevada	772	81.9	2.0	(78.0–85.8)
Richmond, Virginia	1,025	86.1	1.6	(83.0–89.1)
Riverside-San Bernardino-Ontario, California	684	82.1	1.8	(78.5–85.6)
Roanoke, Virginia	322	81.3	3.3	(74.8–87.7)
Rochester, Minnesota	489	91.1	2.1	(87.1–95.2)
Rockingham County-Strafford County, New Hampshire^§^	924	87.0	1.7	(83.6–90.3)
Sacramento-Roseville-Arden-Arcade, California	459	90.2	1.9	(86.4–94.0)
St. Cloud, Minnesota	439	93.8	1.4	(91.0–96.5)
St. Louis, Missouri-Illinois	1,235	86.8	1.7	(83.6–90.1)
Salisbury, Maryland-Delaware	1,060	84.1	2.1	(80.0–88.1)
Salt Lake City, Utah	4,207	83.8	0.7	(82.4–85.2)
San Antonio-New Braunfels, Texas	1,416	72.9	1.6	(69.8–75.9)
San Juan-Carolina-Caguas, Puerto Rico	2,430	91.2	0.8	(89.7–92.7)
Scottsbluff, Nebraska	505	81.6	2.3	(77.1–86.2)
Seattle-Bellevue-Everett, Washington^§^	2,418	86.7	1.1	(84.6–88.8)
Shreveport-Bossier City, Louisiana	377	73.9	3.0	(68.0–79.7)
Silver Spring-Frederick-Rockville, Maryland^§^	1,516	86.0	2.0	(82.0–89.9)
Sioux City, Iowa-Nebraska-South Dakota	673	83.6	2.9	(77.9–89.3)
Sioux Falls, South Dakota	877	87.7	2.2	(83.5–92.0)
Spartanburg, South Carolina	321	77.2	3.4	(70.6–83.8)
Spokane-Spokane Valley, Washington	442	85.0	2.6	(79.9–90.1)
Springfield, Massachusetts	688	91.8	1.5	(88.8–94.8)
Tampa-St. Petersburg-Clearwater, Florida	899	81.3	1.9	(77.5–85.0)
Toledo, Ohio	427	88.7	2.6	(83.5–93.8)
Topeka, Kansas	942	80.8	1.7	(77.4–84.2)
Tulsa, Oklahoma	1,323	84.0	1.3	(81.4–86.6)
Tuscaloosa, Alabama	493	84.0	2.2	(79.6–88.4)
Virginia Beach-Norfolk-Newport News, Virginia-North Carolina	1,287	86.0	1.4	(83.2–88.7)
Warren-Troy-Farmington Hills, Michigan^§^	1,362	88.4	1.2	(86.0–90.8)
Washington-Arlington-Alexandria, District of Columbia-Virginia-Maryland-West Virginia^§^	5,411	85.9	1.0	(84.0–87.9)
Wichita, Kansas	1,843	81.5	1.2	(79.1–83.8)
Wichita Falls, Texas	256	80.5	6.4	(68.0–93.1)
Wilmington, Delaware-Maryland-New Jersey^§^	1,817	90.9	1.0	(88.9–93.0)
Worcester, Massachusetts-Connecticut	1,580	95.5	0.8	(94.0–97.1)
Youngstown-Warren-Boardman, Ohio-Pennsylvania	314	82.9	3.9	(75.4–90.5)
*Median*		*84.3*		
*Range*		*64.7–96.6*		

**Abbreviations:** CI = confidence interval; MMSA = metropolitan and micropolitan statistical areas; SE = standard error.

* Age adjusted to the 2000 U.S. standard population.

^†^ Including health insurance, prepaid plans (e.g., health maintenance organizations), or government plans (e.g., Medicare).

^§^ Metropolitan division.

#### Recent Routine Physical Checkup

A recent routine physical checkup was defined as a visit that occurred during the past 12 months to a doctor for a general physical examination rather than for a specific injury, illness, or condition. In 2013, age-adjusted prevalence estimates of a routine physical checkup during the past 12 months ranged from 57.0% in Oregon to 77.7% in Rhode Island (median: 67.9%) (Table 13). Among selected MMSA, age-adjusted prevalence estimates ranged from 52.6% in Logan, Utah-Idaho, to 79.5% in Memphis, Tennessee-Mississippi-Arkansas (median: 68.9%) (Table 14). In 2014, age-adjusted prevalence estimates ranged from 57.2% in Idaho to 79.2% in Rhode Island (median: 69.2%) (Table 15). Among selected MMSA, age-adjusted prevalence estimates ranged from 55.9% in Scottsbluff, Nebraska, to 80.8% in Providence-Warwick, Rhode Island-Massachusetts (median: 69.8%) (Table 16).

**TABLE 13:** Age-adjusted* prevalence estimates of adults aged ≥18 years who visited a doctor for a routine physical checkup during the past 12 months, by state/territory — Behavioral Risk Factor Surveillance System, United States, 2013

State/Territory	Sample size	%	SE	95% CI
Alabama	6,388	71.6	0.9	(69.7–73.4)
Alaska	4,536	57.8	1.1	(55.7–60.0)
Arizona	4,192	63.8	1.4	(61.0–66.5)
Arkansas	5,165	65.0	1.1	(62.9–67.1)
California	11,464	63.8	0.6	(62.6–65.1)
Colorado	13,435	61.5	0.6	(60.4–62.7)
Connecticut	7,665	70.9	0.9	(69.2–72.6)
Delaware	5,165	75.3	0.9	(73.5–77.1)
District of Columbia	4,890	74.0	1.0	(72.0–76.1)
Florida	33,809	67.9	0.6	(66.6–69.1)
Georgia	8,050	72.6	0.7	(71.1–74.0)
Hawaii	7,828	66.9	0.8	(65.3–68.5)
Idaho	5,583	57.9	1.0	(55.9–59.8)
Illinois	5,597	66.2	1.0	(64.3–68.1)
Indiana	10,220	64.0	0.7	(62.7–65.3)
Iowa	8,073	68.6	0.8	(67.1–70.1)
Kansas	22,855	69.6	0.4	(68.8–70.4)
Kentucky	10,800	68.6	0.7	(67.1–70.0)
Louisiana	5,159	74.8	1.1	(72.6–76.9)
Maine	8,044	69.8	0.8	(68.2–71.4)
Maryland	12,887	74.6	0.7	(73.3–76.0)
Massachusetts	14,963	76.9	0.6	(75.7–78.1)
Michigan	12,602	69.2	0.6	(68.0–70.4)
Minnesota	14,189	67.9	0.7	(66.4–69.3)
Mississippi	7,299	70.6	0.9	(68.9–72.3)
Missouri	7,007	66.6	0.9	(64.8–68.4)
Montana	9,559	60.7	0.8	(59.2–62.2)
Nebraska	16,919	61.4	0.7	(60.1–62.7)
Nevada	5,038	66.0	1.3	(63.5–68.5)
New Hampshire	6,413	70.2	0.9	(68.5–72.0)
New Jersey	13,195	74.7	0.6	(73.5–75.9)
New Mexico	9,177	62.5	0.8	(61.0–64.1)
New York	8,906	74.4	0.7	(73.1–75.7)
North Carolina	8,759	73.0	0.7	(71.6–74.4)
North Dakota	7,720	62.7	0.8	(61.0–64.3)
Ohio	11,819	69.8	0.7	(68.5–71.2)
Oklahoma	8,090	61.5	0.8	(60.0–63.0)
Oregon	5,783	57.0	0.9	(55.2–58.9)
Pennsylvania	11,316	69.9	0.6	(68.7–71.2)
Rhode Island	6,489	77.7	0.8	(76.1–79.3)
South Carolina	10,568	65.6	0.7	(64.2–67.0)
South Dakota	6,812	67.3	1.0	(65.3–69.3)
Tennessee	5,718	74.9	0.9	(73.1–76.7)
Texas	10,673	69.9	0.7	(68.5–71.3)
Utah	12,451	59.6	0.6	(58.5–60.7)
Vermont	6,331	66.1	0.9	(64.3–67.8)
Virginia	8,359	72.5	0.7	(71.1–73.9)
Washington	10,986	61.7	0.7	(60.3–63.0)
West Virginia	5,833	72.5	0.8	(70.9–74.1)
Wisconsin	6,552	67.1	1.0	(65.2–69.0)
Wyoming	6,350	58.6	1.0	(56.7–60.5)
Guam	1,881	65.1	1.5	(62.2–68.0)
Puerto Rico	5,928	76.0	0.8	(74.4–77.5)
*Median*		*67.9*		
*Range*		*57.0–77.7*		

**Abbreviations:** CI = confidence interval; SE = standard error.

* Age adjusted to the 2000 U.S. standard population.

**TABLE 14:** Age-adjusted* prevalence estimates of adults aged ≥18 years who visited a doctor for a routine physical checkup during the past 12 months, by metropolitan and micropolitan statistical area — Behavioral Risk Factor Surveillance System, United States, 2013

MMSA	Sample size	%	SE	95% CI
Aguadilla-Isabela, Puerto Rico	587	78.5	2.5	(73.6–83.3)
Akron, Ohio	683	78.2	2.5	(73.3–83.2)
Albuquerque, New Mexico	2,050	61.2	1.5	(58.3–64.0)
Allentown-Bethlehem-Easton, Pennsylvania-New Jersey	1,020	70.5	2.3	(65.9–75.1)
Anchorage, Alaska	1,517	58.7	1.7	(55.4–62.0)
Atlanta-Sandy Springs-Roswell, Georgia	3,484	72.0	1.1	(69.9–74.1)
Augusta-Richmond County, Georgia-South Carolina	899	78.6	2.1	(74.5–82.8)
Austin-Round Rock, Texas	922	68.3	2.1	(64.2–72.3)
Baltimore-Columbia-Towson, Maryland	4,730	74.5	1.1	(72.4–76.6)
Baton Rouge, Louisiana	916	72.7	2.4	(68.0–77.5)
Billings, Montana	805	64.0	2.0	(60.1–67.9)
Birmingham-Hoover, Alabama	1,338	72.7	1.8	(69.1–76.3)
Bismarck, North Dakota	1,023	61.2	2.1	(57.1–65.2)
Boise City, Idaho	1,480	60.8	1.8	(57.3–64.3)
Boston, Massachusetts^†^	4,031	78.4	1.0	(76.3–80.4)
Buffalo-Cheektowaga-Niagara Falls, New York	502	73.3	2.8	(67.8–78.8)
Burlington-South Burlington, Vermont	1,620	66.7	1.6	(63.7–69.8)
Cambridge-Newton-Framingham, Massachusetts^†^	4,876	75.4	1.0	(73.4–77.5)
Camden, New Jersey^†^	1,838	74.6	1.5	(71.6–77.6)
Cedar Rapids, Iowa	641	68.2	2.6	(63.1–73.3)
Charleston, West Virginia	813	72.8	2.2	(68.4–77.1)
Charleston-North Charleston, South Carolina	1,527	67.4	1.7	(64.0–70.7)
Charlotte-Concord-Gastonia, North Carolina-South Carolina	1,927	70.1	1.5	(67.2–73.1)
Chattanooga, Tennessee-Georgia	573	79.1	3.1	(73.1–85.2)
Chicago-Naperville-Elgin, Illinois-Indiana-Wisconsin	3,328	66.2	1.2	(63.8–68.6)
Cincinnati, Ohio-Kentucky-Indiana	2,570	69.5	1.5	(66.6–72.3)
Claremont-Lebanon, New Hampshire-Vermont	1,671	69.1	1.9	(65.4–72.7)
Cleveland-Elyria, Ohio	1,102	73.3	2.0	(69.4–77.1)
Colorado Springs, Colorado	1,345	65.7	1.7	(62.4–69.0)
Columbia, South Carolina	1,432	65.8	1.8	(62.2–69.3)
Columbus, Ohio	1,831	71.2	1.4	(68.5–73.9)
Crestview-Fort Walton Beach-Destin, Florida	1,069	64.4	2.2	(60.0–68.8)
Dallas-Plano-Irving, Texas^†^	885	73.1	2.0	(69.3–77.0)
Davenport-Moline-Rock Island, Iowa-Illinois	666	66.8	2.9	(61.1–72.5)
Dayton, Ohio	833	67.0	2.6	(61.9–72.1)
Deltona-Daytona Beach-Ormond Beach, Florida	1,101	66.1	2.8	(60.6–71.6)
Denver-Aurora-Lakewood, Colorado	5,633	62.3	0.8	(60.7–63.9)
Des Moines-West Des Moines, Iowa	1,333	71.0	1.8	(67.5–74.5)
Duluth, Minnesota-Wisconsin	700	71.5	3.1	(65.4–77.6)
Durham-Chapel Hill, North Carolina	616	73.2	2.6	(68.1–78.2)
El Paso, Texas	749	65.8	2.4	(61.1–70.6)
Evansville, Indiana-Kentucky	569	65.6	3.1	(59.6–71.6)
Fargo, North Dakota-Minnesota	1,173	66.7	2.1	(62.6–70.7)
Fayetteville-Springdale-Rogers, Arkansas-Missouri	796	63.8	2.6	(58.7–69.0)
Fort Smith, Arkansas-Oklahoma	489	64.4	3.5	(57.6–71.3)
Fort Wayne, Indiana	776	62.1	2.4	(57.4–66.8)
Fort Worth-Arlington, Texas^†^	798	67.6	2.6	(62.6–72.6)
Gainesville, Florida	1,025	64.6	2.7	(59.4–69.8)
Grand Forks, North Dakota-Minnesota	500	66.9	3.3	(60.4–73.5)
Grand Island, Nebraska	790	56.0	2.7	(50.7–61.3)
Grand Rapids-Wyoming, Michigan	1,333	70.7	1.7	(67.4–74.1)
Greensboro-High Point, North Carolina	658	73.2	2.5	(68.3–78.1)
Greenville-Anderson-Mauldin, South Carolina	1,337	61.5	1.9	(57.8–65.3)
Gulfport-Biloxi-Pascagoula, Mississippi	759	65.6	2.5	(60.8–70.5)
Hagerstown-Martinsburg, Maryland-West Virginia	756	72.0	2.8	(66.6–77.5)
Hartford-West Hartford-East Hartford, Connecticut	2,820	73.2	1.3	(70.6–75.8)
Hilton Head Island-Bluffton-Beaufort, South Carolina	817	59.6	3.1	(53.5–65.7)
Houston-The Woodlands-Sugar Land, Texas	1,364	72.1	1.7	(68.8–75.4)
Huntington-Ashland, West Virginia-Kentucky-Ohio	1,159	72.6	1.8	(69.1–76.1)
Idaho Falls, Idaho	506	59.0	3.0	(53.2–64.8)
Indianapolis-Carmel-Anderson, Indiana	2,518	66.5	1.3	(64.0–69.1)
Jackson, Mississippi	792	78.0	2.1	(73.8–82.2)
Jacksonville, Florida	2,852	68.9	1.4	(66.1–71.6)
Kansas City, Missouri-Kansas	7,341	68.5	1.1	(66.3–70.8)
Kingsport-Bristol-Bristol, Tennessee-Virginia	528	74.0	3.6	(66.8–81.1)
Knoxville, Tennessee	643	70.9	2.5	(65.9–75.8)
Lansing-East Lansing, Michigan	679	69.0	2.4	(64.3–73.6)
Lexington-Fayette, Kentucky	631	67.4	2.3	(62.9–71.9)
Lincoln, Nebraska	1,854	59.9	1.4	(57.2–62.5)
Little Rock-North Little Rock-Conway, Arkansas	1,123	68.2	2.1	(64.2–72.3)
Logan, Utah-Idaho	630	52.6	2.4	(47.9–57.3)
Los Angeles-Long Beach-Anaheim, California	3,025	64.8	1.2	(62.5–67.1)
Louisville-Jefferson County, Kentucky-Indiana	2,128	70.3	1.7	(67.0–73.6)
Lubbock, Texas	516	68.4	3.2	(62.2–74.6)
Manhattan, Kansas	653	70.8	2.1	(66.6–75.0)
Memphis, Tennessee-Mississippi-Arkansas	1,190	79.5	1.9	(75.7–83.3)
Miami-Fort Lauderdale-West Palm Beach, Florida	2,196	68.8	1.7	(65.5–72.1)
Milwaukee-Waukesha-West Allis, Wisconsin	1,262	69.8	2.1	(65.7–73.9)
Minneapolis-St. Paul-Bloomington, Minnesota-Wisconsin	9,040	68.0	0.9	(66.2–69.9)
Minot, North Dakota	642	61.4	2.5	(56.6–66.3)
Montgomery County-Bucks County-Chester County, Pennsylvania^§^	964	70.9	1.9	(67.2–74.5)
Myrtle Beach-Conway-North Myrtle Beach, South Carolina-North Carolina	766	64.5	2.6	(59.3–69.6)
Nashville-Davidson County-Murfreesboro-Franklin, Tennessee	1,049	73.4	1.8	(69.8–76.9)
Nassau County-Suffolk County, New York^†^	940	75.1	1.9	(71.3–78.9)
Newark, New Jersey-Pennsylvania^†^	4,076	75.9	1.0	(74.0–77.9)
New Orleans-Metairie, Louisiana	1,265	73.4	2.3	(68.9–77.8)
New York-Jersey City-White Plains, New York-New Jersey^†^	8,847	74.6	0.7	(73.2–76.1)
Norfolk, Nebraska	666	65.4	2.3	(60.9–69.9)
North Platte, Nebraska	712	56.5	2.8	(51.1–61.9)
North Port-Sarasota-Bradenton, Florida	1,081	64.1	2.3	(59.5–68.7)
Oakland-Hayward-Berkeley, California^†^	699	61.8	2.3	(57.2–66.4)
Ogden-Clearfield, Utah	2,390	63.7	1.2	(61.4–66.1)
Oklahoma City, Oklahoma	2,598	61.0	1.3	(58.4–63.5)
Omaha-Council Bluffs, Nebraska-Iowa	3,104	64.7	1.2	(62.3–67.0)
Orlando-Kissimmee-Sanford, Florida	2,259	65.5	1.6	(62.5–68.6)
Panama City, Florida	1,014	62.6	2.5	(57.8–67.5)
Pensacola-Ferry Pass-Brent, Florida	1,307	69.4	1.8	(66.0–72.9)
Philadelphia, Pennsylvania^†^	1,757	75.6	1.4	(72.8–78.4)
Phoenix-Mesa-Scottsdale, Arizona	1,536	64.1	1.9	(60.4–67.8)
Pittsburgh, Pennsylvania	2,349	69.8	1.4	(67.1–72.5)
Ponce, Puerto Rico	523	72.5	2.7	(67.1–77.8)
Portland-South Portland, Maine	2,627	70.4	1.4	(67.8–73.1)
Portland-Vancouver-Hillsboro, Oregon-Washington	3,172	61.0	1.2	(58.6–63.4)
Port St. Lucie, Florida	1,013	67.6	2.8	(62.1–73.1)
Providence-Warwick, Rhode Island-Massachusetts	8,250	78.8	0.9	(77.0–80.6)
Provo-Orem, Utah	1,803	56.7	1.4	(54.1–59.4)
Raleigh, North Carolina	669	74.4	2.0	(70.6–78.3)
Rapid City, South Dakota	866	65.5	2.3	(61.1–70.0)
Reno, Nevada	1,803	63.5	1.6	(60.3–66.7)
Richmond, Virginia	1,300	72.5	1.7	(69.2–75.8)
Riverside-San Bernardino-Ontario, California	1,363	66.5	1.7	(63.2–69.9)
Rochester, New York	508	70.9	2.7	(65.7–76.1)
Rockingham County-Strafford County, New Hampshire^§^	1,654	70.1	1.7	(66.9–73.4)
Sacramento-Roseville-Arden-Arcade, California	891	63.9	2.2	(59.5–68.2)
St. Louis, Missouri-Illinois	2,039	70.2	1.6	(67.0–73.4)
Salem, Oregon	515	54.2	3.4	(47.6–60.8)
Salina, Kansas	518	69.8	2.7	(64.4–75.1)
Salisbury, Maryland-Delaware	2,047	75.4	1.9	(71.6–79.1)
Salt Lake City, Utah	4,571	60.7	0.9	(58.9–62.5)
San Antonio-New Braunfels, Texas	922	65.4	2.1	(61.3–69.5)
San Francisco-Redwood City-South San Francisco, California^†^	538	66.4	2.8	(60.9–71.8)
San Jose-Sunnyvale-Santa Clara, California	626	63.2	2.6	(58.2–68.2)
San Juan-Carolina-Caguas, Puerto Rico	3,609	75.5	1.0	(73.5–77.4)
Scottsbluff, Nebraska	698	54.3	2.8	(48.7–59.8)
Scranton-Wilkes-Barre-Hazleton, Pennsylvania	563	71.5	3.0	(65.7–77.4)
Seattle-Bellevue-Everett, Washington^†^	3,717	61.7	1.1	(59.6–63.8)
Shreveport-Bossier City, Louisiana	567	73.9	3.2	(67.6–80.3)
Silver Spring-Frederick-Rockville, Maryland^†^	2,410	73.4	1.5	(70.6–76.3)
Sioux City, Iowa-Nebraska-South Dakota	1,044	69.7	3.0	(63.8–75.7)
Sioux Falls, South Dakota	1,002	69.8	2.0	(65.8–73.7)
Spartanburg, South Carolina	586	64.6	3.1	(58.6–70.7)
Spokane-Spokane Valley, Washington	852	60.5	2.4	(55.8–65.2)
Springfield, Massachusetts	1,564	75.8	2.1	(71.8–79.9)
Tallahassee, Florida	1,843	70.4	2.2	(66.0–74.8)
Tampa-St. Petersburg-Clearwater, Florida	2,191	68.2	1.5	(65.2–71.2)
Toledo, Ohio	985	68.6	2.5	(63.6–73.5)
Topeka, Kansas	2,383	70.1	1.3	(67.5–72.6)
Tulsa, Oklahoma	1,969	61.0	1.6	(57.9–64.2)
Virginia Beach-Norfolk-Newport News, Virginia-North Carolina	1,662	74.5	1.6	(71.3–77.6)
Warren-Troy-Farmington Hills, Michigan^†^	2,239	70.3	1.4	(67.6–73.1)
Washington-Arlington-Alexandria, District of Columbia-Virginia-Maryland-West Virginia^†^	8,946	74.7	0.9	(72.9–76.5)
Wichita, Kansas	4,842	69.5	0.9	(67.7–71.2)
Wilmington, Delaware-Maryland-New Jersey^†^	3,244	75.1	1.1	(72.9–77.3)
Winston-Salem, North Carolina	691	72.6	2.8	(67.1–78.2)
Worcester, Massachusetts-Connecticut	2,750	75.9	1.4	(73.1–78.7)
*Median*		*68.9*		
*Range*		*52.6–79.5*		

**Abbreviations:** CI = confidence interval; MMSA = metropolitan and micropolitan statistical areas; SE = standard error.

* Age adjusted to the 2000 U.S. standard population.

^†^ Metropolitan division.

**TABLE 15:** Age-adjusted* prevalence estimates of adults aged ≥18 years who visited a doctor for a routine checkup during the past 12 months, by state/territory — Behavioral Risk Factor Surveillance System, United States, 2014

State/Territory	Sample size	%	SE	95% CI
Alabama	8,477	71.4	0.8	(69.8–73.0)
Alaska	4,334	58.7	1.1	(56.6–60.8)
Arizona	14,664	63.4	0.7	(61.9–64.8)
Arkansas	5,127	66.2	1.2	(63.8–68.5)
California	8,762	67.3	0.7	(66.0–68.6)
Colorado	13,141	63.1	0.6	(62.0–64.2)
Connecticut	7,885	71.3	0.8	(69.6–72.9)
Delaware	4,240	72.9	1.1	(70.7–75.2)
District of Columbia	4,033	75.0	1.2	(72.7–77.3)
Florida	9,733	70.9	0.7	(69.4–72.3)
Georgia	6,262	72.3	0.8	(70.7–74.0)
Hawaii	7,207	66.7	0.8	(65.1–68.3)
Idaho	5,433	57.2	1.0	(55.2–59.2)
Illinois	5,045	69.0	0.9	(67.2–70.8)
Indiana	11,415	64.2	0.7	(62.9–65.5)
Iowa	8,028	70.3	0.7	(68.8–71.7)
Kansas	13,395	67.7	0.6	(66.6–68.8)
Kentucky	11,009	71.4	0.8	(69.8–73.1)
Louisiana	6,684	75.2	0.7	(73.8–76.6)
Maine	9,090	69.2	0.8	(67.6–70.8)
Maryland	12,466	75.0	0.8	(73.4–76.6)
Massachusetts	15,457	77.7	0.6	(76.5–78.8)
Michigan	8,370	70.7	0.7	(69.3–72.1)
Minnesota	16,220	69.2	0.5	(68.2–70.1)
Mississippi	4,131	72.3	1.1	(70.1–74.4)
Missouri	6,952	66.4	0.9	(64.5–68.2)
Montana	7,382	62.4	0.9	(60.6–64.2)
Nebraska	22,090	62.9	0.6	(61.8–64.1)
Nevada	3,700	64.0	1.3	(61.4–66.6)
New Hampshire	6,114	66.8	1.0	(64.8–68.8)
New Jersey	12,876	75.8	0.6	(74.6–77.1)
New Mexico	8,785	62.0	0.9	(60.2–63.7)
New York	6,801	74.4	0.7	(73.0–75.8)
North Carolina	7,192	75.1	0.7	(73.8–76.4)
North Dakota	7,720	63.8	1.0	(61.9–65.7)
Ohio	10,777	69.6	0.8	(68.0–71.1)
Oklahoma	8,303	64.0	0.7	(62.5–65.4)
Oregon	5,066	61.8	1.0	(59.9–63.8)
Pennsylvania	10,904	71.0	0.7	(69.6–72.4)
Rhode Island	6,406	79.2	0.9	(77.5–80.9)
South Carolina	10,882	66.9	0.7	(65.5–68.2)
South Dakota	7,290	69.4	1.1	(67.3–71.5)
Tennessee	5,075	73.8	1.0	(71.7–75.8)
Texas	15,130	69.1	0.7	(67.8–70.4)
Utah	14,714	60.2	0.5	(59.2–61.2)
Vermont	6,412	66.1	0.8	(64.5–67.7)
Virginia	9,383	73.1	0.7	(71.8–74.5)
Washington	9,944	63.7	0.7	(62.2–65.1)
West Virginia	6,129	75.3	0.8	(73.7–76.9)
Wisconsin	7,013	69.2	0.9	(67.5–70.9)
Wyoming	6,302	58.0	1.2	(55.7–60.4)
Guam	2,503	61.6	1.4	(58.9–64.3)
Puerto Rico	5,906	76.0	0.8	(74.5–77.6)
*Median*		*69.2*		
*Range*		*57.2–79.2*		

**Abbreviations:** CI = confidence interval; SE = standard error.

* Age adjusted to the 2000 U.S. standard population.

**TABLE 16:** Age-adjusted* prevalence estimates of adults aged ≥18 years who visited a doctor for a routine checkup during the past 12 months, by metropolitan and micropolitan statistical area — Behavioral Risk Factor Surveillance System, United States, 2014

MMSA	Sample size	%	SE	95% CI
Aberdeen, South Dakota	609	66.3	3.2	(60.1–72.5)
Aguadilla-Isabela, Puerto Rico	535	78.6	2.4	(73.9–83.2)
Albuquerque, New Mexico	1,765	62.8	1.6	(59.6–66.0)
Allentown-Bethlehem-Easton, Pennsylvania-New Jersey	1,082	68.2	2.6	(63.0–73.3)
Anchorage, Alaska	1,767	59.4	1.5	(56.5–62.4)
Atlanta-Sandy Springs-Roswell, Georgia	2,752	72.1	1.2	(69.8–74.4)
Augusta-Richmond County, Georgia-South Carolina	876	67.5	2.8	(61.9–73.0)
Austin-Round Rock, Texas	2,226	69.3	1.4	(66.6–72.0)
Baltimore-Columbia-Towson, Maryland	4,585	75.5	1.3	(73.0–77.9)
Baton Rouge, Louisiana	909	77.4	1.8	(73.9–80.9)
Berlin, New Hampshire-Vermont	537	64.7	4.7	(55.5–73.8)
Billings, Montana	796	63.8	2.4	(59.1–68.4)
Birmingham-Hoover, Alabama	1,553	73.7	1.6	(70.6–76.9)
Bismarck, North Dakota	1,023	60.4	2.4	(55.7–65.0)
Boise City, Idaho	1,343	60.6	2.0	(56.7–64.4)
Boston, Massachusetts^†^	4,491	77.6	1.1	(75.4–79.8)
Burlington-South Burlington, Vermont	1,961	65.8	1.4	(63.2–68.5)
Cambridge-Newton-Framingham, Massachusetts^†^	5,108	76.4	1.0	(74.4–78.4)
Camden, New Jersey^†^	1,705	76.9	1.8	(73.4–80.5)
Cedar Rapids, Iowa	632	68.6	2.7	(63.4–73.8)
Charleston, West Virginia	870	79.6	1.9	(76.0–83.3)
Charleston-North Charleston, South Carolina	1,380	68.6	1.8	(65.1–72.1)
Charlotte-Concord-Gastonia, North Carolina-South Carolina	2,126	71.7	1.4	(69.1–74.4)
Chicago-Naperville-Elgin, Illinois-Indiana-Wisconsin	4,109	69.6	1.1	(67.5–71.7)
Cincinnati, Ohio-Kentucky-Indiana	2,019	69.5	1.9	(65.8–73.1)
Claremont-Lebanon, New Hampshire-Vermont	1,672	63.4	2.1	(59.3–67.6)
Cleveland-Elyria, Ohio	957	71.9	2.2	(67.6–76.1)
College Station-Bryan, Texas	572	70.1	3.8	(62.7–77.4)
Colorado Springs, Colorado	1,276	68.3	1.8	(64.9–71.8)
Columbia, South Carolina	1,195	68.3	1.9	(64.7–72.0)
Columbus, Ohio	1,641	70.7	1.8	(67.2–74.1)
Corpus Christi, Texas	611	69.5	3.6	(62.4–76.6)
Dallas-Plano-Irving, Texas^†^	1,273	72.1	1.9	(68.3–75.9)
Dayton, Ohio	553	67.5	3.0	(61.6–73.4)
Denver-Aurora-Lakewood, Colorado	5,705	62.6	0.8	(61.0–64.2)
Des Moines-West Des Moines, Iowa	1,340	71.9	1.8	(68.4–75.5)
Duluth, Minnesota-Wisconsin	938	69.7	2.1	(65.5–73.9)
El Paso, Texas	713	63.8	2.6	(58.7–68.8)
Evansville, Indiana-Kentucky	652	74.4	3.3	(68.0–80.9)
Fargo, North Dakota-Minnesota	1,142	70.0	1.9	(66.2–73.8)
Fayetteville-Springdale-Rogers, Arkansas-Missouri	797	65.2	3.0	(59.3–71.1)
Fort Wayne, Indiana	856	60.8	2.6	(55.7–65.9)
Fort Worth-Arlington, Texas^†^	752	67.6	2.6	(62.5–72.7)
Grand Island, Nebraska	1,051	63.7	2.5	(58.9–68.6)
Grand Rapids-Wyoming, Michigan	886	70.9	2.2	(66.6–75.2)
Greensboro-High Point, North Carolina	510	73.6	2.7	(68.2–78.9)
Greenville-Anderson-Mauldin, South Carolina	1,474	63.5	1.7	(60.1–66.9)
Hagerstown-Martinsburg, Maryland-West Virginia	774	73.8	3.0	(67.9–79.6)
Hartford-West Hartford-East Hartford, Connecticut	2,625	72.3	1.4	(69.6–74.9)
Hilton Head Island-Bluffton-Beaufort, South Carolina	550	69.8	3.4	(63.2–76.4)
Houston-The Woodlands-Sugar Land, Texas	2,117	70.2	1.6	(67.1–73.4)
Huntington-Ashland, West Virginia-Kentucky-Ohio	1,230	73.8	1.9	(70.1–77.6)
Idaho Falls, Idaho	514	60.4	2.8	(55.0–65.8)
Indianapolis-Carmel-Anderson, Indiana	3,585	65.2	1.2	(62.8–67.5)
Jacksonville, Florida	668	73.7	2.5	(68.7–78.6)
Kansas City, Missouri-Kansas	4,804	66.4	1.3	(63.9–69.0)
Kingsport-Bristol-Bristol, Tennessee-Virginia	497	75.2	3.7	(68.0–82.5)
Knoxville, Tennessee	559	77.0	3.3	(70.5–83.4)
Lafayette, Louisiana	553	76.1	2.3	(71.6–80.6)
Lexington-Fayette, Kentucky	614	70.6	2.5	(65.7–75.4)
Lincoln, Nebraska	1,983	59.9	1.5	(57.1–62.8)
Little Rock-North Little Rock-Conway, Arkansas	1,167	69.7	2.1	(65.5–73.8)
Logan, Utah-Idaho	614	58.0	2.2	(53.6–62.4)
Los Angeles-Long Beach-Anaheim, California	2,434	68.4	1.2	(66.0–70.7)
Louisville-Jefferson County, Kentucky-Indiana	2,436	71.8	1.8	(68.2–75.4)
Madison, Wisconsin	549	72.8	2.5	(68.0–77.7)
Memphis, Tennessee-Mississippi-Arkansas	870	76.7	2.7	(71.4–82.0)
Miami-Fort Lauderdale-West Palm Beach, Florida	2,200	73.8	1.4	(71.0–76.6)
Milwaukee-Waukesha-West Allis, Wisconsin	1,354	71.8	1.9	(68.1–75.6)
Minneapolis-St. Paul-Bloomington, Minnesota-Wisconsin	8,655	69.0	0.6	(67.8–70.3)
Minot, North Dakota	594	67.2	2.8	(61.7–72.8)
Montgomery, Alabama	508	76.2	3.0	(70.2–82.1)
Montgomery County-Bucks County-Chester County, Pennsylvania^†^	799	68.8	2.4	(64.1–73.5)
Myrtle Beach-Conway-North Myrtle Beach, South Carolina-North Carolina	991	68.7	2.2	(64.5–73.0)
Nashville-Davidson County-Murfreesboro-Franklin, Tennessee	795	74.5	2.2	(70.1–78.9)
Nassau County-Suffolk County, New York^†^	767	71.7	2.3	(67.2–76.1)
Newark, New Jersey-Pennsylvania^†^	4,093	75.7	1.1	(73.6–77.8)
New Orleans-Metairie, Louisiana	1,888	75.3	1.4	(72.6–77.9)
New York-Jersey City-White Plains, New York-New Jersey^†^	7,453	75.7	0.8	(74.2–77.2)
Norfolk, Nebraska	982	57.5	2.0	(53.5–61.4)
North Platte, Nebraska	948	56.8	2.2	(52.6–61.1)
North Port-Sarasota-Bradenton, Florida	510	61.7	4.2	(53.5–69.9)
Oakland-Hayward-Berkeley, California^†^	696	69.0	2.4	(64.3–73.6)
Ogden-Clearfield, Utah	2,873	62.3	1.0	(60.2–64.3)
Oklahoma City, Oklahoma	2,397	64.0	1.3	(61.5–66.6)
Omaha-Council Bluffs, Nebraska-Iowa	4,824	67.6	1.0	(65.7–69.6)
Orlando-Kissimmee-Sanford, Florida	949	70.7	2.1	(66.5–74.9)
Philadelphia, Pennsylvania^†^	1,509	72.4	1.7	(69.0–75.8)
Phoenix-Mesa-Scottsdale, Arizona	9,277	63.5	0.9	(61.8–65.2)
Pittsburgh, Pennsylvania	2,398	72.1	1.4	(69.4–74.8)
Ponce, Puerto Rico	524	75.8	2.8	(70.4–81.3)
Portland-South Portland, Maine	2,746	70.3	1.4	(67.5–73.0)
Portland-Vancouver-Hillsboro, Oregon-Washington	2,765	63.0	1.4	(60.4–65.7)
Providence-Warwick, Rhode Island-Massachusetts	8,051	80.8	0.9	(79.1–82.5)
Provo-Orem, Utah	2,107	57.8	1.2	(55.4–60.2)
Raleigh, North Carolina	714	73.5	2.0	(69.6–77.4)
Rapid City, South Dakota	1,403	66.5	2.1	(62.3–70.7)
Reno, Nevada	1,193	62.3	2.0	(58.4–66.2)
Richmond, Virginia	1,451	72.4	1.7	(69.0–75.8)
Riverside-San Bernardino-Ontario, California	937	69.5	1.9	(65.8–73.3)
Roanoke, Virginia	524	74.0	3.0	(68.2–79.8)
Rochester, Minnesota	699	64.5	2.3	(60.0–69.0)
Rockingham County-Strafford County, New Hampshire^†^	1,424	68.9	1.9	(65.1–72.7)
Sacramento-Roseville-Arden-Arcade, California	639	65.9	2.4	(61.2–70.7)
St. Cloud, Minnesota	558	72.3	2.3	(67.9–76.8)
St. Louis, Missouri-Illinois	1,911	71.5	1.8	(68.1–75.0)
Salisbury, Maryland-Delaware	1,937	74.0	2.3	(69.5–78.4)
Salt Lake City, Utah	5,326	60.6	0.8	(59.1–62.1)
San Antonio-New Braunfels, Texas	2,241	69.6	1.4	(66.9–72.3)
San Juan-Carolina-Caguas, Puerto Rico	3,699	75.0	1.0	(73.1–77.0)
Scottsbluff, Nebraska	884	55.9	2.4	(51.2–60.6)
Seattle-Bellevue-Everett, Washington^†^	3,653	63.7	1.1	(61.5–66.0)
Shreveport-Bossier City, Louisiana	547	72.8	2.7	(67.4–78.1)
Silver Spring-Frederick-Rockville, Maryland^†^	2,357	72.4	1.7	(69.0–75.8)
Sioux City, Iowa-Nebraska-South Dakota	1,126	76.4	2.6	(71.4–81.4)
Sioux Falls, South Dakota	1,337	73.7	2.1	(69.7–77.8)
Spartanburg, South Carolina	562	63.9	3.3	(57.4–70.4)
Spokane-Spokane Valley, Washington	712	62.1	2.7	(56.9–67.3)
Springfield, Massachusetts	1,095	78.6	2.0	(74.7–82.5)
Tampa-St. Petersburg-Clearwater, Florida	1,574	69.6	1.8	(66.0–73.2)
Toledo, Ohio	639	65.8	3.1	(59.7–71.9)
Topeka, Kansas	1,425	67.3	1.7	(64.0–70.6)
Tulsa, Oklahoma	2,002	61.8	1.4	(59.0–64.6)
Tuscaloosa, Alabama	708	72.1	2.4	(67.4–76.9)
Virginia Beach-Norfolk-Newport News, Virginia-North Carolina	1,872	77.8	1.5	(74.9–80.6)
Warren-Troy-Farmington Hills, Michigan^†^	2,095	71.0	1.4	(68.2–73.8)
Washington-Arlington-Alexandria, District of Columbia-Virginia-Maryland-West Virginia^†^	8,231	73.8	0.9	(71.9–75.6)
Wichita, Kansas	2,667	69.5	1.2	(67.2–71.8)
Wichita Falls, Texas	540	76.7	4.2	(68.5–84.8)
Wilmington, Delaware-Maryland-New Jersey^†^	2,733	73.5	1.4	(70.8–76.3)
Worcester, Massachusetts-Connecticut	2,437	77.1	1.4	(74.4–79.8)
Youngstown-Warren-Boardman, Ohio-Pennsylvania	523	72.3	3.4	(65.6–79.1)
*Median*		*69.8*		
*Range*		*55.9–80.8*		

**Abbreviations:** CI = confidence interval; MMSA = metropolitan and micropolitan statistical areas; SE = standard error.

* Age adjusted to the 2000 U.S. standard population.

^† ^Metropolitan division.

#### Pap Test

A Pap test detects cancer of the cervix. The U.S. Preventive Services Task Force (USPSTF) recommends that women aged ≥21 years should receive a Pap test to screen for cervical cancer at least every 3 years until aged 65 years or a Pap test in combination with a human papillomavirus test every 5 years for women aged 30–65 years (*16*). Women aged 21–65 years who self-reported ever having a Pap test were included in this report. In 2014, age-adjusted prevalence estimates of a Pap test among women aged 21–65 years ranged from 67.7% in Guam to 87.8% in Massachusetts (median: 82.4%) (Table 17). Among selected MMSA, age-adjusted prevalence estimates ranged from 68.0% in Wichita Falls, Texas, to 94.3% in Knoxville, Tennessee (median: 83.1%) (Table 18).

**TABLE 17:** Age-adjusted* prevalence estimates of women aged 21–65 years who reported having a Pap^†^ test, by state/territory — Behavioral Risk Factor Surveillance System, United States, 2014

State/Territory	Sample size	%	SE	95% CI
Alabama	2,056	82.9	1.1	(80.8–85.0)
Alaska	1,420	78.6	1.6	(75.5–81.8)
Arizona	3,342	79.8	1.1	(77.6–81.9)
Arkansas	1,042	77.7	1.8	(74.1–81.3)
California	2,120	83.0	1.1	(80.9–85.1)
Colorado	3,460	84.6	0.8	(83.1–86.2)
Connecticut	2,142	87.5	1.0	(85.5–89.5)
Delaware	1,184	86.4	1.5	(83.5–89.3)
District of Columbia	1,108	85.1	1.7	(81.8–88.5)
Florida	2,304	79.6	1.1	(77.4–81.8)
Georgia	1,563	84.6	1.1	(82.3–86.8)
Hawaii	2,010	77.8	1.4	(75.1–80.6)
Idaho	1,353	76.0	1.5	(73.0–78.9)
Illinois	1,460	81.5	1.4	(78.7–84.2)
Indiana	2,811	78.0	1.0	(75.9–80.0)
Iowa	2,118	84.5	1.1	(82.4–86.6)
Kansas	3,401	81.8	0.8	(80.2–83.5)
Kentucky	2,993	81.4	1.2	(79.1–83.7)
Louisiana	1,761	83.7	1.1	(81.5–85.8)
Maine	2,493	85.2	1.0	(83.1–87.2)
Maryland	3,676	86.7	1.2	(84.3–89.0)
Massachusetts	4,424	87.8	0.8	(86.1–89.4)
Michigan	2,233	83.4	1.0	(81.4–85.4)
Minnesota	4,746	86.1	0.6	(84.9–87.3)
Mississippi	1,011	83.0	1.5	(80.1–85.9)
Missouri	1,737	80.8	1.3	(78.2–83.3)
Montana	1,778	81.5	1.3	(78.9–84.1)
Nebraska	5,779	81.7	0.8	(80.1–83.4)
Nevada	993	78.1	2.1	(73.9–82.2)
New Hampshire	1,765	85.1	1.4	(82.3–87.9)
New Jersey	3,976	83.8	0.9	(82.1–85.6)
New Mexico	2,394	79.0	1.3	(76.5–81.5)
New York	2,073	82.6	1.1	(80.3–84.8)
North Carolina	1,898	85.8	0.9	(84.0–87.6)
North Dakota	1,959	81.5	1.5	(78.6–84.3)
Ohio	2,943	81.6	1.1	(79.5–83.7)
Oklahoma	2,070	77.0	1.2	(74.7–79.3)
Oregon	1,226	83.0	1.3	(80.4–85.6)
Pennsylvania	2,918	80.8	1.1	(78.5–83.0)
Rhode Island	1,925	85.9	1.2	(83.4–88.3)
South Carolina	2,655	82.4	1.0	(80.5–84.4)
South Dakota	2,012	84.8	1.5	(81.8–87.7)
Tennessee	1,210	85.4	1.4	(82.7–88.1)
Texas	3,959	77.7	1.1	(75.6–79.8)
Utah	4,205	77.2	0.8	(75.6–78.8)
Vermont	2,038	85.8	1.0	(83.8–87.8)
Virginia	2,773	85.2	1.0	(83.3–87.1)
Washington	2,657	80.9	1.1	(78.8–83.1)
West Virginia	1,656	80.5	1.2	(78.2–82.8)
Wisconsin	1,831	86.7	1.1	(84.4–88.9)
Wyoming	1,352	81.2	1.5	(78.2–84.2)
Guam	955	67.7	2.2	(63.5–72.0)
Puerto Rico	1,880	77.4	1.2	(75.1–79.7)
*Median*		*82.4*		
*Range*		*67.7–87.8*		

**Abbreviations:** CI = confidence interval; Pap = Papanicolaou; SE = standard error.

* Age adjusted to the 2000 U.S. standard population.

^†^ Test for cancer of the cervix.

**TABLE 18:** Age-adjusted* prevalence estimates of women aged 21–65 years who reported having a Pap^†^ test, by metropolitan and micropolitan statistical area — Behavioral Risk Factor Surveillance System, United States, 2014

MMSA	Sample size	%	SE	95% CI
Aberdeen, South Dakota	161	84.8	4.7	(75.6–94.0)
Aguadilla-Isabela, Puerto Rico	176	68.2	4.3	(59.7–76.6)
Albuquerque, New Mexico	509	80.8	2.3	(76.2–85.4)
Allentown-Bethlehem-Easton, Pennsylvania-New Jersey	302	88.7	2.4	(84.1–93.4)
Anchorage, Alaska	568	78.2	2.4	(73.5–82.8)
Atlanta-Sandy Springs-Roswell, Georgia	747	86.6	1.5	(83.7–89.6)
Augusta-Richmond County, Georgia-South Carolina	209	88.1	3.2	(81.8–94.5)
Austin-Round Rock, Texas	664	83.3	2.0	(79.4–87.1)
Baltimore-Columbia-Towson, Maryland	1,374	87.3	2.0	(83.3–91.2)
Baton Rouge, Louisiana	248	82.6	2.9	(77.0–88.2)
Berlin, New Hampshire-Vermont	140	90.5	3.3	(84.0–96.9)
Billings, Montana	186	82.9	3.5	(76.0–89.8)
Birmingham-Hoover, Alabama	417	83.7	2.2	(79.3–88.0)
Bismarck, North Dakota	259	86.2	2.5	(81.3–91.2)
Boise City, Idaho	323	80.6	2.7	(75.4–85.8)
Boston, Massachusetts^§^	1,325	86.3	1.7	(83.0–89.6)
Burlington-South Burlington, Vermont	648	86.0	1.8	(82.5–89.4)
Cambridge-Newton-Framingham, Massachusetts^§^	1,461	88.3	1.4	(85.6–91.0)
Camden, New Jersey^§^	541	87.4	2.2	(83.0–91.8)
Cedar Rapids, Iowa	177	87.1	3.6	(80.0–94.2)
Charleston, West Virginia	227	78.5	3.3	(71.9–85.0)
Charleston-North Charleston, South Carolina	382	81.7	2.6	(76.6–86.8)
Charlotte-Concord-Gastonia, North Carolina-South Carolina	541	84.7	1.9	(81.1–88.4)
Chicago-Naperville-Elgin, Illinois-Indiana-Wisconsin	1,217	81.5	1.7	(78.2–84.7)
Cincinnati, Ohio-Kentucky-Indiana	568	82.9	2.5	(78.1–87.8)
Claremont-Lebanon, New Hampshire-Vermont	502	84.8	2.5	(79.9–89.7)
Cleveland-Elyria, Ohio	283	83.5	2.9	(77.8–89.1)
College Station-Bryan, Texas	120	78.5	5.9	(66.9–90.2)
Colorado Springs, Colorado	322	82.8	2.6	(77.7–87.9)
Columbia, South Carolina	369	86.8	2.0	(82.9–90.7)
Columbus, Ohio	526	83.7	2.2	(79.3–88.1)
Corpus Christi, Texas	119	84.1	4.4	(75.5–92.7)
Dallas-Plano-Irving, Texas^§^	349	77.4	3.1	(71.3–83.5)
Dayton, Ohio	146	87.2	4.0	(79.4–95.0)
Denver-Aurora-Lakewood, Colorado	1,601	84.3	1.1	(82.0–86.5)
Des Moines-West Des Moines, Iowa	374	88.0	2.6	(82.9–93.2)
Duluth, Minnesota-Wisconsin	263	81.5	3.0	(75.7–87.3)
El Paso, Texas	187	80.2	3.6	(73.2–87.3)
Evansville, Indiana-Kentucky	119	85.1	6.1	(73.0–97.1)
Fargo, North Dakota-Minnesota	288	81.2	3.3	(74.7–87.7)
Fayetteville-Springdale-Rogers, Arkansas-Missouri	176	71.6	4.9	(62.0–81.3)
Fort Wayne, Indiana	222	73.2	4.1	(65.1–81.3)
Fort Worth-Arlington, Texas^§^	209	82.3	3.3	(75.8–88.8)
Grand Island, Nebraska	277	84.2	3.0	(78.4–90.0)
Grand Rapids-Wyoming, Michigan	218	86.1	3.4	(79.3–92.8)
Greensboro-High Point, North Carolina	144	78.8	4.1	(70.8–86.7)
Greenville-Anderson-Mauldin, South Carolina	314	80.9	2.6	(75.7–86.0)
Hagerstown-Martinsburg, Maryland-West Virginia	183	82.2	5.4	(71.7–92.7)
Hartford-West Hartford-East Hartford, Connecticut	669	90.4	1.4	(87.7–93.1)
Hilton Head Island-Bluffton-Beaufort, South Carolina	114	84.8	4.1	(76.8–92.7)
Houston-The Woodlands-Sugar Land, Texas	522	79.2	2.8	(73.7–84.8)
Huntington-Ashland, West Virginia-Kentucky-Ohio	327	75.8	3.4	(69.2–82.4)
Idaho Falls, Idaho	129	73.2	4.6	(64.2–82.2)
Indianapolis-Carmel-Anderson, Indiana	927	81.1	1.8	(77.6–84.5)
Jacksonville, Florida	184	81.4	3.6	(74.4–88.5)
Kansas City, Missouri-Kansas	1,283	81.9	2.0	(78.0–85.8)
Kingsport-Bristol-Bristol, Tennessee-Virginia	108	86.1	3.4	(79.5–92.7)
Knoxville, Tennessee	117	94.3	2.7	(89.0–99.7)
Lafayette, Louisiana	143	81.8	3.8	(74.4–89.2)
Lexington-Fayette, Kentucky	176	83.9	3.3	(77.5–90.3)
Lincoln, Nebraska	577	78.8	2.3	(74.3–83.2)
Little Rock-North Little Rock-Conway, Arkansas	263	85.9	2.9	(80.3–91.6)
Logan, Utah-Idaho	186	72.2	3.9	(64.6–79.8)
Los Angeles-Long Beach-Anaheim, California	570	82.1	2.0	(78.1–86.1)
Louisville/Jefferson County, Kentucky-Indiana	631	82.2	2.5	(77.2–87.1)
Madison, Wisconsin	145	90.6	3.3	(84.2–97.1)
Memphis, Tennessee-Mississippi-Arkansas	230	90.1	2.3	(85.6–94.7)
Miami-Fort Lauderdale-West Palm Beach, Florida	547	81.0	2.2	(76.7–85.3)
Milwaukee-Waukesha-West Allis, Wisconsin	353	90.3	2.0	(86.4–94.1)
Minneapolis-St. Paul-Bloomington, Minnesota-Wisconsin	2,716	86.9	0.8	(85.3–88.5)
Minot, North Dakota	148	75.7	5.0	(65.8–85.5)
Montgomery, Alabama	132	86.4	3.7	(79.1–93.6)
Montgomery County-Bucks County-Chester County, Pennsylvania^§^	244	80.4	4.5	(71.7–89.2)
Myrtle Beach-Conway-North Myrtle Beach, South Carolina-North Carolina	206	75.3	4.2	(67.0–83.6)
Nashville-Davidson County-Murfreesboro-Franklin, Tennessee	220	86.0	2.8	(80.5–91.5)
Nassau County-Suffolk County, New York^§^	235	81.7	3.2	(75.4–88.0)
Newark, New Jersey-Pennsylvania^§^	1,326	84.1	1.5	(81.2–87.0)
New Orleans-Metairie, Louisiana	513	86.9	1.9	(83.1–90.6)
New York-Jersey City-White Plains, New York-New Jersey^§^	2,369	82.0	1.3	(79.5–84.4)
Norfolk, Nebraska	261	79.9	3.5	(73.1–86.7)
North Platte, Nebraska	232	70.9	3.4	(64.2–77.7)
North Port-Sarasota-Bradenton, Florida	95	81.2	5.2	(71.1–91.4)
Oakland-Hayward-Berkeley, California^§^	193	86.7	3.5	(79.9–93.5)
Ogden-Clearfield, Utah	805	76.7	1.8	(73.2–80.1)
Oklahoma City, Oklahoma	688	80.9	1.7	(77.5–84.3)
Omaha-Council Bluffs, Nebraska-Iowa	1,365	85.0	1.5	(82.1–87.9)
Orlando-Kissimmee-Sanford, Florida	236	82.2	3.4	(75.6–88.8)
Philadelphia, Pennsylvania^§^	445	76.6	3.1	(70.6–82.6)
Phoenix-Mesa-Scottsdale, Arizona	2,173	79.9	1.3	(77.3–82.5)
Pittsburgh, Pennsylvania	645	81.1	2.0	(77.2–84.9)
Ponce, Puerto Rico	183	73.1	3.9	(65.4–80.8)
Portland-South Portland, Maine	774	85.0	2.1	(80.9–89.0)
Portland-Vancouver-Hillsboro, Oregon-Washington	734	86.5	1.7	(83.2–89.9)
Providence-Warwick, Rhode Island-Massachusetts	2,406	87.1	1.1	(84.9–89.4)
Provo-Orem, Utah	640	72.1	2.1	(67.9–76.3)
Raleigh, North Carolina	232	89.3	2.6	(84.3–94.3)
Rapid City, South Dakota	365	81.5	3.2	(75.2–87.7)
Reno, Nevada	337	77.4	3.3	(70.9–84.0)
Richmond, Virginia	452	90.4	1.6	(87.2–93.5)
Riverside-San Bernardino-Ontario, California	219	84.4	2.8	(78.9–89.9)
Roanoke, Virginia	134	79.1	4.4	(70.5–87.7)
Rochester, Minnesota	193	87.7	2.9	(82.1–93.3)
Rockingham County-Strafford County, New Hampshire^§^	429	86.6	2.7	(81.4–91.8)
Sacramento-Roseville-Arden-Arcade, California	152	86.1	3.5	(79.2–93.0)
St. Cloud, Minnesota	147	86.3	3.1	(80.2–92.5)
St. Louis, Missouri-Illinois	515	83.7	2.3	(79.1–88.3)
Salisbury, Maryland-Delaware	436	85.5	2.7	(80.3–90.7)
Salt Lake City, Utah	1,555	81.2	1.2	(78.8–83.5)
San Antonio-New Braunfels, Texas	568	75.8	2.4	(71.1–80.6)
San Juan-Carolina-Caguas, Puerto Rico	1,180	80.5	1.4	(77.8–83.3)
Scottsbluff, Nebraska	199	76.2	3.7	(69.0–83.5)
Seattle-Bellevue-Everett, Washington^§^	1,022	81.3	1.8	(77.9–84.8)
Shreveport-Bossier City, Louisiana	180	80.5	4.2	(72.3–88.6)
Silver Spring-Frederick-Rockville, Maryland^§^	731	84.5	2.7	(79.3–89.7)
Sioux City, Iowa-Nebraska-South Dakota	261	83.7	4.0	(75.8–91.6)
Sioux Falls, South Dakota	383	88.3	2.4	(83.6–92.9)
Spartanburg, South Carolina	112	83.2	6.7	(70.0–96.4)
Spokane-Spokane Valley, Washington	191	82.7	3.7	(75.4–89.9)
Springfield, Massachusetts	302	86.3	3.0	(80.4–92.2)
Tampa-St. Petersburg-Clearwater, Florida	417	78.7	2.7	(73.5–83.9)
Toledo, Ohio	176	81.0	4.1	(73.0–89.0)
Topeka, Kansas	379	81.2	2.6	(76.1–86.3)
Tulsa, Oklahoma	483	77.9	2.2	(73.6–82.2)
Tuscaloosa, Alabama	192	83.4	3.5	(76.6–90.3)
Virginia Beach-Norfolk-Newport News, Virginia-North Carolina	551	85.3	2.1	(81.2–89.5)
Warren-Troy-Farmington Hills, Michigan^§^	587	82.0	1.9	(78.2–85.8)
Washington-Arlington-Alexandria, District of Columbia-Virginia-Maryland-West Virginia^§^	2,495	85.8	1.5	(82.8–88.7)
Wichita, Kansas	660	84.9	1.6	(81.7–88.0)
Wichita Falls, Texas	85	68.0	9.6	(49.3–86.8)
Wilmington, Delaware-Maryland-New Jersey^§^	818	87.1	1.7	(83.9–90.4)
Worcester, Massachusetts-Connecticut	690	86.6	2.4	(81.8–91.3)
Youngstown-Warren-Boardman, Ohio-Pennsylvania	142	83.8	5.0	(74.0–93.7)
*Median*		*83.1*		
*Range*		*68.0–94.3*		

**Abbreviations:** CI = confidence interval; MMSA = metropolitan and micropolitan statistical areas; Pap = Papanicolaou; SE = standard error.

* Age adjusted to the 2000 U.S. standard population.

^†^ Test for cancer of the cervix.

^§^ Metropolitan division.

#### Colorectal Cancer Screening

USPSTF recommends colorectal cancer screening for adults aged 50–75 years using a blood stool test (also known as fecal occult blood test [FOBT]) every year, a colonoscopy every 10 years, or a flexible sigmoidoscopy every 5 years with an FOBT every 3 years (*17*). Adults aged 50–75 years who self-reported ever having a colorectal cancer screening were included in this report. In 2014, age-adjusted prevalence estimates of colorectal cancer screening among adults aged 50–75 years ranged from 42.8% in Guam to 76.7% in Massachusetts (median: 66.3%) (Table 19). Among selected MMSA, age-adjusted prevalence estimates ranged from 49.1% in Ponce, Puerto Rico, to 79.6% in Madison, Wisconsin (median: 68.1%) (Table 20).

**TABLE 19:** Age-adjusted* prevalence estimates of adults aged 50–75 years who received colorectal cancer screening according to most recent guidelines,^†^ by state/territory — Behavioral Risk Factor Surveillance System, United States, 2014

State/Territory	Sample size	%	SE	95% CI
Alabama	4,450	64.8	1.0	(62.9–66.7)
Alaska	2,052	61.5	1.5	(58.6–64.4)
Arizona	7,311	63.3	0.8	(61.7–65.0)
Arkansas	2,556	60.5	1.4	(57.9–63.2)
California	2,909	66.3	1.1	(64.1–68.6)
Colorado	6,267	67.2	0.7	(65.8–68.7)
Connecticut	3,771	73.3	1.0	(71.4–75.2)
Delaware	2,215	71.7	1.2	(69.2–74.1)
District of Columbia	1,904	68.1	1.7	(64.8–71.5)
Florida	4,565	65.9	0.9	(64.1–67.7)
Georgia	3,005	66.8	1.1	(64.6–69.1)
Hawaii	3,198	69.5	1.2	(67.2–71.8)
Idaho	2,643	60.8	1.2	(58.4–63.3)
Illinois	2,385	61.9	1.3	(59.3–64.4)
Indiana	5,518	61.9	0.8	(60.3–63.5)
Iowa	3,876	67.6	0.9	(65.8–69.4)
Kansas	6,398	64.9	0.7	(63.5–66.2)
Kentucky	5,839	67.1	1.0	(65.2–69.1)
Louisiana	3,172	64.5	1.0	(62.5–66.4)
Maine	4,939	75.2	0.8	(73.6–76.9)
Maryland	6,641	71.1	0.9	(69.3–73.0)
Massachusetts	7,350	76.7	0.8	(75.2–78.2)
Michigan	4,329	71.2	0.9	(69.5–72.9)
Minnesota	7,514	71.8	0.6	(70.6–73.0)
Mississippi	2,120	60.3	1.4	(57.5–63.0)
Missouri	3,564	61.9	1.1	(59.7–64.2)
Montana	3,877	62.3	1.1	(60.1–64.4)
Nebraska	10,646	64.4	0.7	(63.0–65.7)
Nevada	1,788	59.3	1.8	(55.7–62.8)
New Hampshire	3,109	74.1	1.0	(72.0–76.1)
New Jersey	5,973	65.4	0.9	(63.6–67.3)
New Mexico	4,279	61.3	1.0	(59.2–63.3)
New York	2,974	68.3	1.1	(66.2–70.4)
North Carolina	3,273	70.7	0.9	(68.9–72.5)
North Dakota	3,817	62.3	1.1	(60.2–64.5)
Ohio	5,653	65.2	0.9	(63.4–67.0)
Oklahoma	4,125	58.0	0.9	(56.2–59.8)
Oregon	2,528	66.8	1.2	(64.5–69.1)
Pennsylvania	5,370	67.0	0.9	(65.3–68.7)
Rhode Island	3,184	74.7	1.0	(72.8–76.6)
South Carolina	5,545	67.0	0.8	(65.4–68.7)
South Dakota	3,431	67.0	1.4	(64.2–69.8)
Tennessee	2,554	64.8	1.3	(62.3–67.2)
Texas	6,669	61.4	1.1	(59.3–63.5)
Utah	5,694	70.5	0.7	(69.1–72.0)
Vermont	3,166	70.8	0.9	(69.0–72.6)
Virginia	4,630	69.2	0.9	(67.5–71.0)
Washington	5,052	69.9	0.9	(68.1–71.6)
West Virginia	3,195	64.1	1.0	(62.1–66.0)
Wisconsin	3,388	73.7	1.0	(71.7–75.7)
Wyoming	3,360	57.3	1.2	(54.9–59.7)
Guam	763	42.8	2.3	(38.3–47.3)
Puerto Rico	2,842	52.2	1.2	(49.9–54.6)
*Median*		*66.3*		
*Range*		*42.8–76.7*		

**Abbreviations:** CI = confidence interval; SE = standard error.

* Age adjusted to the 2000 U.S. standard population.

^†^ Adults aged 50–75 years who had a blood stool test during the past year, sigmoidoscopy during the past 5 years and blood stool test during the past 3 years, or a colonoscopy during the past 10 years.

**TABLE 20:** Age-adjusted* prevalence estimates of adults aged 50–75 years who received colorectal cancer screening according to most recent guidelines,^†^ by metropolitan and micropolitan statistical area — Behavioral Risk Factor Surveillance System, United States, 2014

MMSA	Sample size	%	SE	95% CI
Aberdeen, South Dakota	306	75.7	2.8	(70.3–81.2)
Aguadilla-Isabela, Puerto Rico	258	53.4	3.9	(45.8–61.0)
Albuquerque, New Mexico	832	69.8	2.0	(65.9–73.8)
Allentown-Bethlehem-Easton, Pennsylvania-New Jersey	557	68.0	3.0	(62.2–73.8)
Anchorage, Alaska	794	63.7	2.2	(59.4–67.9)
Atlanta-Sandy Springs-Roswell, Georgia	1,229	69.5	1.7	(66.2–72.8)
Augusta-Richmond County, Georgia-South Carolina	406	69.0	4.0	(61.2–76.8)
Austin-Round Rock, Texas	988	60.1	2.1	(55.9–64.2)
Baltimore-Columbia-Towson, Maryland	2,392	70.9	1.5	(68.0–73.8)
Baton Rouge, Louisiana	389	69.5	2.7	(64.3–74.7)
Berlin, New Hampshire-Vermont	314	67.4	3.3	(60.9–73.9)
Billings, Montana	378	67.2	2.8	(61.7–72.8)
Birmingham-Hoover, Alabama	777	67.5	2.0	(63.5–71.5)
Bismarck, North Dakota	533	62.0	2.6	(56.9–67.0)
Boise City, Idaho	645	63.8	2.5	(58.9–68.7)
Boston, Massachusetts^§^	2,073	74.6	1.5	(71.6–77.6)
Burlington-South Burlington, Vermont	898	76.5	1.7	(73.2–79.7)
Cambridge-Newton-Framingham, Massachusetts^§^	2,406	78.6	1.3	(76.0–81.2)
Camden, New Jersey^§^	839	70.4	2.2	(66.0–74.8)
Cedar Rapids, Iowa	300	72.0	3.2	(65.6–78.3)
Charleston, West Virginia	431	69.6	2.7	(64.4–74.8)
Charleston-North Charleston, South Carolina	661	71.2	2.3	(66.6–75.7)
Charlotte-Concord-Gastonia, North Carolina-South Carolina	950	70.2	2.0	(66.3–74.1)
Chicago-Naperville-Elgin, Illinois-Indiana-Wisconsin	1,904	60.9	1.6	(57.8–64.0)
Cincinnati, Ohio-Kentucky-Indiana	971	66.9	2.2	(62.7–71.2)
Claremont-Lebanon, New Hampshire-Vermont	875	69.8	2.0	(65.9–73.7)
Cleveland-Elyria, Ohio	507	66.1	2.6	(60.9–71.2)
College Station-Bryan, Texas	252	68.2	6.1	(56.2–80.3)
Colorado Springs, Colorado	604	71.1	2.2	(66.7–75.5)
Columbia, South Carolina	598	69.5	2.2	(65.2–73.7)
Columbus, Ohio	806	67.9	2.3	(63.3–72.4)
Corpus Christi, Texas	313	68.9	3.9	(61.2–76.5)
Dallas-Plano-Irving, Texas^§^	584	64.5	3.2	(58.2–70.9)
Dayton, Ohio	276	69.7	3.7	(62.5–76.9)
Denver-Aurora-Lakewood, Colorado	2,556	68.8	1.1	(66.7–70.9)
Des Moines-West Des Moines, Iowa	623	68.3	2.4	(63.7–73.0)
Duluth, Minnesota-Wisconsin	473	67.4	2.7	(62.1–72.7)
El Paso, Texas	311	55.7	3.7	(48.5–62.9)
Evansville, Indiana-Kentucky	360	73.8	3.0	(67.9–79.7)
Fargo, North Dakota-Minnesota	534	71.4	2.5	(66.5–76.3)
Fayetteville-Springdale-Rogers, Arkansas-Missouri	386	60.2	3.3	(53.8–66.7)
Fort Wayne, Indiana	417	61.6	2.9	(55.8–67.3)
Fort Worth-Arlington, Texas^§^	332	70.8	3.8	(63.4–78.2)
Grand Island, Nebraska	507	67.5	2.7	(62.1–72.8)
Grand Rapids-Wyoming, Michigan	451	75.0	2.6	(69.9–80.2)
Greensboro-High Point, North Carolina	244	74.2	3.2	(68.0–80.5)
Greenville-Anderson-Mauldin, South Carolina	714	8.8	2.4	(64.1–73.4)
Hagerstown-Martinsburg, Maryland-West Virginia	411	64.2	3.0	(58.3–70.0)
Hartford-West Hartford-East Hartford, Connecticut	1,226	73.5	1.8	(70.0–77.0)
Hilton Head Island-Bluffton-Beaufort, South Carolina	288	70.9	3.5	(64.1–77.8)
Houston-The Woodlands-Sugar Land, Texas	914	61.7	2.9	(56.1–67.3)
Huntington-Ashland, West Virginia-Kentucky-Ohio	688	66.5	2.2	(62.3–70.8)
Idaho Falls, Idaho	218	68.7	3.7	(61.4–76.0)
Indianapolis-Carmel-Anderson, Indiana	1,711	65.8	1.5	(62.9–68.6)
Jacksonville, Florida	302	70.3	3.4	(63.6–77.0)
Kansas City, Missouri-Kansas	2,364	66.3	1.7	(63.0–69.6)
Kingsport-Bristol-Bristol, Tennessee-Virginia	269	64.6	4.4	(55.9–73.3)
Knoxville, Tennessee	298	66.5	3.7	(59.2–73.8)
Lafayette, Louisiana	266	63.7	3.4	(57.1–70.4)
Lexington-Fayette, Kentucky	274	75.1	3.4	(68.4–81.8)
Lincoln, Nebraska	850	68.1	1.9	(64.4–71.9)
Little Rock-North Little Rock-Conway, Arkansas	573	71.3	2.6	(66.2–76.5)
Logan, Utah-Idaho	218	66.2	3.6	(59.1–73.2)
Los Angeles-Long Beach-Anaheim, California	687	64.0	2.3	(59.5–68.5)
Louisville-Jefferson County, Kentucky-Indiana	1,337	67.7	2.1	(63.5–71.9)
Madison, Wisconsin	235	79.6	3.2	(73.4–85.8)
Memphis, Tennessee-Mississippi-Arkansas	408	72.2	3.0	(66.3–78.2)
Miami-Fort Lauderdale-West Palm Beach, Florida	936	63.4	2.0	(59.5–67.4)
Milwaukee-Waukesha-West Allis, Wisconsin	640	72.5	2.5	(67.5–77.4)
Minneapolis-St. Paul-Bloomington, Minnesota-Wisconsin	3,856	72.9	0.8	(71.3–74.6)
Minot, North Dakota	285	56.3	3.6	(49.2–63.4)
Montgomery, Alabama	243	66.8	4.1	(58.9–74.8)
Montgomery County-Bucks County-Chester County, Pennsylvania^§^	387	63.5	2.8	(58.1–69.0)
Myrtle Beach-Conway-North Myrtle Beach, South Carolina-North Carolina	545	66.3	2.5	(61.5–71.2)
Nashville-Davidson County-Murfreesboro-Franklin, Tennessee	367	65.9	3.1	(59.9–71.9)
Nassau County-Suffolk County, New York^§^	345	62.1	3.0	(56.2–68.0)
Newark, New Jersey-Pennsylvania^§^	1,928	66.2	1.5	(63.3–69.2)
New Orleans-Metairie, Louisiana	931	65.5	1.9	(61.8–69.3)
New York-Jersey City-White Plains, New York-New Jersey^§^	2,989	67.1	1.3	(64.5–69.6)
Norfolk, Nebraska	475	61.9	2.5	(57.0–66.7)
North Platte, Nebraska	488	55.5	2.6	(50.4–60.5)
North Port-Sarasota-Bradenton, Florida	239	65.4	4.1	(57.4–73.3)
Oakland-Hayward-Berkeley, California^§^	245	75.2	3.9	(67.5–82.9)
Ogden-Clearfield, Utah	1,050	75.1	1.5	(72.2–78.0)
Oklahoma City, Oklahoma	1,138	60.9	1.8	(57.4–64.3)
Omaha-Council Bluffs, Nebraska-Iowa	2,242	69.1	1.4	(66.4–71.7)
Orlando-Kissimmee-Sanford, Florida	414	66.8	2.9	(61.2–72.4)
Philadelphia, Pennsylvania^§^	690	63.0	2.8	(57.6–68.4)
Phoenix-Mesa-Scottsdale, Arizona	4,546	64.6	1.0	(62.7–66.5)
Pittsburgh, Pennsylvania	1,183	69.1	1.6	(66.1–72.2)
Ponce, Puerto Rico	273	49.1	3.6	(41.9–56.2)
Portland-South Portland, Maine	1,469	77.0	1.5	(74.1–79.8)
Portland-Vancouver-Hillsboro, Oregon-Washington	1,355	70.0	1.6	(66.9–73.1)
Providence-Warwick, Rhode Island-Massachusetts	4,008	75.7	1.1	(73.6–77.8)
Provo-Orem, Utah	625	68.1	2.1	(64.1–72.2)
Raleigh, North Carolina	273	74.3	3.0	(68.5–80.2)
Rapid City, South Dakota	703	64.8	2.7	(59.5–70.1)
Reno, Nevada	585	65.8	2.4	(61.1–70.5)
Richmond, Virginia	710	69.5	2.2	(65.1–73.9)
Riverside-San Bernardino-Ontario, California	322	61.8	3.3	(55.3–68.3)
Roanoke, Virginia	276	67.9	3.5	(61.0–74.9)
Rochester, Minnesota	314	77.5	2.5	(72.6–82.5)
Rockingham County-Strafford County, New Hampshire^§^	703	75.1	2.0	(71.1–79.1)
Sacramento-Roseville-Arden-Arcade, California	227	68.3	4.3	(59.9–76.6)
St. Cloud, Minnesota	233	72.5	3.5	(65.7–79.3)
St. Louis, Missouri-Illinois	901	66.1	2.3	(61.6–70.6)
Salisbury, Maryland-Delaware	1,104	71.8	1.9	(68.1–75.4)
Salt Lake City, Utah	2,051	71.3	1.2	(69.0–73.6)
San Antonio-New Braunfels, Texas	1,033	65.2	1.9	(61.5–69.0)
San Juan-Carolina-Caguas, Puerto Rico	1,772	53.3	1.5	(50.4–56.3)
Scottsbluff, Nebraska	456	53.0	2.8	(47.6–58.5)
Seattle-Bellevue-Everett, Washington^§^	1,757	69.5	1.5	(66.6–72.4)
Shreveport-Bossier City, Louisiana	243	55.4	3.5	(48.5–62.4)
Silver Spring-Frederick-Rockville, Maryland^§^	1,198	70.7	2.3	(66.1–75.3)
Sioux City, Iowa-Nebraska-South Dakota	539	73.2	3.2	(66.9–79.5)
Sioux Falls, South Dakota	575	69.4	3.0	(63.5–75.2)
Spartanburg, South Carolina	312	61.8	3.7	(54.6–69.0)
Spokane-Spokane Valley, Washington	369	75.1	2.9	(69.4–80.7)
Springfield, Massachusetts	481	77.6	2.8	(72.2–83.0)
Tampa-St. Petersburg-Clearwater, Florida	744	63.8	2.4	(59.1–68.4)
Toledo, Ohio	340	66.2	3.0	(60.3–72.1)
Topeka, Kansas	742	68.0	2.0	(64.0–72.0)
Tulsa, Oklahoma	1,022	63.6	1.7	(60.2–67.0)
Tuscaloosa, Alabama	364	70.4	2.8	(64.9–75.8)
Virginia Beach-Norfolk-Newport News, Virginia-North Carolina	930	77.9	1.7	(74.5–81.3)
Warren-Troy-Farmington Hills, Michigan^§^	1,082	74.2	1.6	(71.0–77.4)
Washington-Arlington-Alexandria, District of Columbia-Virginia-Maryland-West Virginia^§^	3,904	70.4	1.3	(67.8–73.0)
Wichita, Kansas	1,248	67.4	1.6	(64.3–70.5)
Wichita Falls, Texas	273	62.2	4.3	(53.8–70.7)
Wilmington, Delaware-Maryland-New Jersey^§^	1,400	70.0	1.6	(66.9–73.2)
Worcester, Massachusetts-Connecticut	1,177	77.5	1.9	(73.8–81.1)
Youngstown-Warren-Boardman, Ohio-Pennsylvania	268	64.2	3.6	(57.1–71.4)
*Median*		*68.1*		
*Range*		*49.1–79.6*		

**Abbreviations:** CI = confidence interval; MMSA = metropolitan and micropolitan statistical areas; SE = standard error.

* Age adjusted to the 2000 U.S. standard population.

^†^ Adults aged 50–75 years who had a blood stool test during the past year, sigmoidoscopy during the past 5 years and blood stool test during the past 3 years, or a colonoscopy during the past 10 years.

^§^ Metropolitan division.

#### Blood Cholesterol Check 

Respondents were categorized as having had a blood cholesterol check if they had their blood cholesterol checked during the past 5 years. In 2013, age-adjusted prevalence estimates of a blood cholesterol check during the past 5 years ranged from 65.9% in Guam to 81.9% in Massachusetts (median: 74.3%) (Table 21). Among selected MMSA, age-adjusted prevalence estimates ranged from 62.5% in Logan, Utah-Idaho, to 83.5% in Nassau County-Suffolk County, New York (median: 75.6%) (Table 22).

**TABLE 21:** Age-adjusted* prevalence estimates of adults aged ≥18 years who have had their blood cholesterol checked during the past 5 years, by state/territory — Behavioral Risk Factor Surveillance System, United States, 2013

State/Territory	Sample size	%	SE	95% CI
Alabama	6,252	75.2	1.0	(73.3–77.1)
Alaska	4,450	69.5	1.0	(67.6–71.5)
Arizona	4,115	70.7	1.4	(68.0–73.5)
Arkansas	5,102	69.3	1.0	(67.3–71.4)
California	11,315	74.4	0.6	(73.2–75.5)
Colorado	13,179	75.0	0.5	(73.9–76.0)
Connecticut	7,491	80.3	0.8	(78.6–81.9)
Delaware	5,078	78.4	0.9	(76.5–80.2)
District of Columbia	4,805	81.5	1.0	(79.5–83.6)
Florida	33,365	76.3	0.6	(75.1–77.5)
Georgia	7,901	76.6	0.7	(75.2–78.0)
Hawaii	7,685	73.2	0.8	(71.7–74.7)
Idaho	5,462	67.2	1.0	(65.3–69.1)
Illinois	5,553	72.2	0.9	(70.3–74.0)
Indiana	10,045	72.1	0.7	(70.8–73.4)
Iowa	7,950	72.8	0.8	(71.3–74.3)
Kansas	22,455	70.9	0.4	(70.1–71.8)
Kentucky	10,727	74.6	0.7	(73.2–76.0)
Louisiana	5,055	74.3	1.1	(72.1–76.5)
Maine	7,904	77.1	0.8	(75.6–78.7)
Maryland	12,696	79.2	0.7	(77.9–80.5)
Massachusetts	14,591	81.9	0.6	(80.7–83.1)
Michigan	12,394	76.5	0.6	(75.3–77.7)
Minnesota	13,957	74.7	0.7	(73.3–76.0)
Mississippi	7,160	72.4	0.9	(70.7–74.1)
Missouri	6,859	71.2	0.9	(69.5–73.0)
Montana	9,413	70.2	0.7	(68.8–71.6)
Nebraska	16,649	71.8	0.6	(70.6–73.1)
Nevada	4,923	72.3	1.3	(69.8–74.9)
New Hampshire	6,306	78.4	0.9	(76.7–80.1)
New Jersey	12,948	78.8	0.6	(77.6–80.0)
New Mexico	9,080	68.5	0.8	(67.0–70.0)
New York	8,678	79.6	0.7	(78.3–80.9)
North Carolina	8,548	77.2	0.7	(75.9–78.6)
North Dakota	7,561	71.1	0.8	(69.5–72.7)
Ohio	11,632	75.3	0.7	(74.0–76.6)
Oklahoma	7,952	71.4	0.7	(70.0–72.9)
Oregon	5,714	71.6	0.9	(69.8–73.4)
Pennsylvania	11,097	74.1	0.6	(72.9–75.3)
Rhode Island	6,381	79.4	0.9	(77.7–81.2)
South Carolina	10,435	76.2	0.7	(74.8–77.5)
South Dakota	6,698	69.9	1.0	(68.0–71.8)
Tennessee	5,598	78.9	0.9	(77.1–80.6)
Texas	10,582	74.2	0.7	(72.9–75.6)
Utah	12,337	69.6	0.5	(68.6–70.6)
Vermont	6,210	74.7	0.9	(73.0–76.5)
Virginia	8,261	78.4	0.7	(77.1–79.8)
Washington	10,823	72.5	0.6	(71.2–73.7)
West Virginia	5,705	74.9	0.8	(73.3–76.5)
Wisconsin	6,488	73.9	0.9	(72.0–75.7)
Wyoming	6,268	71.5	0.9	(69.7–73.4)
Guam	1,857	65.9	1.4	(63.2–68.6)
Puerto Rico	5,827	78.3	0.8	(76.7–79.8)
*Median*		*74.3*		
*Range*		*65.9–81.9*		

**Abbreviations:** CI = confidence interval; SE = standard error.

* Age adjusted to the 2000 U.S. standard population.

**TABLE 22:** Age-adjusted* prevalence estimates of adults aged ≥18 years who have had their blood cholesterol checked during the past 5 years, metropolitan and micropolitan statistical area— Behavioral Risk Factor Surveillance System, United States, 2013

MMSA	Sample size	%	SE	95% CI
Aguadilla-Isabela, Puerto Rico	580	79.0	2.5	(74.0–84.0)
Akron, Ohio	681	74.0	3.0	(68.0–79.9)
Albuquerque, New Mexico	2,032	69.6	1.4	(67.0–72.3)
Allentown-Bethlehem-Easton, Pennsylvania-New Jersey	993	74.6	2.4	(69.9–79.3)
Anchorage, Alaska	1,483	70.7	1.5	(67.7–73.7)
Atlanta-Sandy Springs-Roswell, Georgia	3,417	77.5	1.1	(75.4–79.6)
Augusta-Richmond County, Georgia-South Carolina	896	82.4	2.2	(78.1–86.8)
Austin-Round Rock, Texas	892	78.4	1.9	(74.6–82.1)
Baltimore-Columbia-Towson, Maryland	4,659	80.7	1.0	(78.7–82.7)
Baton Rouge, Louisiana	892	72.7	2.4	(68.0–77.5)
Billings, Montana	796	72.2	1.8	(68.6–75.8)
Birmingham-Hoover, Alabama	1,294	77.9	1.8	(74.5–81.3)
Bismarck, North Dakota	1,016	73.4	1.9	(69.7–77.1)
Boise City, Idaho	1,443	71.1	1.7	(67.7–74.4)
Boston, Massachusetts^†^	3,936	81.9	1.1	(79.8–84.0)
Buffalo-Cheektowaga-Niagara Falls, New York	486	80.2	2.7	(74.9–85.5)
Burlington-South Burlington, Vermont	1,587	76.4	1.5	(73.4–79.3)
Cambridge-Newton-Framingham, Massachusetts^†^	4,741	82.6	1.0	(80.6–84.6)
Camden, New Jersey^†^	1,802	80.7	1.5	(77.8–83.5)
Cedar Rapids, Iowa	630	74.0	2.6	(68.9–79.1)
Charleston, West Virginia	794	78.8	2.1	(74.7–82.8)
Charleston-North Charleston, South Carolina	1,503	78.7	1.6	(75.6–81.8)
Charlotte-Concord-Gastonia, North Carolina-South Carolina	1,876	80.0	1.4	(77.3–82.7)
Chattanooga, Tennessee-Georgia	566	78.9	3.3	(72.4–85.4)
Chicago-Naperville-Elgin, Illinois-Indiana-Wisconsin	3,289	73.1	1.2	(70.7–75.5)
Cincinnati, Ohio-Kentucky-Indiana	2,540	76.1	1.4	(73.3–78.9)
Claremont-Lebanon, New Hampshire-Vermont	1,638	73.8	2.0	(69.9–77.7)
Cleveland-Elyria, Ohio	1,089	78.7	1.9	(74.9–82.4)
Colorado Springs, Colorado	1,335	74.7	1.6	(71.5–77.8)
Columbia, South Carolina	1,411	79.3	1.6	(76.1–82.5)
Columbus, Ohio	1,806	76.9	1.3	(74.4–79.5)
Crestview-Fort Walton Beach-Destin, Florida	1,058	72.8	2.1	(68.6–77.0)
Dallas-Plano-Irving, Texas^†^	872	76.6	1.9	(72.9–80.2)
Davenport-Moline-Rock Island, Iowa-Illinois	662	71.1	2.8	(65.6–76.6)
Dayton, Ohio	818	78.7	2.4	(73.9–83.4)
Deltona-Daytona Beach-Ormond Beach, Florida	1,095	73.9	2.7	(68.5–79.2)
Denver-Aurora-Lakewood, Colorado	5,517	77.1	0.8	(75.6–78.6)
Des Moines-West Des Moines, Iowa	1,308	81.2	1.6	(78.0–84.3)
Duluth, Minnesota-Wisconsin	679	77.7	3.1	(71.6–83.8)
Durham-Chapel Hill, North Carolina	607	79.3	2.5	(74.5–84.2)
El Paso, Texas	736	69.7	2.4	(65.0–74.5)
Evansville, Indiana-Kentucky	558	71.4	3.0	(65.5–77.4)
Fargo, North Dakota-Minnesota	1,151	73.4	2.0	(69.5–77.3)
Fayetteville-Springdale-Rogers, Arkansas-Missouri	789	64.2	2.4	(59.5–69.0)
Fort Smith, Arkansas-Oklahoma	481	70.3	3.3	(63.8–76.8)
Fort Wayne, Indiana	756	71.1	2.3	(66.5–75.7)
Fort Worth-Arlington, Texas^†^	792	74.4	2.3	(69.8–79.0)
Gainesville, Florida	1,008	78.2	2.4	(73.5–82.9)
Grand Forks, North Dakota-Minnesota	485	76.2	3.0	(70.3–82.2)
Grand Island, Nebraska	777	72.9	2.6	(67.8–78.0)
Grand Rapids-Wyoming, Michigan	1,314	78.6	1.7	(75.3–81.8)
Greensboro-High Point, North Carolina	644	76.3	2.5	(71.5–81.1)
Greenville-Anderson-Mauldin, South Carolina	1,320	74.7	1.8	(71.3–78.2)
Gulfport-Biloxi-Pascagoula, Mississippi	746	69.5	2.4	(64.7–74.2)
Hagerstown-Martinsburg, Maryland-West Virginia	750	78.6	2.7	(73.3–83.8)
Hartford-West Hartford-East Hartford, Connecticut	2,761	80.6	1.4	(77.9–83.3)
Hilton Head Island-Bluffton-Beaufort, South Carolina	804	74.4	3.3	(68.0–80.8)
Houston-The Woodlands-Sugar Land, Texas	1,354	76.7	1.7	(73.4–80.1)
Huntington-Ashland, West Virginia-Kentucky-Ohio	1,136	74.9	1.8	(71.5–78.4)
Idaho Falls, Idaho	496	66.8	2.8	(61.3–72.2)
Indianapolis-Carmel-Anderson, Indiana	2,480	74.2	1.2	(71.9–76.6)
Jackson, Mississippi	772	74.6	2.2	(70.3–79.0)
Jacksonville, Florida	2,823	75.4	1.3	(72.8–78.0)
Kansas City, Missouri-Kansas	7,214	75.7	1.1	(73.6–77.7)
Kingsport-Bristol-Bristol, Tennessee-Virginia	520	82.7	3.3	(76.3–89.1)
Knoxville, Tennessee	629	77.2	2.5	(72.3–82.1)
Lansing-East Lansing, Michigan	664	76.2	2.3	(71.7–80.6)
Lexington-Fayette, Kentucky	616	72.4	2.3	(67.9–76.8)
Lincoln, Nebraska	1,825	72.4	1.3	(69.9–74.9)
Little Rock-North Little Rock-Conway, Arkansas	1,111	75.8	2.0	(72.0–79.6)
Logan, Utah-Idaho	619	62.5	2.2	(58.1–66.9)
Los Angeles-Long Beach-Anaheim, California	2,983	75.9	1.1	(73.8–77.9)
Louisville-Jefferson County, Kentucky-Indiana	2,093	75.9	1.6	(72.7–79.2)
Lubbock, Texas	514	71.0	3.1	(64.9–77.1)
Manhattan, Kansas	639	68.6	2.1	(64.5–72.7)
Memphis, Tennessee-Mississippi-Arkansas	1,166	77.5	2.1	(73.5–81.6)
Miami-Fort Lauderdale-West Palm Beach, Florida	2,165	77.3	1.6	(74.2–80.4)
Milwaukee-Waukesha-West Allis, Wisconsin	1,253	72.5	2.0	(68.5–76.4)
Minneapolis-St. Paul-Bloomington, Minnesota-Wisconsin	8,891	76.7	0.9	(75.0–78.4)
Minot, North Dakota	629	70.9	2.4	(66.2–75.6)
Montgomery County-Bucks County-Chester County, Pennsylvania^†^	944	80.4	1.8	(76.8–84.0)
Myrtle Beach-Conway-North Myrtle Beach, South Carolina-North Carolina	754	72.5	2.5	(67.5–77.5)
Nashville-Davidson County-Murfreesboro-Franklin, Tennessee	1,017	80.4	1.6	(77.1–83.6)
Nassau County-Suffolk County, New York^†^	920	83.5	1.7	(80.1–86.9)
Newark, New Jersey-Pennsylvania^†^	3,978	79.4	1.0	(77.3–81.4)
New Orleans-Metairie, Louisiana	1,241	76.4	2.3	(71.9–80.9)
New York-Jersey City-White Plains, New York-New Jersey^†^	8,668	79.9	0.7	(78.6–81.3)
Norfolk, Nebraska	657	73.5	2.2	(69.2–77.8)
North Platte, Nebraska	698	67.9	2.8	(62.5–73.3)
North Port-Sarasota-Bradenton, Florida	1,063	71.4	2.3	(66.8–76.0)
Oakland-Hayward-Berkeley, California^†^	684	74.5	2.2	(70.2–78.9)
Ogden-Clearfield, Utah	2,393	73.4	1.1	(71.2–75.6)
Oklahoma City, Oklahoma	2,563	73.1	1.2	(70.7–75.5)
Omaha-Council Bluffs, Nebraska-Iowa	3,047	74.4	1.2	(72.2–76.7)
Orlando-Kissimmee-Sanford, Florida	2,234	75.6	1.5	(72.7–78.5)
Panama City, Florida	1,001	74.7	2.3	(70.1–79.2)
Pensacola-Ferry Pass-Brent, Florida	1,281	74.3	1.7	(70.9–77.7)
Philadelphia, Pennsylvania^†^	1,723	76.5	1.5	(73.7–79.4)
Phoenix-Mesa-Scottsdale, Arizona	1,502	71.3	1.9	(67.6–75.0)
Pittsburgh, Pennsylvania	2,305	73.8	1.3	(71.2–76.4)
Ponce, Puerto Rico	514	79.4	2.4	(74.6–84.1)
Portland-South Portland, Maine	2,583	78.0	1.3	(75.4–80.6)
Portland-Vancouver-Hillsboro, Oregon-Washington	3,118	74.7	1.2	(72.4–77.0)
Port St. Lucie, Florida	1,010	78.3	2.7	(73.0–83.5)
Providence-Warwick, Rhode Island-Massachusetts	8,107	81.1	0.9	(79.3–82.8)
Provo-Orem, Utah	1,770	67.7	1.2	(65.2–70.1)
Raleigh, North Carolina	646	79.1	1.8	(75.5–82.7)
Rapid City, South Dakota	849	72.5	2.2	(68.2–76.8)
Reno, Nevada	1,762	72.5	1.7	(69.3–75.8)
Richmond, Virginia	1,284	77.5	1.7	(74.2–80.8)
Riverside-San Bernardino-Ontario, California	1,349	74.1	1.6	(71.0–77.3)
Rochester, New York	495	81.3	2.5	(76.3–86.2)
Rockingham County-Strafford County, New Hampshire^†^	1,625	79.2	1.6	(76.0–82.5)
Sacramento-Roseville-Arden-Arcade, California	874	73.5	2.0	(69.5–77.5)
St. Louis, Missouri-Illinois	1,997	74.1	1.6	(71.0–77.2)
Salem, Oregon	509	71.8	3.3	(65.2–78.3)
Salina, Kansas	512	68.9	2.7	(63.5–74.2)
Salisbury, Maryland-Delaware	2,014	75.3	2.0	(71.4–79.2)
Salt Lake City, Utah	4,530	71.1	0.8	(69.4–72.7)
San Antonio-New Braunfels, Texas	917	72.2	2.0	(68.3–76.1)
San Francisco-Redwood City-South San Francisco, California^†^	526	77.6	2.5	(72.6–82.6)
San Jose-Sunnyvale-Santa Clara, California	616	76.3	2.4	(71.6–81.0)
San Juan-Carolina-Caguas, Puerto Rico	3,535	77.7	1.0	(75.7–79.7)
Scottsbluff, Nebraska	684	67.9	2.8	(62.4–73.4)
Scranton-Wilkes-Barre-Hazleton, Pennsylvania	551	78.5	2.9	(72.9–84.1)
Seattle-Bellevue-Everett, Washington^†^	3,664	75.8	1.0	(73.9–77.7)
Shreveport-Bossier City, Louisiana	552	74.9	3.4	(68.1–81.6)
Silver Spring-Frederick-Rockville, Maryland^†^	2,379	79.0	1.4	(76.2–81.7)
Sioux City, Iowa-Nebraska-South Dakota	1,035	67.2	2.9	(61.6–72.9)
Sioux Falls, South Dakota	979	72.5	2.0	(68.6–76.4)
Spartanburg, South Carolina	584	68.9	3.0	(63.1–74.7)
Spokane-Spokane Valley, Washington	833	70.1	2.3	(65.5–74.6)
Springfield, Massachusetts	1,523	80.0	2.0	(76.0–83.9)
Tallahassee, Florida	1,813	77.4	2.2	(73.1–81.7)
Tampa-St. Petersburg-Clearwater, Florida	2,165	78.9	1.4	(76.2–81.7)
Toledo, Ohio	966	72.4	2.5	(67.4–77.3)
Topeka, Kansas	2,308	70.5	1.3	(67.9–73.1)
Tulsa, Oklahoma	1,933	71.1	1.5	(68.1–74.1)
Virginia Beach-Norfolk-Newport News, Virginia-North Carolina	1,629	78.4	1.6	(75.2–81.6)
Warren-Troy-Farmington Hills, Michigan^†^	2,213	79.0	1.4	(76.3–81.7)
Washington-Arlington-Alexandria, District of Columbia-Virginia-Maryland-West Virginia^†^	8,806	79.9	0.9	(78.1–81.7)
Wichita, Kansas	4,766	71.3	0.9	(69.5–73.1)
Wilmington, Delaware-Maryland-New Jersey^†^	3,187	78.2	1.2	(75.9–80.5)
Winston-Salem, North Carolina	671	79.3	2.7	(74.0–84.6)
Worcester, Massachusetts-Connecticut	2,692	81.2	1.4	(78.5–83.9)
*Median*		*75.6*		
*Range*		*62.5–83.5*		

**Abbreviations:** CI = confidence interval; MMSA = metropolitan and micropolitan statistical areas; SE = standard error.

* Age adjusted to the 2000 U.S. standard population.

^†^ Metropolitan division.

### Health Risk Behaviors

#### No Leisure-time Physical Activity

Respondents were categorized as having no leisure-time physical activity if they did not participate in any physical activity or exercise (e.g., running, calisthenics, golf, gardening, or walking for exercise) other than their regular job during the preceding month. In 2013, age-adjusted prevalence estimates of no leisure-time physical activity ranged from 17.9% in Colorado to 47.4% in Puerto Rico (median: 25.1%) (Table 23). Among selected MMSA, age-adjusted prevalence estimates ranged from 14.8% in San Francisco-Redwood City-South San Francisco, California, to 48.3% in San Juan-Carolina-Caguas, Puerto Rico (median: 25.1%) (Table 24). In 2014, age-adjusted prevalence estimates ranged from 15.9% in Oregon to 39.7% in Puerto Rico (median: 22.4%) (Table 25). Among selected MMSA, age-adjusted prevalence estimates ranged from 11.7% in Logan, Utah-Idaho, to 42.1% in Aguadilla-Isabela, Puerto Rico (median: 22.6%) (Table 26).

**TABLE 23:** Age-adjusted* prevalence estimates of adults aged ≥18 years who reported no leisure-time physical activity^†^ during the past 30 days, by state/territory — Behavioral Risk Factor Surveillance System, United States, 2013

State/Territory	Sample size	%	SE	95% CI
Alabama	6,164	30.7	0.9	(28.9–32.5)
Alaska	4,275	22.4	0.9	(20.5–24.2)
Arizona	3,910	24.8	1.3	(22.2–27.4)
Arkansas	4,871	33.4	1.0	(31.4–35.4)
California	10,190	21.3	0.6	(20.2–22.5)
Colorado	12,370	17.9	0.5	(17.0–18.8)
Connecticut	7,121	24.2	0.8	(22.6–25.8)
Delaware	4,926	27.0	0.9	(25.2–28.7)
District of Columbia	4,429	19.7	1.0	(17.8–21.7)
Florida	31,527	27.0	0.6	(25.8–28.2)
Georgia	7,381	27.0	0.7	(25.5–28.4)
Hawaii	7,368	21.6	0.8	(20.1–23.1)
Idaho	5,260	23.3	0.9	(21.6–25.1)
Illinois	5,305	24.7	0.9	(23.0–26.4)
Indiana	9,584	30.5	0.7	(29.2–31.8)
Iowa	7,678	27.7	0.7	(26.2–29.1)
Kansas	22,177	26.1	0.4	(25.4–26.9)
Kentucky	10,156	29.4	0.7	(27.9–30.8)
Louisiana	5,026	31.4	1.1	(29.3–33.6)
Maine	7,724	22.0	0.7	(20.6–23.4)
Maryland	12,261	24.7	0.6	(23.5–26.0)
Massachusetts	13,532	23.0	0.6	(21.8–24.2)
Michigan	12,214	23.7	0.6	(22.6–24.9)
Minnesota	13,323	22.9	0.7	(21.6–24.3)
Mississippi	7,009	37.3	0.9	(35.6–39.1)
Missouri	6,793	27.7	0.8	(26.0–29.3)
Montana	9,218	21.5	0.6	(20.3–22.7)
Nebraska	16,158	24.9	0.6	(23.7–26.0)
Nevada	4,799	23.2	1.1	(21.0–25.5)
New Hampshire	6,010	21.7	0.8	(20.1–23.3)
New Jersey	12,049	26.4	0.7	(25.1–27.7)
New Mexico	8,512	23.9	0.7	(22.5–25.2)
New York	8,164	26.2	0.7	(24.9–27.6)
North Carolina	8,401	26.0	0.7	(24.7–27.3)
North Dakota	7,380	27.1	0.8	(25.6–28.6)
Ohio	11,117	27.6	0.7	(26.3–28.9)
Oklahoma	7,831	32.2	0.7	(30.8–33.6)
Oregon	5,407	18.1	0.8	(16.6–19.7)
Pennsylvania	10,564	25.1	0.6	(24.0–26.3)
Rhode Island	5,922	26.2	0.8	(24.6–27.9)
South Carolina	10,067	26.2	0.7	(24.9–27.5)
South Dakota	6,553	23.2	0.9	(21.4–24.9)
Tennessee	5,196	36.6	1.0	(34.6–38.6)
Texas	10,100	30.0	0.7	(28.5–31.4)
Utah	11,937	20.9	0.5	(19.9–21.8)
Vermont	5,986	19.4	0.7	(18.1–20.8)
Virginia	7,767	25.1	0.7	(23.7–26.5)
Washington	10,734	19.7	0.6	(18.5–20.8)
West Virginia	5,769	30.1	0.8	(28.6–31.6)
Wisconsin	5,823	22.9	0.9	(21.2–24.6)
Wyoming	6,055	24.7	0.9	(23.0–26.4)
Guam	1,728	33.3	1.5	(30.3–36.3)
Puerto Rico	5,922	47.4	0.9	(45.7–49.1)
*Median*		*25.1*		
*Range*		*17.9–47.4*		

**Abbreviations:** CI = confidence interval; SE = standard error.

* Age adjusted to the 2000 U.S. standard population.

^†^ Any physical activity or exercise (e.g., running, calisthenics, golf, gardening, or walking for exercise).

**TABLE 24:** Age-adjusted* prevalence of adults aged ≥18 years who reported no leisure-time physical activity^†^ during the past 30 days, by metropolitan and micropolitan statistical area — Behavioral Risk Factor Surveillance System, United States, 2013

MMSA	Sample size	%	SE	95% CI
Aguadilla-Isabela, Puerto Rico	585	42.5	2.7	(37.2–47.7)
Akron, Ohio	642	22.7	2.6	(17.7–27.8)
Albuquerque, New Mexico	1,918	22.4	1.3	(19.9–24.8)
Allentown-Bethlehem-Easton, Pennsylvania-New Jersey	949	25.2	2.1	(21.1–29.2)
Anchorage, Alaska	1,423	21.2	1.4	(18.4–24.0)
Atlanta-Sandy Springs-Roswell, Georgia	3,193	24.1	1.0	(22.1–26.2)
Augusta-Richmond County, Georgia-South Carolina	852	24.5	2.2	(20.1–28.9)
Austin-Round Rock, Texas	879	22.0	2.0	(18.1–25.9)
Baltimore-Columbia-Towson, Maryland	4,474	25.0	1.0	(23.1–26.9)
Baton Rouge, Louisiana	879	33.1	2.5	(28.1–38.0)
Billings, Montana	774	24.3	1.8	(20.7–27.9)
Birmingham-Hoover, Alabama	1,288	31.8	1.9	(28.2–35.5)
Bismarck, North Dakota	984	28.5	2.0	(24.6–32.5)
Boise City, Idaho	1,393	23.0	1.6	(19.8–26.2)
Boston, Massachusetts^§^	3,653	21.0	1.1	(18.9–23.2)
Buffalo-Cheektowaga-Niagara Falls, New York	468	24.5	2.9	(18.8–30.2)
Burlington-South Burlington, Vermont	1,517	18.8	1.3	(16.4–21.3)
Cambridge-Newton-Framingham, Massachusetts^§^	4,393	21.9	1.0	(19.9–23.9)
Camden, New Jersey^§^	1,700	25.3	1.5	(22.3–28.2)
Cedar Rapids, Iowa	612	24.7	2.3	(20.1–29.3)
Charleston, West Virginia	803	32.9	2.3	(28.4–37.3)
Charleston-North Charleston, South Carolina	1,462	21.6	1.5	(18.7–24.5)
Charlotte-Concord-Gastonia, North Carolina-South Carolina	1,852	23.6	1.3	(21.1–26.2)
Chattanooga, Tennessee-Georgia	507	32.5	3.4	(25.9–39.1)
Chicago-Naperville-Elgin, Illinois-Indiana-Wisconsin	3,124	24.0	1.1	(21.8–26.3)
Cincinnati, Ohio-Kentucky-Indiana	2,405	25.8	1.4	(23.1–28.5)
Claremont-Lebanon, New Hampshire-Vermont	1,578	20.2	1.6	(17.1–23.4)
Cleveland-Elyria, Ohio	1,006	25.4	1.9	(21.7–29.0)
Colorado Springs, Colorado	1,254	16.3	1.3	(13.8–18.8)
Columbia, South Carolina	1,367	23.6	1.6	(20.4–26.7)
Columbus, Ohio	1,724	28.7	1.5	(25.9–31.6)
Crestview-Fort Walton Beach-Destin, Florida	997	24.7	2.0	(20.7–28.7)
Dallas-Plano-Irving, Texas^§^	830	29.3	2.0	(25.3–33.3)
Davenport-Moline-Rock Island, Iowa-Illinois	642	26.9	2.7	(21.6–32.2)
Dayton, Ohio	792	27.1	2.3	(22.5–31.7)
Deltona-Daytona Beach-Ormond Beach, Florida	1,027	23.6	2.4	(18.8–28.4)
Denver-Aurora-Lakewood, Colorado	5,168	17.9	0.7	(16.6–19.3)
Des Moines-West Des Moines, Iowa	1,271	25.0	1.8	(21.5–28.5)
Duluth, Minnesota-Wisconsin	665	22.5	2.7	(17.2–27.7)
Durham-Chapel Hill, North Carolina	597	22.2	2.3	(17.6–26.8)
El Paso, Texas	706	31.2	2.3	(26.7–35.7)
Evansville, Indiana-Kentucky	542	31.3	3.0	(25.4–37.2)
Fargo, North Dakota-Minnesota	1,126	25.1	2.1	(21.1–29.2)
Fayetteville-Springdale-Rogers, Arkansas-Missouri	768	28.2	2.3	(23.7–32.8)
Fort Smith, Arkansas-Oklahoma	473	28.5	2.9	(22.7–34.2)
Fort Wayne, Indiana	730	29.4	2.4	(24.7–34.0)
Fort Worth-Arlington, Texas^§^	755	31.1	2.5	(26.2–35.9)
Gainesville, Florida	966	21.5	2.2	(17.2–25.8)
Grand Forks, North Dakota-Minnesota	477	23.1	3.1	(16.9–29.2)
Grand Island, Nebraska	751	33.9	2.8	(28.5–39.3)
Grand Rapids-Wyoming, Michigan	1,296	20.4	1.5	(17.5–23.3)
Greensboro-High Point, North Carolina	629	31.0	2.5	(26.0–35.9)
Greenville-Anderson-Mauldin, South Carolina	1,254	27.8	1.7	(24.4–31.2)
Gulfport-Biloxi-Pascagoula, Mississippi	718	29.8	2.2	(25.5–34.1)
Hagerstown-Martinsburg, Maryland-West Virginia	729	27.5	2.9	(21.9–33.1)
Hartford-West Hartford-East Hartford, Connecticut	2,653	23.0	1.3	(20.5–25.5)
Hilton Head Island-Bluffton-Beaufort, South Carolina	764	19.7	2.7	(14.4–25.0)
Houston-The Woodlands-Sugar Land, Texas	1,268	28.7	2.0	(24.8–32.5)
Huntington-Ashland, West Virginia-Kentucky-Ohio	1,123	30.0	1.7	(26.7–33.3)
Idaho Falls, Idaho	476	24.1	2.6	(19.0–29.3)
Indianapolis-Carmel-Anderson, Indiana	2,352	28.8	1.2	(26.4–31.2)
Jackson, Mississippi	751	35.1	2.4	(30.5–39.8)
Jacksonville, Florida	2,658	25.4	1.4	(22.7–28.0)
Kansas City, Missouri-Kansas	7,063	24.7	1.1	(22.6–26.8)
Kingsport-Bristol-Bristol, Tennessee-Virginia	488	38.2	3.7	(30.9–45.5)
Knoxville, Tennessee	591	33.6	2.7	(28.4–38.8)
Lansing-East Lansing, Michigan	665	20.0	1.9	(16.2–23.8)
Lexington-Fayette, Kentucky	599	25.1	2.4	(20.5–29.8)
Lincoln, Nebraska	1,774	19.8	1.1	(17.6–22.1)
Little Rock-North Little Rock-Conway, Arkansas	1,054	30.6	2.0	(26.7–34.5)
Logan, Utah-Idaho	598	17.9	2.0	(14.0–21.8)
Los Angeles-Long Beach-Anaheim, California	2,651	23.4	1.1	(21.2–25.7)
Louisville-Jefferson County, Kentucky-Indiana	1,986	28.2	1.7	(24.9–31.5)
Lubbock, Texas	503	27.2	2.9	(21.6–32.8)
Manhattan, Kansas	629	21.1	1.9	(17.3–24.9)
Memphis, Tennessee-Mississippi-Arkansas	1,102	36.2	2.2	(31.8–40.6)
Miami-Fort Lauderdale-West Palm Beach, Florida	2,034	29.9	1.7	(26.6–33.1)
Milwaukee-Waukesha-West Allis, Wisconsin	1,098	19.6	1.8	(16.0–23.2)
Minneapolis-St. Paul-Bloomington, Minnesota-Wisconsin	8,458	19.9	0.8	(18.2–21.5)
Minot, North Dakota	606	27.2	2.4	(22.5–31.9)
Montgomery County-Bucks County-Chester County, Pennsylvania^§^	904	20.8	1.7	(17.5–24.2)
Myrtle Beach-Conway-North Myrtle Beach, South Carolina-North Carolina	729	24.2	2.3	(19.6–28.8)
Nashville-Davidson County-Murfreesboro-Franklin, Tennessee	967	31.7	2.0	(27.9–35.6)
Nassau County-Suffolk County, New York^§^	852	27.3	2.0	(23.4–31.3)
Newark, New Jersey-Pennsylvania^§^	3,713	25.3	1.1	(23.2–27.4)
New Orleans-Metairie, Louisiana	1,230	27.6	2.1	(23.4–31.7)
New York-Jersey City-White Plains, New York-New Jersey^§^	8,014	27.2	0.7	(25.8–28.7)
Norfolk, Nebraska	635	24.0	2.0	(20.0–28.0)
North Platte, Nebraska	679	27.3	2.6	(22.3–32.3)
North Port-Sarasota-Bradenton, Florida	983	22.0	2.0	(18.0–26.0)
Oakland-Hayward-Berkeley, California^§^	622	20.6	2.2	(16.3–24.9)
Ogden-Clearfield, Utah	2,312	20.1	1.0	(18.1–22.1)
Oklahoma City, Oklahoma	2,506	31.1	1.2	(28.7–33.5)
Omaha-Council Bluffs, Nebraska-Iowa	2,937	24.7	1.1	(22.6–26.9)
Orlando-Kissimmee-Sanford, Florida	2,118	26.6	1.5	(23.8–29.5)
Panama City, Florida	954	28.0	2.3	(23.5–32.5)
Pensacola-Ferry Pass-Brent, Florida	1,216	26.6	1.7	(23.2–30.0)
Philadelphia, Pennsylvania^§^	1,624	27.3	1.5	(24.3–30.3)
Phoenix-Mesa-Scottsdale, Arizona	1,428	25.3	1.9	(21.7–29.0)
Pittsburgh, Pennsylvania	2,174	24.1	1.2	(21.6–26.5)
Ponce, Puerto Rico	526	46.6	2.9	(40.9–52.2)
Portland-South Portland, Maine	2,534	19.3	1.2	(17.0–21.6)
Portland-Vancouver-Hillsboro, Oregon-Washington	3,007	17.6	1.1	(15.5–19.8)
Port St. Lucie, Florida	932	27.7	2.5	(22.8–32.5)
Providence-Warwick, Rhode Island-Massachusetts	7,505	27.7	1.0	(25.7–29.6)
Provo-Orem, Utah	1,734	19.5	1.1	(17.3–21.8)
Raleigh, North Carolina	628	20.9	1.9	(17.3–24.6)
Rapid City, South Dakota	828	20.3	1.9	(16.6–24.0)
Reno, Nevada	1,697	17.6	1.2	(15.3–20.0)
Richmond, Virginia	1,182	21.5	1.5	(18.6–24.5)
Riverside-San Bernardino-Ontario, California	1,226	23.3	1.6	(20.3–26.4)
Rochester, New York	469	20.8	2.6	(15.6–25.9)
Rockingham County-Strafford County, New Hampshire^§^	1,537	21.8	1.5	(18.8–24.7)
Sacramento-Roseville-Arden-Arcade, California	809	17.0	2.0	(13.1–20.9)
St. Louis, Missouri-Illinois	1,948	25.8	1.5	(22.9–28.7)
Salem, Oregon	463	18.5	2.7	(13.2–23.7)
Salina, Kansas	498	29.7	2.6	(24.5–34.8)
Salisbury, Maryland-Delaware	1,934	27.9	1.7	(24.5–31.3)
Salt Lake City, Utah	4,331	21.4	0.8	(19.8–23.0)
San Antonio-New Braunfels, Texas	879	29.3	2.0	(25.3–33.2)
San Francisco-Redwood City-South San Francisco, California^§^	473	14.8	2.1	(10.7–18.9)
San Jose-Sunnyvale-Santa Clara, California	543	21.2	2.7	(15.9–26.5)
San Juan-Carolina-Caguas, Puerto Rico	3,601	48.3	1.1	(46.1–50.4)
Scottsbluff, Nebraska	682	28.8	2.6	(23.6–33.9)
Scranton-Wilkes-Barre-Hazleton, Pennsylvania	517	24.3	2.6	(19.2–29.5)
Seattle-Bellevue-Everett, Washington^§^	3,610	18.0	0.9	(16.3–19.8)
Shreveport-Bossier City, Louisiana	555	31.3	3.2	(25.0–37.6)
Silver Spring-Frederick-Rockville, Maryland^§^	2,304	20.5	1.5	(17.6–23.4)
Sioux City, Iowa-Nebraska-South Dakota	997	32.7	3.3	(26.3–39.1)
Sioux Falls, South Dakota	951	21.1	1.8	(17.6–24.7)
Spartanburg, South Carolina	562	27.3	2.7	(22.0–32.6)
Spokane-Spokane Valley, Washington	830	17.4	2.0	(13.5–21.3)
Springfield, Massachusetts	1,418	26.5	2.2	(22.2–30.8)
Tallahassee, Florida	1,730	21.2	1.9	(17.5–24.9)
Tampa-St. Petersburg-Clearwater, Florida	2,028	26.5	1.5	(23.6–29.5)
Toledo, Ohio	939	29.5	2.7	(24.2–34.9)
Topeka, Kansas	2,288	26.2	1.2	(23.8–28.7)
Tulsa, Oklahoma	1,913	32.1	1.5	(29.3–35.0)
Virginia Beach-Norfolk-Newport News, Virginia-North Carolina	1,541	26.2	1.6	(23.0–29.4)
Warren-Troy-Farmington Hills, Michigan^§^	2,152	21.3	1.2	(18.8–23.7)
Washington-Arlington-Alexandria, District of Columbia-Virginia-Maryland-West Virginia^§^	8,270	22.6	1.0	(20.7–24.5)
Wichita, Kansas	4,730	26.5	0.8	(24.9–28.2)
Wilmington, Delaware-Maryland-New Jersey^§^	3,090	27.1	1.1	(24.9–29.3)
Winston-Salem, North Carolina	658	25.8	2.3	(21.2–30.4)
Worcester, Massachusetts-Connecticut	2,517	24.9	1.4	(22.1–27.7)
*Median*		*25.1*		
*Range*		*14.8–48.3*		

**Abbreviations:** CI = confidence interval; MMSA = metropolitan and micropolitan statistical areas; SE = standard error.

* Age adjusted to the 2000 U.S. standard population.

^†^ Any physical activity or exercise (e.g., running, calisthenics, golf, gardening, or walking for exercise).

^§^ Metropolitan division.

**TABLE 25:** Age-adjusted* prevalence estimates of adults aged ≥18 years who reported no leisure-time physical activity^†^ during the past 30 days, by state/territory — Behavioral Risk Factor Surveillance System, United States, 2014

State/Territory	Sample size	%	SE	95% CI
Alabama	8,639	26.8	0.7	(25.4–28.2)
Alaska	4,378	19.2	0.8	(17.5–20.8)
Arizona	14,829	20.8	0.6	(19.6–22.0)
Arkansas	5,250	29.4	1.0	(27.5–31.4)
California	7,822	21.5	0.6	(20.3–22.7)
Colorado	13,383	16.4	0.4	(15.5–17.2)
Connecticut	7,943	19.9	0.7	(18.5–21.3)
Delaware	4,293	24.1	1.0	(22.2–26.1)
District of Columbia	4,059	21.2	1.1	(19.1–23.4)
Florida	9,796	22.7	0.7	(21.4–24.0)
Georgia	6,347	23.3	0.7	(21.9–24.8)
Hawaii	7,245	19.0	0.7	(17.7–20.3)
Idaho	5,468	18.4	0.8	(16.8–20.0)
Illinois	5,050	23.6	0.8	(22.0–25.2)
Indiana	11,510	25.4	0.6	(24.2–26.5)
Iowa	8,121	21.5	0.6	(20.3–22.6)
Kansas	13,727	23.1	0.5	(22.2–24.0)
Kentucky	11,189	27.1	0.7	(25.7–28.5)
Louisiana	6,772	29.0	0.7	(27.6–30.4)
Maine	9,118	18.3	0.6	(17.1–19.5)
Maryland	12,556	21.1	0.7	(19.7–22.6)
Massachusetts	15,632	19.6	0.5	(18.5–20.6)
Michigan	8,455	24.8	0.7	(23.5–26.1)
Minnesota	16,395	19.8	0.4	(19.0–20.6)
Mississippi	4,203	31.1	1.0	(29.0–33.1)
Missouri	7,076	24.2	0.8	(22.6–25.8)
Montana	7,493	18.5	0.7	(17.1–19.8)
Nebraska	22,397	20.7	0.5	(19.8–21.6)
Nevada	3,754	22.0	1.1	(19.9–24.1)
New Hampshire	6,174	18.3	0.8	(16.8–19.8)
New Jersey	13,025	22.8	0.6	(21.7–24.0)
New Mexico	8,927	23.0	0.7	(21.6–24.5)
New York	6,845	25.4	0.7	(24.0–26.8)
North Carolina	7,281	22.6	0.6	(21.4–23.8)
North Dakota	7,764	20.7	0.7	(19.2–22.1)
Ohio	10,904	24.0	0.7	(22.6–25.3)
Oklahoma	8,444	27.5	0.6	(26.3–28.8)
Oregon	5,214	15.9	0.7	(14.5–17.3)
Pennsylvania	10,977	22.4	0.6	(21.2–23.6)
Rhode Island	6,436	21.8	0.8	(20.3–23.4)
South Carolina	11,009	24.6	0.6	(23.4–25.8)
South Dakota	7,399	20.7	0.9	(18.9–22.5)
Tennessee	5,139	25.7	0.9	(24.0–27.4)
Texas	15,394	27.5	0.6	(26.3–28.7)
Utah	14,993	17.1	0.4	(16.3–17.8)
Vermont	6,458	18.1	0.6	(17.0–19.3)
Virginia	9,452	23.1	0.6	(21.9–24.3)
Washington	10,074	17.9	0.6	(16.7–19.0)
West Virginia	6,190	27.1	0.7	(25.6–28.5)
Wisconsin	7,036	20.7	0.8	(19.2–22.2)
Wyoming	6,396	21.3	0.9	(19.6–23.1)
Guam	2,518	28.6	1.3	(26.1–31.1)
Puerto Rico	5,988	39.7	0.8	(38.1–41.3)
*Median*		*22.4*		
*Range*		*15.9–39.7*		

**Abbreviations:** CI = confidence interval; SE = standard error.

* Age adjusted to the 2000 U.S. standard population.

^†^ Any physical activity or exercise (e.g., running, calisthenics, golf, gardening, or walking for exercise).

**TABLE 26:** Age-adjusted* prevalence estimates of adults aged ≥18 years who reported no leisure-time physical activity^†^ during the past 30 days, by metropolitan and micropolitan statistical area — Behavioral Risk Factor Surveillance System, United States, 2014

MMSA	Sample size	%	SE	95% CI
Aberdeen, South Dakota	620	25.6	3.1	(19.5–31.7)
Aguadilla-Isabela, Puerto Rico	544	42.1	2.7	(36.9–47.3)
Albuquerque, New Mexico	1,787	21.3	1.4	(18.6–24.1)
Allentown-Bethlehem-Easton, Pennsylvania-New Jersey	1,092	22.5	2.1	(18.5–26.6)
Anchorage, Alaska	1,784	17.7	1.1	(15.5–19.9)
Atlanta-Sandy Springs-Roswell, Georgia	2,776	20.1	1.0	(18.1–22.0)
Augusta-Richmond County, Georgia-South Carolina	888	23.3	2.4	(18.5–28.0)
Austin-Round Rock, Texas	2,254	19.4	1.1	(17.3–21.6)
Baltimore-Columbia-Towson, Maryland	4,612	21.5	1.1	(19.2–23.7)
Baton Rouge, Louisiana	922	27.4	1.8	(23.9–30.8)
Berlin, New Hampshire-Vermont	543	20.5	2.9	(14.9–26.1)
Billings, Montana	808	21.2	2.0	(17.2–25.2)
Birmingham-Hoover, Alabama	1,571	25.2	1.5	(22.3–28.1)
Bismarck, North Dakota	1,037	20.0	1.9	(16.3–23.8)
Boise City, Idaho	1,349	18.2	1.6	(15.1–21.2)
Boston, Massachusetts^§^	4,545	18.7	1.0	(16.7–20.6)
Burlington-South Burlington, Vermont	1,978	16.3	1.0	(14.3–18.3)
Cambridge-Newton-Framingham, Massachusetts^§^	5,171	18.4	0.9	(16.6–20.1)
Camden, New Jersey^§^	1,717	24.8	1.7	(21.4–28.2)
Cedar Rapids, Iowa	640	22.6	2.2	(18.4–26.9)
Charleston, West Virginia	876	26.0	1.9	(22.3–29.7)
Charleston-North Charleston, South Carolina	1,402	20.7	1.4	(18.0–23.5)
Charlotte-Concord-Gastonia, North Carolina-South Carolina	2,156	20.7	1.1	(18.5–22.9)
Chicago-Naperville-Elgin, Illinois-Indiana-Wisconsin	4,119	22.9	1.0	(21.0–24.8)
Cincinnati, Ohio-Kentucky-Indiana	2,043	22.8	1.6	(19.7–25.9)
Claremont-Lebanon, New Hampshire-Vermont	1,685	20.9	1.9	(17.2–24.5)
Cleveland-Elyria, Ohio	962	23.7	2.2	(19.4–27.9)
College Station-Bryan, Texas	573	31.9	3.8	(24.5–39.3)
Colorado Springs, Colorado	1,299	19.1	1.5	(16.1–22.0)
Columbia, South Carolina	1,206	21.3	1.5	(18.4–24.3)
Columbus, Ohio	1,652	22.1	1.6	(19.1–25.2)
Corpus Christi, Texas	623	25.9	2.9	(20.3–31.5)
Dallas-Plano-Irving, Texas^§^	1,297	26.1	1.9	(22.4–29.8)
Dayton, Ohio	564	22.6	2.5	(17.7–27.5)
Denver-Aurora-Lakewood, Colorado	5,797	15.7	0.6	(14.5–16.9)
Des Moines-West Des Moines, Iowa	1,358	18.5	1.4	(15.7–21.2)
Duluth, Minnesota-Wisconsin	947	19.3	1.8	(15.8–22.7)
El Paso, Texas	722	30.0	2.4	(25.4–34.7)
Evansville, Indiana-Kentucky	656	20.9	2.2	(16.7–25.2)
Fargo, North Dakota-Minnesota	1,149	16.8	1.4	(14.1–19.6)
Fayetteville-Springdale-Rogers, Arkansas-Missouri	815	27.8	2.6	(22.8–32.8)
Fort Wayne, Indiana	863	24.2	2.2	(19.8–28.6)
Fort Worth-Arlington, Texas^§^	759	22.3	2.2	(18.0–26.7)
Grand Island, Nebraska	1,059	27.1	2.2	(22.7–31.5)
Grand Rapids-Wyoming, Michigan	896	23.6	2.1	(19.6–27.7)
Greensboro-High Point, North Carolina	521	23.2	2.2	(18.8–27.6)
Greenville-Anderson-Mauldin, South Carolina	1,491	24.6	1.6	(21.5–27.7)
Hagerstown-Martinsburg, Maryland-West Virginia	782	30.1	3.3	(23.7–36.5)
Hartford-West Hartford-East Hartford, Connecticut	2,640	19.4	1.1	(17.2–21.5)
Hilton Head Island-Bluffton-Beaufort, South Carolina	554	16.7	2.2	(12.4–21.1)
Houston-The Woodlands-Sugar Land, Texas	2,159	26.5	1.5	(23.5–29.5)
Huntington-Ashland, West Virginia-Kentucky-Ohio	1,245	26.4	1.8	(22.8–30.0)
Idaho Falls, Idaho	518	20.9	2.4	(16.1–25.7)
Indianapolis-Carmel-Anderson, Indiana	3,598	25.3	1.1	(23.2–27.5)
Jacksonville, Florida	672	22.5	2.3	(18.0–26.9)
Kansas City, Missouri-Kansas	4,878	21.7	1.0	(19.6–23.7)
Kingsport-Bristol-Bristol, Tennessee-Virginia	507	26.3	2.7	(21.0–31.5)
Knoxville, Tennessee	564	23.9	2.6	(18.9–29.0)
Lafayette, Louisiana	562	32.5	2.6	(27.5–37.5)
Lexington-Fayette, Kentucky	623	27.3	2.4	(22.6–31.9)
Lincoln, Nebraska	2,008	18.3	1.1	(16.1–20.6)
Little Rock-North Little Rock-Conway, Arkansas	1,179	27.1	1.8	(23.5–30.7)
Logan, Utah-Idaho	622	11.7	1.4	(9.0–14.5)
Los Angeles-Long Beach-Anaheim, California	2,037	20.9	1.1	(18.7–23.0)
Louisville-Jefferson County, Kentucky-Indiana	2,460	24.4	1.6	(21.2–27.5)
Madison, Wisconsin	551	17.9	2.1	(13.8–22.0)
Memphis, Tennessee-Mississippi-Arkansas	882	21.2	1.8	(17.7–24.7)
Miami-Fort Lauderdale-West Palm Beach, Florida	2,220	22.7	1.2	(20.3–25.2)
Milwaukee-Waukesha-West Allis, Wisconsin	1,355	21.8	1.8	(18.3–25.4)
Minneapolis-St. Paul-Bloomington, Minnesota-Wisconsin	8,756	18.3	0.5	(17.3–19.4)
Minot, North Dakota	594	23.8	2.7	(18.6–29.0)
Montgomery, Alabama	513	23.3	2.4	(18.6–28.0)
Montgomery County-Bucks County-Chester County, Pennsylvania^§^	805	15.9	1.7	(12.6–19.1)
Myrtle Beach-Conway-North Myrtle Beach, South Carolina-North Carolina	999	26.3	2.1	(22.2–30.4)
Nashville-Davidson County-Murfreesboro-Franklin, Tennessee	803	21.9	2.0	(17.9–25.9)
Nassau County-Suffolk County, New York^§^	770	25.9	2.2	(21.5–30.3)
Newark, New Jersey-Pennsylvania^§^	4,144	22.6	1.1	(20.5–24.7)
New Orleans-Metairie, Louisiana	1,913	25.7	1.3	(23.1–28.3)
New York-Jersey City-White Plains, New York-New Jersey^§^	7,531	26.6	0.8	(25.1–28.2)
Norfolk, Nebraska	996	22.9	1.7	(19.5–26.3)
North Platte, Nebraska	967	22.8	1.7	(19.4–26.1)
North Port-Sarasota-Bradenton, Florida	513	22.9	3.7	(15.6–30.2)
Oakland-Hayward-Berkeley, California^§^	638	21.3	2.2	(16.9–25.7)
Ogden-Clearfield, Utah	2,922	16.2	0.8	(14.7–17.8)
Oklahoma City, Oklahoma	2,436	25.1	1.1	(23.0–27.3)
Omaha-Council Bluffs, Nebraska-Iowa	4,869	19.1	0.8	(17.5–20.7)
Orlando-Kissimmee-Sanford, Florida	956	23.3	2.0	(19.4–27.2)
Philadelphia, Pennsylvania^§^	1,520	23.9	1.6	(20.8–27.1)
Phoenix-Mesa-Scottsdale, Arizona	9,377	21.0	0.7	(19.6–22.4)
Pittsburgh, Pennsylvania	2,410	22.5	1.2	(20.2–24.8)
Ponce, Puerto Rico	530	37.1	2.8	(31.5–42.6)
Portland-South Portland, Maine	2,755	15.2	1.0	(13.2–17.2)
Portland-Vancouver-Hillsboro, Oregon-Washington	2,839	15.5	1.0	(13.5–17.5)
Providence-Warwick, Rhode Island-Massachusetts	8,100	22.4	0.9	(20.7–24.1)
Provo-Orem, Utah	2,147	15.1	0.9	(13.3–16.8)
Raleigh, North Carolina	720	16.1	1.6	(12.9–19.2)
Rapid City, South Dakota	1,420	20.3	1.8	(16.8–23.8)
Reno, Nevada	1,200	15.6	1.4	(12.9–18.3)
Richmond, Virginia	1,465	22.3	1.5	(19.4–25.2)
Riverside-San Bernardino-Ontario, California	840	21.5	1.7	(18.1–24.8)
Roanoke, Virginia	532	22.5	2.4	(17.7–27.2)
Rochester, Minnesota	699	22.6	2.2	(18.3–26.9)
Rockingham County-Strafford County, New Hampshire^§^	1,439	18.7	1.5	(15.8–21.6)
Sacramento-Roseville-Arden-Arcade, California	583	19.9	2.2	(15.6–24.3)
St. Cloud, Minnesota	561	22.7	2.3	(18.1–27.3)
St. Louis, Missouri-Illinois	1,926	24.0	1.6	(20.9–27.1)
Salisbury, Maryland-Delaware	1,956	29.7	2.3	(25.1–34.2)
Salt Lake City, Utah	5,418	18.2	0.6	(17.0–19.5)
San Antonio-New Braunfels, Texas	2,276	27.8	1.3	(25.2–30.4)
San Juan-Carolina-Caguas, Puerto Rico	3,749	39.9	1.0	(37.9–41.9)
Scottsbluff, Nebraska	909	26.1	2.1	(22.0–30.2)
Seattle-Bellevue-Everett, Washington^§^	3,690	15.8	0.9	(14.0–17.6)
Shreveport-Bossier City, Louisiana	552	27.4	2.3	(22.9–32.0)
Silver Spring-Frederick-Rockville, Maryland^§^	2,378	17.5	1.6	(14.3–20.6)
Sioux City, Iowa-Nebraska-South Dakota	1,143	26.6	2.6	(21.6–31.6)
Sioux Falls, South Dakota	1,348	20.5	1.8	(16.9–24.1)
Spartanburg, South Carolina	562	28.0	3.1	(21.9–34.2)
Spokane-Spokane Valley, Washington	725	16.6	2.0	(12.7–20.5)
Springfield, Massachusetts	1,106	24.1	2.1	(20.0–28.2)
Tampa-St. Petersburg-Clearwater, Florida	1,576	20.7	1.6	(17.6–23.7)
Toledo, Ohio	649	27.1	2.7	(21.9–32.3)
Topeka, Kansas	1,441	22.2	1.4	(19.4–24.9)
Tulsa, Oklahoma	2,031	24.0	1.2	(21.7–26.4)
Tuscaloosa, Alabama	720	29.7	2.6	(24.7–34.7)
Virginia Beach-Norfolk-Newport News, Virginia-North Carolina	1,879	23.3	1.4	(20.6–26.0)
Warren-Troy-Farmington Hills, Michigan^§^	2,116	21.8	1.2	(19.3–24.2)
Washington-Arlington-Alexandria, District of Columbia-Virginia-Maryland-West Virginia^§^	8,280	19.7	0.8	(18.1–21.3)
Wichita, Kansas	2,735	23.2	1.0	(21.3–25.1)
Wichita Falls, Texas	545	26.7	3.8	(19.2–34.1)
Wilmington, Delaware-Maryland-New Jersey^§^	2,765	23.4	1.2	(21.0–25.9)
Worcester, Massachusetts-Connecticut	2,459	20.6	1.3	(18.1–23.1)
Youngstown-Warren-Boardman, Ohio-Pennsylvania	522	23.0	2.8	(17.6–28.5)
*Median*		*22.6*		
*Range*		*11.7–42.1*		

**Abbreviations:** CI = confidence interval; MMSA = metropolitan and micropolitan statistical areas; SE = standard error.

* Age adjusted to the 2000 U.S. standard population.

^†^ Any physical activity or exercise (e.g., running, calisthenics, golf, gardening, or walking for exercise).

^§^ Metropolitan division.

#### Inadequate Sleep

Respondents were asked to determine the average number of hours of sleep they usually get during a 24-hour period. Those having <7 hours of sleep were classified as having inadequate sleep and those having >7 hours of sleep as having adequate sleep. In 2013, age-adjusted prevalence estimates of inadequate sleep ranged from 27.6% in South Dakota to 49.2% in Guam (median: 35.3%) (Table 27). Among selected MMSA, age-adjusted prevalence estimates ranged from 26.5% in Sioux Falls, South Dakota, to 44.4% in Augusta-Richmond County, Georgia-South Carolina (median: 34.9%) (Table 28). In 2014, age-adjusted prevalence estimates ranged from 28.4% in South Dakota to 48.6% in Guam (median: 34.7%) (Table 29). Among selected MMSA, age-adjusted prevalence estimates ranged from 25.4% in Fargo, North Dakota-Minnesota, to 45.3% in Kingsport-Bristol-Bristol, Tennessee-Virginia (median: 34.8%) (Table 30).

**TABLE 27:** Age-adjusted* prevalence estimates of adults aged ≥18 years who reported having inadequate sleep^†^ during a 24-hour period, by state/territory — Behavioral Risk Factor Surveillance System, United States, 2013

State/Territory	Sample size	%	SE	95% CI
Alabama	6,319	37.6	1.0	(35.7–39.5)
Alaska	4,545	34.9	1.1	(32.8–36.9)
Arizona	4,195	35.2	1.4	(32.4–38.0)
Arkansas	5,166	36.6	1.1	(34.5–38.7)
California	11,461	33.5	0.6	(32.3–34.8)
Colorado	13,457	28.9	0.5	(27.9–30.0)
Connecticut	7,614	34.9	0.9	(33.2–36.5)
Delaware	5,133	36.9	1.0	(34.9–38.8)
District of Columbia	4,828	35.6	1.1	(33.4–37.7)
Florida	33,620	37.6	0.7	(36.3–38.9)
Georgia	7,981	38.2	0.8	(36.6–39.7)
Hawaii	7,816	41.2	0.8	(39.5–42.8)
Idaho	5,563	31.7	1.0	(29.8–33.6)
Illinois	5,601	33.9	1.0	(32.1–35.8)
Indiana	10,203	36.4	0.7	(35.0–37.7)
Iowa	8,075	31.4	0.8	(29.9–32.9)
Kansas	22,995	29.4	0.4	(28.7–30.2)
Kentucky	10,838	41.6	0.8	(40.0–43.1)
Louisiana	5,106	37.0	1.2	(34.7–39.3)
Maine	8,003	32.6	0.8	(31.0–34.1)
Maryland	12,751	38.9	0.7	(37.4–40.3)
Massachusetts	14,857	34.6	0.6	(33.3–35.9)
Michigan	12,628	38.4	0.7	(37.1–39.6)
Minnesota	14,176	31.9	0.7	(30.5–33.4)
Mississippi	7,180	36.2	0.9	(34.5–38.0)
Missouri	6,942	33.2	0.9	(31.4–35.0)
Montana	9,588	31.4	0.7	(30.0–32.8)
Nebraska	16,972	32.1	0.6	(30.9–33.4)
Nevada	5,035	35.3	1.3	(32.8–37.9)
New Hampshire	6,398	32.7	0.9	(30.9–34.4)
New Jersey	13,121	39.1	0.7	(37.8–40.4)
New Mexico	9,199	32.5	0.8	(31.0–34.0)
New York	8,832	40.1	0.7	(38.6–41.5)
North Carolina	8,644	35.8	0.7	(34.3–37.2)
North Dakota	7,717	29.6	0.8	(28.0–31.1)
Ohio	11,758	40.2	0.7	(38.8–41.6)
Oklahoma	8,059	37.6	0.8	(36.1–39.1)
Oregon	5,886	31.2	0.9	(29.4–32.9)
Pennsylvania	11,291	37.8	0.7	(36.5–39.1)
Rhode Island	6,424	38.0	0.9	(36.3–39.8)
South Carolina	10,408	38.0	0.7	(36.5–39.4)
South Dakota	6,840	27.6	1.0	(25.7–29.5)
Tennessee	5,659	38.5	1.0	(36.5–40.4)
Texas	10,687	32.5	0.7	(31.1–33.9)
Utah	12,638	32.6	0.5	(31.6–33.7)
Vermont	6,322	31.5	0.9	(29.8–33.1)
Virginia	8,321	37.4	0.8	(35.9–38.9)
Washington	11,025	32.9	0.6	(31.7–34.2)
West Virginia	5,810	40.5	0.9	(38.9–42.2)
Wisconsin	6,558	31.7	1.0	(29.8–33.5)
Wyoming	6,360	29.3	0.9	(27.5–31.0)
Guam	1,879	49.2	1.5	(46.2–52.1)
Puerto Rico	5,917	37.0	0.8	(35.4–38.7)
*Median*		*35.3*		
*Range*		*27.6–49.2*		

**Abbreviations:** CI = confidence interval; SE = standard error.

* Age adjusted to the 2000 U.S. standard population.

^†^ Inadequate sleep is defined as <7 hours of sleep during a 24-hour period on average.

**TABLE 28:** Age-adjusted* prevalence estimates of adults aged ≥18 years who reported having inadequate sleep^†^ during a 24-hour period, by metropolitan and micropolitan statistical area — Behavioral Risk Factor Surveillance System, United States, 2013

MMSA	Sample size	%	SE	95% CI
Aguadilla-Isabela, Puerto Rico	583	34.3	2.7	(29.1–39.6)
Akron, Ohio	686	42.9	3.2	(36.6–49.1)
Albuquerque, New Mexico	2,063	32.9	1.4	(30.2–35.6)
Allentown-Bethlehem-Easton, Pennsylvania-New Jersey	1,016	39.2	2.5	(34.4–44.1)
Anchorage, Alaska	1,511	36.3	1.6	(33.1–39.5)
Atlanta-Sandy Springs-Roswell, Georgia	3,458	37.0	1.1	(34.8–39.2)
Augusta-Richmond County, Georgia-South Carolina	897	44.4	2.7	(39.1–49.8)
Austin-Round Rock, Texas	931	33.4	2.1	(29.2–37.5)
Baltimore-Columbia-Towson, Maryland	4,685	39.1	1.1	(36.9–41.3)
Baton Rouge, Louisiana	914	34.2	2.4	(29.4–38.9)
Billings, Montana	810	34.2	1.9	(30.4–38.0)
Birmingham-Hoover, Alabama	1,324	35.3	1.8	(31.7–38.9)
Bismarck, North Dakota	1,030	29.9	1.9	(26.1–33.7)
Boise City, Idaho	1,476	31.0	1.7	(27.6–34.3)
Boston, Massachusetts^§^	4,012	37.3	1.2	(34.8–39.7)
Buffalo-Cheektowaga-Niagara Falls, New York	497	38.6	3.1	(32.5–44.8)
Burlington-South Burlington, Vermont	1,620	31.6	1.5	(28.6–34.6)
Cambridge-Newton-Framingham, Massachusetts^§^	4,842	32.1	1.1	(29.9–34.2)
Camden, New Jersey^§^	1,831	40.7	1.6	(37.4–43.9)
Cedar Rapids, Iowa	641	29.9	2.6	(24.9–35.0)
Charleston, West Virginia	806	43.7	2.3	(39.1–48.2)
Charleston-North Charleston, South Carolina	1,520	34.9	1.7	(31.5–38.3)
Charlotte-Concord-Gastonia, North Carolina-South Carolina	1,915	34.9	1.5	(32.0–37.9)
Chattanooga, Tennessee-Georgia	563	31.3	3.0	(25.5–37.2)
Chicago-Naperville-Elgin, Illinois-Indiana-Wisconsin	3,333	34.3	1.2	(31.9–36.7)
Cincinnati, Ohio-Kentucky-Indiana	2,564	38.7	1.5	(35.8–41.7)
Claremont-Lebanon, New Hampshire-Vermont	1,667	29.3	1.8	(25.9–32.8)
Cleveland-Elyria, Ohio	1,088	42.8	2.2	(38.6–47.1)
Colorado Springs, Colorado	1,363	32.0	1.7	(28.7–35.2)
Columbia, South Carolina	1,411	38.5	1.8	(34.9–42.1)
Columbus, Ohio	1,836	40.4	1.5	(37.5–43.3)
Crestview-Fort Walton Beach-Destin, Florida	1,074	36.7	2.3	(32.3–41.2)
Dallas-Plano-Irving, Texas^§^	893	33.0	2.0	(29.1–36.8)
Davenport-Moline-Rock Island, Iowa-Illinois	669	33.0	2.8	(27.5–38.4)
Dayton, Ohio	833	36.0	2.5	(31.1–40.9)
Deltona-Daytona Beach-Ormond Beach, Florida	1,094	39.1	2.8	(33.7–44.6)
Denver-Aurora-Lakewood, Colorado	5,642	29.9	0.8	(28.4–31.4)
Des Moines-West Des Moines, Iowa	1,334	27.5	1.7	(24.1–30.8)
Duluth, Minnesota-Wisconsin	697	37.5	3.4	(30.8–44.2)
Durham-Chapel Hill, North Carolina	606	29.5	2.4	(24.7–34.2)
El Paso, Texas	740	37.1	2.4	(32.4–41.8)
Evansville, Indiana-Kentucky	570	40.7	3.1	(34.6–46.9)
Fargo, North Dakota-Minnesota	1,171	27.0	1.9	(23.4–30.6)
Fayetteville-Springdale-Rogers, Arkansas-Missouri	812	32.5	2.5	(27.7–37.4)
Fort Smith, Arkansas-Oklahoma	490	34.3	3.3	(27.9–40.8)
Fort Wayne, Indiana	776	32.7	2.3	(28.2–37.2)
Fort Worth-Arlington, Texas^§^	791	36.9	2.5	(32.0–41.8)
Gainesville, Florida	1,011	37.2	2.7	(31.9–42.4)
Grand Forks, North Dakota-Minnesota	499	32.0	3.5	(25.2–38.9)
Grand Island, Nebraska	785	32.1	2.6	(27.1–37.2)
Grand Rapids-Wyoming, Michigan	1,338	35.7	1.8	(32.2–39.3)
Greensboro-High Point, North Carolina	645	37.5	2.6	(32.4–42.6)
Greenville-Anderson-Mauldin, South Carolina	1,310	37.7	1.9	(34.1–41.4)
Gulfport-Biloxi-Pascagoula, Mississippi	750	44.1	2.5	(39.2–49.0)
Hagerstown-Martinsburg, Maryland-West Virginia	753	39.5	3.0	(33.7–45.3)
Hartford-West Hartford-East Hartford, Connecticut	2,804	34.6	1.4	(31.9–37.4)
Hilton Head Island-Bluffton-Beaufort, South Carolina	807	36.2	3.1	(30.1–42.4)
Houston-The Woodlands-Sugar Land, Texas	1,365	31.5	1.8	(28.0–34.9)
Huntington-Ashland, West Virginia-Kentucky-Ohio	1,170	41.7	1.9	(38.0–45.4)
Idaho Falls, Idaho	502	31.6	2.9	(25.9–37.2)
Indianapolis-Carmel-Anderson, Indiana	2,515	34.8	1.3	(32.3–37.3)
Jackson, Mississippi	776	32.3	2.3	(27.8–36.9)
Jacksonville, Florida	2,845	38.8	1.5	(36.0–41.7)
Kansas City, Missouri-Kansas	7,324	30.2	1.1	(28.0–32.5)
Kingsport-Bristol-Bristol, Tennessee-Virginia	526	33.6	3.4	(26.9–40.2)
Knoxville, Tennessee	640	37.2	2.7	(31.8–42.6)
Lansing-East Lansing, Michigan	685	34.6	2.4	(29.9–39.2)
Lexington-Fayette, Kentucky	632	35.5	2.4	(30.8–40.1)
Lincoln, Nebraska	1,864	30.9	1.3	(28.4–33.5)
Little Rock-North Little Rock-Conway, Arkansas	1,134	37.8	2.1	(33.7–41.9)
Logan, Utah-Idaho	634	29.5	2.3	(25.0–33.9)
Los Angeles-Long Beach-Anaheim, California	3,022	34.9	1.2	(32.5–37.3)
Louisville/Jefferson County, Kentucky-Indiana	2,110	40.9	1.7	(37.6–44.3)
Lubbock, Texas	522	38.2	3.2	(31.9–44.6)
Manhattan, Kansas	655	26.8	2.1	(22.8–30.9)
Memphis, Tennessee-Mississippi-Arkansas	1,180	36.0	2.2	(31.7–40.2)
Miami-Fort Lauderdale-West Palm Beach, Florida	2,182	39.0	1.7	(35.6–42.3)
Milwaukee-Waukesha-West Allis, Wisconsin	1,262	32.6	2.2	(28.3–36.8)
Minneapolis-St. Paul-Bloomington, Minnesota-Wisconsin	9,036	30.9	1.0	(29.1–32.8)
Minot, North Dakota	647	32.2	2.4	(27.5–36.9)
Montgomery County-Bucks County-Chester County, Pennsylvania^§^	964	36.4	2.0	(32.4–40.4)
Myrtle Beach-Conway-North Myrtle Beach, South Carolina-North Carolina	758	36.1	2.6	(31.0–41.3)
Nashville-Davidson County-Murfreesboro-Franklin, Tennessee	1,034	34.2	1.9	(30.4–38.0)
Nassau County-Suffolk County, New York^§^	932	40.1	2.1	(36.0–44.2)
Newark, New Jersey-Pennsylvania^§^	4,048	37.7	1.1	(35.5–40.0)
New Orleans-Metairie, Louisiana	1,250	38.3	2.4	(33.7–43.0)
New York-Jersey City-White Plains, New York-New Jersey^§^	8,768	40.9	0.8	(39.3–42.5)
Norfolk, Nebraska	664	33.7	2.2	(29.3–38.1)
North Platte, Nebraska	708	33.8	2.7	(28.6–39.1)
North Port-Sarasota-Bradenton, Florida	1,070	32.9	2.3	(28.5–37.4)
Oakland-Hayward-Berkeley, California^§^	699	35.2	2.4	(30.6–39.8)
Ogden-Clearfield, Utah	2,441	33.6	1.2	(31.3–35.9)
Oklahoma City, Oklahoma	2,599	38.3	1.3	(35.8–40.8)
Omaha-Council Bluffs, Nebraska-Iowa	3,108	34.5	1.2	(32.2–36.9)
Orlando-Kissimmee-Sanford, Florida	2,237	37.1	1.6	(34.0–40.2)
Panama City, Florida	1,011	41.3	2.5	(36.4–46.2)
Pensacola-Ferry Pass-Brent, Florida	1,294	39.1	1.8	(35.4–42.7)
Philadelphia, Pennsylvania^§^	1,752	42.5	1.7	(39.3–45.8)
Phoenix-Mesa-Scottsdale, Arizona	1,537	36.5	1.9	(32.6–40.3)
Pittsburgh, Pennsylvania	2,337	37.9	1.4	(35.1–40.7)
Ponce, Puerto Rico	523	30.7	2.5	(25.8–35.5)
Portland-South Portland, Maine	2,611	30.1	1.3	(27.5–32.6)
Portland-Vancouver-Hillsboro, Oregon-Washington	3,202	28.3	1.2	(26.1–30.6)
Port St. Lucie, Florida	1,014	35.4	2.7	(30.1–40.7)
Providence-Warwick, Rhode Island-Massachusetts	8,181	37.5	1.0	(35.5–39.5)
Provo-Orem, Utah	1,841	31.5	1.3	(29.0–33.9)
Raleigh, North Carolina	665	29.5	2.0	(25.5–33.5)
Rapid City, South Dakota	865	30.8	2.2	(26.5–35.0)
Reno, Nevada	1,805	32.8	1.5	(29.8–35.8)
Richmond, Virginia	1,298	38.5	1.8	(35.0–42.1)
Riverside-San Bernardino-Ontario, California	1,362	36.4	1.7	(33.2–39.7)
Rochester, New York	504	34.5	3.0	(28.5–40.4)
Rockingham County-Strafford County, New Hampshire^§^	1,645	32.5	1.7	(29.2–35.8)
Sacramento-Roseville-Arden-Arcade, California	888	30.1	2.1	(26.1–34.2)
St. Louis, Missouri-Illinois	2,024	34.8	1.6	(31.7–38.0)
Salem, Oregon	526	34.0	3.2	(27.7–40.4)
Salina, Kansas	521	32.0	2.8	(26.5–37.5)
Salisbury, Maryland-Delaware	2,032	34.9	2.0	(31.1–38.8)
Salt Lake City, Utah	4,625	33.6	0.9	(31.9–35.4)
San Antonio-New Braunfels, Texas	923	31.3	2.0	(27.3–35.3)
San Francisco-Redwood City-South San Francisco, California^§^	535	32.4	2.9	(26.7–38.2)
San Jose-Sunnyvale-Santa Clara, California	624	28.0	2.5	(23.2–32.9)
San Juan-Carolina-Caguas, Puerto Rico	3,603	39.2	1.1	(37.1–41.3)
Scottsbluff, Nebraska	705	35.6	2.7	(30.3–41.0)
Scranton-Wilkes-Barre-Hazleton, Pennsylvania	558	43.7	3.1	(37.5–49.8)
Seattle-Bellevue-Everett, Washington^§^	3,739	33.8	1.0	(31.8–35.9)
Shreveport-Bossier City, Louisiana	560	27.8	2.7	(22.4–33.2)
Silver Spring-Frederick-Rockville, Maryland^§^	2,387	36.3	1.6	(33.2–39.3)
Sioux City, Iowa-Nebraska-South Dakota	1,042	27.1	2.7	(21.8–32.5)
Sioux Falls, South Dakota	1,005	26.5	2.0	(22.5–30.5)
Spartanburg, South Carolina	581	38.7	3.1	(32.6–44.8)
Spokane-Spokane Valley, Washington	851	27.6	2.2	(23.3–32.0)
Springfield, Massachusetts	1,539	36.3	2.2	(32.1–40.5)
Tallahassee, Florida	1,833	35.7	2.3	(31.3–40.2)
Tampa-St. Petersburg-Clearwater, Florida	2,186	36.2	1.6	(33.2–39.3)
Toledo, Ohio	985	37.1	2.5	(32.2–42.0)
Topeka, Kansas	2,371	30.7	1.3	(28.1–33.2)
Tulsa, Oklahoma	1,955	36.4	1.6	(33.3–39.5)
Virginia Beach-Norfolk-Newport News, Virginia-North Carolina	1,656	38.0	1.7	(34.6–41.3)
Warren-Troy-Farmington Hills, Michigan^§^	2,244	37.0	1.4	(34.1–39.8)
Washington-Arlington-Alexandria, District of Columbia-Virginia-Maryland-West Virginia^§^	8,861	35.9	1.0	(33.9–37.8)
Wichita, Kansas	4,887	30.9	0.9	(29.1–32.6)
Wilmington, Delaware-Maryland-New Jersey^§^	3,223	39.2	1.2	(36.8–41.7)
Winston-Salem, North Carolina	681	36.5	2.8	(31.0–41.9)
Worcester, Massachusetts-Connecticut	2,729	35.1	1.5	(32.1–38.0)
*Median*		*34.9*		
*Range*		*26.5–44.4*		

**Abbreviations:** CI = confidence interval; MMSA = metropolitan and micropolitan statistical areas; SE = standard error.

* Age adjusted to the 2000 U.S. standard population.

^†^ Inadequate sleep is defined as <7 hours of sleep during a 24-hour period on average.

^§ ^Metropolitan division.

**TABLE 29:** Age-adjusted* prevalence estimates of adults aged ≥18 years who reported receiving inadequate^†^ sleep during a 24-hour period, by state/territory — Behavioral Risk Factor Surveillance System, United States, 2014

State/Territory	Sample size	%	SE	95% CI
Alabama	8,405	38.6	0.8	(37.0–40.2)
Alaska	4,345	34.9	1.1	(32.8–37.0)
Arizona	14,685	33.0	0.7	(31.6–34.4)
Arkansas	5,144	37.1	1.2	(34.8–39.4)
California	8,788	33.5	0.7	(32.2–34.8)
Colorado	13,218	28.5	0.5	(27.4–29.5)
Connecticut	7,862	34.7	0.9	(33.0–36.4)
Delaware	4,212	37.7	1.2	(35.3–40.0)
District of Columbia	3,959	32.0	1.2	(29.6–34.4)
Florida	9,703	35.7	0.8	(34.2–37.2)
Georgia	6,243	38.6	0.9	(36.9–40.3)
Hawaii	7,220	43.6	0.9	(41.9–45.3)
Idaho	5,426	30.6	1.0	(28.7–32.5)
Illinois	5,042	34.3	0.9	(32.5–36.2)
Indiana	11,384	38.1	0.7	(36.8–39.4)
Iowa	8,046	30.8	0.7	(29.3–32.2)
Kansas	13,547	30.7	0.5	(29.7–31.8)
Kentucky	11,006	39.4	0.8	(37.8–41.1)
Louisiana	6,665	36.0	0.8	(34.4–37.5)
Maine	9,050	32.6	0.8	(31.1–34.2)
Maryland	12,361	38.7	0.9	(37.0–40.4)
Massachusetts	15,399	34.0	0.7	(32.7–35.3)
Michigan	8,381	38.2	0.8	(36.7–39.7)
Minnesota	16,236	29.2	0.5	(28.3–30.1)
Mississippi	4,069	36.7	1.1	(34.5–38.9)
Missouri	6,953	33.6	0.9	(31.7–35.4)
Montana	7,377	30.5	0.9	(28.7–32.2)
Nebraska	22,172	30.3	0.6	(29.2–31.4)
Nevada	3,693	36.2	1.3	(33.7–38.7)
New Hampshire	6,109	32.3	1.0	(30.4–34.2)
New Jersey	12,838	37.0	0.7	(35.6–38.3)
New Mexico	8,835	31.9	0.8	(30.3–33.5)
New York	6,751	38.2	0.8	(36.7–39.8)
North Carolina	7,135	32.4	0.7	(31.0–33.8)
North Dakota	7,712	31.4	0.9	(29.6–33.2)
Ohio	10,805	37.6	0.8	(36.0–39.2)
Oklahoma	8,291	35.5	0.7	(34.1–37.0)
Oregon	5,154	31.5	0.9	(29.6–33.3)
Pennsylvania	10,875	37.3	0.7	(35.9–38.8)
Rhode Island	6,340	36.5	1.0	(34.6–38.4)
South Carolina	10,747	38.2	0.7	(36.8–39.6)
South Dakota	7,338	28.4	1.0	(26.4–30.4)
Tennessee	5,026	37.1	1.1	(34.9–39.2)
Texas	15,121	33.0	0.7	(31.7–34.3)
Utah	14,875	30.7	0.5	(29.8–31.6)
Vermont	6,423	30.6	0.8	(29.1–32.1)
Virginia	9,333	35.9	0.7	(34.5–37.3)
Washington	10,005	31.7	0.7	(30.3–33.0)
West Virginia	6,093	38.0	0.8	(36.3–39.6)
Wisconsin	7,019	31.8	0.9	(30.1–33.5)
Wyoming	6,339	31.3	1.1	(29.1–33.5)
Guam	2,513	48.6	1.4	(45.8–51.4)
Puerto Rico	5,904	36.2	0.8	(34.6–37.7)
*Median*		*34.7*		
*Range*		*28.4–48.6*		

**Abbreviations:** CI = confidence interval; SE = standard error.

* Age adjusted to the 2000 U.S. standard population.

^†^ Inadequate sleep is defined as <7 hours of sleep during a 24-hour period on average.

**TABLE 30:** Age-adjusted* prevalence estimates of adults aged ≥18 years who reported receiving inadequate sleep^†^ during a 24-hour period, by metropolitan and micropolitan statistical area — Behavioral Risk Factor Surveillance System, United States, 2014

MMSA	Sample size	%	SE	95% CI
Aberdeen, South Dakota	611	30.9	3.0	(25.0–36.9)
Aguadilla-Isabela, Puerto Rico	534	33.6	2.5	(28.7–38.6)
Albuquerque, New Mexico	1,774	32.2	1.5	(29.2–35.2)
Allentown-Bethlehem-Easton, Pennsylvania-New Jersey	1,084	41.2	2.7	(36.0–46.5)
Anchorage, Alaska	1,764	36.3	1.5	(33.3–39.3)
Atlanta-Sandy Springs-Roswell, Georgia	2,741	37.8	1.2	(35.4–40.2)
Augusta-Richmond County, Georgia-South Carolina	876	39.3	3.1	(33.3–45.4)
Austin-Round Rock, Texas	2,228	27.6	1.3	(25.1–30.2)
Baltimore-Columbia-Towson, Maryland	4,547	38.1	1.3	(35.5–40.7)
Baton Rouge, Louisiana	905	36.4	2.0	(32.4–40.4)
Berlin, New Hampshire-Vermont	534	35.7	4.8	(26.3–45.1)
Billings, Montana	800	31.5	2.3	(27.0–36.0)
Birmingham-Hoover, Alabama	1,543	42.4	1.7	(39.0–45.8)
Bismarck, North Dakota	1,030	32.7	2.4	(28.0–37.3)
Boise City, Idaho	1,343	28.8	1.8	(25.2–32.4)
Boston, Massachusetts^§^	4,470	33.7	1.2	(31.3–36.0)
Burlington-South Burlington, Vermont	1,969	28.4	1.3	(25.8–30.9)
Cambridge-Newton-Framingham, Massachusetts^§^	5,109	32.8	1.1	(30.6–34.9)
Camden, New Jersey^§^	1,698	35.7	1.8	(32.1–39.3)
Cedar Rapids, Iowa	636	33.1	2.7	(27.8–38.4)
Charleston, West Virginia	861	37.5	2.2	(33.2–41.9)
Charleston-North Charleston, South Carolina	1,374	38.0	1.8	(34.4–41.5)
Charlotte-Concord-Gastonia, North Carolina-South Carolina	2,104	32.9	1.4	(30.2–35.6)
Chicago-Naperville-Elgin, Illinois-Indiana-Wisconsin	4,110	34.6	1.1	(32.4–36.8)
Cincinnati, Ohio-Kentucky-Indiana	2,019	38.0	1.9	(34.2–41.7)
Claremont-Lebanon, New Hampshire-Vermont	1,670	32.2	2.0	(28.2–36.2)
Cleveland-Elyria, Ohio	957	33.8	2.2	(29.4–38.2)
College Station-Bryan, Texas	560	36.0	3.9	(28.3–43.7)
Colorado Springs, Colorado	1,278	31.7	1.7	(28.3–35.1)
Columbia, South Carolina	1,183	40.5	1.9	(36.8–44.3)
Columbus, Ohio	1,648	39.7	1.9	(36.1–43.4)
Corpus Christi, Texas	604	39.9	3.9	(32.2–47.6)
Dallas-Plano-Irving, Texas^§^	1,275	30.1	1.9	(26.4–33.8)
Dayton, Ohio	561	37.0	3.0	(31.2–42.9)
Denver-Aurora-Lakewood, Colorado	5,733	28.5	0.8	(27.0–30.0)
Des Moines-West Des Moines, Iowa	1,338	27.2	1.8	(23.6–30.8)
Duluth, Minnesota-Wisconsin	934	30.6	2.1	(26.4–34.8)
El Paso, Texas	712	33.1	2.5	(28.2–38.0)
Evansville, Indiana-Kentucky	649	37.9	3.4	(31.3–44.5)
Fargo, North Dakota-Minnesota	1,143	25.4	1.8	(22.0–28.9)
Fayetteville-Springdale-Rogers, Arkansas-Missouri	807	34.8	2.9	(29.2–40.4)
Fort Wayne, Indiana	854	34.7	2.5	(29.7–39.6)
Fort Worth-Arlington, Texas^§^	748	35.7	2.6	(30.7–40.7)
Grand Island, Nebraska	1,045	27.4	2.1	(23.2–31.5)
Grand Rapids-Wyoming, Michigan	894	34.8	2.2	(30.5–39.2)
Greensboro-High Point, North Carolina	513	35.9	3.0	(30.0–41.7)
Greenville-Anderson-Mauldin, South Carolina	1,456	35.7	1.7	(32.4–39.0)
Hagerstown-Martinsburg, Maryland-West Virginia	772	40.7	3.4	(33.9–47.4)
Hartford-West Hartford-East Hartford, Connecticut	2,612	33.7	1.4	(31.0–36.5)
Hilton Head Island-Bluffton-Beaufort, South Carolina	542	38.7	3.6	(31.7–45.7)
Houston-The Woodlands-Sugar Land, Texas	2,129	33.9	1.6	(30.7–37.1)
Huntington-Ashland, West Virginia-Kentucky-Ohio	1,227	40.3	2.1	(36.2–44.5)
Idaho Falls, Idaho	510	34.6	2.8	(29.2–40.0)
Indianapolis-Carmel-Anderson, Indiana	3,559	37.2	1.2	(34.8–39.6)
Jacksonville, Florida	663	39.4	2.8	(34.0–44.9)
Kansas City, Missouri-Kansas	4,818	30.7	1.2	(28.3–33.0)
Kingsport-Bristol-Bristol, Tennessee-Virginia	493	45.3	3.9	(37.7–52.9)
Knoxville, Tennessee	557	36.7	3.3	(30.3–43.2)
Lafayette, Louisiana	557	33.6	2.5	(28.7–38.6)
Lexington-Fayette, Kentucky	615	37.1	2.6	(32.0–42.1)
Lincoln, Nebraska	1,999	32.3	1.4	(29.5–35.1)
Little Rock-North Little Rock-Conway, Arkansas	1,167	35.1	2.1	(31.0–39.3)
Logan, Utah-Idaho	622	28.6	2.1	(24.5–32.6)
Los Angeles-Long Beach-Anaheim, California	2,442	34.6	1.2	(32.1–37.0)
Louisville-Jefferson County, Kentucky-Indiana	2,417	37.0	1.9	(33.3–40.7)
Madison, Wisconsin	550	29.9	2.5	(25.0–34.8)
Memphis, Tennessee-Mississippi-Arkansas	861	30.3	2.6	(25.1–35.4)
Miami-Fort Lauderdale-West Palm Beach, Florida	2,194	35.7	1.5	(32.8–38.7)
Milwaukee-Waukesha-West Allis, Wisconsin	1,347	33.0	2.0	(29.0–36.9)
Minneapolis-St. Paul-Bloomington, Minnesota-Wisconsin	8,675	29.4	0.6	(28.1–30.6)
Minot, North Dakota	594	32.0	2.9	(26.4–37.7)
Montgomery, Alabama	498	35.9	3.0	(30.0–41.7)
Montgomery County-Bucks County-Chester County, Pennsylvania^§^	799	30.7	2.3	(26.2–35.2)
Myrtle Beach-Conway-North Myrtle Beach, South Carolina-North Carolina	981	35.2	2.3	(30.7–39.6)
Nashville-Davidson County-Murfreesboro-Franklin, Tennessee	794	39.2	2.4	(34.4–44.0)
Nassau County-Suffolk County, New York^§^	762	40.9	2.4	(36.1–45.7)
Newark, New Jersey-Pennsylvania^§^	4,087	35.8	1.2	(33.5–38.1)
New Orleans-Metairie, Louisiana	1,889	36.4	1.5	(33.5–39.3)
New York-Jersey City-White Plains, New York-New Jersey^§^	7,405	39.4	0.9	(37.7–41.1)
Norfolk, Nebraska	985	32.4	2.0	(28.5–36.2)
North Platte, Nebraska	953	31.3	2.1	(27.2–35.4)
North Port-Sarasota-Bradenton, Florida	510	43.1	4.2	(34.9–51.4)
Oakland-Hayward-Berkeley, California^§^	698	34.1	2.5	(29.2–38.9)
Ogden-Clearfield, Utah	2,903	32.7	1.0	(30.8–34.7)
Oklahoma City, Oklahoma	2,406	35.1	1.2	(32.7–37.6)
Omaha-Council Bluffs, Nebraska-Iowa	4,830	29.6	0.9	(27.7–31.4)
Orlando-Kissimmee-Sanford, Florida	946	34.1	2.1	(29.9–38.3)
Philadelphia, Pennsylvania^§^	1,494	40.9	1.9	(37.2–44.6)
Phoenix-Mesa-Scottsdale, Arizona	9,304	33.0	0.8	(31.4–34.6)
Pittsburgh, Pennsylvania	2,395	39.9	1.5	(37.0–42.7)
Ponce, Puerto Rico	521	35.0	2.8	(29.5–40.5)
Portland-South Portland, Maine	2,732	28.7	1.4	(26.0–31.3)
Portland-Vancouver-Hillsboro, Oregon-Washington	2,810	31.5	1.3	(29.0–34.0)
Providence-Warwick, Rhode Island-Massachusetts	7,968	36.9	1.0	(34.9–39.0)
Provo-Orem, Utah	2,133	29.1	1.1	(26.9–31.3)
Raleigh, North Carolina	718	28.1	2.0	(24.2–32.0)
Rapid City, South Dakota	1,407	29.1	2.0	(25.2–33.0)
Reno, Nevada	1,188	32.4	2.0	(28.5–36.3)
Richmond, Virginia	1,446	33.0	1.7	(29.6–36.4)
Riverside-San Bernardino-Ontario, California	934	36.3	2.0	(32.5–40.2)
Roanoke, Virginia	528	33.5	3.0	(27.6–39.4)
Rochester, Minnesota	691	31.7	2.3	(27.1–36.3)
Rockingham County-Strafford County, New Hampshire^§^	1,421	28.7	1.8	(25.2–32.2)
Sacramento-Roseville-Arden-Arcade, California	641	30.2	2.3	(25.7–34.8)
St. Cloud, Minnesota	560	25.6	2.2	(21.2–29.9)
St. Louis, Missouri-Illinois	1,880	34.7	1.8	(31.2–38.3)
Salisbury, Maryland-Delaware	1,914	39.2	2.3	(34.6–43.7)
Salt Lake City, Utah	5,368	31.0	0.7	(29.6–32.5)
San Antonio-New Braunfels, Texas	2,246	35.5	1.4	(32.8–38.2)
San Juan-Carolina-Caguas, Puerto Rico	3,698	37.1	1.0	(35.1–39.2)
Scottsbluff, Nebraska	889	35.8	2.3	(31.2–40.4)
Seattle-Bellevue-Everett, Washington^§^	3,667	30.7	1.1	(28.6–32.8)
Shreveport-Bossier City, Louisiana	538	38.8	2.8	(33.3–44.3)
Silver Spring-Frederick-Rockville, Maryland^§^	2,344	34.7	1.9	(31.1–38.4)
Sioux City, Iowa-Nebraska-South Dakota	1,130	37.3	3.0	(31.4–43.1)
Sioux Falls, South Dakota	1,342	29.8	2.1	(25.7–33.9)
Spartanburg, South Carolina	549	38.1	3.4	(31.4–44.7)
Spokane-Spokane Valley, Washington	716	28.6	2.4	(23.9–33.3)
Springfield, Massachusetts	1,092	36.0	2.3	(31.6–40.5)
Tampa-St. Petersburg-Clearwater, Florida	1,570	40.1	1.9	(36.3–43.9)
Toledo, Ohio	643	37.2	3.1	(31.1–43.4)
Topeka, Kansas	1,425	31.9	1.7	(28.6–35.2)
Tulsa, Oklahoma	1,998	35.4	1.4	(32.7–38.2)
Tuscaloosa, Alabama	706	37.3	2.5	(32.5–42.2)
Virginia Beach-Norfolk-Newport News, Virginia-North Carolina	1,853	39.0	1.7	(35.7–42.2)
Warren-Troy-Farmington Hills, Michigan^§^	2,096	39.4	1.5	(36.4–42.4)
Washington-Arlington-Alexandria, District of Columbia-Virginia-Maryland-West Virginia^§^	8,131	35.8	1.0	(33.8–37.7)
Wichita, Kansas	2,705	32.7	1.1	(30.5–34.9)
Wichita Falls, Texas	530	30.8	5.2	(20.7–41.0)
Wilmington, Delaware-Maryland-New Jersey^§^	2,720	38.1	1.5	(35.1–41.0)
Worcester, Massachusetts-Connecticut	2,426	37.5	1.6	(34.4–40.6)
Youngstown-Warren-Boardman, Ohio-Pennsylvania	517	40.0	3.7	(32.7–47.3)
*Median*		*34.8*		
*Range*		*25.4–45.3*		

**Abbreviations:** CI = confidence interval; MMSA = metropolitan and micropolitan statistical areas; SE = standard error.

* Age adjusted to the 2000 U.S. standard population.

^†^ Inadequate sleep is defined as <7 hours of sleep during a 24-hour period on average.

^§^ Metropolitan division.

#### Current Cigarette Smoking

Current cigarette smokers were defined as respondents who reported having smoked at least 100 cigarettes in their lifetime and who smoked every day or some days at the time of the interview. In 2013, age-adjusted prevalence estimates of current cigarette smoking ranged from 10.1% in Utah to 28.8% in West Virginia (median: 19.3%) (Table 31). Among selected MMSA, age-adjusted prevalence estimates ranged from 6.1% in Provo-Orem, Utah, to 33.6% in Kingsport-Bristol-Bristol, Tennessee-Virginia (median: 19.5%) (Table 32). In 2014, age-adjusted prevalence estimates ranged from 9.5% in Utah to 28.1% in West Virginia (median: 18.7%) (Table 33). Among selected MMSA, age-adjusted prevalence estimates ranged from 5.0% in Logan, Utah-Idaho, to 29.3% in Charleston, West Virginia (median: 18.6%) (Table 34).

**TABLE 31:** Age-adjusted* prevalence estimates of current smoking^†^ among adults aged ≥18 years, by state/territory — Behavioral Risk Factor Surveillance System, United States, 2013

State/Territory	Sample size	%	SE	95% CI
Alabama	6,383	21.9	0.9	(20.2–23.6)
Alaska	4,476	22.0	0.9	(20.3–23.8)
Arizona	4,111	16.4	1.0	(14.4–18.5)
Arkansas	5,101	26.7	1.0	(24.7–28.7)
California	10,622	12.5	0.5	(11.6–13.4)
Colorado	12,774	17.7	0.5	(16.8–18.7)
Connecticut	7,479	15.9	0.7	(14.6–17.2)
Delaware	5,052	20.1	0.9	(18.4–21.9)
District of Columbia	4,702	18.7	1.0	(16.8–20.6)
Florida	33,028	17.5	0.5	(16.5–18.5)
Georgia	7,725	18.8	0.7	(17.5–20.1)
Hawaii	7,676	13.9	0.6	(12.6–15.1)
Idaho	5,491	17.5	0.8	(15.9–19.1)
Illinois	5,526	18.3	0.8	(16.7–19.8)
Indiana	10,068	22.4	0.6	(21.2–23.6)
Iowa	7,983	20.1	0.7	(18.7–21.5)
Kansas	22,891	20.4	0.4	(19.7–21.1)
Kentucky	10,584	27.2	0.7	(25.8–28.7)
Louisiana	5,162	23.7	1.1	(21.6–25.9)
Maine	7,962	21.9	0.8	(20.4–23.4)
Maryland	12,593	16.6	0.6	(15.4–17.7)
Massachusetts	14,274	16.9	0.6	(15.8–18.0)
Michigan	12,519	22.1	0.6	(20.9–23.2)
Minnesota	13,794	18.4	0.6	(17.2–19.6)
Mississippi	7,243	25.2	0.8	(23.5–26.8)
Missouri	6,988	22.6	0.8	(21.0–24.2)
Montana	9,526	19.7	0.6	(18.5–20.9)
Nebraska	16,687	18.9	0.5	(17.8–19.9)
Nevada	4,984	19.3	1.1	(17.2–21.4)
New Hampshire	6,291	16.9	0.7	(15.5–18.4)
New Jersey	12,590	15.9	0.5	(14.9–16.9)
New Mexico	9,096	19.4	0.7	(18.1–20.7)
New York	8,659	17.0	0.6	(15.8–18.2)
North Carolina	8,698	20.5	0.6	(19.3–21.7)
North Dakota	7,655	21.7	0.8	(20.2–23.2)
Ohio	11,490	24.1	0.6	(22.9–25.4)
Oklahoma	8,133	24.2	0.7	(22.8–25.5)
Oregon	5,784	17.7	0.8	(16.2–19.2)
Pennsylvania	11,109	21.8	0.6	(20.6–23.0)
Rhode Island	6,246	18.1	0.8	(16.6–19.6)
South Carolina	10,481	22.6	0.7	(21.3–23.9)
South Dakota	6,769	20.4	0.8	(18.8–22.1)
Tennessee	5,467	24.6	0.9	(22.8–26.4)
Texas	10,594	15.8	0.6	(14.7–16.9)
Utah	12,451	10.1	0.4	(9.4–10.8)
Vermont	6,241	17.5	0.7	(16.1–19.0)
Virginia	8,091	19.1	0.6	(17.9–20.3)
Washington	10,996	16.2	0.5	(15.2–17.3)
West Virginia	5,841	28.8	0.8	(27.2–30.4)
Wisconsin	6,249	19.2	0.9	(17.5–20.9)
Wyoming	6,315	21.0	0.9	(19.3–22.7)
Guam	1,835	25.8	1.4	(23.1–28.5)
Puerto Rico	5,966	11.1	0.6	(9.9–12.3)
*Median*		*19.3*		
*Range*		*10.1–28.8*		

**Abbreviations:** CI = confidence interval; SE = standard error.

* Age adjusted to the 2000 U.S. standard population.

^†^ Current smoking is defined as having smoked at least 100 cigarettes and smoking daily or some days during the period of the survey.

**TABLE 32:** Age-adjusted* prevalence estimates of current smoking^†^ among adults aged ≥18 years, by metropolitan and micropolitan statistical area — Behavioral Risk Factor Surveillance System, United States, 2013

MMSA	Sample size	%	SE	95% CI
Aguadilla-Isabela, Puerto Rico	586	12.9	2.2	(8.7–17.2)
Akron, Ohio	663	20.4	2.5	(15.5–25.3)
Albuquerque, New Mexico	2,037	19.2	1.2	(16.8–21.5)
Allentown-Bethlehem-Easton, Pennsylvania-New Jersey	993	21.2	2.2	(16.9–25.5)
Anchorage, Alaska	1,486	18.9	1.3	(16.3–21.5)
Atlanta-Sandy Springs-Roswell, Georgia	3,331	16.0	0.9	(14.2–17.8)
Augusta-Richmond County, Georgia-South Carolina	888	19.0	2.2	(14.7–23.4)
Austin-Round Rock, Texas	919	13.5	1.5	(10.6–16.5)
Baltimore-Columbia-Towson, Maryland	4,610	18.8	1.0	(16.9–20.7)
Baton Rouge, Louisiana	911	22.6	2.3	(18.0–27.2)
Billings, Montana	802	19.0	1.6	(15.9–22.1)
Birmingham-Hoover, Alabama	1,325	22.1	1.7	(18.8–25.5)
Bismarck, North Dakota	1,009	23.2	2.0	(19.3–27.1)
Boise City, Idaho	1,451	18.6	1.5	(15.7–21.6)
Boston, Massachusetts^§ ^	3,849	15.7	1.0	(13.7–17.8)
Buffalo-Cheektowaga-Niagara Falls, New York	495	24.9	3.1	(18.9–30.9)
Burlington-South Burlington, Vermont	1,596	15.5	1.2	(13.1–17.9)
Cambridge-Newton-Framingham, Massachusetts^§^	4,632	14.0	0.9	(12.3–15.8)
Camden, New Jersey^§^	1,761	20.9	1.4	(18.1–23.6)
Cedar Rapids, Iowa	634	18.1	2.2	(13.8–22.3)
Charleston, West Virginia	811	28.3	2.2	(24.0–32.6)
Charleston-North Charleston, South Carolina	1,518	20.4	1.5	(17.5–23.4)
Charlotte-Concord-Gastonia, North Carolina-South Carolina	1,923	19.6	1.2	(17.1–22.0)
Chattanooga, Tennessee-Georgia	531	25.4	3.4	(18.7–32.1)
Chicago-Naperville-Elgin, Illinois-Indiana-Wisconsin	3,273	16.7	1.0	(14.7–18.6)
Cincinnati, Ohio-Kentucky-Indiana	2,504	22.0	1.4	(19.3–24.6)
Claremont-Lebanon, New Hampshire-Vermont	1,648	15.2	1.4	(12.5–18.0)
Cleveland-Elyria, Ohio	1,053	23.4	1.9	(19.7–27.2)
Colorado Springs, Colorado	1,293	18.1	1.5	(15.2–20.9)
Columbia, South Carolina	1,423	21.5	1.6	(18.4–24.5)
Columbus, Ohio	1,794	23.1	1.3	(20.5–25.7)
Crestview-Fort Walton Beach-Destin, Florida	1,042	22.8	2.0	(19.0–26.7)
Dallas-Plano-Irving, Texas^§^	866	12.7	1.4	(10.0–15.3)
Davenport-Moline-Rock Island, Iowa-Illinois	664	19.8	2.6	(14.6–24.9)
Dayton, Ohio	805	22.3	2.4	(17.6–27.0)
Deltona-Daytona Beach-Ormond Beach, Florida	1,078	21.3	2.5	(16.4–26.2)
Denver-Aurora-Lakewood, Colorado	5,366	17.3	0.7	(16.0–18.7)
Des Moines-West Des Moines, Iowa	1,320	18.2	1.6	(15.1–21.3)
Duluth, Minnesota-Wisconsin	681	27.9	3.1	(21.8–33.9)
Durham-Chapel Hill, North Carolina	609	14.5	2.0	(10.5–18.5)
El Paso, Texas	731	14.0	1.8	(10.4–17.5)
Evansville, Indiana-Kentucky	564	23.6	2.7	(18.3–29.0)
Fargo, North Dakota-Minnesota	1,167	20.3	1.7	(16.9–23.7)
Fayetteville-Springdale-Rogers, Arkansas-Missouri	799	23.5	2.4	(18.7–28.2)
Fort Smith, Arkansas-Oklahoma	486	26.3	3.1	(20.1–32.4)
Fort Wayne, Indiana	763	18.9	2.0	(15.0–22.7)
Fort Worth-Arlington, Texas^§^	793	16.5	1.9	(12.7–20.3)
Gainesville, Florida	1,005	16.5	2.4	(11.8–21.3)
Grand Forks, North Dakota-Minnesota	493	14.5	2.4	(9.7–19.3)
Grand Island, Nebraska	779	20.5	2.3	(15.9–25.1)
Grand Rapids-Wyoming, Michigan	1,325	19.6	1.7	(16.3–22.8)
Greensboro-High Point, North Carolina	649	20.7	2.3	(16.3–25.2)
Greenville-Anderson-Mauldin, South Carolina	1,315	22.6	1.8	(19.1–26.0)
Gulfport-Biloxi-Pascagoula, Mississippi	742	26.7	2.3	(22.2–31.1)
Hagerstown-Martinsburg, Maryland-West Virginia	750	25.4	2.7	(20.1–30.6)
Hartford-West Hartford-East Hartford, Connecticut	2,763	14.8	1.1	(12.7–17.0)
Hilton Head Island-Bluffton-Beaufort, South Carolina	802	18.3	2.7	(13.1–23.5)
Houston-The Woodlands-Sugar Land, Texas	1,343	13.6	1.4	(11.0–16.3)
Huntington-Ashland, West Virginia-Kentucky-Ohio	1,145	30.4	1.9	(26.7–34.0)
Idaho Falls, Idaho	494	18.0	2.6	(12.9–23.1)
Indianapolis-Carmel-Anderson, Indiana	2,474	20.8	1.1	(18.6–23.1)
Jackson, Mississippi	778	21.5	2.2	(17.3–25.8)
Jacksonville, Florida	2,789	17.5	1.1	(15.3–19.8)
Kansas City, Missouri-Kansas	7,293	19.7	1.0	(17.6–21.7)
Kingsport-Bristol-Bristol, Tennessee-Virginia	511	33.6	3.8	(26.2–41.0)
Knoxville, Tennessee	622	22.0	2.2	(17.7–26.3)
Lansing-East Lansing, Michigan	669	19.3	2.1	(15.2–23.3)
Lexington-Fayette, Kentucky	619	21.9	2.1	(17.7–26.1)
Lincoln, Nebraska	1,828	18.9	1.2	(16.6–21.2)
Little Rock-North Little Rock-Conway, Arkansas	1,109	21.6	1.8	(18.1–25.1)
Logan, Utah-Idaho	624	8.7	1.6	(5.6–11.8)
Los Angeles-Long Beach-Anaheim, California	2,770	11.3	0.8	(9.6–12.9)
Louisville-Jefferson County, Kentucky-Indiana	2,071	23.5	1.5	(20.5–26.5)
Lubbock, Texas	522	19.4	2.6	(14.3–24.5)
Manhattan, Kansas	654	18.0	1.8	(14.4–21.6)
Memphis, Tennessee-Mississippi-Arkansas	1,150	21.6	1.9	(17.8–25.4)
Miami-Fort Lauderdale-West Palm Beach, Florida	2,127	12.7	1.2	(10.3–15.0)
Milwaukee-Waukesha-West Allis, Wisconsin	1,194	20.8	1.9	(17.0–24.5)
Minneapolis-St. Paul-Bloomington, Minnesota-Wisconsin	8,756	15.7	0.7	(14.3–17.1)
Minot, North Dakota	640	22.0	2.3	(17.5–26.5)
Montgomery County-Bucks County-Chester County, Pennsylvania^§^	946	19.7	1.7	(16.4–23.0)
Myrtle Beach-Conway-North Myrtle Beach, South Carolina-North Carolina	760	24.2	2.4	(19.4–29.0)
Nashville-Davidson County-Murfreesboro-Franklin, Tennessee	1,015	19.4	1.7	(16.0–22.8)
Nassau County-Suffolk County, New York^§^	906	17.7	1.9	(14.1–21.4)
Newark, New Jersey-Pennsylvania^§^	3,877	13.7	0.9	(12.0–15.4)
New Orleans-Metairie, Louisiana	1,259	24.0	2.3	(19.6–28.5)
New York-Jersey City-White Plains, New York-New Jersey^§^	8,481	15.0	0.6	(13.8–16.2)
Norfolk, Nebraska	649	19.5	2.0	(15.7–23.4)
North Platte, Nebraska	697	19.4	2.2	(15.0–23.8)
North Port-Sarasota-Bradenton, Florida	1,039	22.1	2.3	(17.7–26.6)
Oakland-Hayward-Berkeley, California^§^	652	11.5	1.5	(8.5–14.5)
Ogden-Clearfield, Utah	2,415	10.4	0.8	(8.8–11.9)
Oklahoma City, Oklahoma	2,610	21.1	1.1	(19.0–23.3)
Omaha-Council Bluffs, Nebraska-Iowa	3,045	19.3	1.0	(17.2–21.3)
Orlando-Kissimmee-Sanford, Florida	2,198	16.0	1.2	(13.5–18.4)
Panama City, Florida	996	26.4	2.4	(21.6–31.2)
Pensacola-Ferry Pass-Brent, Florida	1,266	24.0	1.6	(20.8–27.2)
Philadelphia, Pennsylvania^§^	1,708	21.9	1.5	(19.0–24.7)
Phoenix-Mesa-Scottsdale, Arizona	1,501	15.7	1.4	(12.9–18.4)
Pittsburgh, Pennsylvania	2,301	21.5	1.3	(18.9–24.1)
Ponce, Puerto Rico	527	8.4	1.8	(5.0–11.9)
Portland-South Portland, Maine	2,595	19.7	1.2	(17.3–22.2)
Portland-Vancouver-Hillsboro, Oregon-Washington	3,178	16.2	0.9	(14.3–18.0)
Port St. Lucie, Florida	985	19.5	2.3	(15.0–24.0)
Providence-Warwick, Rhode Island-Massachusetts	7,926	18.7	0.8	(17.1–20.4)
Provo-Orem, Utah	1,811	6.1	0.7	(4.8–7.4)
Raleigh, North Carolina	659	13.8	1.6	(10.7–16.8)
Rapid City, South Dakota	855	24.1	2.1	(19.9–28.2)
Reno, Nevada	1,773	15.4	1.1	(13.1–17.6)
Richmond, Virginia	1,247	21.0	1.6	(17.9–24.1)
Riverside-San Bernardino-Ontario, California	1,279	14.8	1.3	(12.4–17.3)
Rochester, New York	494	16.0	2.4	(11.3–20.6)
Rockingham County-Strafford County, New Hampshire^§^	1,614	16.6	1.4	(13.9–19.2)
Sacramento-Roseville-Arden-Arcade, California	839	16.4	1.9	(12.7–20.0)
St. Louis, Missouri-Illinois	2,015	21.4	1.5	(18.5–24.2)
Salem, Oregon	507	15.8	2.7	(10.5–21.2)
Salina, Kansas	516	20.3	2.4	(15.5–25.0)
Salisbury, Maryland-Delaware	1,983	19.5	1.6	(16.4–22.6)
Salt Lake City, Utah	4,537	10.9	0.6	(9.8–12.1)
San Antonio-New Braunfels, Texas	914	15.2	1.7	(11.9–18.4)
San Francisco-Redwood City-South San Francisco, California^§^	499	11.7	2.3	(7.1–16.3)
San Jose-Sunnyvale-Santa Clara, California	574	9.4	2.0	(5.5–13.3)
San Juan-Carolina-Caguas, Puerto Rico	3,632	11.7	0.8	(10.2–13.2)
Scottsbluff, Nebraska	700	23.8	2.6	(18.8–28.9)
Scranton-Wilkes-Barre-Hazleton, Pennsylvania	554	24.3	2.7	(19.1–29.6)
Seattle-Bellevue-Everett, Washington^§^	3,707	13.3	0.8	(11.8–14.8)
Shreveport-Bossier City, Louisiana	565	20.7	2.7	(15.4–26.0)
Silver Spring-Frederick-Rockville, Maryland^§^	2,350	10.9	1.1	(8.7–13.0)
Sioux City, Iowa-Nebraska-South Dakota	1,031	22.6	2.9	(16.9–28.3)
Sioux Falls, South Dakota	991	16.3	1.7	(13.0–19.5)
Spartanburg, South Carolina	581	25.2	2.9	(19.6–30.8)
Spokane-Spokane Valley, Washington	848	20.4	2.1	(16.2–24.6)
Springfield, Massachusetts	1,496	21.3	2.1	(17.2–25.5)
Tallahassee, Florida	1,800	14.5	1.6	(11.4–17.6)
Tampa-St. Petersburg-Clearwater, Florida	2,129	20.9	1.4	(18.1–23.6)
Toledo, Ohio	963	24.7	2.5	(19.8–29.6)
Topeka, Kansas	2,365	20.5	1.1	(18.3–22.8)
Tulsa, Oklahoma	1,979	22.3	1.4	(19.6–25.0)
Virginia Beach-Norfolk-Newport News, Virginia-North Carolina	1,604	21.2	1.6	(18.0–24.4)
Warren-Troy-Farmington Hills, Michigan^§^	2,212	21.2	1.4	(18.5–23.8)
Washington-Arlington-Alexandria, District of Columbia-Virginia-Maryland-West Virginia^§^	8,660	14.1	0.7	(12.7–15.5)
Wichita, Kansas	4,857	22.3	0.8	(20.7–23.9)
Wilmington, Delaware-Maryland-New Jersey^§ ^	3,179	19.3	1.1	(17.2–21.4)
Winston-Salem, North Carolina	679	28.0	2.8	(22.6–33.4)
Worcester, Massachusetts-Connecticut	2,656	20.5	1.4	(17.8–23.3)
*Median*		*19.5*		
*Range*		*6.1–33.6*		

**Abbreviations:** CI = confidence interval; MMSA = metropolitan and micropolitan statistical areas; SE = standard error.

* Age adjusted to the 2000 U.S. standard population.

^†^ Current smoking is defined as having smoked at least 100 cigarettes and smoking daily or some days during the period of the survey.

^§^ Metropolitan division.

**TABLE 33:** Age-adjusted* prevalence estimates of current smoking^†^ among adults aged ≥18 years, by state/territory — Behavioral Risk Factor Surveillance System, United States, 2014

State/Territory	Sample size	%	SE	95% CI
Alabama	8,368	21.7	0.8	(20.2–23.2)
Alaska	4,226	19.5	0.9	(17.8–21.2)
Arizona	14,002	16.9	0.6	(15.7–18.1)
Arkansas	4,925	25.4	1.1	(23.3–27.6)
California	8,195	12.8	0.5	(11.8–13.8)
Colorado	12,253	15.9	0.5	(14.9–16.8)
Connecticut	7,447	16.0	0.7	(14.7–17.4)
Delaware	4,148	20.6	1.1	(18.5–22.7)
District of Columbia	3,832	16.4	1.1	(14.3–18.5)
Florida	9,207	18.7	0.7	(17.3–20.0)
Georgia	5,952	17.4	0.7	(15.9–18.8)
Hawaii	6,890	14.6	0.6	(13.4–15.9)
Idaho	5,257	16.3	0.8	(14.7–17.9)
Illinois	4,844	16.6	0.8	(15.1–18.1)
Indiana	10,978	23.4	0.6	(22.2–24.6)
Iowa	7,751	19.1	0.7	(17.8–20.4)
Kansas	13,163	18.4	0.5	(17.5–19.3)
Kentucky	10,767	26.8	0.8	(25.2–28.4)
Louisiana	6,514	24.3	0.7	(22.9–25.7)
Maine	8,808	21.2	0.8	(19.7–22.7)
Maryland	12,126	14.6	0.7	(13.3–15.9)
Massachusetts	14,686	14.9	0.5	(13.8–15.9)
Michigan	8,231	22.0	0.7	(20.7–23.4)
Minnesota	15,842	16.6	0.4	(15.8–17.4)
Mississippi	4,073	23.6	1.0	(21.5–25.6)
Missouri	6,832	21.1	0.8	(19.5–22.7)
Montana	7,299	21.0	0.8	(19.4–22.6)
Nebraska	21,729	17.8	0.5	(16.8–18.7)
Nevada	3,651	17.1	1.1	(15.0–19.2)
New Hampshire	5,855	18.6	0.9	(16.8–20.3)
New Jersey	12,194	15.4	0.5	(14.4–16.5)
New Mexico	8,365	19.7	0.8	(18.2–21.2)
New York	6,408	14.6	0.6	(13.4–15.8)
North Carolina	6,982	19.4	0.6	(18.2–20.6)
North Dakota	7,499	20.3	0.8	(18.7–22.0)
Ohio	10,616	21.7	0.7	(20.2–23.1)
Oklahoma	8,194	21.5	0.7	(20.2–22.8)
Oregon	4,968	17.5	0.8	(15.9–19.0)
Pennsylvania	10,489	20.7	0.6	(19.4–21.9)
Rhode Island	6,097	16.8	0.8	(15.2–18.5)
South Carolina	10,635	22.2	0.6	(21.0–23.5)
South Dakota	7,186	19.1	0.9	(17.4–20.8)
Tennessee	4,851	24.8	1.0	(22.8–26.8)
Texas	14,536	14.5	0.5	(13.5–15.4)
Utah	14,459	9.5	0.3	(8.9–10.1)
Vermont	6,168	17.8	0.7	(16.4–19.1)
Virginia	9,100	19.9	0.6	(18.6–21.1)
Washington	9,727	15.5	0.6	(14.4–16.7)
West Virginia	6,086	28.1	0.8	(26.5–29.7)
Wisconsin	6,727	17.8	0.8	(16.3–19.3)
Wyoming	6,078	19.9	1.0	(17.9–21.9)
Guam	2,370	27.8	1.3	(25.2–30.4)
Puerto Rico	5,907	11.6	0.6	(10.5–12.8)
*Median*		*18.7*		
*Range*		*9.5–28.1*		

**Abbreviations:** CI = confidence interval; SE = standard error.

* Age adjusted to the 2000 U.S. standard population.

^†^ Current smoking is defined as having smoked at least 100 cigarettes and smoking daily or some days during the period of the survey.

**TABLE 34:** Age-adjusted* prevalence estimates of current smoking^†^ among adults aged ≥18 years, by metropolitan and micropolitan statistical area — Behavioral Risk Factor Surveillance System, United States, 2014

MMSA	Sample size	%	SE	95% CI
Aberdeen, South Dakota	603	20.7	3.1	(14.7–26.7)
Aguadilla-Isabela, Puerto Rico	542	10.8	1.7	(7.4–14.2)
Albuquerque, New Mexico	1,669	19.8	1.5	(17.0–22.7)
Allentown-Bethlehem-Easton, Pennsylvania-New Jersey	1,040	21.6	2.4	(17.0–26.3)
Anchorage, Alaska	1,716	17.6	1.2	(15.3–19.9)
Atlanta-Sandy Springs-Roswell, Georgia	2,610	14.4	1.0	(12.5–16.3)
Augusta-Richmond County, Georgia-South Carolina	844	21.1	2.6	(16.1–26.2)
Austin-Round Rock, Texas	2,140	11.1	1.0	(9.2–13.1)
Baltimore-Columbia-Towson, Maryland	4,437	17.2	1.1	(15.0–19.4)
Baton Rouge, Louisiana	894	22.5	1.9	(18.7–26.2)
Berlin, New Hampshire-Vermont	521	20.3	3.7	(13.0–27.6)
Billings, Montana	779	25.5	2.3	(21.0–30.1)
Birmingham-Hoover, Alabama	1,519	18.4	1.5	(15.6–21.3)
Bismarck, North Dakota	1,006	21.4	2.2	(17.1–25.7)
Boise City, Idaho	1,290	14.6	1.5	(11.7–17.5)
Boston, Massachusetts^§^	4,274	14.5	1.0	(12.6–16.5)
Burlington-South Burlington, Vermont	1,890	15.3	1.1	(13.1–17.4)
Cambridge-Newton-Framingham, Massachusetts^§^	4,877	11.6	0.8	(10.1–13.1)
Camden, New Jersey^§^	1,627	14.1	1.5	(11.3–17.0)
Cedar Rapids, Iowa	611	18.6	2.3	(14.0–23.1)
Charleston, West Virginia	862	29.3	2.2	(25.0–33.5)
Charleston-North Charleston, South Carolina	1,358	21.9	1.7	(18.6–25.2)
Charlotte-Concord-Gastonia, North Carolina-South Carolina	2,062	19.2	1.2	(16.8–21.7)
Chicago-Naperville-Elgin, Illinois-Indiana-Wisconsin	3,924	16.2	0.9	(14.5–17.9)
Cincinnati, Ohio-Kentucky-Indiana	1,978	23.9	1.8	(20.4–27.5)
Claremont-Lebanon, New Hampshire-Vermont	1,626	22.2	1.9	(18.4–26.0)
Cleveland-Elyria, Ohio	944	19.1	2.1	(15.1–23.2)
College Station-Bryan, Texas	556	11.2	2.4	(6.6–15.9)
Colorado Springs, Colorado	1,195	18.1	1.6	(15.0–21.1)
Columbia, South Carolina	1,173	21.0	1.7	(17.7–24.3)
Columbus, Ohio	1,603	19.4	1.6	(16.2–22.6)
Corpus Christi, Texas	592	15.5	2.7	(10.3–20.8)
Dallas-Plano-Irving, Texas^§^	1,221	12.1	1.5	(9.1–15.0)
Dayton, Ohio	556	22.4	2.8	(16.9–27.8)
Denver-Aurora-Lakewood, Colorado	5,303	15.6	0.7	(14.3–16.9)
Des Moines-West Des Moines, Iowa	1,282	18.6	1.7	(15.3–22.0)
Duluth, Minnesota-Wisconsin	920	24.9	2.1	(20.8–29.1)
El Paso, Texas	668	13.3	2.0	(9.3–17.3)
Evansville, Indiana-Kentucky	626	21.6	3.0	(15.6–27.5)
Fargo, North Dakota-Minnesota	1,106	15.2	1.6	(12.1–18.3)
Fayetteville-Springdale-Rogers, Arkansas-Missouri	779	21.8	2.6	(16.8–26.8)
Fort Wayne, Indiana	828	22.4	2.4	(17.8–27.1)
Fort Worth-Arlington, Texas^§^	720	15.4	2.1	(11.4–19.5)
Grand Island, Nebraska	1,014	17.0	2.0	(13.1–20.9)
Grand Rapids-Wyoming, Michigan	872	18.6	2.0	(14.7–22.5)
Greensboro-High Point, North Carolina	495	20.2	2.6	(15.2–25.3)
Greenville-Anderson-Mauldin, South Carolina	1,445	18.0	1.4	(15.2–20.7)
Hagerstown-Martinsburg, Maryland-West Virginia	765	24.6	3.2	(18.4–30.8)
Hartford-West Hartford-East Hartford, Connecticut	2,487	15.6	1.1	(13.4–17.9)
Hilton Head Island-Bluffton-Beaufort, South Carolina	527	21.4	4.1	(13.5–29.4)
Houston-The Woodlands-Sugar Land, Texas	2,003	11.9	1.1	(9.6–14.1)
Huntington-Ashland, West Virginia-Kentucky-Ohio	1,214	26.3	2.0	(22.3–30.3)
Idaho Falls, Idaho	499	10.4	1.8	(6.9–13.9)
Indianapolis-Carmel-Anderson, Indiana	3,443	20.6	1.1	(18.5–22.8)
Jacksonville, Florida	636	19.9	2.4	(15.2–24.7)
Kansas City, Missouri-Kansas	4,696	18.4	1.1	(16.2–20.6)
Kingsport-Bristol-Bristol, Tennessee-Virginia	481	27.4	3.6	(20.4–34.5)
Knoxville, Tennessee	536	24.4	3.3	(18.0–30.8)
Lafayette, Louisiana	534	25.1	2.4	(20.3–29.9)
Lexington-Fayette, Kentucky	602	28.4	2.4	(23.6–33.2)
Lincoln, Nebraska	1,964	18.3	1.2	(16.0–20.7)
Little Rock-North Little Rock-Conway, Arkansas	1,118	22.2	2.0	(18.3–26.1)
Logan, Utah-Idaho	602	5.0	1.0	(3.0–6.9)
Los Angeles-Long Beach-Anaheim, California	2,165	12.8	0.9	(10.9–14.6)
Louisville-Jefferson County, Kentucky-Indiana	2,350	21.5	1.7	(18.1–24.9)
Madison, Wisconsin	528	11.5	2.0	(7.5–15.4)
Memphis, Tennessee-Mississippi-Arkansas	823	18.7	2.4	(14.1–23.4)
Miami-Fort Lauderdale-West Palm Beach, Florida	2,020	14.9	1.3	(12.4–17.4)
Milwaukee-Waukesha-West Allis, Wisconsin	1,284	17.5	1.7	(14.2–20.9)
Minneapolis-St. Paul-Bloomington, Minnesota-Wisconsin	8,452	15.1	0.5	(14.2–16.1)
Minot, North Dakota	574	23.9	2.8	(18.3–29.4)
Montgomery, Alabama	491	21.2	2.7	(15.9–26.5)
Montgomery County-Bucks County-Chester County, Pennsylvania^§^	780	13.3	1.8	(9.9–16.8)
Myrtle Beach-Conway-North Myrtle Beach, South Carolina-North Carolina	958	21.3	2.0	(17.4–25.2)
Nashville-Davidson County-Murfreesboro-Franklin, Tennessee	756	23.4	2.2	(19.0–27.8)
Nassau County-Suffolk County, New York^§^	722	12.9	1.7	(9.7–16.2)
Newark, New Jersey-Pennsylvania^§^	3,867	16.0	1.0	(13.9–18.0)
New Orleans-Metairie, Louisiana	1,856	21.9	1.3	(19.3–24.4)
New York-Jersey City-White Plains, New York-New Jersey^§^	6,982	13.1	0.6	(11.9–14.3)
Norfolk, Nebraska	953	19.8	1.7	(16.5–23.1)
North Platte, Nebraska	928	26.8	2.1	(22.7–30.8)
North Port-Sarasota-Bradenton, Florida	488	23.6	4.1	(15.6–31.7)
Oakland-Hayward-Berkeley, California^§^	661	10.1	1.5	(7.3–13.0)
Ogden-Clearfield, Utah	2,829	10.8	0.7	(9.4–12.2)
Oklahoma City, Oklahoma	2,366	18.7	1.1	(16.6–20.8)
Omaha-Council Bluffs, Nebraska-Iowa	4,725	17.6	0.8	(15.9–19.2)
Orlando-Kissimmee-Sanford, Florida	901	16.4	1.8	(13.0–19.9)
Philadelphia, Pennsylvania^§^	1,427	22.0	1.7	(18.8–25.3)
Phoenix-Mesa-Scottsdale, Arizona	8,831	16.9	0.7	(15.5–18.2)
Pittsburgh, Pennsylvania	2,301	21.0	1.3	(18.4–23.5)
Ponce, Puerto Rico	524	13.6	2.3	(9.1–18.1)
Portland-South Portland, Maine	2,665	16.6	1.3	(14.2–19.1)
Portland-Vancouver-Hillsboro, Oregon-Washington	2,699	14.9	1.0	(13.0–16.9)
Providence-Warwick, Rhode Island-Massachusetts	7,648	17.6	0.9	(15.8–19.4)
Provo-Orem, Utah	2,077	5.3	0.6	(4.2–6.4)
Raleigh, North Carolina	698	15.8	1.8	(12.4–19.3)
Rapid City, South Dakota	1,370	20.6	1.8	(17.0–24.2)
Reno, Nevada	1,169	15.0	1.5	(12.0–18.0)
Richmond, Virginia	1,404	20.0	1.5	(17.0–23.0)
Riverside-San Bernardino-Ontario, California	884	14.2	1.5	(11.3–17.2)
Roanoke, Virginia	510	24.5	3.1	(18.5–30.6)
Rochester, Minnesota	684	15.6	2.0	(11.7–19.4)
Rockingham County-Strafford County, New Hampshire^§^	1,367	18.8	1.7	(15.4–22.2)
Sacramento-Roseville-Arden-Arcade, California	605	13.8	1.7	(10.4–17.2)
St. Cloud, Minnesota	535	16.5	2.0	(12.5–20.4)
St. Louis, Missouri-Illinois	1,845	17.5	1.5	(14.5–20.4)
Salisbury, Maryland-Delaware	1,896	21.6	1.9	(17.9–25.3)
Salt Lake City, Utah	5,197	10.9	0.5	(9.9–12.0)
San Antonio-New Braunfels, Texas	2,169	13.2	1.0	(11.2–15.2)
San Juan-Carolina-Caguas, Puerto Rico	3,717	11.6	0.7	(10.1–13.0)
Scottsbluff, Nebraska	879	21.6	2.0	(17.7–25.5)
Seattle-Bellevue-Everett, Washington^§^	3,555	12.8	0.9	(11.1–14.5)
Shreveport-Bossier City, Louisiana	528	25.6	2.6	(20.5–30.7)
Silver Spring-Frederick-Rockville, Maryland^§^	2,314	8.4	1.1	(6.1–10.6)
Sioux City, Iowa-Nebraska-South Dakota	1,109	20.5	2.5	(15.6–25.3)
Sioux Falls, South Dakota	1,314	17.2	1.8	(13.8–20.7)
Spartanburg, South Carolina	538	28.8	3.3	(22.3–35.3)
Spokane-Spokane Valley, Washington	708	19.8	2.4	(15.2–24.5)
Springfield, Massachusetts	1,026	20.3	2.2	(16.0–24.5)
Tampa-St. Petersburg-Clearwater, Florida	1,497	22.8	1.8	(19.2–26.4)
Toledo, Ohio	635	18.2	2.6	(13.1–23.2)
Topeka, Kansas	1,390	21.9	1.6	(18.8–24.9)
Tulsa, Oklahoma	1,972	18.3	1.2	(16.0–20.7)
Tuscaloosa, Alabama	697	21.5	2.2	(17.3–25.8)
Virginia Beach-Norfolk-Newport News, Virginia-North Carolina	1,805	22.1	1.5	(19.1–25.1)
Warren-Troy-Farmington Hills, Michigan^§^	2,063	21.5	1.4	(18.7–24.3)
Washington-Arlington-Alexandria, District of Columbia-Virginia-Maryland-West Virginia^§^	7,907	13.4	0.7	(12.0–14.8)
Wichita, Kansas	2,621	19.3	1.0	(17.3–21.2)
Wichita Falls, Texas	513	13.0	2.4	(8.3–17.7)
Wilmington, Delaware-Maryland-New Jersey^§^	2,660	18.7	1.3	(16.2–21.2)
Worcester, Massachusetts-Connecticut	2,293	18.1	1.4	(15.4–20.8)
Youngstown-Warren-Boardman, Ohio-Pennsylvania	503	27.5	3.7	(20.1–34.8)
*Median*		*18.6*		
*Range*		*5.0–29.3*		

**Abbreviations:** CI = confidence interval; MMSA = metropolitan and micropolitan statistical areas; SE = standard error.

* Age adjusted to the 2000 U.S. standard population.

^†^ Current smoking is defined as having smoked at least 100 cigarettes and smoking daily or some days during the period of the survey.

^§^ Metropolitan division.

#### Binge Drinking

Respondents were considered to be binge drinkers if during the past 30 days a man had five or more drinks on one occasion and a woman had four or more drinks on one occasion. In 2013, age-adjusted prevalence estimates of binge drinking among both men and women ranged from 10.5% in Tennessee to 25.2% in North Dakota (median: 17.7%) (Table 35). Among selected MMSA, age-adjusted prevalence estimates ranged from 7.2% in Provo-Orem, Utah, to 25.3% in Buffalo-Cheektowaga-Niagara Falls, New York (median: 17.8%) (Table 36). In 2014, age-adjusted prevalence estimates ranged from 10.7% in West Virginia to 25.1% in North Dakota (median: 17.0%) (Table 37). Among selected MMSA, age-adjusted prevalence estimates ranged from 6.7% in Provo-Orem, Utah, to 26.3% in Fargo, North Dakota-Minnesota (median: 17.0%) (Table 38).

**TABLE 35:** Age-adjusted* prevalence estimates of adults aged ≥18 years who reported binge drinking^†^ during the past 30 days, by state/territory — Behavioral Risk Factor Surveillance System, United States, 2013

State/Territory	Sample size	%	SE	95% CI
Alabama	6,263	11.8	0.7	(10.5–13.2)
Alaska	4,390	19.6	0.9	(17.8–21.3)
Arizona	4,047	14.0	1.1	(11.8–16.1)
Arkansas	5,042	13.4	0.8	(11.8–15.1)
California	9,945	17.9	0.6	(16.8–19.0)
Colorado	12,602	19.0	0.5	(18.0–20.0)
Connecticut	7,354	19.8	0.8	(18.2–21.3)
Delaware	4,972	18.2	0.8	(16.6–19.9)
District of Columbia	4,591	21.6	1.0	(19.5–23.6)
Florida	32,367	17.2	0.6	(16.1–18.3)
Georgia	7,612	13.3	0.6	(12.2–14.4)
Hawaii	7,584	19.4	0.7	(18.0–20.8)
Idaho	5,426	15.4	0.8	(13.8–17.0)
Illinois	5,462	22.6	0.9	(20.8–24.4)
Indiana	9,910	16.0	0.5	(14.9–17.0)
Iowa	7,903	23.4	0.7	(22.0–24.9)
Kansas	22,508	16.2	0.3	(15.5–16.8)
Kentucky	10,350	14.4	0.6	(13.2–15.5)
Louisiana	5,032	16.9	1.0	(14.9–18.8)
Maine	7,849	19.6	0.7	(18.2–21.0)
Maryland	12,363	14.9	0.5	(13.8–15.9)
Massachusetts	14,040	20.8	0.6	(19.6–22.0)
Michigan	12,368	19.9	0.6	(18.8–21.0)
Minnesota	13,576	21.9	0.6	(20.6–23.1)
Mississippi	7,113	12.9	0.7	(11.6–14.2)
Missouri	6,898	18.3	0.8	(16.7–19.9)
Montana	9,362	22.6	0.7	(21.3–23.9)
Nebraska	16,500	21.1	0.6	(20.0–22.2)
Nevada	4,881	15.7	1.0	(13.7–17.7)
New Hampshire	6,165	18.4	0.8	(16.8–19.9)
New Jersey	12,293	17.4	0.5	(16.4–18.5)
New Mexico	8,914	15.3	0.6	(14.1–16.5)
New York	8,471	18.3	0.6	(17.1–19.5)
North Carolina	8,470	13.8	0.6	(12.7–14.9)
North Dakota	7,504	25.2	0.8	(23.6–26.7)
Ohio	11,350	18.4	0.6	(17.2–19.6)
Oklahoma	8,027	13.4	0.6	(12.2–14.5)
Oregon	5,661	17.6	0.7	(16.1–19.0)
Pennsylvania	10,921	18.8	0.5	(17.7–19.8)
Rhode Island	6,133	19.1	0.8	(17.5–20.7)
South Carolina	10,300	15.9	0.6	(14.8–17.1)
South Dakota	6,640	20.6	0.9	(18.9–22.4)
Tennessee	5,334	10.5	0.7	(9.1–11.8)
Texas	10,353	16.7	0.6	(15.6–17.9)
Utah	12,354	11.9	0.4	(11.1–12.6)
Vermont	6,166	19.1	0.8	(17.5–20.7)
Virginia	7,961	16.8	0.6	(15.5–18.0)
Washington	10,889	17.5	0.5	(16.4–18.5)
West Virginia	5,765	12.4	0.6	(11.2–13.7)
Wisconsin	6,160	24.2	0.9	(22.4–26.0)
Wyoming	6,228	17.7	0.8	(16.0–19.3)
Guam	1,787	20.5	1.3	(18.0–23.0)
Puerto Rico	5,884	15.6	0.7	(14.2–17.0)
*Median*		*17.7*		
*Range*		*10.5–25.2*		

**Abbreviations:** CI = confidence interval; SE = standard error.

* Age adjusted to the 2000 U.S. standard population.

^†^ For men, having five or more drinks on at least one occasion; for women, having four or more drinks on at least one occasion.

**TABLE 36:** Age-adjusted* prevalence estimates of adults aged ≥18 years who reported binge drinking^†^ during the past 30 days, by metropolitan and micropolitan statistical area — Behavioral Risk Factor Surveillance System, United States, 2013

MMSA	Sample size	%	SE	95% CI
Aguadilla-Isabela, Puerto Rico	583	14.1	2.4	(9.4–18.9)
Akron, Ohio	657	20.4	2.7	(15.2–25.7)
Albuquerque, New Mexico	1,994	15.5	1.1	(13.3–17.8)
Allentown-Bethlehem-Easton, Pennsylvania-New Jersey	968	18.8	2.0	(14.8–22.7)
Anchorage, Alaska	1,463	18.0	1.4	(15.4–20.7)
Atlanta-Sandy Springs-Roswell, Georgia	3,288	13.8	0.9	(12.1–15.5)
Augusta-Richmond County, Georgia-South Carolina	866	18.9	2.4	(14.3–23.6)
Austin-Round Rock, Texas	885	22.4	2.0	(18.6–26.3)
Baltimore-Columbia-Towson, Maryland	4,516	16.3	0.9	(14.5–18.1)
Baton Rouge, Louisiana	889	20.7	2.3	(16.1–25.3)
Billings, Montana	786	21.6	1.7	(18.2–24.9)
Birmingham-Hoover, Alabama	1,303	14.4	1.5	(11.5–17.4)
Bismarck, North Dakota	990	25.1	1.9	(21.3–28.8)
Boise City, Idaho	1,434	16.9	1.5	(14.0–19.7)
Boston, Massachusetts^§^	3,789	22.9	1.1	(20.7–25.2)
Buffalo-Cheektowaga-Niagara Falls, New York	484	25.3	2.9	(19.6–30.9)
Burlington-South Burlington, Vermont	1,577	20.0	1.4	(17.3–22.8)
Cambridge-Newton-Framingham, Massachusetts^§^	4,544	20.6	1.0	(18.5–22.6)
Camden, New Jersey^§^	1,730	18.4	1.4	(15.8–21.1)
Cedar Rapids, Iowa	629	21.8	2.4	(17.0–26.6)
Charleston, West Virginia	798	15.7	1.8	(12.1–19.3)
Charleston-North Charleston, South Carolina	1,486	20.2	1.5	(17.3–23.1)
Charlotte-Concord-Gastonia, North Carolina-South Carolina	1,869	14.9	1.2	(12.6–17.2)
Chattanooga, Tennessee-Georgia	524	9.6	2.3	(5.0–14.1)
Chicago-Naperville-Elgin, Illinois-Indiana-Wisconsin	3,227	23.5	1.2	(21.1–25.8)
Cincinnati, Ohio-Kentucky-Indiana	2,466	19.5	1.4	(16.9–22.2)
Claremont-Lebanon, New Hampshire-Vermont	1,620	15.2	1.8	(11.7–18.6)
Cleveland-Elyria, Ohio	1,033	17.2	1.8	(13.7–20.8)
Colorado Springs, Colorado	1,282	15.2	1.4	(12.5–17.8)
Columbia, South Carolina	1,403	18.5	1.6	(15.5–21.6)
Columbus, Ohio	1,766	18.4	1.2	(16.1–20.8)
Crestview-Fort Walton Beach-Destin, Florida	1,022	19.0	2.0	(15.1–23.0)
Dallas-Plano-Irving, Texas^§^	857	17.4	1.7	(14.1–20.7)
Davenport-Moline-Rock Island, Iowa-Illinois	659	22.8	2.7	(17.6–28.0)
Dayton, Ohio	797	17.7	2.1	(13.5–21.9)
Deltona-Daytona Beach-Ormond Beach, Florida	1,052	18.2	2.4	(13.4–23.0)
Denver-Aurora-Lakewood, Colorado	5,281	20.2	0.7	(18.8–21.7)
Des Moines-West Des Moines, Iowa	1,310	20.0	1.6	(16.9–23.2)
Duluth, Minnesota-Wisconsin	677	21.8	2.8	(16.2–27.3)
Durham-Chapel Hill, North Carolina	596	14.8	2.2	(10.4–19.2)
El Paso, Texas	713	15.1	2.0	(11.1–19.1)
Evansville, Indiana-Kentucky	557	16.2	2.6	(11.1–21.2)
Fargo, North Dakota-Minnesota	1,147	24.8	1.8	(21.2–28.3)
Fayetteville-Springdale-Rogers, Arkansas-Missouri	787	18.0	2.3	(13.5–22.4)
Fort Smith, Arkansas-Oklahoma	485	12.6	2.4	(8.0–17.3)
Fort Wayne, Indiana	748	15.2	2.0	(11.4–19.1)
Fort Worth-Arlington, Texas^§^	782	14.7	1.9	(11.1–18.4)
Gainesville, Florida	993	17.2	2.3	(12.6–21.8)
Grand Forks, North Dakota-Minnesota	484	21.0	3.0	(15.1–26.9)
Grand Island, Nebraska	773	20.2	2.4	(15.5–24.9)
Grand Rapids-Wyoming, Michigan	1,313	20.3	1.5	(17.4–23.2)
Greensboro-High Point, North Carolina	636	12.0	1.7	(8.6–15.4)
Greenville-Anderson-Mauldin, South Carolina	1,284	14.3	1.4	(11.5–17.1)
Gulfport-Biloxi-Pascagoula, Mississippi	733	15.5	1.8	(11.9–19.1)
Hagerstown-Martinsburg, Maryland-West Virginia	736	13.3	2.6	(8.3–18.3)
Hartford-West Hartford-East Hartford, Connecticut	2,714	19.1	1.3	(16.5–21.7)
Hilton Head Island-Bluffton-Beaufort, South Carolina	777	20.9	2.8	(15.4–26.4)
Houston-The Woodlands-Sugar Land, Texas	1,302	15.5	1.4	(12.8–18.3)
Huntington-Ashland, West Virginia-Kentucky-Ohio	1,136	12.8	1.5	(10.0–15.7)
Idaho Falls, Idaho	492	8.5	1.7	(5.1–11.8)
Indianapolis-Carmel-Anderson, Indiana	2,430	15.0	1.0	(13.0–16.9)
Jackson, Mississippi	759	11.9	1.7	(8.5–15.3)
Jacksonville, Florida	2,737	16.6	1.2	(14.3–19.0)
Kansas City, Missouri-Kansas	7,178	17.8	1.0	(15.9–19.8)
Kingsport-Bristol-Bristol, Tennessee-Virginia	507	12.4	2.9	(6.8–18.1)
Knoxville, Tennessee	603	9.3	1.6	(6.1–12.4)
Lansing-East Lansing, Michigan	668	24.6	2.3	(20.1–29.1)
Lexington-Fayette, Kentucky	608	16.3	1.8	(12.8–19.8)
Lincoln, Nebraska	1,807	23.0	1.2	(20.5–25.4)
Little Rock-North Little Rock-Conway, Arkansas	1,099	14.0	1.6	(10.8–17.2)
Logan, Utah-Idaho	622	9.0	1.4	(6.3–11.8)
Los Angeles-Long Beach-Anaheim, California	2,573	17.2	1.0	(15.1–19.2)
Louisville-Jefferson County, Kentucky-Indiana	2,029	18.1	1.4	(15.3–20.8)
Lubbock, Texas	514	18.5	2.8	(13.0–24.1)
Manhattan, Kansas	642	22.1	2.0	(18.1–26.0)
Memphis, Tennessee-Mississippi-Arkansas	1,125	11.8	1.5	(8.8–14.7)
Miami-Fort Lauderdale-West Palm Beach, Florida	2,077	18.0	1.5	(15.2–20.9)
Milwaukee-Waukesha-West Allis, Wisconsin	1,170	21.6	2.0	(17.6–25.6)
Minneapolis-St. Paul-Bloomington, Minnesota-Wisconsin	8,611	22.6	0.9	(20.9–24.4)
Minot, North Dakota	626	24.7	2.3	(20.2–29.1)
Montgomery County-Bucks County-Chester County, Pennsylvania^§^	930	22.6	1.8	(19.1–26.2)
Myrtle Beach-Conway-North Myrtle Beach, South Carolina-North Carolina	746	16.7	2.2	(12.4–21.0)
Nashville-Davidson County-Murfreesboro-Franklin, Tennessee	985	13.3	1.4	(10.5–16.0)
Nassau County-Suffolk County, New York^§^	879	18.7	1.9	(14.9–22.5)
Newark, New Jersey-Pennsylvania^§^	3,768	16.8	0.9	(15.0–18.7)
New Orleans-Metairie, Louisiana	1,230	19.6	2.2	(15.3–23.9)
New York-Jersey City-White Plains, New York-New Jersey^§^	8,295	17.5	0.6	(16.3–18.8)
Norfolk, Nebraska	641	19.3	2.0	(15.4–23.1)
North Platte, Nebraska	689	18.9	2.3	(14.4–23.4)
North Port-Sarasota-Bradenton, Florida	1,015	20.3	2.2	(16.0–24.5)
Oakland-Hayward-Berkeley, California^§^	620	16.2	2.1	(12.2–20.3)
Ogden-Clearfield, Utah	2,397	12.0	0.9	(10.3–13.7)
Oklahoma City, Oklahoma	2,581	14.7	1.1	(12.6–16.8)
Omaha-Council Bluffs, Nebraska-Iowa	3,016	20.9	1.0	(18.9–23.0)
Orlando-Kissimmee-Sanford, Florida	2,154	15.4	1.3	(12.9–17.8)
Panama City, Florida	978	19.2	2.1	(15.0–23.3)
Pensacola-Ferry Pass-Brent, Florida	1,245	14.7	1.4	(11.9–17.4)
Philadelphia, Pennsylvania^§^	1,678	19.2	1.4	(16.5–21.9)
Phoenix-Mesa-Scottsdale, Arizona	1,474	14.4	1.5	(11.4–17.4)
Pittsburgh, Pennsylvania	2,248	21.5	1.3	(19.0–24.1)
Ponce, Puerto Rico	521	14.2	2.2	(9.9–18.5)
Portland-South Portland, Maine	2,549	21.6	1.3	(19.1–24.1)
Portland-Vancouver-Hillsboro, Oregon-Washington	3,118	16.9	0.9	(15.1–18.7)
Port St. Lucie, Florida	956	15.9	2.3	(11.3–20.4)
Providence-Warwick, Rhode Island-Massachusetts	7,795	19.5	1.0	(17.6–21.4)
Provo-Orem, Utah	1,806	7.2	0.7	(5.7–8.6)
Raleigh, North Carolina	640	16.6	1.6	(13.4–19.8)
Rapid City, South Dakota	841	20.0	2.0	(16.1–23.8)
Reno, Nevada	1,756	19.9	1.3	(17.3–22.4)
Richmond, Virginia	1,224	20.6	1.6	(17.4–23.8)
Riverside-San Bernardino-Ontario, California	1,194	16.4	1.4	(13.7–19.2)
Rochester, New York	492	15.1	2.2	(10.7–19.5)
Rockingham County-Strafford County, New Hampshire^§^	1,585	20.0	1.5	(17.0–23.0)
Sacramento-Roseville-Arden-Arcade, California	794	19.5	2.0	(15.6–23.4)
St. Louis, Missouri-Illinois	1,991	22.1	1.6	(19.0–25.1)
Salem, Oregon	497	15.3	2.7	(10.1–20.5)
Salina, Kansas	510	13.8	2.2	(9.6–18.1)
Salisbury, Maryland-Delaware	1,956	15.4	1.5	(12.5–18.4)
Salt Lake City, Utah	4,480	14.2	0.7	(12.8–15.5)
San Antonio-New Braunfels, Texas	902	19.7	2.0	(15.9–23.6)
San Francisco-Redwood City-South San Francisco, California^§^	461	23.9	2.8	(18.5–29.4)
San Jose-Sunnyvale-Santa Clara, California	533	15.9	2.0	(12.0–19.8)
San Juan-Carolina-Caguas, Puerto Rico	3,568	16.4	0.9	(14.6–18.1)
Scottsbluff, Nebraska	691	15.7	2.3	(11.3–20.2)
Scranton-Wilkes-Barre-Hazleton, Pennsylvania	543	23.8	2.9	(18.1–29.4)
Seattle-Bellevue-Everett, Washington^§^	3,667	18.3	0.9	(16.6–20.0)
Shreveport-Bossier City, Louisiana	551	17.8	3.1	(11.6–23.9)
Silver Spring-Frederick-Rockville, Maryland^§^	2,314	14.8	1.1	(12.6–16.9)
Sioux City, Iowa-Nebraska-South Dakota	1,013	21.9	2.8	(16.5–27.3)
Sioux Falls, South Dakota	966	22.2	1.9	(18.5–26.0)
Spartanburg, South Carolina	571	8.8	2.0	(4.9–12.7)
Spokane-Spokane Valley, Washington	844	17.8	2.0	(14.0–21.7)
Springfield, Massachusetts	1,466	18.5	2.0	(14.6–22.5)
Tallahassee, Florida	1,771	17.4	1.9	(13.6–21.1)
Tampa-St. Petersburg-Clearwater, Florida	2,088	17.1	1.3	(14.6–19.5)
Toledo, Ohio	951	20.1	2.2	(15.9–24.4)
Topeka, Kansas	2,325	14.7	1.1	(12.6–16.9)
Tulsa, Oklahoma	1,948	12.5	1.1	(10.2–14.7)
Virginia Beach-Norfolk-Newport News, Virginia-North Carolina	1,572	17.5	1.4	(14.7–20.2)
Warren-Troy-Farmington Hills, Michigan^§^	2,181	21.1	1.3	(18.7–23.6)
Washington-Arlington-Alexandria, District of Columbia-Virginia-Maryland-West Virginia^§^	8,483	16.5	0.8	(14.9–18.1)
Wichita, Kansas	4,769	15.5	0.7	(14.1–17.0)
Wilmington, Delaware-Maryland-New Jersey^§^	3,132	17.9	1.0	(15.9–19.9)
Winston-Salem, North Carolina	661	12.2	2.1	(8.1–16.3)
Worcester, Massachusetts-Connecticut	2,614	20.0	1.4	(17.3–22.7)
*Median*		*17.8*		
*Range*		*7.2–25.3*		

**Abbreviations:** CI = confidence interval; MMSA = metropolitan and micropolitan statistical areas; SE = standard error.

* Age adjusted to the 2000 U.S. standard population.

^†^ For men, having five or more drinks on one occasion; for women, having four or more drinks on one occasion.

^§^ Metropolitan division.

**TABLE 37:** Age-adjusted* prevalence estimates of adults aged ≥18 years who reported binge drinking^†^ during the past 30 days, by state/territory — Behavioral Risk Factor Surveillance System, United States, 2014

State/Territory	Sample size	%	SE	95% CI
Alabama	8,230	12.9	0.6	(11.6–14.1)
Alaska	4,134	20.0	0.9	(18.2–21.7)
Arizona	13,743	15.7	0.6	(14.5–17.0)
Arkansas	4,846	14.3	1.0	(12.4–16.2)
California	7,279	16.0	0.6	(14.9–17.1)
Colorado	12,113	17.9	0.5	(17.0–18.9)
Connecticut	7,305	17.4	0.7	(16.0–18.9)
Delaware	4,101	17.0	1.0	(15.0–19.0)
District of Columbia	3,750	24.1	1.2	(21.6–26.5)
Florida	8,965	16.5	0.6	(15.3–17.8)
Georgia	5,854	14.6	0.7	(13.1–16.0)
Hawaii	6,812	21.0	0.8	(19.5–22.5)
Idaho	5,178	15.5	0.9	(13.8–17.2)
Illinois	4,797	21.0	0.9	(19.3–22.7)
Indiana	10,804	15.6	0.5	(14.5–16.6)
Iowa	7,655	23.1	0.7	(21.6–24.5)
Kansas	12,989	16.8	0.5	(15.9–17.7)
Kentucky	10,428	13.6	0.7	(12.3–14.9)
Louisiana	6,365	16.5	0.7	(15.2–17.8)
Maine	8,705	19.6	0.7	(18.2–21.1)
Maryland	11,829	16.2	0.7	(14.8–17.6)
Massachusetts	14,343	18.7	0.6	(17.5–19.8)
Michigan	8,162	20.3	0.7	(19.0–21.6)
Minnesota	15,544	20.5	0.4	(19.7–21.4)
Mississippi	3,993	13.6	0.9	(11.9–15.4)
Missouri	6,765	16.1	0.8	(14.6–17.6)
Montana	7,164	20.8	0.8	(19.1–22.4)
Nebraska	21,443	21.5	0.5	(20.5–22.5)
Nevada	3,558	16.7	1.1	(14.6–18.9)
New Hampshire	5,762	18.8	0.9	(16.9–20.6)
New Jersey	11,968	17.0	0.6	(15.9–18.2)
New Mexico	8,238	14.6	0.7	(13.2–16.0)
New York	6,263	15.8	0.6	(14.6–17.1)
North Carolina	6,797	14.5	0.6	(13.3–15.7)
North Dakota	7,353	25.1	0.9	(23.2–26.9)
Ohio	10,499	19.6	0.7	(18.2–21.0)
Oklahoma	8,076	13.6	0.6	(12.5–14.8)
Oregon	4,893	17.9	0.8	(16.3–19.5)
Pennsylvania	10,315	18.3	0.6	(17.0–19.5)
Rhode Island	5,952	19.6	0.9	(17.8–21.4)
South Carolina	10,468	15.1	0.6	(14.0–16.2)
South Dakota	7,032	18.5	0.9	(16.8–20.3)
Tennessee	4,766	11.4	0.8	(9.7–13.0)
Texas	14,193	16.4	0.5	(15.4–17.5)
Utah	14,350	11.1	0.3	(10.4–11.7)
Vermont	6,078	20.1	0.7	(18.7–21.6)
Virginia	8,959	16.0	0.6	(14.9–17.2)
Washington	9,656	18.0	0.6	(16.8–19.2)
West Virginia	6,002	10.7	0.6	(9.5–11.8)
Wisconsin	6,641	23.2	0.8	(21.6–24.8)
Wyoming	5,965	18.1	1.1	(16.0–20.2)
Guam	2,326	22.2	1.2	(19.8–24.6)
Puerto Rico	5,830	14.1	0.6	(12.8–15.3)
*Median*		*17.0*		
*Range*		*10.7–25.1*		

**Abbreviations:** CI = confidence interval; SE = standard error.

* Age adjusted to the 2000 U.S. standard population.

^†^ For men, having five or more drinks on one occasion; for women, having four or more drinks on one occasion.

**TABLE 38:** Age-adjusted* prevalence estimates of adults aged ≥18 years who reported binge drinking^†^ during the past 30 days, by metropolitan and micropolitan statistical area — Behavioral Risk Factor Surveillance System, United States, 2014

MMSA	Sample size	%	SE	95% CI
Aberdeen, South Dakota	587	21.3	2.9	(15.5–27.0)
Aguadilla-Isabela, Puerto Rico	537	11.1	1.9	(7.3–14.9)
Albuquerque, New Mexico	1,638	13.5	1.3	(11.0–15.9)
Allentown-Bethlehem-Easton, Pennsylvania-New Jersey	1,027	17.2	2.3	(12.7–21.7)
Anchorage, Alaska	1,685	19.4	1.3	(16.9–21.9)
Atlanta-Sandy Springs-Roswell, Georgia	2,557	15.7	1.0	(13.7–17.7)
Augusta-Richmond County, Georgia-South Carolina	827	11.6	2.3	(7.2–16.1)
Austin-Round Rock, Texas	2,099	20.4	1.3	(17.9–22.9)
Baltimore-Columbia-Towson, Maryland	4,320	17.3	1.1	(15.1–19.6)
Baton Rouge, Louisiana	880	17.9	1.8	(14.5–21.4)
Berlin, New Hampshire-Vermont	510	16.1	3.3	(9.7–22.5)
Billings, Montana	764	20.7	2.2	(16.4–24.9)
Birmingham-Hoover, Alabama	1,494	13.7	1.3	(11.1–16.3)
Bismarck, North Dakota	977	21.6	2.2	(17.3–25.9)
Boise City, Idaho	1,274	14.3	1.6	(11.1–17.5)
Boston, Massachusetts^§^	4,149	19.4	1.1	(17.3–21.5)
Burlington-South Burlington, Vermont	1,867	22.2	1.3	(19.7–24.7)
Cambridge-Newton-Framingham, Massachusetts^§^	4,762	18.5	0.9	(16.6–20.3)
Camden, New Jersey^§^	1,592	18.5	1.7	(15.3–21.8)
Cedar Rapids, Iowa	610	20.9	2.4	(16.2–25.5)
Charleston, West Virginia	849	10.8	1.5	(7.9–13.8)
Charleston-North Charleston, South Carolina	1,325	19.7	1.6	(16.6–22.7)
Charlotte-Concord-Gastonia, North Carolina-South Carolina	2,007	14.8	1.1	(12.6–17.0)
Chicago-Naperville-Elgin, Illinois-Indiana-Wisconsin	3,884	21.0	1.0	(19.0–23.0)
Cincinnati, Ohio-Kentucky-Indiana	1,935	21.1	1.8	(17.5–24.6)
Claremont-Lebanon, New Hampshire-Vermont	1,609	16.6	1.6	(13.4–19.8)
Cleveland-Elyria, Ohio	927	24.4	2.2	(20.1–28.7)
College Station-Bryan, Texas	549	12.4	2.5	(7.5–17.3)
Colorado Springs, Colorado	1,175	12.2	1.3	(9.7–14.7)
Columbia, South Carolina	1,155	17.0	1.6	(13.9–20.0)
Columbus, Ohio	1,581	18.9	1.6	(15.8–22.0)
Corpus Christi, Texas	570	16.7	3.6	(9.7–23.7)
Dallas-Plano-Irving, Texas^§^	1,192	18.2	1.8	(14.7–21.7)
Dayton, Ohio	550	16.7	2.5	(11.9–21.6)
Denver-Aurora-Lakewood, Colorado	5,229	19.4	0.7	(18.0–20.8)
Des Moines-West Des Moines, Iowa	1,266	21.2	1.8	(17.7–24.8)
Duluth, Minnesota-Wisconsin	895	22.2	2.0	(18.3–26.2)
El Paso, Texas	655	18.9	2.4	(14.2–23.7)
Evansville, Indiana-Kentucky	615	16.2	3.0	(10.3–22.1)
Fargo, North Dakota-Minnesota	1,092	26.3	2.0	(22.4–30.1)
Fayetteville-Springdale-Rogers, Arkansas-Missouri	766	15.2	2.3	(10.7–19.8)
Fort Wayne, Indiana	809	14.0	2.0	(10.1–17.9)
Fort Worth-Arlington, Texas^§^	705	13.2	2.0	(9.3–17.2)
Grand Island, Nebraska	1,003	18.5	2.2	(14.2–22.7)
Grand Rapids-Wyoming, Michigan	869	21.1	2.0	(17.1–25.1)
Greensboro-High Point, North Carolina	482	15.2	2.3	(10.6–19.7)
Greenville-Anderson-Mauldin, South Carolina	1,421	11.9	1.1	(9.7–14.2)
Hagerstown-Martinsburg, Maryland-West Virginia	748	15.8	3.2	(9.6–22.0)
Hartford-West Hartford-East Hartford, Connecticut	2,443	17.9	1.2	(15.5–20.4)
Hilton Head Island-Bluffton-Beaufort, South Carolina	514	18.3	2.9	(12.6–24.0)
Houston-The Woodlands-Sugar Land, Texas	1,963	15.3	1.3	(12.8–17.9)
Huntington-Ashland, West Virginia-Kentucky-Ohio	1,202	10.2	1.5	(7.3–13.1)
Idaho Falls, Idaho	494	12.4	2.1	(8.2–16.5)
Indianapolis-Carmel-Anderson, Indiana	3,390	14.7	0.9	(12.9–16.6)
Jacksonville, Florida	619	17.2	2.4	(12.5–22.0)
Kansas City, Missouri-Kansas	4,640	16.4	1.0	(14.4–18.4)
Kingsport-Bristol-Bristol, Tennessee-Virginia	477	10.3	2.6	(5.2–15.4)
Knoxville, Tennessee	517	7.2	1.8	(3.8–10.7)
Lafayette, Louisiana	523	17.9	2.1	(13.8–22.0)
Lexington-Fayette, Kentucky	585	11.9	1.8	(8.4–15.4)
Lincoln, Nebraska	1,937	23.2	1.3	(20.6–25.8)
Little Rock-North Little Rock-Conway, Arkansas	1,100	13.8	1.7	(10.4–17.2)
Logan, Utah-Idaho	602	6.9	1.2	(4.5–9.3)
Los Angeles-Long Beach-Anaheim, California	1,943	15.0	1.0	(13.0–16.9)
Louisville-Jefferson County, Kentucky-Indiana	2,281	16.7	1.5	(13.7–19.6)
Madison, Wisconsin	518	23.8	2.3	(19.2–28.3)
Memphis, Tennessee-Mississippi-Arkansas	813	9.4	1.7	(6.1–12.7)
Miami-Fort Lauderdale-West Palm Beach, Florida	1,963	14.2	1.2	(11.8–16.7)
Milwaukee-Waukesha-West Allis, Wisconsin	1,267	23.2	1.9	(19.5–26.8)
Minneapolis-St. Paul-Bloomington, Minnesota-Wisconsin	8,309	20.0	0.6	(18.9–21.1)
Minot, North Dakota	569	20.6	2.6	(15.5–25.7)
Montgomery, Alabama	486	16.2	2.7	(10.9–21.5)
Montgomery County-Bucks County-Chester County, Pennsylvania^§^	768	21.3	2.3	(16.8–25.8)
Myrtle Beach-Conway-North Myrtle Beach, South Carolina-North Carolina	945	16.4	1.9	(12.6–20.2)
Nashville-Davidson County-Murfreesboro-Franklin, Tennessee	734	17.0	2.3	(12.4–21.6)
Nassau County-Suffolk County, New York^§^	709	17.9	2.2	(13.5–22.3)
Newark, New Jersey-Pennsylvania^§^	3,818	16.4	1.0	(14.4–18.5)
New Orleans-Metairie, Louisiana	1,800	18.4	1.3	(15.9–20.9)
New York-Jersey City-White Plains, New York-New Jersey^§^	6,813	15.7	0.7	(14.4–17.0)
Norfolk, Nebraska	940	24.4	1.9	(20.7–28.0)
North Platte, Nebraska	913	19.3	1.9	(15.6–23.0)
North Port-Sarasota-Bradenton, Florida	472	11.1	2.5	(6.2–15.9)
Oakland-Hayward-Berkeley, California^§^	596	15.3	2.1	(11.2–19.4)
Ogden-Clearfield, Utah	2,814	10.1	0.7	(8.7–11.4)
Oklahoma City, Oklahoma	2,326	13.9	1.0	(11.9–15.9)
Omaha-Council Bluffs, Nebraska-Iowa	4,667	20.8	0.9	(19.0–22.6)
Orlando-Kissimmee-Sanford, Florida	876	14.8	1.8	(11.4–18.3)
Philadelphia, Pennsylvania^§^	1,382	20.3	1.6	(17.1–23.5)
Phoenix-Mesa-Scottsdale, Arizona	8,689	16.2	0.7	(14.8–17.7)
Pittsburgh, Pennsylvania	2,260	20.6	1.3	(18.0–23.1)
Ponce, Puerto Rico	517	16.7	2.5	(11.8–21.6)
Portland-South Portland, Maine	2,625	20.5	1.3	(17.9–23.0)
Portland-Vancouver-Hillsboro, Oregon-Washington	2,666	16.8	1.0	(14.7–18.8)
Providence-Warwick, Rhode Island-Massachusetts	7,476	19.5	0.9	(17.7–21.4)
Provo-Orem, Utah	2,063	6.7	0.6	(5.5–8.0)
Raleigh, North Carolina	681	16.6	1.9	(12.9–20.3)
Rapid City, South Dakota	1,351	15.5	1.6	(12.4–18.6)
Reno, Nevada	1,154	21.7	1.8	(18.1–25.3)
Richmond, Virginia	1,397	16.5	1.5	(13.7–19.4)
Riverside-San Bernardino-Ontario, California	769	15.6	1.8	(12.1–19.0)
Roanoke, Virginia	496	16.9	2.7	(11.7–22.2)
Rochester, Minnesota	666	19.5	1.9	(15.8–23.1)
Rockingham County-Strafford County, New Hampshire^§^	1,337	20.1	1.8	(16.6–23.7)
Sacramento-Roseville-Arden-Arcade, California	545	17.9	2.3	(13.5–22.4)
St. Cloud, Minnesota	522	20.7	2.0	(16.7–24.7)
St. Louis, Missouri-Illinois	1,822	18.1	1.5	(15.2–21.0)
Salisbury, Maryland-Delaware	1,862	13.7	1.9	(10.0–17.4)
Salt Lake City, Utah	5,149	13.9	0.6	(12.7–15.0)
San Antonio-New Braunfels, Texas	2,107	17.3	1.2	(14.9–19.7)
San Juan-Carolina-Caguas, Puerto Rico	3,663	14.1	0.8	(12.6–15.7)
Scottsbluff, Nebraska	869	17.5	1.9	(13.8–21.3)
Seattle-Bellevue-Everett, Washington^§^	3,533	18.0	0.9	(16.1–19.8)
Shreveport-Bossier City, Louisiana	518	16.1	2.3	(11.6–20.5)
Silver Spring-Frederick-Rockville, Maryland^§^	2,261	14.9	1.5	(12.0–17.8)
Sioux City, Iowa-Nebraska-South Dakota	1,093	21.2	2.7	(16.0–26.5)
Sioux Falls, South Dakota	1,289	18.1	1.8	(14.6–21.5)
Spartanburg, South Carolina	534	11.1	2.2	(6.7–15.5)
Spokane-Spokane Valley, Washington	698	22.3	2.5	(17.4–27.2)
Springfield, Massachusetts	1,012	19.6	2.1	(15.6–23.7)
Tampa-St. Petersburg-Clearwater, Florida	1,459	20.5	1.8	(17.1–24.0)
Toledo, Ohio	624	18.8	2.5	(13.9–23.7)
Topeka, Kansas	1,375	16.9	1.4	(14.1–19.7)
Tulsa, Oklahoma	1,944	14.0	1.1	(11.8–16.3)
Tuscaloosa, Alabama	685	12.0	1.8	(8.5–15.4)
Virginia Beach-Norfolk-Newport News, Virginia-North Carolina	1,777	16.4	1.3	(13.8–19.0)
Warren-Troy-Farmington Hills, Michigan^§^	2,043	20.2	1.3	(17.7–22.7)
Washington-Arlington-Alexandria, District of Columbia-Virginia-Maryland-West Virginia^§ ^	7,724	16.6	0.8	(15.1–18.2)
Wichita, Kansas	2,586	14.4	0.9	(12.6–16.1)
Wichita Falls, Texas	507	12.5	3.4	(5.9–19.1)
Wilmington, Delaware-Maryland-New Jersey^§^	2,630	18.2	1.3	(15.6–20.7)
Worcester, Massachusetts-Connecticut	2,248	17.7	1.3	(15.1–20.3)
Youngstown-Warren-Boardman, Ohio-Pennsylvania	496	17.1	3.2	(10.9–23.3)
*Median*		*17.0*		
*Range*		*6.7–26.3*		

**Abbreviations:** CI = confidence interval; MMSA = metropolitan and micropolitan statistical areas; SE = standard error.

* Age adjusted to the 2000 U.S. standard population.

^†^ For men, having five or more drinks on one occasion; for women, having four or more drinks on one occasion.

^§^ Metropolitan division.

### Chronic Diseases and Conditions

#### Obesity

Obesity, calculated from self-reported height and weight, was defined as having a body mass index of ≥30 (weight [kg]/height [m^2^]). In 2013, age-adjusted prevalence estimates of obesity ranged from 21.0% in Colorado to 35.2% in West Virginia (median: 28.2%) (Table 39). Among selected MMSA, age-adjusted prevalence estimates ranged from 12.1% in San Francisco-Redwood City-South San Francisco, California, to 37.1% in Huntington-Ashland, West Virginia-Kentucky-Ohio (median: 28.3%) (Table 40). In 2014, age-adjusted prevalence estimates ranged from 21.0% in Colorado to 35.9% in Arkansas (median: 29.0%) (Table 41). Among selected MMSA, age-adjusted prevalence estimates ranged from 19.7% in Reno, Nevada, to 42.5% in Corpus Christi, Texas (median: 29.3%) (Table 42).

**TABLE 39:** Age-adjusted* prevalence estimates of adults aged ≥18 years with obesity,^†^ by state/territory — Behavioral Risk Factor Surveillance System, United States, 2013

State/Territory	Sample size	%	SE	95% CI
Alabama	6,244	32.0	0.9	(30.3–33.8)
Alaska	4,396	28.2	1.0	(26.2–30.1)
Arizona	4,029	26.6	1.4	(24.0–29.3)
Arkansas	5,023	34.9	1.1	(32.8–37.0)
California	10,669	23.8	0.6	(22.7–24.9)
Colorado	12,920	21.0	0.5	(20.1–22.0)
Connecticut	7,309	24.5	0.8	(22.9–26.0)
Delaware	4,893	30.4	0.9	(28.5–32.2)
District of Columbia	4,705	23.1	1.0	(21.2–25.0)
Florida	32,552	26.1	0.6	(24.9–27.2)
Georgia	7,606	29.9	0.7	(28.5–31.4)
Hawaii	7,609	21.9	0.7	(20.5–23.4)
Idaho	5,315	29.2	0.9	(27.4–31.0)
Illinois	5,490	28.9	0.9	(27.1–30.6)
Indiana	9,770	31.2	0.6	(30.0–32.5)
Iowa	7,672	30.8	0.7	(29.4–32.3)
Kansas	21,991	29.7	0.4	(28.9–30.4)
Kentucky	10,325	32.6	0.7	(31.2–34.1)
Louisiana	5,014	32.6	1.1	(30.5–34.8)
Maine	7,791	28.0	0.7	(26.5–29.4)
Maryland	12,169	27.6	0.7	(26.3–28.9)
Massachusetts	13,883	22.9	0.6	(21.8–24.1)
Michigan	12,177	30.8	0.6	(29.6–32.0)
Minnesota	13,450	24.9	0.7	(23.5–26.2)
Mississippi	7,032	34.8	0.9	(33.1–36.5)
Missouri	6,783	30.2	0.9	(28.4–31.9)
Montana	9,323	24.0	0.6	(22.8–25.3)
Nebraska	16,250	29.1	0.6	(27.9–30.3)
Nevada	4,871	26.1	1.2	(23.8–28.4)
New Hampshire	6,141	26.1	0.8	(24.5–27.7)
New Jersey	12,256	25.7	0.6	(24.5–26.9)
New Mexico	8,914	26.3	0.7	(24.9–27.7)
New York	8,465	24.8	0.6	(23.6–26.1)
North Carolina	8,292	28.9	0.7	(27.5–30.3)
North Dakota	7,413	30.5	0.8	(29.0–32.1)
Ohio	11,260	29.8	0.7	(28.5–31.1)
Oklahoma	7,875	32.2	0.7	(30.8–33.7)
Oregon	5,668	26.0	0.8	(24.4–27.7)
Pennsylvania	10,857	29.3	0.6	(28.1–30.6)
Rhode Island	6,191	27.0	0.8	(25.4–28.6)
South Carolina	10,227	31.5	0.7	(30.2–32.9)
South Dakota	6,531	29.4	1.0	(27.4–31.3)
Tennessee	5,452	33.4	1.0	(31.5–35.3)
Texas	10,085	30.6	0.7	(29.2–32.0)
Utah	12,027	24.4	0.5	(23.4–25.3)
Vermont	6,106	24.1	0.8	(22.6–25.6)
Virginia	7,941	26.7	0.7	(25.4–28.0)
Washington	10,554	26.6	0.6	(25.4–27.8)
West Virginia	5,636	35.2	0.8	(33.5–36.8)
Wisconsin	6,188	29.0	0.9	(27.2–30.8)
Wyoming	6,147	27.6	0.9	(25.8–29.3)
Guam	1,803	26.6	1.4	(23.9–29.4)
Puerto Rico	5,762	27.8	0.8	(26.2–29.3)
*Median*		*28.2*		
*Range*		*21.0–35.2*		

**Abbreviations:** CI = confidence interval; SE = standard error.

* Age adjusted to the 2000 U.S. standard population.

^†^ Body mass index ≥30.

**TABLE 40:** Age-adjusted* prevalence estimates of adults aged ≥18 years with obesity,^†^ by metropolitan and micropolitan statistical area — Behavioral Risk Factor Surveillance System, United States, 2013

MMSA	Sample size	%	SE	95% CI
Aguadilla-Isabela, Puerto Rico	569	24.4	2.3	(19.8–29.0)
Akron, Ohio	643	32.3	2.9	(26.6–38.0)
Albuquerque, New Mexico	1,994	23.6	1.3	(21.0–26.1)
Allentown-Bethlehem-Easton, Pennsylvania-New Jersey	960	28.6	2.3	(24.0–33.1)
Anchorage, Alaska	1,455	26.9	1.5	(23.9–29.9)
Atlanta-Sandy Springs-Roswell, Georgia	3,258	26.2	1.0	(24.2–28.2)
Augusta-Richmond County, Georgia-South Carolina	866	36.1	2.7	(30.8–41.5)
Austin-Round Rock, Texas	869	25.1	2.0	(21.1–29.1)
Baltimore-Columbia-Towson, Maryland	4,465	27.2	1.0	(25.3–29.2)
Baton Rouge, Louisiana	878	34.4	2.6	(29.4–39.5)
Billings, Montana	783	25.4	1.8	(21.9–28.8)
Birmingham-Hoover, Alabama	1,312	32.3	1.8	(28.8–35.7)
Bismarck, North Dakota	976	29.3	1.9	(25.6–33.1)
Boise City, Idaho	1,404	27.7	1.7	(24.4–31.0)
Boston, Massachusetts^§^	3,725	22.0	1.1	(19.8–24.1)
Buffalo-Cheektowaga-Niagara Falls, New York	487	23.3	2.4	(18.5–28.0)
Burlington-South Burlington, Vermont	1,562	21.9	1.3	(19.4–24.5)
Cambridge-Newton-Framingham, Massachusetts^§^	4,521	20.9	1.0	(19.0–22.8)
Camden, New Jersey^§^	1,726	28.1	1.5	(25.1–31.1)
Cedar Rapids, Iowa	609	34.6	2.7	(29.3–39.8)
Charleston, West Virginia	777	35.4	2.3	(30.9–39.8)
Charleston-North Charleston, South Carolina	1,494	27.2	1.6	(24.1–30.2)
Charlotte-Concord-Gastonia, North Carolina-South Carolina	1,823	28.3	1.4	(25.5–31.1)
Chattanooga, Tennessee-Georgia	535	25.7	2.7	(20.4–31.0)
Chicago-Naperville-Elgin, Illinois-Indiana-Wisconsin	3,231	28.7	1.2	(26.4–31.0)
Cincinnati, Ohio-Kentucky-Indiana	2,465	30.8	1.5	(28.0–33.7)
Claremont-Lebanon, New Hampshire-Vermont	1,618	25.3	1.9	(21.7–29.0)
Cleveland-Elyria, Ohio	1,034	27.1	1.9	(23.3–30.8)
Colorado Springs, Colorado	1,315	23.7	1.5	(20.7–26.6)
Columbia, South Carolina	1,380	31.8	1.8	(28.3–35.3)
Columbus, Ohio	1,752	31.4	1.5	(28.6–34.3)
Crestview-Fort Walton Beach-Destin, Florida	1,031	25.5	2.1	(21.4–29.7)
Dallas-Plano-Irving, Texas^§^	829	25.8	1.8	(22.2–29.4)
Davenport-Moline-Rock Island, Iowa-Illinois	646	32.6	2.8	(27.2–38.0)
Dayton, Ohio	795	31.0	2.4	(26.2–35.8)
Deltona-Daytona Beach-Ormond Beach, Florida	1,059	23.0	2.3	(18.5–27.6)
Denver-Aurora-Lakewood, Colorado	5,408	20.4	0.7	(19.0–21.7)
Des Moines-West Des Moines, Iowa	1,276	29.6	1.8	(26.1–33.1)
Duluth, Minnesota-Wisconsin	660	21.3	2.4	(16.5–26.0)
Durham-Chapel Hill, North Carolina	577	23.7	2.3	(19.1–28.3)
El Paso, Texas	692	30.4	2.3	(25.9–34.8)
Evansville, Indiana-Kentucky	545	33.9	3.1	(27.9–39.9)
Fargo, North Dakota-Minnesota	1,130	27.2	1.9	(23.5–30.9)
Fayetteville-Springdale-Rogers, Arkansas-Missouri	782	29.0	2.5	(24.1–33.9)
Fort Smith, Arkansas-Oklahoma	478	35.5	3.1	(29.4–41.7)
Fort Wayne, Indiana	742	25.2	2.0	(21.2–29.2)
Fort Worth-Arlington, Texas^§^	756	31.1	2.5	(26.3–36.0)
Gainesville, Florida	982	25.1	2.3	(20.6–29.6)
Grand Forks, North Dakota-Minnesota	476	29.4	3.3	(22.8–35.9)
Grand Island, Nebraska	756	35.5	2.8	(29.9–41.0)
Grand Rapids-Wyoming, Michigan	1,285	28.8	1.7	(25.5–32.1)
Greensboro-High Point, North Carolina	619	30.8	2.5	(26.0–35.7)
Greenville-Anderson-Mauldin, South Carolina	1,274	28.7	1.8	(25.3–32.2)
Gulfport-Biloxi-Pascagoula, Mississippi	734	33.3	2.4	(28.6–37.9)
Hagerstown-Martinsburg, Maryland-West Virginia	726	34.8	3.0	(28.9–40.8)
Hartford-West Hartford-East Hartford, Connecticut	2,686	26.8	1.4	(24.1–29.5)
Hilton Head Island-Bluffton-Beaufort, South Carolina	795	25.6	2.8	(20.0–31.1)
Houston-The Woodlands-Sugar Land, Texas	1,289	26.3	1.8	(22.8–29.7)
Huntington-Ashland, West Virginia-Kentucky-Ohio	1,102	37.1	1.9	(33.4–40.9)
Idaho Falls, Idaho	486	35.7	3.0	(29.7–41.6)
Indianapolis-Carmel-Anderson, Indiana	2,409	31.8	1.3	(29.3–34.2)
Jackson, Mississippi	757	31.1	2.3	(26.6–35.6)
Jacksonville, Florida	2,751	28.4	1.4	(25.7–31.1)
Kansas City, Missouri-Kansas	7,031	29.2	1.1	(27.0–31.3)
Kingsport-Bristol-Bristol, Tennessee-Virginia	509	29.3	3.2	(23.1–35.6)
Knoxville, Tennessee	616	31.3	2.6	(26.2–36.5)
Lansing-East Lansing, Michigan	651	27.2	2.1	(23.0–31.3)
Lexington-Fayette, Kentucky	610	32.9	2.5	(28.0–37.8)
Lincoln, Nebraska	1,785	25.5	1.2	(23.2–27.8)
Little Rock-North Little Rock-Conway, Arkansas	1,092	32.9	2.1	(28.8–37.0)
Logan, Utah-Idaho	608	23.1	2.1	(19.0–27.1)
Los Angeles-Long Beach-Anaheim, California	2,782	22.4	1.0	(20.4–24.5)
Louisville-Jefferson County, Kentucky-Indiana	2,021	29.5	1.6	(26.3–32.7)
Lubbock, Texas	500	33.2	3.2	(27.0–39.5)
Manhattan, Kansas	632	22.9	1.9	(19.1–26.7)
Memphis, Tennessee-Mississippi-Arkansas	1,141	32.4	2.1	(28.2–36.5)
Miami-Fort Lauderdale-West Palm Beach, Florida	2,107	23.6	1.5	(20.7–26.6)
Milwaukee-Waukesha-West Allis, Wisconsin	1,182	27.6	2.1	(23.5–31.7)
Minneapolis-St. Paul-Bloomington, Minnesota-Wisconsin	8,556	23.7	0.9	(21.9–25.4)
Minot, North Dakota	625	26.4	2.2	(22.2–30.7)
Montgomery County-Bucks County-Chester County, Pennsylvania^§^	937	22.7	1.7	(19.4–26.0)
Myrtle Beach-Conway-North Myrtle Beach, South Carolina-North Carolina	736	32.1	2.6	(27.0–37.2)
Nashville-Davidson County-Murfreesboro-Franklin, Tennessee	980	33.4	2.0	(29.4–37.4)
Nassau County-Suffolk County, New York^§^	888	24.3	1.9	(20.6–28.0)
Newark, New Jersey-Pennsylvania^§^	3,762	23.7	1.0	(21.7–25.7)
New Orleans-Metairie, Louisiana	1,236	30.2	2.1	(26.1–34.4)
New York-Jersey City-White Plains, New York-New Jersey^§^	8,284	23.7	0.7	(22.3–25.0)
Norfolk, Nebraska	623	34.8	2.3	(30.3–39.4)
North Platte, Nebraska	686	31.7	2.8	(26.2–37.1)
North Port-Sarasota-Bradenton, Florida	1,024	23.9	2.3	(19.4–28.5)
Oakland-Hayward-Berkeley, California^§^	657	19.5	2.0	(15.6–23.3)
Ogden-Clearfield, Utah	2,307	24.5	1.0	(22.5–26.6)
Oklahoma City, Oklahoma	2,510	29.4	1.2	(27.1–31.7)
Omaha-Council Bluffs, Nebraska-Iowa	2,972	30.2	1.2	(27.9–32.5)
Orlando-Kissimmee-Sanford, Florida	2,161	25.8	1.4	(23.0–28.6)
Panama City, Florida	978	28.5	2.2	(24.1–32.9)
Pensacola-Ferry Pass-Brent, Florida	1,263	26.4	1.6	(23.2–29.6)
Philadelphia, Pennsylvania^§^	1,685	27.3	1.5	(24.4–30.2)
Phoenix-Mesa-Scottsdale, Arizona	1,470	25.1	1.9	(21.4–28.7)
Pittsburgh, Pennsylvania	2,247	29.2	1.4	(26.5–31.8)
Ponce, Puerto Rico	507	28.7	2.7	(23.4–34.0)
Portland-South Portland, Maine	2,555	24.6	1.2	(22.2–27.0)
Portland-Vancouver-Hillsboro, Oregon-Washington	3,094	24.7	1.1	(22.5–26.8)
Port St. Lucie, Florida	985	23.2	2.5	(18.3–28.0)
Providence-Warwick, Rhode Island-Massachusetts	7,821	27.3	1.0	(25.4–29.2)
Provo-Orem, Utah	1,735	24.0	1.2	(21.7–26.3)
Raleigh, North Carolina	624	25.0	2.0	(21.1–29.0)
Rapid City, South Dakota	832	28.7	2.2	(24.3–33.0)
Reno, Nevada	1,745	23.5	1.5	(20.6–26.3)
Richmond, Virginia	1,222	29.1	1.8	(25.7–32.6)
Riverside-San Bernardino-Ontario, California	1,273	29.1	1.6	(25.9–32.3)
Rochester, New York	480	26.7	2.4	(21.9–31.5)
Rockingham County-Strafford County, New Hampshire^§^	1,566	26.1	1.5	(23.1–29.1)
Sacramento-Roseville-Arden-Arcade, California	834	25.7	2.2	(21.3–30.0)
St. Louis, Missouri-Illinois	1,966	26.9	1.5	(24.0–29.9)
Salem, Oregon	496	33.9	3.3	(27.5–40.3)
Salina, Kansas	497	34.1	2.8	(28.7–39.5)
Salisbury, Maryland-Delaware	1,923	31.3	1.7	(27.9–34.7)
Salt Lake City, Utah	4,395	25.0	0.8	(23.4–26.6)
San Antonio-New Braunfels, Texas	884	35.6	2.2	(31.4–39.9)
San Francisco-Redwood City-South San Francisco, California^§^	497	12.1	1.7	(8.9–15.4)
San Jose-Sunnyvale-Santa Clara, California	577	18.3	2.3	(13.8–22.7)
San Juan-Carolina-Caguas, Puerto Rico	3,511	28.5	1.0	(26.4–30.5)
Scottsbluff, Nebraska	683	37.0	2.8	(31.5–42.4)
Scranton-Wilkes-Barre-Hazleton, Pennsylvania	540	30.3	3.0	(24.4–36.1)
Seattle-Bellevue-Everett, Washington	3,570	23.2	0.9	(21.4–24.9)
Shreveport-Bossier City, Louisiana	543	33.9	3.4	(27.2–40.6)
Silver Spring-Frederick-Rockville, Maryland^§^	2,241	19.8	1.2	(17.3–22.2)
Sioux City, Iowa-Nebraska-South Dakota	984	36.9	3.2	(30.5–43.2)
Sioux Falls, South Dakota	949	27.0	2.0	(23.1–30.9)
Spartanburg, South Carolina	566	30.7	3.0	(24.8–36.6)
Spokane-Spokane Valley, Washington	815	29.8	2.3	(25.3–34.2)
Springfield, Massachusetts	1,449	25.7	1.9	(21.9–29.5)
Tallahassee, Florida	1,769	29.6	2.1	(25.5–33.7)
Tampa-St. Petersburg-Clearwater, Florida	2,117	27.3	1.4	(24.5–30.0)
Toledo, Ohio	933	30.0	2.2	(25.6–34.4)
Topeka, Kansas	2,268	32.3	1.3	(29.7–34.9)
Tulsa, Oklahoma	1,899	32.3	1.5	(29.4–35.3)
Virginia Beach-Norfolk-Newport News, Virginia-North Carolina	1,573	27.9	1.6	(24.8–31.0)
Warren-Troy-Farmington Hills, Michigan^§^	2,172	29.3	1.4	(26.6–32.0)
Washington-Arlington-Alexandria, District of Columbia-Virginia-Maryland-West Virginia^§^	8,558	24.3	0.8	(22.6–25.9)
Wichita, Kansas	4,689	31.3	0.9	(29.6–33.0)
Wilmington, Delaware-Maryland-New Jersey^§^	3,066	29.6	1.2	(27.3–31.9)
Winston-Salem, North Carolina	652	30.6	2.7	(25.4–35.8)
Worcester, Massachusetts-Connecticut	2,595	26.5	1.4	(23.7–29.2)
*Median*		*28.3*		
*Range*		*12.1–37.1*		

**Abbreviations:** CI = confidence interval; MMSA = metropolitan and micropolitan statistical areas; SE = standard error.

* Age adjusted to the 2000 U.S. standard population.

^† ^Body mass index ≥30.

^§^ Metropolitan division.

**TABLE 41:** Age-adjusted* prevalence estimates of adults aged ≥18 years with obesity,^†^ by state/territory — Behavioral Risk Factor Surveillance System, United States, 2014

State/Territory	Sample size	%	SE	95% CI
Alabama	8,190	33.3	0.8	(31.7–34.8)
Alaska	4,142	29.3	1.0	(27.3–31.2)
Arizona	13,849	28.9	0.7	(27.5–30.2)
Arkansas	4,886	35.9	1.2	(33.6–38.2)
California	8,025	24.4	0.6	(23.2–25.6)
Colorado	12,567	21.0	0.5	(20.0–21.9)
Connecticut	7,237	25.4	0.8	(23.9–26.9)
Delaware	3,983	30.0	1.1	(27.8–32.2)
District of Columbia	3,787	22.0	1.1	(19.9–24.1)
Florida	9,156	26.1	0.7	(24.7–27.4)
Georgia	5,844	30.1	0.8	(28.5–31.8)
Hawaii	6,905	22.4	0.7	(20.9–23.8)
Idaho	5,125	28.8	1.0	(26.9–30.7)
Illinois	4,835	28.7	0.9	(26.9–30.5)
Indiana	10,773	32.3	0.6	(31.1–33.6)
Iowa	7,572	30.3	0.7	(28.8–31.7)
Kansas	12,825	30.9	0.5	(29.8–31.9)
Kentucky	10,442	31.3	0.8	(29.7–32.8)
Louisiana	6,366	34.6	0.8	(33.0–36.2)
Maine	8,705	27.5	0.7	(26.1–28.9)
Maryland	11,713	29.0	0.8	(27.4–30.6)
Massachusetts	14,394	22.6	0.6	(21.5–23.7)
Michigan	8,024	29.8	0.7	(28.5–31.2)
Minnesota	15,309	27.1	0.5	(26.2–28.0)
Mississippi	3,975	35.5	1.1	(33.3–37.7)
Missouri	6,695	30.0	0.9	(28.3–31.8)
Montana	7,064	26.0	0.8	(24.4–27.7)
Nebraska	21,130	29.9	0.5	(28.8–30.9)
Nevada	3,555	27.7	1.2	(25.3–30.2)
New Hampshire	5,730	26.8	0.9	(25.0–28.5)
New Jersey	11,955	26.3	0.6	(25.0–27.5)
New Mexico	8,284	28.6	0.8	(27.0–30.2)
New York	6,325	26.4	0.7	(25.0–27.9)
North Carolina	6,621	29.5	0.7	(28.1–30.9)
North Dakota	7,269	32.1	0.9	(30.2–33.9)
Ohio	10,313	31.7	0.8	(30.2–33.2)
Oklahoma	8,009	32.7	0.7	(31.3–34.0)
Oregon	4,919	27.5	0.9	(25.7–29.3)
Pennsylvania	10,374	29.8	0.7	(28.4–31.1)
Rhode Island	5,969	26.7	0.9	(25.0–28.5)
South Carolina	10,393	31.8	0.7	(30.5–33.1)
South Dakota	6,960	29.5	1.0	(27.5–31.5)
Tennessee	4,793	30.9	1.0	(28.9–33.0)
Texas	14,058	31.5	0.7	(30.2–32.9)
Utah	13,965	25.9	0.4	(25.1–26.8)
Vermont	6,068	24.1	0.7	(22.7–25.5)
Virginia	8,879	28.0	0.6	(26.7–29.2)
Washington	9,444	26.8	0.7	(25.5–28.1)
West Virginia	5,846	35.5	0.8	(33.9–37.2)
Wisconsin	6,655	30.8	0.9	(29.1–32.5)
Wyoming	5,973	29.3	1.1	(27.2–31.5)
Guam	2,352	27.7	1.2	(25.3–30.2)
Puerto Rico	5,743	28.0	0.8	(26.5–29.6)
*Median*		*29.0*		
*Range*		*21.0–35.9*		

**Abbreviations**: CI = confidence interval; SE = standard error.

* Age adjusted to the 2000 U.S. standard population.

^†^ Body mass index ≥30.

**TABLE 42:** Age-adjusted* prevalence estimates of adults aged ≥18 years with obesity,^†^ by metropolitan and micropolitan statistical area — Behavioral Risk Factor Surveillance System, United States, 2014

MMSA	Sample size	%	SE	95% CI
Aberdeen, South Dakota	573	33.2	3.0	(27.3–39.1)
Aguadilla-Isabela, Puerto Rico	522	29.7	2.6	(24.6–34.7)
Albuquerque, New Mexico	1,668	27.6	1.5	(24.6–30.6)
Allentown-Bethlehem-Easton, Pennsylvania-New Jersey	1,018	30.1	2.5	(25.2–35.1)
Anchorage, Alaska	1,689	29.5	1.4	(26.7–32.2)
Atlanta-Sandy Springs-Roswell, Georgia	2,568	28.0	1.1	(25.7–30.2)
Augusta-Richmond County, Georgia-South Carolina	832	28.6	2.7	(23.3–33.9)
Austin-Round Rock, Texas	2,084	25.7	1.3	(23.2–28.2)
Baltimore-Columbia-Towson, Maryland	4,298	29.9	1.3	(27.4–32.5)
Baton Rouge, Louisiana	868	35.5	2.1	(31.4–39.6)
Berlin, New Hampshire-Vermont	511	33.1	6.3	(20.8–45.4)
Billings, Montana	754	28.7	2.3	(24.2–33.2)
Birmingham-Hoover, Alabama	1,488	32.4	1.7	(29.2–35.7)
Bismarck, North Dakota	962	31.0	2.4	(26.4–35.7)
Boise City, Idaho	1,267	30.0	1.9	(26.3–33.7)
Boston, Massachusetts^§^	4,161	22.3	1.0	(20.3–24.2)
Burlington-South Burlington, Vermont	1,852	20.3	1.1	(18.1–22.5)
Cambridge-Newton-Framingham, Massachusetts^§^	4,774	21.8	1.0	(19.9–23.7)
Camden, New Jersey^§^	1,608	28.9	1.8	(25.4–32.4)
Cedar Rapids, Iowa	586	33.6	2.7	(28.3–38.8)
Charleston, West Virginia	830	34.6	2.2	(30.3–38.8)
Charleston-North Charleston, South Carolina	1,319	31.7	1.8	(28.1–35.2)
Charlotte-Concord-Gastonia, North Carolina-South Carolina	1,967	26.6	1.4	(23.8–29.3)
Chicago-Naperville-Elgin, Illinois-Indiana-Wisconsin	3,906	27.2	1.0	(25.2–29.3)
Cincinnati, Ohio-Kentucky-Indiana	1,911	31.7	1.8	(28.1–35.3)
Claremont-Lebanon, New Hampshire-Vermont	1,587	22.8	1.5	(19.9–25.6)
Cleveland-Elyria, Ohio	917	28.0	2.1	(23.9–32.1)
College Station-Bryan, Texas	530	34.0	4.0	(26.3–41.8)
Colorado Springs, Colorado	1,226	24.2	1.6	(21.1–27.4)
Columbia, South Carolina	1,130	33.0	1.8	(29.4–36.6)
Columbus, Ohio	1,578	31.9	1.8	(28.5–35.4)
Corpus Christi, Texas	576	42.5	4.1	(34.4–50.6)
Dallas-Plano-Irving, Texas^§^	1,192	29.0	2.0	(25.0–32.9)
Dayton, Ohio	538	36.5	2.9	(30.8–42.2)
Denver-Aurora-Lakewood, Colorado	5,400	19.8	0.7	(18.5–21.1)
Des Moines-West Des Moines, Iowa	1,254	27.5	1.8	(24.0–30.9)
Duluth, Minnesota-Wisconsin	893	28.3	2.1	(24.2–32.4)
El Paso, Texas	645	33.8	2.6	(28.8–38.9)
Evansville, Indiana-Kentucky	603	34.6	3.5	(27.7–41.6)
Fargo, North Dakota-Minnesota	1,083	27.7	1.8	(24.3–31.2)
Fayetteville-Springdale-Rogers, Arkansas-Missouri	758	31.5	2.9	(25.8–37.2)
Fort Wayne, Indiana	797	31.9	2.5	(27.0–36.8)
Fort Worth-Arlington, Texas^§^	698	30.7	2.5	(25.8–35.6)
Grand Island, Nebraska	981	35.6	2.6	(30.6–40.6)
Grand Rapids-Wyoming, Michigan	849	26.0	1.8	(22.5–29.5)
Greensboro-High Point, North Carolina	462	34.9	3.2	(28.7–41.1)
Greenville-Anderson-Mauldin, South Carolina	1,402	31.4	1.7	(28.1–34.8)
Hagerstown-Martinsburg, Maryland-West Virginia	739	28.8	2.8	(23.2–34.3)
Hartford-West Hartford-East Hartford, Connecticut	2,441	24.9	1.1	(22.6–27.1)
Hilton Head Island-Bluffton-Beaufort, South Carolina	524	22.4	2.6	(17.3–27.5)
Houston-The Woodlands-Sugar Land, Texas	1,943	31.0	1.7	(27.7–34.4)
Huntington-Ashland, West Virginia-Kentucky-Ohio	1,168	35.7	2.1	(31.6–39.7)
Idaho Falls, Idaho	487	32.9	2.8	(27.3–38.4)
Indianapolis-Carmel-Anderson, Indiana	3,391	31.9	1.2	(29.6–34.2)
Jacksonville, Florida	631	29.8	2.7	(24.6–35.0)
Kansas City, Missouri-Kansas	4,576	30.0	1.2	(27.6–32.4)
Kingsport-Bristol-Bristol, Tennessee-Virginia	477	29.3	3.6	(22.1–36.4)
Knoxville, Tennessee	531	30.7	3.3	(24.2–37.2)
Lafayette, Louisiana	518	33.2	2.6	(28.1–38.4)
Lexington-Fayette, Kentucky	592	25.9	2.3	(21.5–30.4)
Lincoln, Nebraska	1,909	25.5	1.3	(22.9–28.0)
Little Rock-North Little Rock-Conway, Arkansas	1,096	32.1	2.1	(27.9–36.2)
Logan, Utah-Idaho	578	22.8	1.9	(19.0–26.5)
Los Angeles-Long Beach-Anaheim, California	2,170	22.5	1.1	(20.3–24.7)
Louisville-Jefferson County, Kentucky-Indiana	2,323	30.5	1.8	(27.0–33.9)
Madison, Wisconsin	517	26.8	2.4	(22.0–31.6)
Memphis, Tennessee-Mississippi-Arkansas	826	34.1	2.8	(28.7–39.6)
Miami-Fort Lauderdale-West Palm Beach, Florida	2,027	24.3	1.4	(21.6–27.1)
Milwaukee-Waukesha-West Allis, Wisconsin	1,285	30.6	2.0	(26.7–34.6)
Minneapolis-St. Paul-Bloomington, Minnesota-Wisconsin	8,147	24.8	0.6	(23.6–25.9)
Minot, North Dakota	559	30.3	3.0	(24.5–36.1)
Montgomery, Alabama	482	33.2	2.9	(27.6–38.8)
Montgomery County-Bucks County-Chester County, Pennsylvania^§^	765	23.4	2.1	(19.2–27.6)
Myrtle Beach-Conway-North Myrtle Beach, South Carolina-North Carolina	935	30.0	2.2	(25.7–34.3)
Nashville-Davidson County-Murfreesboro-Franklin, Tennessee	743	27.2	2.1	(23.1–31.3)
Nassau County-Suffolk County, New York^§^	706	23.7	2.1	(19.6–27.9)
Newark, New Jersey-Pennsylvania^§^	3,796	26.1	1.1	(24.0–28.3)
New Orleans-Metairie, Louisiana	1,814	33.9	1.5	(31.0–36.8)
New York-Jersey City-White Plains, New York-New Jersey^§^	6,868	24.4	0.8	(22.9–25.9)
Norfolk, Nebraska	917	31.0	2.0	(27.2–34.9)
North Platte, Nebraska	921	31.9	2.1	(27.9–36.0)
North Port-Sarasota-Bradenton, Florida	477	26.9	4.3	(18.4–35.3)
Oakland-Hayward-Berkeley, California^§^	647	24.4	2.2	(20.1–28.7)
Ogden-Clearfield, Utah	2,742	27.8	1.0	(25.9–29.7)
Oklahoma City, Oklahoma	2,295	30.6	1.2	(28.2–33.0)
Omaha-Council Bluffs, Nebraska-Iowa	4,578	29.3	1.0	(27.4–31.2)
Orlando-Kissimmee-Sanford, Florida	897	26.3	2.0	(22.4–30.2)
Philadelphia, Pennsylvania^§^	1,429	29.9	1.8	(26.3–33.5)
Phoenix-Mesa-Scottsdale, Arizona	8,730	28.6	0.8	(27.1–30.2)
Pittsburgh, Pennsylvania	2,292	31.4	1.5	(28.5–34.3)
Ponce, Puerto Rico	511	30.9	2.8	(25.5–36.4)
Portland-South Portland, Maine	2,613	24.0	1.2	(21.6–26.5)
Portland-Vancouver-Hillsboro, Oregon-Washington	2,675	24.1	1.2	(21.7–26.4)
Providence-Warwick, Rhode Island-Massachusetts	7,503	26.5	0.9	(24.7–28.3)
Provo-Orem, Utah	1,995	25.6	1.1	(23.5–27.8)
Raleigh, North Carolina	660	23.9	2.0	(20.1–27.8)
Rapid City, South Dakota	1,337	26.9	2.1	(22.7–31.0)
Reno, Nevada	1,146	19.7	1.6	(16.7–22.8)
Richmond, Virginia	1,364	28.6	1.6	(25.4–31.7)
Riverside-San Bernardino-Ontario, California	859	27.7	2.0	(23.9–31.6)
Roanoke, Virginia	500	27.5	2.9	(21.8–33.2)
Rochester, Minnesota	667	24.1	1.9	(20.4–27.8)
Rockingham County-Strafford County, New Hampshire^§^	1,350	26.2	1.7	(22.9–29.5)
Sacramento-Roseville-Arden-Arcade, California	594	26.1	2.3	(21.5–30.7)
St. Cloud, Minnesota	525	29.3	2.3	(24.9–33.8)
St. Louis, Missouri-Illinois	1,807	27.6	1.7	(24.2–31.0)
Salisbury, Maryland-Delaware	1,804	32.5	2.1	(28.3–36.6)
Salt Lake City, Utah	5,010	26.5	0.7	(25.0–27.9)
San Antonio-New Braunfels, Texas	2,117	30.2	1.3	(27.6–32.8)
San Juan-Carolina-Caguas, Puerto Rico	3,615	27.3	0.9	(25.4–29.1)
Scottsbluff, Nebraska	854	35.1	2.4	(30.4–39.8)
Seattle-Bellevue-Everett, Washington^§^	3,459	22.7	1.0	(20.8–24.6)
Shreveport-Bossier City, Louisiana	515	36.0	3.0	(30.2–41.8)
Silver Spring-Frederick-Rockville, Maryland^§^	2,227	21.5	1.5	(18.5–24.5)
Sioux City, Iowa-Nebraska-South Dakota	1,069	30.5	2.7	(25.1–35.8)
Sioux Falls, South Dakota	1,260	27.3	2.0	(23.4–31.1)
Spartanburg, South Carolina	531	28.5	3.0	(22.6–34.4)
Spokane-Spokane Valley, Washington	688	31.1	2.4	(26.3–35.9)
Springfield, Massachusetts	1,024	23.9	2.1	(19.8–28.0)
Tampa-St. Petersburg-Clearwater, Florida	1,476	28.7	1.8	(25.1–32.3)
Toledo, Ohio	619	36.7	2.8	(31.3–42.2)
Topeka, Kansas	1,360	32.9	1.7	(29.6–36.3)
Tulsa, Oklahoma	1,916	30.2	1.4	(27.6–32.9)
Tuscaloosa, Alabama	680	32.2	2.2	(27.8–36.5)
Virginia Beach-Norfolk-Newport News, Virginia-North Carolina	1,771	31.0	1.5	(28.0–34.0)
Warren-Troy-Farmington Hills, Michigan^§^	2,012	27.8	1.4	(25.1–30.6)
Washington-Arlington-Alexandria, District of Columbia-Virginia-Maryland-West Virginia^§^	7,725	24.1	0.9	(22.4–25.8)
Wichita, Kansas	2,561	32.0	1.1	(29.8–34.2)
Wichita Falls, Texas	502	30.8	4.5	(22.0–39.7)
Wilmington, Delaware-Maryland-New Jersey^§^	2,575	30.6	1.4	(27.8–33.4)
Worcester, Massachusetts-Connecticut	2,239	25.4	1.4	(22.8–28.1)
Youngstown-Warren-Boardman, Ohio-Pennsylvania	495	29.5	3.4	(22.8–36.1)
*Median*		*29.3*		
*Range*		*19.7–42.5*		

**Abbreviations:** CI = confidence interval; MMSA = metropolitan and micropolitan statistical areas; SE = standard error.

* Age adjusted to the 2000 U.S. standard population.

^†^ Body mass index ≥30.

^§^ Metropolitan division.

#### Diabetes

Diabetes was defined as respondents aged ≥45 years who reported having ever been told by a doctor, nurse, or other health professional they have diabetes, excluding prediabetes or borderline diabetes and diabetes during pregnancy for women. In 2013, age-adjusted prevalence estimates of diabetes among adults aged ≥45 years ranged from 11.1% in Colorado to 27.5% in Guam (median: 16.2%) (Table 43). Among selected MMSA, age-adjusted prevalence estimates ranged from 10.6% in Buffalo-Cheektowaga-Niagara Falls, New York, to 24.8% in Aguadilla-Isabela, Puerto Rico (median: 15.8%) (Table 44).

**TABLE 43:** Age-adjusted* prevalence estimates of adults aged ≥45 years who have ever been told by a health professional they have diabetes,^†^ by state/territory — Behavioral Risk Factor Surveillance System, United States, 2013

State/Territory	Sample size	%	SE	95% CI
Alabama	4,837	21.5	0.8	(19.9–23.1)
Alaska	2,715	13.7	1.0	(11.7–15.7)
Arizona	3,063	17.9	1.3	(15.3–20.6)
Arkansas	3,945	17.5	0.9	(15.8–19.2)
California	6,863	18.2	0.7	(16.8–19.6)
Colorado	9,231	11.1	0.4	(10.3–12.0)
Connecticut	5,341	13.0	0.6	(11.8–14.2)
Delaware	3,678	17.1	0.8	(15.6–18.7)
District of Columbia	3,478	16.0	0.9	(14.2–17.9)
Florida	26,015	17.2	0.6	(16.1–18.3)
Georgia	5,396	18.7	0.7	(17.3–20.1)
Hawaii	4,776	14.0	0.8	(12.4–15.7)
Idaho	3,880	13.5	0.8	(12.1–15.0)
Illinois	3,912	16.6	0.8	(15.0–18.3)
Indiana	7,375	18.3	0.6	(17.2–19.4)
Iowa	5,918	14.8	0.6	(13.7–15.9)
Kansas	16,075	15.7	0.3	(15.0–16.3)
Kentucky	7,792	16.8	0.6	(15.6–18.1)
Louisiana	3,803	19.6	0.9	(17.8–21.4)
Maine	6,015	14.3	0.6	(13.1–15.4)
Maryland	9,246	16.2	0.6	(15.1–17.3)
Massachusetts	10,399	13.8	0.5	(12.7–14.9)
Michigan	8,954	16.3	0.5	(15.2–17.3)
Minnesota	9,688	12.6	0.7	(11.2–14.0)
Mississippi	5,497	20.1	0.7	(18.6–21.6)
Missouri	5,057	14.8	0.7	(13.4–16.1)
Montana	6,703	11.6	0.5	(10.6–12.6)
Nebraska	12,159	15.1	0.5	(14.0–16.2)
Nevada	3,441	16.3	1.4	(13.6–18.9)
New Hampshire	4,743	14.4	0.7	(13.1–15.7)
New Jersey	8,465	15.5	0.6	(14.2–16.7)
New Mexico	6,323	17.1	0.8	(15.6–18.6)
New York	5,673	18.0	0.7	(16.5–19.4)
North Carolina	6,032	18.8	0.7	(17.4–20.1)
North Dakota	5,335	15.0	0.7	(13.7–16.3)
Ohio	8,426	16.5	0.6	(15.4–17.7)
Oklahoma	5,816	17.8	0.6	(16.6–19.0)
Oregon	4,239	14.9	0.8	(13.4–16.4)
Pennsylvania	7,956	15.8	0.6	(14.7–16.8)
Rhode Island	4,636	14.7	0.7	(13.4–16.0)
South Carolina	7,664	20.1	0.7	(18.8–21.4)
South Dakota	4,652	14.2	0.9	(12.5–15.9)
Tennessee	3,815	21.1	1.0	(19.2–23.0)
Texas	7,098	19.3	0.8	(17.7–20.8)
Utah	7,395	14.5	0.5	(13.4–15.5)
Vermont	4,673	12.2	0.6	(11.0–13.3)
Virginia	5,648	16.5	0.7	(15.2–17.8)
Washington	7,837	14.2	0.5	(13.2–15.3)
West Virginia	4,063	19.5	0.7	(18.1–20.9)
Wisconsin	4,630	13.5	0.8	(11.9–15.0)
Wyoming	4,938	13.2	0.6	(12.0–14.4)
Guam	761	27.5	2.4	(22.8–32.3)
Puerto Rico	3,905	24.7	0.8	(23.1–26.4)
*Median*		*16.2*		
*Range*		*11.1–27.5*		

**Abbreviations:** CI = confidence interval; SE = standard error.

* Age adjusted to the 2000 U.S. standard population.

^†^ Excluding prediabetes or borderline diabetes and diabetes during pregnancy for women.

**TABLE 44:** Age-adjusted* prevalence estimates of adults aged ≥45 years who have ever been told by a health professional they have diabetes,^†^ by metropolitan and micropolitan statistical area — Behavioral Risk Factor Surveillance System, United States, 2013

MMSA	Sample size	%	SE	95% CI
Aguadilla-Isabela, Puerto Rico	397	24.8	2.6	(19.6–29.9)
Akron, Ohio	529	14.5	2.0	(10.6–18.3)
Albuquerque, New Mexico	1,311	16.4	1.5	(13.4–19.4)
Allentown-Bethlehem-Easton, Pennsylvania-New Jersey	701	16.0	2.3	(11.4–20.6)
Anchorage, Alaska	898	14.5	1.6	(11.3–17.7)
Atlanta-Sandy Springs-Roswell, Georgia	2,187	17.6	1.1	(15.4–19.9)
Augusta-Richmond County, Georgia-South Carolina	650	22.0	2.3	(17.5–26.5)
Austin-Round Rock, Texas	585	16.2	2.2	(11.9–20.4)
Baltimore-Columbia-Towson, Maryland	3,305	16.0	0.9	(14.3–17.6)
Baton Rouge, Louisiana	645	23.0	2.4	(18.4–27.7)
Billings, Montana	478	11.0	1.5	(8.1–14.0)
Birmingham-Hoover, Alabama	954	19.6	1.6	(16.5–22.7)
Bismarck, North Dakota	684	12.9	1.6	(9.7–16.0)
Boise City, Idaho	970	13.0	1.4	(10.3–15.8)
Boston, Massachusetts^§^	2,722	14.4	1.0	(12.4–16.5)
Buffalo-Cheektowaga-Niagara Falls, New York	352	10.6	1.9	(7.0–14.2)
Burlington-South Burlington, Vermont	1,067	11.3	1.1	(9.0–13.5)
Cambridge-Newton-Framingham, Massachusetts^§^	3,266	13.1	1.0	(11.1–15.1)
Camden, New Jersey^§^	1,211	16.6	1.3	(14.0–19.2)
Cedar Rapids, Iowa	460	15.4	1.9	(11.8–19.1)
Charleston, West Virginia	576	21.5	2.0	(17.7–25.3)
Charleston-North Charleston, South Carolina	1,061	20.6	1.8	(17.0–24.1)
Charlotte-Concord-Gastonia, North Carolina-South Carolina	1,267	19.1	1.4	(16.2–21.9)
Chattanooga, Tennessee-Georgia	423	23.8	2.9	(18.2–29.5)
Chicago-Naperville-Elgin, Illinois-Indiana-Wisconsin	2,253	16.7	1.1	(14.4–18.9)
Cincinnati, Ohio-Kentucky-Indiana	1,806	15.7	1.2	(13.4–18.0)
Claremont-Lebanon, New Hampshire-Vermont	1,253	14.3	2.0	(10.4–18.2)
Cleveland-Elyria, Ohio	787	17.8	1.8	(14.4–21.3)
Colorado Springs, Colorado	920	11.6	1.3	(9.0–14.3)
Columbia, South Carolina	960	16.0	1.8	(12.4–19.5)
Columbus, Ohio	1,158	17.3	1.5	(14.3–20.3)
Crestview-Fort Walton Beach-Destin, Florida	771	15.2	1.5	(12.2–18.2)
Dallas-Plano-Irving, Texas^§^	514	15.0	1.9	(11.3–18.7)
Davenport-Moline-Rock Island, Iowa-Illinois	494	18.6	2.7	(13.3–23.8)
Dayton, Ohio	611	15.4	1.7	(12.0–18.7)
Deltona-Daytona Beach-Ormond Beach, Florida	901	15.0	1.8	(11.4–18.5)
Denver-Aurora-Lakewood, Colorado	3,583	11.2	0.7	(9.9–12.5)
Des Moines-West Des Moines, Iowa	960	15.0	1.3	(12.4–17.6)
Duluth, Minnesota-Wisconsin	505	16.2	2.9	(10.5–22.0)
Durham-Chapel Hill, North Carolina	421	14.5	2.2	(10.2–18.7)
El Paso, Texas	479	22.2	2.3	(17.7–26.6)
Evansville, Indiana-Kentucky	428	21.9	2.8	(16.4–27.4)
Fargo, North Dakota-Minnesota	718	13.4	1.8	(9.9–16.9)
Fayetteville-Springdale-Rogers, Arkansas-Missouri	569	12.3	1.7	(8.9–15.6)
Fort Smith, Arkansas-Oklahoma	382	18.6	2.6	(13.5–23.7)
Fort Wayne, Indiana	549	15.5	1.8	(11.9–19.1)
Fort Worth-Arlington, Texas^§^	564	24.0	2.7	(18.8–29.2)
Gainesville, Florida	745	14.0	2.1	(10.0–18.0)
Grand Forks, North Dakota-Minnesota	340	16.7	3.2	(10.4–23.0)
Grand Island, Nebraska	595	15.4	1.8	(11.8–19.0)
Grand Rapids-Wyoming, Michigan	880	14.7	1.4	(11.9–17.5)
Greensboro-High Point, North Carolina	453	20.5	2.4	(15.8–25.3)
Greenville-Anderson-Mauldin, South Carolina	922	18.9	1.6	(15.8–22.1)
Gulfport-Biloxi-Pascagoula, Mississippi	548	18.7	1.9	(15.1–22.4)
Hagerstown-Martinsburg, Maryland-West Virginia	559	17.2	1.9	(13.4–20.9)
Hartford-West Hartford-East Hartford, Connecticut	1,991	12.0	1.0	(10.1–14.0)
Hilton Head Island-Bluffton-Beaufort, South Carolina	673	14.7	1.9	(11.0–18.4)
Houston-The Woodlands-Sugar Land, Texas	757	19.5	2.4	(14.7–24.2)
Huntington-Ashland, West Virginia-Kentucky-Ohio	793	23.2	1.8	(19.6–26.8)
Idaho Falls, Idaho	320	13.5	2.4	(8.8–18.2)
Indianapolis-Carmel-Anderson, Indiana	1,734	18.4	1.1	(16.2–20.5)
Jackson, Mississippi	536	12.8	1.8	(9.4–16.3)
Jacksonville, Florida	2,009	19.7	1.5	(16.7–22.7)
Kansas City, Missouri-Kansas	5,092	14.0	1.0	(12.0–16.0)
Kingsport-Bristol-Bristol, Tennessee-Virginia	397	21.9	3.0	(16.1–27.8)
Knoxville, Tennessee	409	20.4	2.9	(14.7–26.0)
Lansing-East Lansing, Michigan	462	18.4	2.2	(14.0–22.8)
Lexington-Fayette, Kentucky	372	15.1	2.4	(10.3–19.8)
Lincoln, Nebraska	1,087	14.6	1.2	(12.3–16.9)
Little Rock-North Little Rock-Conway, Arkansas	822	19.2	2.1	(15.1–23.3)
Logan, Utah-Idaho	356	12.8	2.3	(8.2–17.4)
Los Angeles-Long Beach-Anaheim, California	1,708	22.1	1.5	(19.1–25.1)
Louisville-Jefferson County, Kentucky-Indiana	1,525	15.0	1.4	(12.2–17.7)
Lubbock, Texas	400	16.8	2.2	(12.6–21.1)
Manhattan, Kansas	380	12.2	1.8	(8.6–15.8)
Memphis, Tennessee-Mississippi-Arkansas	811	22.8	2.2	(18.6–27.1)
Miami-Fort Lauderdale-West Palm Beach, Florida	1,561	16.8	1.6	(13.7–19.9)
Milwaukee-Waukesha-West Allis, Wisconsin	887	13.5	1.8	(10.0–17.1)
Minneapolis-St. Paul-Bloomington, Minnesota-Wisconsin	6,093	12.3	1.0	(10.4–14.2)
Minot, North Dakota	408	16.4	2.3	(11.9–20.9)
Montgomery County-Bucks County-Chester County, Pennsylvania^§^	626	10.8	1.3	(8.2–13.4)
Myrtle Beach-Conway-North Myrtle Beach, South Carolina-North Carolina	571	14.7	1.8	(11.2–18.2)
Nashville-Davidson County-Murfreesboro-Franklin, Tennessee	646	16.3	1.9	(12.7–19.9)
Nassau County-Suffolk County, New York^§^	604	14.5	1.8	(10.9–18.1)
Newark, New Jersey-Pennsylvania^§^	2,610	13.4	0.9	(11.6–15.3)
New Orleans-Metairie, Louisiana	919	19.6	2.1	(15.5–23.7)
New York-Jersey City-White Plains, New York-New Jersey^§^	5,323	19.7	0.9	(17.9–21.6)
Norfolk, Nebraska	448	18.1	2.2	(13.8–22.5)
North Platte, Nebraska	530	16.5	2.1	(12.3–20.7)
North Port-Sarasota-Bradenton, Florida	856	12.6	1.6	(9.4–15.7)
Oakland-Hayward-Berkeley, California^§^	391	14.9	2.4	(10.2–19.6)
Ogden-Clearfield, Utah	1,394	15.3	1.1	(13.1–17.6)
Oklahoma City, Oklahoma	1,770	17.2	1.1	(15.1–19.3)
Omaha-Council Bluffs, Nebraska-Iowa	2,009	16.9	1.2	(14.6–19.2)
Orlando-Kissimmee-Sanford, Florida	1,575	19.8	1.5	(16.9–22.8)
Panama City, Florida	801	18.7	2.3	(14.2–23.3)
Pensacola-Ferry Pass-Brent, Florida	878	17.6	1.6	(14.4–20.8)
Philadelphia, Pennsylvania^§^	1,161	16.8	1.5	(13.9–19.7)
Phoenix-Mesa-Scottsdale, Arizona	1,056	18.4	2.0	(14.6–22.3)
Pittsburgh, Pennsylvania	1,698	15.3	1.2	(12.9–17.6)
Ponce, Puerto Rico	333	23.2	2.7	(17.9–28.6)
Portland-South Portland, Maine	1,930	11.5	0.9	(9.8–13.1)
Portland-Vancouver-Hillsboro, Oregon-Washington	2,257	15.2	1.1	(13.1–17.3)
Port St. Lucie, Florida	831	15.7	1.7	(12.3–19.1)
Providence-Warwick, Rhode Island-Massachusetts	5,942	14.2	0.8	(12.7–15.7)
Provo-Orem, Utah	857	14.0	1.4	(11.4–16.7)
Raleigh, North Carolina	360	19.3	2.4	(14.7–24.0)
Rapid City, South Dakota	601	13.3	1.7	(10.0–16.5)
Reno, Nevada	1,208	13.8	1.3	(11.2–16.4)
Richmond, Virginia	858	17.1	1.8	(13.5–20.6)
Riverside-San Bernardino-Ontario, California	814	20.1	1.8	(16.5–23.7)
Rochester, New York	336	14.1	1.9	(10.3–17.9)
Rockingham County-Strafford County, New Hampshire^§^	1,178	14.2	1.2	(11.9–16.6)
Sacramento-Roseville-Arden-Arcade, California	566	15.8	2.6	(10.7–20.8)
St. Louis, Missouri-Illinois	1,438	15.4	1.3	(12.9–17.9)
Salem, Oregon	379	15.2	2.2	(10.9–19.5)
Salina, Kansas	369	17.8	2.2	(13.4–22.1)
Salisbury, Maryland-Delaware	1,598	19.2	1.4	(16.5–21.9)
Salt Lake City, Utah	2,689	15.3	0.9	(13.6–17.0)
San Antonio-New Braunfels, Texas	613	18.9	2.3	(14.5–23.3)
San Francisco-Redwood City-South San Francisco, California^§^	303	11.2	2.9	(5.6–16.8)
San Jose-Sunnyvale-Santa Clara, California	336	14.1	2.6	(9.0–19.2)
San Juan-Carolina-Caguas, Puerto Rico	2,338	23.7	1.0	(21.7–25.7)
Scottsbluff, Nebraska	547	14.0	1.7	(10.7–17.3)
Scranton-Wilkes-Barre-Hazleton, Pennsylvania	427	12.8	1.8	(9.4–16.3)
Seattle-Bellevue-Everett, Washington^§^	2,449	13.2	0.9	(11.4–14.9)
Shreveport-Bossier City, Louisiana	437	14.7	1.9	(11.0–18.4)
Silver Spring-Frederick-Rockville, Maryland^§^	1,651	13.6	1.2	(11.3–15.9)
Sioux City, Iowa-Nebraska-South Dakota	750	17.4	2.3	(12.8–22.0)
Sioux Falls, South Dakota	596	15.3	2.0	(11.3–19.3)
Spartanburg, South Carolina	453	22.7	2.8	(17.3–28.2)
Spokane-Spokane Valley, Washington	630	13.8	1.7	(10.5–17.2)
Springfield, Massachusetts	1,133	14.7	1.6	(11.6–17.8)
Tallahassee, Florida	1,385	17.8	1.8	(14.3–21.3)
Tampa-St. Petersburg-Clearwater, Florida	1,586	17.0	1.3	(14.5–19.6)
Toledo, Ohio	711	15.5	1.7	(12.1–18.9)
Topeka, Kansas	1,707	15.8	1.1	(13.7–17.9)
Tulsa, Oklahoma	1,427	16.9	1.2	(14.5–19.2)
Virginia Beach-Norfolk-Newport News, Virginia-North Carolina	1,114	18.1	1.7	(14.8–21.3)
Warren-Troy-Farmington Hills, Michigan^§^	1,596	15.6	1.1	(13.4–17.8)
Washington-Arlington-Alexandria, District of Columbia-Virginia-Maryland-West Virginia^§^	6,065	15.0	0.9	(13.1–16.8)
Wichita, Kansas	3,382	17.9	0.8	(16.4–19.5)
Wilmington, Delaware-Maryland-New Jersey^§^	2,239	15.2	1.0	(13.3–17.1)
Winston-Salem, North Carolina	513	16.9	1.9	(13.2–20.5)
Worcester, Massachusetts-Connecticut	1,891	15.9	1.4	(13.2–18.6)
*Median*		*15.8*		
*Range*		*10.6–24.8*		

**Abbreviations:** CI = confidence interval; MMSA = metropolitan and micropolitan statistical areas; SE = standard error.

* Age adjusted to the 2000 U.S. standard population.

^†^ Excluding prediabetes or borderline diabetes and diabetes during pregnancy for women.

^§^ Metropolitan division.

#### Arthritis 

Arthritis was defined as respondents aged ≥45 years who reported having ever been told by a doctor, nurse, or other health professional they have some form of arthritis, including rheumatoid arthritis, gout, lupus, or fibromyalgia. In 2013, age-adjusted prevalence estimates of arthritis among adults aged ≥45 years ranged from 30.6% in Hawaii to 51.0% in West Virginia (median: 39.4%) (Table 45). Among selected MMSA, age-adjusted prevalence estimates ranged from 27.6% in Dallas-Plano-Irving, Texas, to 52.4% in Huntington-Ashland, West Virginia-Kentucky-Ohio (median: 39.4%) (Table 46). In 2014, age-adjusted prevalence estimates ranged from 31.2% in Hawaii to 54.7% in West Virginia (median: 39.8%) (Table 47). Among selected MMSA, age-adjusted prevalence ranged from 28.4% in College Station-Bryan, Texas, to 54.7% in Montgomery, Alabama (median: 40.1%) (Table 48).

**TABLE 45:** Age-adjusted* prevalence estimates of adults aged ≥45 years who have ever been told by a health professional they have some form of arthritis,^†^ by state/territory — Behavioral Risk Factor Surveillance System, United States, 2013

State/Territory	Sample size	%	SE	95% CI
Alabama	4,976	48.8	1.0	(46.9–50.7)
Alaska	2,786	38.6	1.3	(36.0–41.3)
Arizona	3,112	39.0	1.5	(36.1–41.9)
Arkansas	4,006	43.7	1.1	(41.5–45.8)
California	7,150	35.9	0.8	(34.3–37.4)
Colorado	9,389	36.5	0.6	(35.3–37.7)
Connecticut	5,432	37.2	0.9	(35.4–38.9)
Delaware	3,755	40.3	1.1	(38.3–42.4)
District of Columbia	3,542	35.9	1.2	(33.6–38.1)
Florida	26,506	37.6	0.7	(36.3–39.0)
Georgia	5,548	40.4	0.9	(38.7–42.1)
Hawaii	5,126	30.6	1.0	(28.7–32.5)
Idaho	3,960	37.9	1.1	(35.8–40.0)
Illinois	3,977	39.4	1.1	(37.3–41.5)
Indiana	7,485	42.6	0.7	(41.3–44.0)
Iowa	6,023	36.9	0.8	(35.4–38.4)
Kansas	16,437	37.4	0.4	(36.6–38.3)
Kentucky	8,060	46.2	0.9	(44.5–47.9)
Louisiana	3,976	41.0	1.2	(38.8–43.3)
Maine	6,118	42.4	0.8	(40.8–44.0)
Maryland	9,552	38.2	0.7	(36.9–39.6)
Massachusetts	10,636	38.0	0.7	(36.6–39.4)
Michigan	9,124	46.3	0.7	(45.0–47.7)
Minnesota	9,912	31.0	0.9	(29.2–32.9)
Mississippi	5,603	45.9	1.0	(44.0–47.8)
Missouri	5,196	42.8	1.0	(40.7–44.8)
Montana	6,837	39.0	0.8	(37.4–40.7)
Nebraska	12,400	39.0	0.7	(37.6–40.3)
Nevada	3,495	33.1	1.4	(30.3–35.8)
New Hampshire	4,823	39.7	0.9	(37.9–41.4)
New Jersey	8,730	35.0	0.7	(33.6–36.5)
New Mexico	6,445	37.8	0.9	(36.1–39.5)
New York	5,783	39.7	0.8	(38.0–41.3)
North Carolina	6,179	40.7	0.8	(39.1–42.4)
North Dakota	5,429	40.1	0.9	(38.3–41.8)
Ohio	8,608	43.4	0.8	(41.9–44.9)
Oklahoma	5,942	40.6	0.8	(39.0–42.1)
Oregon	4,320	40.0	0.9	(38.2–41.9)
Pennsylvania	8,103	43.2	0.7	(41.8–44.6)
Rhode Island	4,729	41.3	0.9	(39.6–43.0)
South Carolina	7,818	44.8	0.8	(43.2–46.4)
South Dakota	4,719	38.3	1.2	(35.9–40.7)
Tennessee	4,254	39.8	1.1	(37.6–41.9)
Texas	7,211	35.5	0.9	(33.7–37.2)
Utah	7,533	35.9	0.7	(34.6–37.3)
Vermont	4,721	40.3	0.9	(38.7–42.0)
Virginia	5,800	40.2	0.8	(38.5–41.8)
Washington	7,995	41.0	0.7	(39.6–42.5)
West Virginia	4,147	51.0	0.9	(49.2–52.8)
Wisconsin	4,687	38.0	1.1	(35.8–40.2)
Wyoming	4,996	38.3	0.9	(36.5–40.0)
Guam	810	32.0	2.2	(27.7–36.2)
Puerto Rico	4,011	39.0	1.0	(37.1–40.8)
*Median*		*39.4*		
*Range *		*30.6–51.0*		

**Abbreviations:** CI = confidence interval; SE = standard error.

* Age adjusted to the 2000 U.S. standard population.

^†^ Including arthritis, rheumatoid arthritis, gout, lupus, or fibromyalgia.

**TABLE 46:** Age-adjusted* prevalence estimates of adults aged ≥45 years who have ever been told by a health professional they have some form of arthritis,^†^ by metropolitan and micropolitan statistical area — Behavioral Risk Factor Surveillance System, United States, 2013

MMSA	Sample size	%	SE	95% CI
Aguadilla-Isabela, Puerto Rico	406	36.1	2.8	(30.5–41.6)
Akron, Ohio	539	41.7	2.7	(36.4–47.0)
Albuquerque, New Mexico	1,337	38.3	1.7	(35.0–41.6)
Allentown-Bethlehem-Easton, Pennsylvania-New Jersey	719	42.5	2.8	(37.1–47.9)
Anchorage, Alaska	925	38.0	2.1	(34.0–42.0)
Atlanta-Sandy Springs-Roswell, Georgia	2,250	36.8	1.3	(34.2–39.3)
Augusta-Richmond County, Georgia-South Carolina	669	42.8	2.7	(37.5–48.0)
Austin-Round Rock, Texas	592	35.2	2.7	(29.8–40.6)
Baltimore-Columbia-Towson, Maryland	3,428	38.8	1.1	(36.6–41.0)
Baton Rouge, Louisiana	671	42.1	2.5	(37.3–47.0)
Billings, Montana	492	39.6	2.4	(34.9–44.3)
Birmingham-Hoover, Alabama	984	48.7	1.9	(44.9–52.5)
Bismarck, North Dakota	692	39.9	2.2	(35.6–44.2)
Boise City, Idaho	988	34.9	2.0	(31.0–38.7)
Boston, Massachusetts^§^	2,785	37.6	1.4	(34.9–40.4)
Buffalo-Cheektowaga-Niagara Falls, New York	365	42.0	3.2	(35.7–48.4)
Burlington-South Burlington, Vermont	1,074	39.0	1.7	(35.6–42.4)
Cambridge-Newton-Framingham, Massachusetts^§^	3,341	34.7	1.2	(32.4–37.1)
Camden, New Jersey^§^	1,246	38.6	1.8	(35.1–42.0)
Cedar Rapids, Iowa	468	33.6	2.5	(28.8–38.5)
Charleston, West Virginia	585	50.0	2.4	(45.3–54.8)
Charleston-North Charleston, South Carolina	1,082	39.3	2.0	(35.5–43.2)
Charlotte-Concord-Gastonia, North Carolina-South Carolina	1,291	39.4	1.8	(35.9–43.0)
Chattanooga, Tennessee-Georgia	459	36.6	2.9	(30.8–42.3)
Chicago-Naperville-Elgin, Illinois-Indiana-Wisconsin	2,278	38.8	1.4	(36.0–41.5)
Cincinnati, Ohio-Kentucky-Indiana	1,846	41.2	1.6	(38.1–44.3)
Claremont-Lebanon, New Hampshire-Vermont	1,260	37.8	1.8	(34.3–41.3)
Cleveland-Elyria, Ohio	800	43.8	2.2	(39.5–48.2)
Colorado Springs, Colorado	934	38.8	1.9	(35.1–42.5)
Columbia, South Carolina	983	44.4	2.3	(39.9–48.8)
Columbus, Ohio	1,185	42.0	1.8	(38.6–45.5)
Crestview-Fort Walton Beach-Destin, Florida	781	40.0	2.2	(35.6–44.3)
Dallas-Plano-Irving, Texas^§^	523	27.6	2.1	(23.4–31.8)
Davenport-Moline-Rock Island, Iowa-Illinois	506	37.8	2.7	(32.6–43.0)
Dayton, Ohio	631	42.0	2.5	(37.2–46.9)
Deltona-Daytona Beach-Ormond Beach, Florida	917	44.2	2.5	(39.4–49.1)
Denver-Aurora-Lakewood, Colorado	3,654	36.3	0.9	(34.4–38.1)
Des Moines-West Des Moines, Iowa	971	39.5	1.9	(35.7–43.3)
Duluth, Minnesota-Wisconsin	515	39.7	3.8	(32.3–47.1)
Durham-Chapel Hill, North Carolina	430	36.9	2.7	(31.7–42.2)
El Paso, Texas	483	35.7	2.8	(30.2–41.1)
Evansville, Indiana-Kentucky	437	44.3	3.0	(38.4–50.1)
Fargo, North Dakota-Minnesota	737	39.2	2.7	(33.9–44.5)
Fayetteville-Springdale-Rogers, Arkansas-Missouri	582	39.1	2.7	(33.8–44.4)
Fort Smith, Arkansas-Oklahoma	380	40.7	3.2	(34.6–46.9)
Fort Wayne, Indiana	562	42.2	2.5	(37.3–47.1)
Fort Worth-Arlington, Texas^§^	567	37.4	2.9	(31.7–43.1)
Gainesville, Florida	762	40.7	2.7	(35.4–46.0)
Grand Forks, North Dakota-Minnesota	344	35.7	3.7	(28.5–43.0)
Grand Island, Nebraska	610	42.9	2.5	(37.9–47.9)
Grand Rapids-Wyoming, Michigan	899	42.6	2.0	(38.6–46.6)
Greensboro-High Point, North Carolina	461	41.4	2.9	(35.7–47.2)
Greenville-Anderson-Mauldin, South Carolina	936	43.5	2.1	(39.5–47.5)
Gulfport-Biloxi-Pascagoula, Mississippi	560	44.1	2.6	(39.0–49.2)
Hagerstown-Martinsburg, Maryland-West Virginia	578	45.5	2.6	(40.5–50.6)
Hartford-West Hartford-East Hartford, Connecticut	2,022	37.8	1.5	(34.9–40.7)
Hilton Head Island-Bluffton-Beaufort, South Carolina	679	37.6	2.6	(32.6–42.6)
Houston-The Woodlands-Sugar Land, Texas	774	35.9	2.5	(30.9–40.8)
Huntington-Ashland, West Virginia-Kentucky-Ohio	817	52.4	2.2	(48.2–56.7)
Idaho Falls, Idaho	333	40.6	3.6	(33.5–47.6)
Indianapolis-Carmel-Anderson, Indiana	1,759	41.4	1.4	(38.7–44.0)
Jackson, Mississippi	545	43.3	2.8	(37.8–48.8)
Jacksonville, Florida	2,057	39.6	1.7	(36.3–42.9)
Kansas City, Missouri-Kansas	5,189	42.1	1.6	(39.0–45.2)
Kingsport-Bristol-Bristol, Tennessee-Virginia	440	48.5	3.5	(41.6–55.5)
Knoxville, Tennessee	467	43.1	3.3	(36.6–49.5)
Lansing-East Lansing, Michigan	467	46.4	2.7	(41.0–51.8)
Lexington-Fayette, Kentucky	381	38.8	3.2	(32.6–45.1)
Lincoln, Nebraska	1,104	36.7	1.6	(33.5–39.9)
Little Rock-North Little Rock-Conway, Arkansas	841	41.7	2.2	(37.3–46.0)
Logan, Utah-Idaho	362	34.8	3.0	(28.9–40.7)
Los Angeles-Long Beach-Anaheim, California	1,786	34.1	1.6	(31.0–37.1)
Louisville-Jefferson County, Kentucky-Indiana	1,579	43.2	2.1	(39.0–47.3)
Lubbock, Texas	406	34.9	2.8	(29.4–40.5)
Manhattan, Kansas	388	34.4	2.6	(29.3–39.4)
Memphis, Tennessee-Mississippi-Arkansas	847	37.5	2.2	(33.1–41.8)
Miami-Fort Lauderdale-West Palm Beach, Florida	1,593	31.7	1.8	(28.2–35.2)
Milwaukee-Waukesha-West Allis, Wisconsin	900	38.9	2.6	(33.8–44.0)
Minneapolis-St. Paul-Bloomington, Minnesota-Wisconsin	6,240	29.7	1.2	(27.3–32.1)
Minot, North Dakota	418	34.2	2.7	(28.8–39.6)
Montgomery County-Bucks County-Chester County, Pennsylvania^§^	638	36.9	2.2	(32.7–41.2)
Myrtle Beach-Conway-North Myrtle Beach, South Carolina-North Carolina	583	52.0	3.0	(46.1–57.9)
Nashville-Davidson County-Murfreesboro-Franklin, Tennessee	699	35.5	2.3	(30.9–40.0)
Nassau County-Suffolk County, New York^§^	610	36.5	2.3	(31.9–41.0)
Newark, New Jersey-Pennsylvania^§^	2,693	31.5	1.2	(29.1–33.9)
New Orleans-Metairie, Louisiana	960	39.4	2.6	(34.3–44.5)
New York-Jersey City-White Plains, New York-New Jersey^§^	5,441	36.3	1.0	(34.3–38.2)
Norfolk, Nebraska	452	39.7	2.6	(34.7–44.7)
North Platte, Nebraska	539	40.1	2.6	(34.9–45.3)
North Port-Sarasota-Bradenton, Florida	862	37.9	2.3	(33.4–42.5)
Oakland-Hayward-Berkeley, California^§^	408	32.4	2.9	(26.8–38.1)
Ogden-Clearfield, Utah	1,424	38.4	1.5	(35.4–41.3)
Oklahoma City, Oklahoma	1,813	39.2	1.4	(36.5–41.9)
Omaha-Council Bluffs, Nebraska-Iowa	2,050	40.0	1.4	(37.2–42.7)
Orlando-Kissimmee-Sanford, Florida	1,611	37.3	1.7	(33.9–40.6)
Panama City, Florida	806	46.8	2.8	(41.3–52.3)
Pensacola-Ferry Pass-Brent, Florida	894	41.1	2.1	(37.1–45.2)
Philadelphia, Pennsylvania^§^	1,183	45.8	2.0	(41.9–49.7)
Phoenix-Mesa-Scottsdale, Arizona	1,077	38.8	2.1	(34.6–43.0)
Pittsburgh, Pennsylvania	1,725	45.2	1.5	(42.2–48.2)
Ponce, Puerto Rico	342	34.4	3.1	(28.4–40.5)
Portland-South Portland, Maine	1,973	39.3	1.4	(36.6–42.1)
Portland-Vancouver-Hillsboro, Oregon-Washington	2,294	38.4	1.3	(35.9–40.9)
Port St. Lucie, Florida	840	40.3	2.4	(35.6–45.0)
Providence-Warwick, Rhode Island-Massachusetts	6,061	42.1	1.1	(39.9–44.4)
Provo-Orem, Utah	876	34.5	1.8	(31.0–38.1)
Raleigh, North Carolina	369	33.9	3.0	(28.0–39.9)
Rapid City, South Dakota	611	44.2	2.9	(38.6–49.9)
Reno, Nevada	1,221	32.7	1.8	(29.1–36.2)
Richmond, Virginia	883	38.5	2.1	(34.4–42.6)
Riverside-San Bernardino-Ontario, California	850	40.5	2.1	(36.5–44.6)
Rochester, New York	344	45.0	3.4	(38.3–51.6)
Rockingham County-Strafford County, New Hampshire^§^	1,195	41.5	1.8	(38.0–44.9)
Sacramento-Roseville-Arden-Arcade, California	587	37.0	2.8	(31.6–42.4)
St. Louis, Missouri-Illinois	1,477	42.6	1.9	(38.9–46.3)
Salem, Oregon	394	45.1	3.2	(38.9–51.4)
Salina, Kansas	377	32.5	2.7	(27.1–37.8)
Salisbury, Maryland-Delaware	1,640	43.4	1.7	(40.0–46.7)
Salt Lake City, Utah	2,737	35.4	1.1	(33.2–37.5)
San Antonio-New Braunfels, Texas	626	37.2	2.4	(32.6–41.8)
San Francisco-Redwood City-South San Francisco, California^§^	321	31.4	3.3	(24.9–38.0)
San Jose-Sunnyvale-Santa Clara, California	353	32.5	3.2	(26.3–38.7)
San Juan-Carolina-Caguas, Puerto Rico	2,404	39.6	1.2	(37.2–42.0)
Scottsbluff, Nebraska	558	43.6	2.8	(38.1–49.1)
Scranton-Wilkes-Barre-Hazleton, Pennsylvania	432	45.8	3.0	(40.0–51.6)
Seattle-Bellevue-Everett, Washington^§^	2,499	37.5	1.2	(35.2–39.9)
Shreveport-Bossier City, Louisiana	445	41.5	3.1	(35.5–47.5)
Silver Spring-Frederick-Rockville, Maryland^§^	1,710	34.4	1.4	(31.5–37.2)
Sioux City, Iowa-Nebraska-South Dakota	768	29.2	2.9	(23.4–35.0)
Sioux Falls, South Dakota	604	34.3	2.5	(29.3–39.3)
Spartanburg, South Carolina	465	48.8	3.4	(42.3–55.4)
Spokane-Spokane Valley, Washington	640	42.6	2.6	(37.6–47.6)
Springfield, Massachusetts	1,167	40.5	2.2	(36.2–44.7)
Tallahassee, Florida	1,423	38.9	2.3	(34.3–43.4)
Tampa-St. Petersburg-Clearwater, Florida	1,607	41.1	1.8	(37.7–44.5)
Toledo, Ohio	735	48.2	2.5	(43.3–53.1)
Topeka, Kansas	1,761	39.4	1.4	(36.8–42.1)
Tulsa, Oklahoma	1,465	39.5	1.6	(36.4–42.6)
Virginia Beach-Norfolk-Newport News, Virginia-North Carolina	1,142	39.7	1.8	(36.1–43.3)
Warren-Troy-Farmington Hills, Michigan^§^	1,615	42.5	1.5	(39.6–45.3)
Washington-Arlington-Alexandria, District of Columbia-Virginia-Maryland-West Virginia^§^	6,199	36.3	1.1	(34.1–38.4)
Wichita, Kansas	3,445	36.6	1.0	(34.7–38.5)
Wilmington, Delaware-Maryland-New Jersey^§^	2,279	40.1	1.3	(37.6–42.7)
Winston-Salem, North Carolina	527	43.5	2.7	(38.1–48.9)
Worcester, Massachusetts-Connecticut	1,926	40.8	1.8	(37.3–44.4)
*Median*		*39.4*		
*Range*		*27.6–52.4*		

**Abbreviations:** CI = confidence interval; MMSA = micropolitan and metropolitan statistical areas; SE = standard error.

* Age adjusted to the 2000 U.S. standard population.

^†^ Including arthritis, rheumatoid arthritis, gout, lupus, or fibromyalgia.

^§^ Metropolitan division.

**TABLE 47:** Age-adjusted* prevalence estimates of adults aged ≥45 years who have ever been told by a health professional they have some form of arthritis,^†^ by state/territory — Behavioral Risk Factor Surveillance System, United States, 2014

State/Territory	Sample size	%	SE	95% CI
Alabama	6,457	50.5	0.8	(48.9–52.2)
Alaska	2,855	36.7	1.3	(34.3–39.2)
Arizona	11,683	38.0	0.7	(36.7–39.3)
Arkansas	4,182	46.0	1.1	(43.7–48.2)
California	5,302	34.1	0.8	(32.5–35.7)
Colorado	9,458	37.4	0.6	(36.2–38.5)
Connecticut	5,850	36.4	0.8	(34.8–38.0)
Delaware	3,249	41.3	1.1	(39.1–43.5)
District of Columbia	3,032	36.1	1.3	(33.6–38.6)
Florida	7,529	39.5	0.8	(38.0–41.0)
Georgia	4,638	41.8	0.9	(40.0–43.6)
Hawaii	4,807	31.2	1.0	(29.3–33.1)
Idaho	3,919	37.7	1.1	(35.6–39.8)
Illinois	3,558	39.9	1.0	(37.8–41.9)
Indiana	8,602	43.5	0.7	(42.2–44.8)
Iowa	6,043	38.4	0.8	(36.9–39.9)
Kansas	9,639	39.8	0.6	(38.7–41.0)
Kentucky	8,581	49.2	0.9	(47.5–51.0)
Louisiana	4,743	41.8	0.9	(40.1–43.5)
Maine	7,093	43.8	0.8	(42.3–45.4)
Maryland	9,764	40.0	0.8	(38.4–41.5)
Massachusetts	11,655	41.9	0.7	(40.5–43.3)
Michigan	6,210	46.5	0.8	(45.0–48.0)
Minnesota	10,987	33.9	0.5	(32.9–34.9)
Mississippi	3,152	46.1	1.2	(43.8–48.5)
Missouri	5,395	42.1	1.0	(40.2–44.0)
Montana	5,697	37.9	0.9	(36.0–39.7)
Nebraska	16,061	38.0	0.6	(36.9–39.1)
Nevada	2,636	36.5	1.4	(33.8–39.3)
New Hampshire	4,699	39.4	0.9	(37.6–41.3)
New Jersey	9,243	35.1	0.7	(33.7–36.6)
New Mexico	6,482	38.0	0.9	(36.3–39.8)
New York	4,684	38.2	0.8	(36.5–39.8)
North Carolina	4,923	43.1	0.8	(41.4–44.7)
North Dakota	5,815	40.4	0.9	(38.5–42.2)
Ohio	8,379	44.9	0.8	(43.3–46.5)
Oklahoma	6,095	41.8	0.7	(40.3–43.2)
Oregon	3,881	38.2	1.0	(36.2–40.2)
Pennsylvania	8,210	44.7	0.7	(43.3–46.2)
Rhode Island	4,912	41.9	0.9	(40.1–43.6)
South Carolina	8,087	44.6	0.7	(43.2–46.1)
South Dakota	5,207	39.6	1.1	(37.3–41.8)
Tennessee	3,964	47.8	1.1	(45.7–49.9)
Texas	10,621	34.3	0.8	(32.7–35.8)
Utah	8,548	36.8	0.6	(35.6–38.0)
Vermont	4,589	40.4	0.8	(38.8–42.1)
Virginia	6,751	41.5	0.8	(39.9–43.0)
Washington	7,413	39.7	0.7	(38.3–41.2)
West Virginia	4,508	54.7	0.9	(53.0–56.5)
Wisconsin	5,067	38.9	0.9	(37.1–40.8)
Wyoming	5,163	37.9	1.0	(35.9–40.0)
Guam	1,200	31.8	1.9	(28.1–35.5)
Puerto Rico	4,072	40.4	0.9	(38.6–42.3)
*Median*		*39.8*		
*Range*		*31.2–54.7*		

**Abbreviations:** CI = confidence interval; SE = standard error.

* Age adjusted to the 2000 U.S. standard population.

^†^ Including arthritis, rheumatoid arthritis, gout, lupus, or fibromyalgia.

**TABLE 48:** Age-adjusted* prevalence estimates of adults aged ≥45 years who have ever been told by a health professional they have some form of arthritis,^†^ by metropolitan and micropolitan statistical area — Behavioral Risk Factor Surveillance System, United States, 2014

MMSA	Sample size	%	SE	95% CI
Aberdeen, South Dakota	615	45.5	2.7	(40.2–50.9)
Aguadilla-Isabela, Puerto Rico	541	40.9	3.3	(34.4–47.3)
Albuquerque, New Mexico	1,786	37.6	1.7	(34.3–40.9)
Allentown-Bethlehem-Easton, Pennsylvania-New Jersey	1,089	42.4	2.4	(37.7–47.1)
Anchorage, Alaska	1,771	35.9	1.8	(32.3–39.5)
Atlanta-Sandy Springs-Roswell, Georgia	2,767	36.7	1.4	(34.1–39.4)
Augusta-Richmond County, Georgia-South Carolina	882	44.4	3.4	(37.8–51.0)
Austin-Round Rock, Texas	2,241	31.2	1.5	(28.2–34.1)
Baltimore-Columbia-Towson, Maryland	4,597	40.2	1.3	(37.7–42.7)
Baton Rouge, Louisiana	919	37.8	2.2	(33.5–42.1)
Berlin, New Hampshire-Vermont	538	41.7	3.0	(35.8–47.5)
Billings, Montana	809	37.3	2.4	(32.5–42.1)
Birmingham-Hoover, Alabama	1,570	46.2	1.9	(42.5–49.9)
Bismarck, North Dakota	1,032	40.1	2.3	(35.6–44.5)
Boise City, Idaho	1,345	35.9	2.0	(31.9–39.9)
Boston, Massachusetts^§^	4,529	39.1	1.2	(36.7–41.5)
Burlington-South Burlington, Vermont	1,979	39.6	1.6	(36.5–42.6)
Cambridge-Newton-Framingham, Massachusetts^§^	5,149	40.2	1.2	(37.8–42.5)
Camden, New Jersey^§^	1,711	39.9	1.7	(36.6–43.2)
Cedar Rapids, Iowa	639	35.7	2.4	(30.9–40.5)
Charleston, West Virginia	872	53.7	2.5	(48.8–58.6)
Charleston-North Charleston, South Carolina	1,398	41.3	1.9	(37.5–45.0)
Charlotte-Concord-Gastonia, North Carolina-South Carolina	2,147	41.1	1.7	(37.7–44.4)
Chicago-Naperville-Elgin, Illinois-Indiana-Wisconsin	4,118	39.4	1.3	(36.9–41.9)
Cincinnati, Ohio-Kentucky-Indiana	2,037	43.9	1.9	(40.2–47.6)
Claremont-Lebanon, New Hampshire-Vermont	1,679	41.6	1.8	(38.2–45.1)
Cleveland-Elyria, Ohio	961	43.8	2.4	(39.0–48.5)
College Station-Bryan, Texas	572	28.4	3.9	(20.8–36.0)
Colorado Springs, Colorado	1,292	38.5	1.9	(34.8–42.3)
Columbia, South Carolina	1,201	43.5	2.1	(39.4–47.6)
Columbus, Ohio	1,654	44.5	1.9	(40.7–48.3)
Corpus Christi, Texas	619	39.9	3.6	(32.7–47.0)
Dallas-Plano-Irving, Texas^§^	1,290	32.6	2.4	(27.9–37.3)
Dayton, Ohio	562	47.1	3.3	(40.5–53.6)
Denver-Aurora-Lakewood, Colorado	5,760	36.5	0.9	(34.7–38.2)
Des Moines-West Des Moines, Iowa	1,353	43.2	2.0	(39.4–47.1)
Duluth, Minnesota-Wisconsin	945	45.0	2.7	(39.8–50.2)
El Paso, Texas	721	37.0	2.8	(31.5–42.4)
Evansville, Indiana-Kentucky	656	48.0	2.9	(42.4–53.6)
Fargo, North Dakota-Minnesota	1,147	39.5	2.3	(35.0–43.9)
Fayetteville-Springdale-Rogers, Arkansas-Missouri	811	42.5	2.9	(36.8–48.1)
Fort Wayne, Indiana	855	39.8	2.4	(35.1–44.5)
Fort Worth-Arlington, Texas^§^	755	32.5	2.5	(27.6–37.3)
Grand Island, Nebraska	1,051	41.8	2.3	(37.3–46.3)
Grand Rapids-Wyoming, Michigan	894	48.7	2.5	(43.8–53.7)
Greensboro-High Point, North Carolina	521	39.3	2.8	(33.8–44.7)
Greenville-Anderson-Mauldin, South Carolina	1,484	44.4	2.1	(40.3–48.5)
Hagerstown-Martinsburg, Maryland-West Virginia	782	49.5	2.7	(44.2–54.9)
Hartford-West Hartford-East Hartford, Connecticut	2,627	37.3	1.4	(34.6–40.1)
Hilton Head Island-Bluffton-Beaufort, South Carolina	551	43.3	3.2	(36.9–49.6)
Houston-The Woodlands-Sugar Land, Texas	2,148	32.3	2.1	(28.2–36.4)
Huntington-Ashland, West Virginia-Kentucky-Ohio	1,243	50.5	2.0	(46.5–54.4)
Idaho Falls, Idaho	516	37.9	3.1	(31.8–44.0)
Indianapolis-Carmel-Anderson, Indiana	3,587	43.6	1.2	(41.2–46.0)
Jacksonville, Florida	670	42.4	2.8	(37.0–47.8)
Kansas City, Missouri-Kansas	4,857	38.7	1.3	(36.1–41.3)
Kingsport-Bristol-Bristol, Tennessee-Virginia	503	54.1	3.8	(46.6–61.6)
Knoxville, Tennessee	559	49.7	3.1	(43.6–55.8)
Lafayette, Louisiana	562	39.3	2.8	(33.9–44.7)
Lexington-Fayette, Kentucky	622	42.2	3.4	(35.6–48.9)
Lincoln, Nebraska	1,995	36.8	1.6	(33.6–39.9)
Little Rock-North Little Rock-Conway, Arkansas	1,179	43.2	2.3	(38.6–47.8)
Logan, Utah-Idaho	623	36.7	2.8	(31.1–42.2)
Los Angeles-Long Beach-Anaheim, California	2,431	31.6	1.5	(28.6–34.6)
Louisville-Jefferson County, Kentucky-Indiana	2,449	46.9	1.9	(43.1–50.6)
Madison, Wisconsin	549	36.5	3.0	(30.6–42.4)
Memphis, Tennessee-Mississippi-Arkansas	879	41.9	2.8	(36.5–47.4)
Miami-Fort Lauderdale-West Palm Beach, Florida	2,205	34.4	1.6	(31.3–37.4)
Milwaukee-Waukesha-West Allis, Wisconsin	1,352	39.2	2.0	(35.2–43.2)
Minneapolis-St. Paul-Bloomington, Minnesota-Wisconsin	8,724	32.4	0.7	(31.0–33.8)
Minot, North Dakota	592	41.2	3.1	(35.2–47.2)
Montgomery, Alabama	514	54.7	3.3	(48.2–61.2)
Montgomery County-Bucks County-Chester County, Pennsylvania^§^	801	37.8	2.1	(33.6–42.0)
Myrtle Beach-Conway-North Myrtle Beach, South Carolina-North Carolina	992	46.0	2.3	(41.4–50.5)
Nashville-Davidson County-Murfreesboro-Franklin, Tennessee	798	43.0	2.6	(37.9–48.2)
Nassau County-Suffolk County, New York^§^	766	35.7	2.3	(31.3–40.2)
Newark, New Jersey-Pennsylvania^§^	4,122	32.3	1.2	(30.0–34.7)
New Orleans-Metairie, Louisiana	1,905	38.5	1.6	(35.4–41.6)
New York-Jersey City-White Plains, New York-New Jersey^§^	7,495	34.8	0.9	(33.0–36.7)
Norfolk, Nebraska	995	36.2	2.0	(32.3–40.2)
North Platte, Nebraska	962	43.6	2.3	(39.2–48.1)
North Port-Sarasota-Bradenton, Florida	512	38.6	3.3	(32.2–45.0)
Oakland-Hayward-Berkeley, California^§^	697	32.3	2.7	(27.1–37.5)
Ogden-Clearfield, Utah	2,913	37.4	1.3	(34.8–40.0)
Oklahoma City, Oklahoma	2,422	39.1	1.4	(36.3–41.8)
Omaha-Council Bluffs, Nebraska-Iowa	4,853	37.8	1.1	(35.7–40.0)
Orlando-Kissimmee-Sanford, Florida	952	40.3	2.4	(35.5–45.0)
Philadelphia, Pennsylvania^§^	1,513	45.2	2.0	(41.2–49.2)
Phoenix-Mesa-Scottsdale, Arizona	9,356	37.6	0.8	(36.1–39.1)
Pittsburgh, Pennsylvania	2,408	47.3	1.5	(44.4–50.2)
Ponce, Puerto Rico	529	40.8	3.1	(34.8–46.8)
Portland-South Portland, Maine	2,743	42.3	1.4	(39.5–45.0)
Portland-Vancouver-Hillsboro, Oregon-Washington	2,833	36.4	1.3	(33.9–38.9)
Providence-Warwick, Rhode Island-Massachusetts	8,061	43.8	1.0	(41.8–45.9)
Provo-Orem, Utah	2,143	36.5	1.7	(33.1–39.8)
Raleigh, North Carolina	721	36.3	2.5	(31.4–41.2)
Rapid City, South Dakota	1,414	41.8	2.3	(37.3–46.3)
Reno, Nevada	1,192	37.2	1.9	(33.4–41.0)
Richmond, Virginia	1,456	41.6	2.0	(37.6–45.5)
Riverside-San Bernardino-Ontario, California	938	36.4	2.4	(31.8–41.1)
Roanoke, Virginia	531	38.6	2.9	(32.8–44.3)
Rochester, Minnesota	695	31.4	2.3	(26.9–35.9)
Rockingham County-Strafford County, New Hampshire^§^	1,434	37.7	1.7	(34.3–41.1)
Sacramento-Roseville-Arden-Arcade, California	642	36.8	3.0	(31.0–42.6)
St. Cloud, Minnesota	559	34.4	2.8	(28.9–39.8)
St. Louis, Missouri-Illinois	1,924	40.1	1.8	(36.5–43.7)
Salisbury, Maryland-Delaware	1,956	44.3	1.8	(40.8–47.8)
Salt Lake City, Utah	5,396	35.9	1.0	(33.9–37.9)
San Antonio-New Braunfels, Texas	2,262	33.7	1.5	(30.8–36.5)
San Juan-Carolina-Caguas, Puerto Rico	3,735	40.2	1.2	(37.9–42.6)
Scottsbluff, Nebraska	903	41.7	2.6	(36.7–46.7)
Seattle-Bellevue-Everett, Washington^§^	3,681	37.5	1.1	(35.3–39.8)
Shreveport-Bossier City, Louisiana	548	44.6	3.1	(38.5–50.7)
Silver Spring-Frederick-Rockville, Maryland^§^	2,365	33.7	1.7	(30.3–37.0)
Sioux City, Iowa-Nebraska-South Dakota	1,139	35.3	3.0	(29.4–41.1)
Sioux Falls, South Dakota	1,338	37.6	2.2	(33.3–41.9)
Spartanburg, South Carolina	560	50.0	3.2	(43.7–56.3)
Spokane-Spokane Valley, Washington	723	41.8	2.6	(36.7–46.9)
Springfield, Massachusetts	1,094	51.5	2.7	(46.1–56.8)
Tampa-St. Petersburg-Clearwater, Florida	1,574	41.3	1.9	(37.6–45.0)
Toledo, Ohio	649	46.7	2.8	(41.2–52.2)
Topeka, Kansas	1,434	40.8	1.8	(37.3–44.3)
Tulsa, Oklahoma	2,021	38.1	1.4	(35.3–40.8)
Tuscaloosa, Alabama	719	47.8	2.7	(42.4–53.1)
Virginia Beach-Norfolk-Newport News, Virginia-North Carolina	1,876	42.3	1.7	(39.0–45.7)
Warren-Troy-Farmington Hills, Michigan^§^	2,100	43.2	1.5	(40.2–46.1)
Washington-Arlington-Alexandria, District of Columbia-Virginia-Maryland-West Virginia^§^	8,238	38.9	1.1	(36.7–41.1)
Wichita, Kansas	2,725	41.0	1.3	(38.5–43.5)
Wichita Falls, Texas	543	45.0	3.0	(39.1–50.8)
Wilmington, Delaware-Maryland-New Jersey^§^	2,749	39.8	1.4	(37.1–42.5)
Worcester, Massachusetts-Connecticut	2,456	40.5	1.7	(37.2–43.8)
Youngstown-Warren-Boardman, Ohio-Pennsylvania	522	44.9	3.1	(38.7–51.0)
*Median*		*40.1*		
*Range*		*28.4–54.7*		

**Abbreviations:** CI = confidence interval; MMSA = metropolitan and micropolitan statistical areas; SE = standard error.

* Age adjusted to the 2000 U.S. standard population.

^†^ Including arthritis, rheumatoid arthritis, gout, lupus, or fibromyalgia.

^§^ Metropolitan division.

#### Depression

Respondents were asked if they had ever been told by a doctor, nurse, or other health professional they have a depressive disorder, including depression, major depression, dysthymia, or minor depression. In 2013, age-adjusted prevalence estimates of depression ranged from 8.6% in Guam to 26.8% in Oregon (median: 18.6%) (Table 49). Among selected MMSA, age-adjusted prevalence estimates ranged from 7.7% in San Jose-Sunnyvale-Santa Clara, California, to 28.5% in Fort Smith, Arkansas-Oklahoma (median: 18.7%) (Table 50). In 2014, age-adjusted prevalence estimates ranged from 8.9% in Guam to 24.2% in Maine (median: 18.8%) (Table 51). Among selected MMSA, age-adjusted prevalence estimates ranged from 10.5% in Riverside-San Bernardino-Ontario, California, to 29.5% in Springfield, Massachusetts (median: 18.8%) (Table 52).

**TABLE 49:** Age-adjusted* prevalence estimates of adults aged ≥18 years who have ever been told by a health professional they have a depressive disorder,^†^ by state/territory — Behavioral Risk Factor Surveillance System, United States, 2013

State/Territory	Sample size	%	SE	95% CI
Alabama	6,467	21.4	0.7	(19.9–22.8)
Alaska	4,561	16.1	0.8	(14.6–17.6)
Arizona	4,223	17.9	1.0	(15.9–19.9)
Arkansas	5,240	23.2	0.9	(21.4–24.9)
California	11,495	12.9	0.4	(12.0–13.7)
Colorado	13,572	18.0	0.5	(17.1–18.9)
Connecticut	7,673	17.2	0.6	(16.0–18.5)
Delaware	5,181	17.6	0.8	(16.0–19.1)
District of Columbia	4,903	20.8	0.9	(18.9–22.6)
Florida	33,983	16.7	0.5	(15.8–17.6)
Georgia	8,095	17.2	0.6	(16.0–18.3)
Hawaii	7,834	11.4	0.5	(10.4–12.4)
Idaho	5,604	20.8	0.8	(19.2–22.4)
Illinois	5,599	15.4	0.7	(14.0–16.7)
Indiana	10,289	19.5	0.6	(18.4–20.6)
Iowa	8,138	19.4	0.6	(18.1–20.6)
Kansas	23,166	18.1	0.3	(17.4–18.7)
Kentucky	10,973	20.1	0.6	(18.8–21.3)
Louisiana	5,226	18.6	0.9	(16.8–20.4)
Maine	8,070	23.8	0.7	(22.4–25.2)
Maryland	12,957	15.8	0.5	(14.8–16.8)
Massachusetts	14,997	19.7	0.6	(18.6–20.8)
Michigan	12,704	21.2	0.5	(20.1–22.2)
Minnesota	14,284	18.4	0.6	(17.3–19.6)
Mississippi	7,415	19.1	0.7	(17.7–20.4)
Missouri	7,088	21.3	0.7	(19.8–22.7)
Montana	9,652	21.1	0.6	(19.9–22.4)
Nebraska	17,065	18.3	0.5	(17.3–19.4)
Nevada	5,075	17.6	1.0	(15.6–19.5)
New Hampshire	6,430	22.4	0.8	(20.9–24.0)
New Jersey	13,339	13.7	0.5	(12.8–14.6)
New Mexico	9,286	19.4	0.6	(18.1–20.6)
New York	8,923	16.0	0.6	(14.9–17.1)
North Carolina	8,825	18.5	0.6	(17.4–19.6)
North Dakota	7,767	16.9	0.7	(15.6–18.2)
Ohio	11,913	20.3	0.6	(19.2–21.5)
Oklahoma	8,214	23.3	0.6	(22.1–24.6)
Oregon	5,913	26.8	0.8	(25.1–28.4)
Pennsylvania	11,363	18.2	0.5	(17.2–19.2)
Rhode Island	6,509	22.3	0.8	(20.8–23.8)
South Carolina	10,621	19.4	0.6	(18.3–20.6)
South Dakota	6,854	14.6	0.7	(13.2–16.0)
Tennessee	5,786	19.0	0.8	(17.5–20.5)
Texas	10,837	15.9	0.5	(14.8–16.9)
Utah	12,718	21.8	0.5	(20.9–22.7)
Vermont	6,360	23.1	0.8	(21.6–24.7)
Virginia	8,423	16.3	0.5	(15.2–17.3)
Washington	11,112	23.4	0.6	(22.2–24.5)
West Virginia	5,876	22.0	0.7	(20.7–23.3)
Wisconsin	6,565	18.1	0.8	(16.5–19.7)
Wyoming	6,435	18.7	0.7	(17.2–20.1)
Guam	1,887	8.6	0.8	(7.0–10.2)
Puerto Rico	6,000	18.3	0.6	(17.0–19.5)
*Median*		*18.6*		
*Range*		*8.6–26.8*		

**Abbreviations:** CI = confidence interval; SE = standard error.

* Age adjusted to the 2000 U.S. standard population.

^†^ Including depression, major depression, dysthymia, or minor depression.

**TABLE 50:** Age-adjusted* prevalence estimates of adults aged ≥18 years who have ever been told by a health professional they have a depressive disorder,^† ^by metropolitan and micropolitan statistical area — Behavioral Risk Factor Surveillance System, United States, 2013

MMSA	Sample size	%	SE	95% CI
Aguadilla-Isabela, Puerto Rico	593	16.5	1.9	(12.8–20.2)
Akron, Ohio	690	23.4	2.8	(18.0–28.8)
Albuquerque, New Mexico	2,078	20.3	1.1	(18.0–22.5)
Allentown-Bethlehem-Easton, Pennsylvania-New Jersey	1,028	19.0	2.0	(15.1–22.9)
Anchorage, Alaska	1,516	16.2	1.2	(13.9–18.6)
Atlanta-Sandy Springs-Roswell, Georgia	3,498	15.4	0.8	(13.8–17.0)
Augusta-Richmond County, Georgia-South Carolina	907	16.5	1.9	(12.8–20.3)
Austin-Round Rock, Texas	929	17.0	1.6	(13.8–20.2)
Baltimore-Columbia-Towson, Maryland	4,753	17.4	0.8	(15.8–19.1)
Baton Rouge, Louisiana	923	15.8	1.8	(12.2–19.3)
Billings, Montana	816	23.7	1.7	(20.3–27.1)
Birmingham-Hoover, Alabama	1,348	21.2	1.5	(18.3–24.0)
Bismarck, North Dakota	1,034	16.7	1.5	(13.8–19.6)
Boise City, Idaho	1,479	20.8	1.5	(18.0–23.7)
Boston, Massachusetts^§^	4,041	17.9	0.9	(16.0–19.7)
Buffalo-Cheektowaga-Niagara Falls, New York	502	13.6	1.9	(9.9–17.3)
Burlington-South Burlington, Vermont	1,624	23.2	1.4	(20.5–25.9)
Cambridge-Newton-Framingham, Massachusetts^§^	4,878	17.6	0.9	(15.9–19.4)
Camden, New Jersey^§^	1,860	17.6	1.3	(15.1–20.0)
Cedar Rapids, Iowa	647	21.1	2.3	(16.5–25.7)
Charleston, West Virginia	817	24.0	1.9	(20.3–27.8)
Charleston-North Charleston, South Carolina	1,539	18.5	1.4	(15.8–21.2)
Charlotte-Concord-Gastonia, North Carolina-South Carolina	1,945	18.0	1.1	(15.8–20.3)
Chattanooga, Tennessee-Georgia	575	19.5	2.6	(14.4–24.7)
Chicago-Naperville-Elgin, Illinois-Indiana-Wisconsin	3,337	14.9	0.9	(13.2–16.7)
Cincinnati, Ohio-Kentucky-Indiana	2,601	20.1	1.2	(17.7–22.5)
Claremont-Lebanon, New Hampshire-Vermont	1,675	23.3	1.8	(19.8–26.7)
Cleveland-Elyria, Ohio	1,104	17.0	1.7	(13.7–20.3)
Colorado Springs, Colorado	1,369	20.8	1.4	(18.1–23.6)
Columbia, South Carolina	1,435	18.7	1.5	(15.7–21.7)
Columbus, Ohio	1,853	23.3	1.3	(20.8–25.9)
Crestview-Fort Walton Beach-Destin, Florida	1,076	18.7	1.8	(15.1–22.3)
Dallas-Plano-Irving, Texas^§^	899	13.6	1.3	(11.1–16.2)
Davenport-Moline-Rock Island, Iowa-Illinois	671	18.0	2.2	(13.6–22.4)
Dayton, Ohio	838	20.6	2.2	(16.3–24.9)
Deltona-Daytona Beach-Ormond Beach, Florida	1,108	15.8	2.0	(11.8–19.7)
Denver-Aurora-Lakewood, Colorado	5,685	17.6	0.6	(16.4–18.9)
Des Moines-West Des Moines, Iowa	1,343	19.8	1.5	(16.9–22.7)
Duluth, Minnesota-Wisconsin	700	18.0	2.3	(13.4–22.6)
Durham-Chapel Hill, North Carolina	614	15.7	2.0	(11.7–19.7)
El Paso, Texas	752	14.5	1.7	(11.2–17.8)
Evansville, Indiana-Kentucky	574	22.4	2.4	(17.7–27.1)
Fargo, North Dakota-Minnesota	1,179	21.5	1.7	(18.1–24.9)
Fayetteville-Springdale-Rogers, Arkansas-Missouri	817	22.8	2.2	(18.5–27.0)
Fort Smith, Arkansas-Oklahoma	499	28.5	2.8	(22.9–34.0)
Fort Wayne, Indiana	779	17.8	1.9	(14.1–21.4)
Fort Worth-Arlington, Texas^§^	808	18.5	1.9	(14.8–22.1)
Gainesville, Florida	1,027	17.5	2.0	(13.7–21.4)
Grand Forks, North Dakota-Minnesota	502	18.1	2.8	(12.7–23.5)
Grand Island, Nebraska	797	18.9	2.0	(15.0–22.8)
Grand Rapids-Wyoming, Michigan	1,340	21.3	1.5	(18.4–24.3)
Greensboro-High Point, North Carolina	663	21.1	2.1	(16.9–25.2)
Greenville-Anderson-Mauldin, South Carolina	1,340	22.5	1.7	(19.1–25.8)
Gulfport-Biloxi-Pascagoula, Mississippi	768	24.5	2.1	(20.4–28.7)
Hagerstown-Martinsburg, Maryland-West Virginia	765	24.7	2.7	(19.4–30.1)
Hartford-West Hartford-East Hartford, Connecticut	2,825	19.0	1.1	(16.7–21.2)
Hilton Head Island-Bluffton-Beaufort, South Carolina	821	12.4	1.8	(9.0–15.9)
Houston-The Woodlands-Sugar Land, Texas	1,382	13.7	1.3	(11.2–16.3)
Huntington-Ashland, West Virginia-Kentucky-Ohio	1,170	25.1	1.7	(21.8–28.3)
Idaho Falls, Idaho	506	28.2	2.9	(22.6–33.8)
Indianapolis-Carmel-Anderson, Indiana	2,529	20.8	1.1	(18.7–22.9)
Jackson, Mississippi	798	15.3	1.6	(12.3–18.4)
Jacksonville, Florida	2,859	20.2	1.3	(17.7–22.6)
Kansas City, Missouri-Kansas	7,389	19.3	0.9	(17.4–21.1)
Kingsport-Bristol-Bristol, Tennessee-Virginia	532	25.0	3.3	(18.5–31.6)
Knoxville, Tennessee	649	21.0	2.0	(17.0–25.0)
Lansing-East Lansing, Michigan	683	20.4	1.9	(16.6–24.2)
Lexington-Fayette, Kentucky	636	17.3	1.8	(13.8–20.8)
Lincoln, Nebraska	1,862	17.3	1.0	(15.3–19.2)
Little Rock-North Little Rock-Conway, Arkansas	1,140	22.6	1.8	(19.2–26.1)
Logan, Utah-Idaho	641	20.2	2.0	(16.3–24.1)
Los Angeles-Long Beach-Anaheim, California	3,034	11.9	0.8	(10.3–13.5)
Louisville-Jefferson County, Kentucky-Indiana	2,144	18.3	1.4	(15.7–21.0)
Lubbock, Texas	530	20.1	2.6	(15.0–25.2)
Manhattan, Kansas	662	23.2	1.9	(19.4–27.0)
Memphis, Tennessee-Mississippi-Arkansas	1,202	12.4	1.3	(10.0–14.9)
Miami-Fort Lauderdale-West Palm Beach, Florida	2,194	12.9	1.1	(10.7–15.0)
Milwaukee-Waukesha-West Allis, Wisconsin	1,266	20.3	1.9	(16.6–24.1)
Minneapolis-St. Paul-Bloomington, Minnesota-Wisconsin	9,100	18.1	0.7	(16.6–19.5)
Minot, North Dakota	651	12.4	1.7	(9.0–15.8)
Montgomery County-Bucks County-Chester County, Pennsylvania^§^	970	16.5	1.5	(13.6–19.5)
Myrtle Beach-Conway-North Myrtle Beach, South Carolina-North Carolina	767	19.7	2.1	(15.6–23.7)
Nashville-Davidson County-Murfreesboro-Franklin, Tennessee	1,063	16.7	1.5	(13.7–19.7)
Nassau County-Suffolk County, New York^§^	937	13.8	1.5	(10.9–16.6)
Newark, New Jersey-Pennsylvania^§^	4,106	12.6	0.8	(11.0–14.1)
New Orleans-Metairie, Louisiana	1,283	17.9	1.8	(14.4–21.5)
New York-Jersey City-White Plains, New York-New Jersey^§^	8,912	13.8	0.6	(12.7–14.9)
Norfolk, Nebraska	674	19.0	1.8	(15.4–22.6)
North Platte, Nebraska	713	23.1	2.4	(18.4–27.8)
North Port-Sarasota-Bradenton, Florida	1,087	22.7	2.1	(18.5–26.8)
Oakland-Hayward-Berkeley, California^§^	699	12.1	1.4	(9.4–14.9)
Ogden-Clearfield, Utah	2,443	22.5	1.0	(20.5–24.4)
Oklahoma City, Oklahoma	2,634	20.1	1.0	(18.1–22.0)
Omaha-Council Bluffs, Nebraska-Iowa	3,115	20.2	1.0	(18.2–22.2)
Orlando-Kissimmee-Sanford, Florida	2,267	16.6	1.2	(14.3–19.0)
Panama City, Florida	1,020	19.8	2.0	(16.0–23.7)
Pensacola-Ferry Pass-Brent, Florida	1,307	18.2	1.3	(15.6–20.8)
Philadelphia, Pennsylvania^§^	1,768	19.5	1.4	(16.7–22.2)
Phoenix-Mesa-Scottsdale, Arizona	1,544	17.2	1.4	(14.5–19.9)
Pittsburgh, Pennsylvania	2,352	16.3	1.1	(14.2–18.4)
Ponce, Puerto Rico	531	17.2	2.3	(12.6–21.8)
Portland-South Portland, Maine	2,634	23.0	1.2	(20.7–25.4)
Portland-Vancouver-Hillsboro, Oregon-Washington	3,226	25.7	1.1	(23.6–27.8)
Port St. Lucie, Florida	1,023	15.2	1.8	(11.6–18.8)
Providence-Warwick, Rhode Island-Massachusetts	8,279	23.1	0.9	(21.3–24.9)
Provo-Orem, Utah	1,856	20.2	1.1	(18.0–22.4)
Raleigh, North Carolina	675	14.3	1.5	(11.4–17.3)
Rapid City, South Dakota	868	18.7	1.9	(15.0–22.4)
Reno, Nevada	1,803	15.6	1.2	(13.3–17.9)
Richmond, Virginia	1,306	18.7	1.5	(15.9–21.6)
Riverside-San Bernardino-Ontario, California	1,369	13.0	1.1	(10.9–15.1)
Rochester, New York	507	21.0	2.5	(16.2–25.9)
Rockingham County-Strafford County, New Hampshire^§^	1,656	22.8	1.5	(19.8–25.8)
Sacramento-Roseville-Arden-Arcade, California	890	17.5	1.9	(13.8–21.1)
St. Louis, Missouri-Illinois	2,052	18.8	1.2	(16.4–21.2)
Salem, Oregon	523	26.7	2.8	(21.1–32.2)
Salina, Kansas	524	17.4	2.1	(13.3–21.5)
Salisbury, Maryland-Delaware	2,052	16.6	1.4	(13.8–19.4)
Salt Lake City, Utah	4,657	22.5	0.8	(21.0–24.0)
San Antonio-New Braunfels, Texas	936	17.6	1.6	(14.5–20.7)
San Francisco-Redwood City-South San Francisco, California^§^	538	16.2	2.1	(12.1–20.2)
San Jose-Sunnyvale-Santa Clara, California	625	7.7	1.2	(5.4–10.0)
San Juan-Carolina-Caguas, Puerto Rico	3,648	19.0	0.8	(17.4–20.6)
Scottsbluff, Nebraska	710	21.0	2.3	(16.5–25.6)
Scranton-Wilkes-Barre-Hazleton, Pennsylvania	562	22.4	2.7	(17.1–27.7)
Seattle-Bellevue-Everett, Washington^§^	3,757	23.1	0.9	(21.3–24.8)
Shreveport-Bossier City, Louisiana	572	15.9	2.1	(11.8–20.0)
Silver Spring-Frederick-Rockville, Maryland^§^	2,415	14.6	1.0	(12.7–16.6)
Sioux City, Iowa-Nebraska-South Dakota	1,052	15.3	2.3	(10.8–19.8)
Sioux Falls, South Dakota	1,006	13.1	1.4	(10.4–15.9)
Spartanburg, South Carolina	592	17.0	2.3	(12.6–21.5)
Spokane-Spokane Valley, Washington	859	21.1	2.0	(17.2–25.0)
Springfield, Massachusetts	1,575	23.2	1.9	(19.5–26.9)
Tallahassee, Florida	1,852	17.8	1.6	(14.6–21.0)
Tampa-St. Petersburg-Clearwater, Florida	2,202	19.5	1.3	(17.0–22.0)
Toledo, Ohio	993	19.6	1.9	(15.9–23.3)
Topeka, Kansas	2,397	20.9	1.1	(18.7–23.0)
Tulsa, Oklahoma	1,993	26.1	1.4	(23.3–28.9)
Virginia Beach-Norfolk-Newport News, Virginia-North Carolina	1,677	15.6	1.2	(13.3–17.9)
Warren-Troy-Farmington Hills, Michigan^§^	2,253	19.5	1.2	(17.2–21.9)
Washington-Arlington-Alexandria, District of Columbia-Virginia-Maryland-West Virginia^§^	8,983	14.0	0.7	(12.7–15.3)
Wichita, Kansas	4,910	19.0	0.7	(17.6–20.4)
Wilmington, Delaware-Maryland-New Jersey^§^	3,271	17.0	0.9	(15.2–18.7)
Winston-Salem, North Carolina	693	23.2	2.4	(18.6–27.9)
Worcester, Massachusetts-Connecticut	2,755	22.6	1.4	(19.9–25.3)
*Median*		*18.7*		
*Range*		*7.7–28.5*		

**Abbreviations:** CI = confidence interval; MMSA = metropolitan and micropolitan statistical areas; SE = standard error.

* Age adjusted to the 2000 U.S. standard population.

^†^ Including depression, major depression, dysthymia, or minor depression.

^§^ Metropolitan division.

**TABLE 51:** Age-adjusted* prevalence estimates of adults aged ≥18 years who have ever been told by a health professional they have a depressive disorder,^†^ by state/territory — Behavioral Risk Factor Surveillance System, United States, 2014

State/Territory	Sample size	%	SE	95% CI
Alabama	8,611	21.1	0.6	(19.9–22.4)
Alaska	4,373	15.4	0.8	(13.9–16.9)
Arizona	14,801	18.7	0.6	(17.6–19.8)
Arkansas	5,225	21.5	0.9	(19.6–23.3)
California	8,781	13.1	0.4	(12.3–14.0)
Colorado	13,331	16.9	0.4	(16.1–17.7)
Connecticut	7,914	18.2	0.7	(16.9–19.5)
Delaware	4,285	18.3	0.9	(16.4–20.1)
District of Columbia	4,054	18.1	1.0	(16.1–20.2)
Florida	9,766	15.9	0.5	(14.9–17.0)
Georgia	6,322	18.1	0.7	(16.8–19.4)
Hawaii	7,217	10.7	0.5	(9.7–11.7)
Idaho	5,468	19.6	0.8	(18.0–21.2)
Illinois	5,040	16.6	0.7	(15.2–18.1)
Indiana	11,473	20.8	0.6	(19.7–21.9)
Iowa	8,095	18.9	0.6	(17.7–20.2)
Kansas	13,676	18.6	0.4	(17.7–19.4)
Kentucky	11,144	23.9	0.8	(22.4–25.4)
Louisiana	6,753	18.5	0.6	(17.3–19.7)
Maine	9,094	24.2	0.7	(22.7–25.6)
Maryland	12,520	15.9	0.6	(14.7–17.2)
Massachusetts	15,545	21.7	0.6	(20.6–22.8)
Michigan	8,439	20.4	0.6	(19.2–21.7)
Minnesota	16,359	18.3	0.4	(17.5–19.0)
Mississippi	4,184	19.8	0.9	(18.0–21.6)
Missouri	7,046	21.9	0.8	(20.3–23.4)
Montana	7,460	20.6	0.8	(19.1–22.1)
Nebraska	22,346	17.8	0.5	(16.9–18.7)
Nevada	3,734	15.8	1.0	(13.9–17.7)
New Hampshire	6,159	21.7	0.9	(19.9–23.4)
New Jersey	12,977	13.1	0.5	(12.2–14.0)
New Mexico	8,919	21.0	0.7	(19.6–22.4)
New York	6,834	15.7	0.6	(14.6–16.9)
North Carolina	7,254	18.8	0.6	(17.7–19.9)
North Dakota	7,756	17.3	0.7	(15.9–18.8)
Ohio	10,893	21.1	0.7	(19.7–22.5)
Oklahoma	8,413	21.7	0.6	(20.5–22.9)
Oregon	5,197	24.1	0.8	(22.5–25.8)
Pennsylvania	10,949	19.9	0.6	(18.7–21.0)
Rhode Island	6,413	20.7	0.8	(19.1–22.3)
South Carolina	10,952	19.7	0.5	(18.6–20.7)
South Dakota	7,369	17.0	0.9	(15.3–18.7)
Tennessee	5,115	21.2	0.9	(19.5–22.9)
Texas	15,343	14.5	0.5	(13.6–15.4)
Utah	14,959	20.7	0.4	(19.9–21.4)
Vermont	6,443	22.1	0.7	(20.8–23.5)
Virginia	9,433	17.4	0.6	(16.3–18.5)
Washington	10,047	21.4	0.6	(20.3–22.6)
West Virginia	6,178	23.8	0.7	(22.4–25.2)
Wisconsin	7,020	16.9	0.7	(15.5–18.3)
Wyoming	6,380	19.0	0.9	(17.2–20.9)
Guam	2,506	8.9	0.8	(7.3–10.6)
Puerto Rico	5,981	17.9	0.6	(16.7–19.1)
*Median*		*18.8*		
*Range*		*8.9–24.2*		

**Abbreviations:** CI = confidence interval; SE = standard error.

* Age adjusted to the 2000 U.S. standard population.

^†^ Including depression, major depression, dysthymia, or minor depression.

**TABLE 52:** Age-adjusted* prevalence estimates of adults aged ≥18 years who have ever been told by a health professional they have a depressive disorder,^†^ by metropolitan and micropolitan statistical area — Behavioral Risk Factor Surveillance System, United States, 2014

MMSA	Sample size	%	SE	95% CI
Aberdeen, South Dakota	617	19.4	3.0	(13.5–25.3)
Aguadilla-Isabela, Puerto Rico	544	17.4	1.9	(13.8–21.1)
Albuquerque, New Mexico	1,787	22.6	1.4	(20.0–25.3)
Allentown-Bethlehem-Easton, Pennsylvania-New Jersey	1,092	18.6	2.1	(14.5–22.7)
Anchorage, Alaska	1,781	16.0	1.1	(13.7–18.2)
Atlanta-Sandy Springs-Roswell, Georgia	2,767	17.1	1.0	(15.2–19.0)
Augusta-Richmond County, Georgia-South Carolina	881	20.2	2.6	(15.0–25.3)
Austin-Round Rock, Texas	2,248	15.2	1.0	(13.3–17.1)
Baltimore-Columbia-Towson, Maryland	4,595	17.5	1.1	(15.4–19.6)
Baton Rouge, Louisiana	918	17.5	1.6	(14.4–20.5)
Berlin, New Hampshire-Vermont	540	18.8	3.2	(12.6–25.0)
Billings, Montana	805	23.3	2.0	(19.3–27.3)
Birmingham-Hoover, Alabama	1,570	22.2	1.4	(19.5–24.9)
Bismarck, North Dakota	1,035	17.0	1.9	(13.3–20.6)
Boise City, Idaho	1,353	20.7	1.6	(17.6–23.8)
Boston, Massachusetts^§^	4,517	20.9	1.1	(18.8–23.1)
Burlington-South Burlington, Vermont	1,970	22.4	1.2	(20.0–24.8)
Cambridge-Newton-Framingham, Massachusetts^§^	5,150	20.0	0.9	(18.2–21.8)
Camden, New Jersey^§^	1,710	14.7	1.3	(12.1–17.2)
Cedar Rapids, Iowa	640	22.4	2.3	(17.9–26.8)
Charleston, West Virginia	876	27.3	2.1	(23.2–31.4)
Charleston-North Charleston, South Carolina	1,393	19.8	1.5	(16.8–22.8)
Charlotte-Concord-Gastonia, North Carolina-South Carolina	2,146	18.1	1.1	(15.9–20.2)
Chicago-Naperville-Elgin, Illinois-Indiana-Wisconsin	4,116	15.8	0.9	(14.1–17.5)
Cincinnati, Ohio-Kentucky-Indiana	2,032	23.9	1.8	(20.5–27.4)
Claremont-Lebanon, New Hampshire-Vermont	1,678	22.6	1.8	(19.0–26.1)
Cleveland-Elyria, Ohio	964	20.6	2.1	(16.4–24.7)
College Station-Bryan, Texas	576	15.1	2.5	(10.2–20.0)
Colorado Springs, Colorado	1,291	18.9	1.4	(16.2–21.6)
Columbia, South Carolina	1,202	18.3	1.4	(15.6–21.1)
Columbus, Ohio	1,652	18.6	1.4	(15.9–21.2)
Corpus Christi, Texas	618	17.5	2.4	(12.7–22.2)
Dallas-Plano-Irving, Texas^§^	1,292	13.2	1.4	(10.5–15.9)
Dayton, Ohio	563	28.3	2.8	(22.7–33.8)
Denver-Aurora-Lakewood, Colorado	5,771	16.6	0.6	(15.4–17.8)
Des Moines-West Des Moines, Iowa	1,348	19.2	1.6	(16.1–22.3)
Duluth, Minnesota-Wisconsin	948	20.9	1.9	(17.3–24.6)
El Paso, Texas	720	14.8	1.6	(11.6–18.0)
Evansville, Indiana-Kentucky	655	25.5	3.1	(19.3–31.6)
Fargo, North Dakota-Minnesota	1,148	19.9	1.6	(16.7–23.1)
Fayetteville-Springdale-Rogers, Arkansas-Missouri	815	17.4	1.8	(13.8–20.9)
Fort Wayne, Indiana	858	21.6	2.2	(17.2–25.9)
Fort Worth-Arlington, Texas^§^	755	17.1	2.0	(13.1–21.1)
Grand Island, Nebraska	1,056	15.5	1.6	(12.3–18.6)
Grand Rapids-Wyoming, Michigan	895	21.0	2.0	(17.0–24.9)
Greensboro-High Point, North Carolina	521	17.6	2.1	(13.5–21.7)
Greenville-Anderson-Mauldin, South Carolina	1,485	19.5	1.3	(16.9–22.1)
Hagerstown-Martinsburg, Maryland-West Virginia	783	21.6	2.9	(15.9–27.4)
Hartford-West Hartford-East Hartford, Connecticut	2,634	17.8	1.0	(15.7–19.8)
Hilton Head Island-Bluffton-Beaufort, South Carolina	552	16.7	3.6	(9.7–23.8)
Houston-The Woodlands-Sugar Land, Texas	2,147	13.0	1.1	(10.8–15.1)
Huntington-Ashland, West Virginia-Kentucky-Ohio	1,243	25.2	1.9	(21.5–28.8)
Idaho Falls, Idaho	517	20.0	2.2	(15.6–24.4)
Indianapolis-Carmel-Anderson, Indiana	3,588	21.4	1.0	(19.4–23.4)
Jacksonville, Florida	668	14.7	1.9	(11.0–18.4)
Kansas City, Missouri-Kansas	4,856	19.8	1.1	(17.7–21.9)
Kingsport-Bristol-Bristol, Tennessee-Virginia	506	27.5	3.3	(21.1–34.0)
Knoxville, Tennessee	561	28.1	3.3	(21.7–34.5)
Lafayette, Louisiana	562	13.9	1.7	(10.6–17.2)
Lexington-Fayette, Kentucky	620	22.5	2.2	(18.2–26.8)
Lincoln, Nebraska	2,005	19.2	1.2	(16.8–21.5)
Little Rock-North Little Rock-Conway, Arkansas	1,174	23.9	1.9	(20.2–27.7)
Logan, Utah-Idaho	622	19.3	1.8	(15.8–22.7)
Los Angeles-Long Beach-Anaheim, California	2,441	11.7	0.8	(10.2–13.2)
Louisville-Jefferson County, Kentucky-Indiana	2,453	22.6	1.7	(19.2–26.0)
Madison, Wisconsin	549	16.7	2.0	(12.7–20.7)
Memphis, Tennessee-Mississippi-Arkansas	878	13.8	1.8	(10.3–17.2)
Miami-Fort Lauderdale-West Palm Beach, Florida	2,213	12.6	1.0	(10.7–14.5)
Milwaukee-Waukesha-West Allis, Wisconsin	1,351	18.6	1.8	(15.1–22.0)
Minneapolis-St. Paul-Bloomington, Minnesota-Wisconsin	8,729	18.0	0.5	(17.0–19.0)
Minot, North Dakota	595	17.9	2.4	(13.2–22.7)
Montgomery, Alabama	513	16.5	2.0	(12.6–20.3)
Montgomery County-Bucks County-Chester County, Pennsylvania^§^	800	16.6	2.0	(12.7–20.6)
Myrtle Beach-Conway-North Myrtle Beach, South Carolina-North Carolina	995	20.5	1.9	(16.9–24.2)
Nashville-Davidson County-Murfreesboro-Franklin, Tennessee	800	18.8	1.9	(15.2–22.5)
Nassau County-Suffolk County, New York^§^	770	11.2	1.6	(8.1–14.3)
Newark, New Jersey-Pennsylvania^§^	4,124	12.0	0.8	(10.5–13.6)
New Orleans-Metairie, Louisiana	1,908	19.2	1.2	(16.9–21.5)
New York-Jersey City-White Plains, New York-New Jersey^§^	7,503	14.3	0.6	(13.1–15.4)
Norfolk, Nebraska	996	16.7	1.6	(13.5–19.9)
North Platte, Nebraska	962	19.8	1.7	(16.4–23.2)
North Port-Sarasota-Bradenton, Florida	511	22.4	3.9	(14.9–30.0)
Oakland-Hayward-Berkeley, California^§^	694	14.5	1.6	(11.4–17.6)
Ogden-Clearfield, Utah	2,917	21.3	0.9	(19.6–23.0)
Oklahoma City, Oklahoma	2,429	20.0	1.0	(18.0–22.0)
Omaha-Council Bluffs, Nebraska-Iowa	4,858	18.4	0.8	(16.8–20.1)
Orlando-Kissimmee-Sanford, Florida	950	16.5	1.7	(13.2–19.7)
Philadelphia, Pennsylvania^§^	1,509	18.4	1.4	(15.6–21.2)
Phoenix-Mesa-Scottsdale, Arizona	9,357	17.5	0.6	(16.3–18.8)
Pittsburgh, Pennsylvania	2,413	19.8	1.2	(17.5–22.1)
Ponce, Puerto Rico	530	16.4	2.1	(12.2–20.5)
Portland-South Portland, Maine	2,744	21.8	1.2	(19.3–24.2)
Portland-Vancouver-Hillsboro, Oregon-Washington	2,832	22.9	1.1	(20.7–25.0)
Providence-Warwick, Rhode Island-Massachusetts	8,063	21.8	0.9	(20.0–23.6)
Provo-Orem, Utah	2,142	18.4	0.9	(16.5–20.2)
Raleigh, North Carolina	719	17.5	1.8	(13.9–21.0)
Rapid City, South Dakota	1,414	17.3	1.6	(14.1–20.5)
Reno, Nevada	1,192	16.7	1.5	(13.8–19.7)
Richmond, Virginia	1,457	15.5	1.2	(13.1–17.8)
Riverside-San Bernardino-Ontario, California	937	10.5	1.1	(8.4–12.6)
Roanoke, Virginia	533	19.7	2.6	(14.5–24.8)
Rochester, Minnesota	696	14.9	1.6	(11.7–18.1)
Rockingham County-Strafford County, New Hampshire^§^	1,439	21.8	1.7	(18.4–25.1)
Sacramento-Roseville-Arden-Arcade, California	640	13.9	1.7	(10.7–17.2)
St. Cloud, Minnesota	559	17.5	1.9	(13.8–21.2)
St. Louis, Missouri-Illinois	1,916	20.0	1.4	(17.2–22.8)
Salisbury, Maryland-Delaware	1,953	20.6	2.0	(16.7–24.6)
Salt Lake City, Utah	5,400	21.8	0.6	(20.6–23.1)
San Antonio-New Braunfels, Texas	2,268	14.7	1.0	(12.8–16.6)
San Juan-Carolina-Caguas, Puerto Rico	3,743	18.0	0.8	(16.5–19.5)
Scottsbluff, Nebraska	906	23.5	2.1	(19.4–27.6)
Seattle-Bellevue-Everett, Washington^¶^	3,684	19.8	0.9	(18.1–21.6)
Shreveport-Bossier City, Louisiana	550	17.4	2.0	(13.5–21.2)
Silver Spring-Frederick-Rockville, Maryland^¶^	2,371	15.2	1.4	(12.5–18.0)
Sioux City, Iowa-Nebraska-South Dakota	1,141	16.8	2.2	(12.4–21.2)
Sioux Falls, South Dakota	1,338	19.7	1.9	(16.0–23.5)
Spartanburg, South Carolina	562	25.8	3.0	(19.8–31.7)
Spokane-Spokane Valley, Washington	723	22.1	2.2	(17.7–26.4)
Springfield, Massachusetts	1,098	29.5	2.3	(25.1–34.0)
Tampa-St. Petersburg-Clearwater, Florida	1,576	18.1	1.5	(15.2–21.1)
Toledo, Ohio	646	25.1	2.8	(19.7–30.6)
Topeka, Kansas	1,439	19.0	1.3	(16.4–21.5)
Tulsa, Oklahoma	2,023	21.4	1.2	(19.0–23.7)
Tuscaloosa, Alabama	718	20.2	2.1	(16.2–24.3)
Virginia Beach-Norfolk-Newport News, Virginia-North Carolina	1,878	16.7	1.3	(14.2–19.2)
Warren-Troy-Farmington Hills, Michigan^§^	2,105	18.6	1.3	(16.1–21.2)
Washington-Arlington-Alexandria, District of Columbia-Virginia-Maryland-West Virginia^§^	8,261	13.4	0.7	(12.1–14.7)
Wichita, Kansas	2,720	20.9	1.0	(19.0–22.8)
Wichita Falls, Texas	542	16.7	2.8	(11.1–22.2)
Wilmington, Delaware-Maryland-New Jersey^§^	2,758	18.9	1.2	(16.5–21.2)
Worcester, Massachusetts-Connecticut	2,446	22.6	1.4	(19.9–25.2)
Youngstown-Warren-Boardman, Ohio-Pennsylvania	521	19.3	2.8	(13.8–24.8)
*Median*		*18.8*		
*Range*		*10.5–29.5*		

**Abbreviations:** CI = confidence interval; MMSA = metropolitan and micropolitan statistical areas; SE = standard error.

* Age adjusted to the 2000 U.S. standard population.

^†^ Including depression, major depression, dysthymia, or minor depression.

^§^ Metropolitan division.

### Cardiovascular Conditions

#### Coronary Heart Disease 

Respondents aged ≥45 years were categorized as having coronary heart disease if they reported having ever been told by a doctor, nurse, or other health professional they have had a heart attack (myocardial infarction) or angina. In 2013, age-adjusted prevalence estimates of coronary heart disease among adults aged ≥45 years ranged from 7.4% in Hawaii to 17.5% in West Virginia (median: 11.0%) (Table 53). Among selected MMSA, age-adjusted prevalence estimates ranged from 6.2% in San Francisco-Redwood City-South San Francisco, California, to 20.9% in Ponce, Puerto Rico (median: 11.1%) (Table 54). In 2014, age-adjusted prevalence estimates ranged from 8.0% in Hawaii to 17.1% in Puerto Rico (median: 11.0%) (Table 55). Among selected MMSA, the age-adjusted prevalence estimates ranged from 7.6% in Provo-Orem, Utah, to 19.2% in Ponce, Puerto Rico (median: 11.3%) (Table 56).

**TABLE 53:** Age-adjusted* prevalence estimates of adults aged ≥45 years who have ever been told by a health professional they have had a coronary heart disease,^†^ by state/territory — Behavioral Risk Factor Surveillance System, United States, 2013

State/Territory	Sample size	%	SE	95% CI
Alabama	4,900	14.0	0.6	(12.7–15.3)
Alaska	2,775	11.5	0.9	(9.7–13.3)
Arizona	3,094	10.8	0.9	(9.0–12.7)
Arkansas	3,956	13.3	0.7	(11.9–14.7)
California	7,123	8.8	0.5	(7.8–9.7)
Colorado	9,372	8.2	0.4	(7.5–8.9)
Connecticut	5,392	9.8	0.5	(8.8–10.9)
Delaware	3,736	11.4	0.6	(10.1–12.7)
District of Columbia	3,534	10.1	0.8	(8.7–11.6)
Florida	26,393	12.2	0.5	(11.3–13.1)
Georgia	5,529	10.9	0.5	(9.9–12.0)
Hawaii	5,100	7.4	0.5	(6.3–8.5)
Idaho	3,932	9.2	0.6	(8.0–10.4)
Illinois	3,962	10.5	0.6	(9.3–11.8)
Indiana	7,430	12.5	0.5	(11.6–13.4)
Iowa	5,996	11.2	0.5	(10.3–12.1)
Kansas	16,354	11.3	0.3	(10.7–11.8)
Kentucky	8,019	14.9	0.6	(13.7–16.2)
Louisiana	3,951	13.9	0.8	(12.4–15.4)
Maine	6,098	11.6	0.5	(10.5–12.6)
Maryland	9,525	9.8	0.4	(8.9–10.7)
Massachusetts	10,620	9.3	0.4	(8.4–10.1)
Michigan	9,082	12.9	0.5	(12.0–13.8)
Minnesota	9,900	8.5	0.6	(7.3–9.6)
Mississippi	5,525	13.3	0.7	(12.0–14.6)
Missouri	5,152	13.2	0.7	(11.9–14.6)
Montana	6,810	9.7	0.5	(8.7–10.7)
Nebraska	12,314	10.5	0.4	(9.7–11.3)
Nevada	3,487	10.8	0.9	(9.1–12.6)
New Hampshire	4,815	10.2	0.6	(9.0–11.4)
New Jersey	8,685	10.4	0.5	(9.4–11.4)
New Mexico	6,418	9.2	0.4	(8.4–10.1)
New York	5,773	12.3	0.6	(11.1–13.5)
North Carolina	6,141	11.7	0.5	(10.7–12.7)
North Dakota	5,397	10.9	0.5	(9.9–12.0)
Ohio	8,544	12.7	0.5	(11.7–13.8)
Oklahoma	5,933	13.3	0.5	(12.3–14.3)
Oregon	4,308	10.1	0.6	(8.9–11.2)
Pennsylvania	8,067	11.9	0.5	(11.0–12.8)
Rhode Island	4,713	11.0	0.6	(9.9–12.1)
South Carolina	7,740	12.3	0.5	(11.3–13.3)
South Dakota	4,708	12.4	0.8	(10.8–14.0)
Tennessee	4,249	16.9	0.8	(15.2–18.5)
Texas	7,175	11.0	0.6	(9.9–12.2)
Utah	7,488	9.6	0.4	(8.8–10.4)
Vermont	4,719	9.9	0.5	(8.9–10.9)
Virginia	5,766	10.6	0.5	(9.6–11.5)
Washington	7,958	10.5	0.5	(9.6–11.4)
West Virginia	4,134	17.5	0.7	(16.1–18.8)
Wisconsin	4,686	11.1	0.8	(9.6–12.6)
Wyoming	4,987	10.6	0.5	(9.6–11.7)
Guam	806	14.3	1.8	(10.7–17.8)
Puerto Rico	4,033	16.1	0.7	(14.7–17.6)
*Median*		*11.0*		
*Range*		*7.4–17.5*		

**Abbreviations:** CI = confidence interval; SE = standard error.

* Age adjusted to the 2000 U.S. standard population.

^† ^Including heart attack (also known as myocardial infarction) or angina.

**TABLE 54:** Age-adjusted* prevalence estimates of adults aged ≥45 years who have ever been told by a health professional they have had a coronary heart disease,^†^ by metropolitan and micropolitan statistical area — Behavioral Risk Factor Surveillance System, United States, 2013

MMSA	Sample size	%	SE	95% CI
Aguadilla-Isabela, Puerto Rico	408	15.6	2.2	(11.2–20.0)
Akron, Ohio	535	13.8	2.4	(9.1–18.5)
Albuquerque, New Mexico	1,333	7.6	0.8	(6.0–9.1)
Allentown-Bethlehem-Easton, Pennsylvania-New Jersey	716	14.4	1.8	(10.8–18.0)
Anchorage, Alaska	921	11.7	1.4	(8.9–14.4)
Atlanta-Sandy Springs-Roswell, Georgia	2,243	10.3	0.9	(8.6–12.0)
Augusta-Richmond County, Georgia-South Carolina	669	12.7	2.0	(8.8–16.7)
Austin-Round Rock, Texas	587	8.8	1.6	(5.7–12.0)
Baltimore-Columbia-Towson, Maryland	3,411	11.1	0.7	(9.7–12.6)
Baton Rouge, Louisiana	670	12.8	1.5	(9.9–15.7)
Billings, Montana	492	10.6	1.5	(7.7–13.5)
Birmingham-Hoover, Alabama	974	12.8	1.3	(10.3–15.2)
Bismarck, North Dakota	690	10.3	1.3	(7.7–12.9)
Boise City, Idaho	984	8.8	1.1	(6.7–11.0)
Boston, Massachusetts^§^	2,780	9.6	0.9	(7.9–11.3)
Buffalo-Cheektowaga-Niagara Falls, New York	362	12.0	2.1	(7.9–16.1)
Burlington-South Burlington, Vermont	1,074	9.4	1.1	(7.3–11.4)
Cambridge-Newton-Framingham, Massachusetts^§^	3,333	8.3	0.7	(7.0–9.6)
Camden, New Jersey^§^	1,246	11.3	1.2	(8.9–13.6)
Cedar Rapids, Iowa	463	10.9	1.6	(7.8–13.9)
Charleston, West Virginia	581	16.1	1.6	(12.8–19.3)
Charleston-North Charleston, South Carolina	1,066	11.2	1.5	(8.3–14.2)
Charlotte-Concord-Gastonia, North Carolina-South Carolina	1,284	13.5	1.3	(11.0–16.1)
Chattanooga, Tennessee-Georgia	458	14.4	2.1	(10.2–18.6)
Chicago-Naperville-Elgin, Illinois-Indiana-Wisconsin	2,262	9.9	0.9	(8.2–11.5)
Cincinnati, Ohio-Kentucky-Indiana	1,837	12.7	1.1	(10.6–14.9)
Claremont-Lebanon, New Hampshire-Vermont	1,260	10.6	2.0	(6.7–14.6)
Cleveland-Elyria, Ohio	793	10.7	1.5	(7.8–13.6)
Colorado Springs, Colorado	933	6.7	1.1	(4.6–8.8)
Columbia, South Carolina	972	10.2	1.2	(7.8–12.7)
Columbus, Ohio	1,174	12.4	1.2	(10.1–14.7)
Crestview-Fort Walton Beach-Destin, Florida	778	12.2	1.5	(9.2–15.1)
Dallas-Plano-Irving, Texas^§^	521	11.9	1.8	(8.5–15.3)
Davenport-Moline-Rock Island, Iowa-Illinois	502	14.1	1.9	(10.4–17.9)
Dayton, Ohio	627	12.2	1.6	(9.2–15.3)
Deltona-Daytona Beach-Ormond Beach, Florida	915	10.6	1.3	(8.0–13.2)
Denver-Aurora-Lakewood, Colorado	3,655	7.4	0.5	(6.4–8.4)
Des Moines-West Des Moines, Iowa	967	11.3	1.1	(9.1–13.5)
Duluth, Minnesota-Wisconsin	516	13.2	2.8	(7.7–18.7)
Durham-Chapel Hill, North Carolina	430	7.8	1.4	(5.1–10.5)
El Paso, Texas	481	7.7	1.7	(4.4–11.0)
Evansville, Indiana-Kentucky	436	11.6	2.1	(7.5–15.8)
Fargo, North Dakota-Minnesota	733	11.2	1.8	(7.6–14.7)
Fayetteville-Springdale-Rogers, Arkansas-Missouri	574	10.5	1.4	(7.8–13.2)
Fort Smith, Arkansas-Oklahoma	380	15.2	2.2	(10.9–19.4)
Fort Wayne, Indiana	559	11.7	1.5	(8.7–14.7)
Fort Worth-Arlington, Texas^§^	569	11.7	2.0	(7.8–15.6)
Gainesville, Florida	763	9.1	1.4	(6.5–11.8)
Grand Forks, North Dakota-Minnesota	343	9.7	1.8	(6.2–13.2)
Grand Island, Nebraska	605	8.5	1.2	(6.1–10.8)
Grand Rapids-Wyoming, Michigan	896	12.5	1.4	(9.7–15.2)
Greensboro-High Point, North Carolina	455	10.9	1.7	(7.6–14.3)
Greenville-Anderson-Mauldin, South Carolina	927	13.2	1.3	(10.6–15.9)
Gulfport-Biloxi-Pascagoula, Mississippi	553	14.1	1.6	(10.9–17.2)
Hagerstown-Martinsburg, Maryland-West Virginia	578	14.2	2.0	(10.2–18.1)
Hartford-West Hartford-East Hartford, Connecticut	2,002	10.2	0.9	(8.4–12.1)
Hilton Head Island-Bluffton-Beaufort, South Carolina	676	10.1	1.4	(7.4–12.8)
Houston-The Woodlands-Sugar Land, Texas	767	11.5	1.8	(8.1–15.0)
Huntington-Ashland, West Virginia-Kentucky-Ohio	820	20.7	1.8	(17.1–24.2)
Idaho Falls, Idaho	331	8.7	1.9	(5.0–12.5)
Indianapolis-Carmel-Anderson, Indiana	1,751	12.5	0.9	(10.7–14.3)
Jackson, Mississippi	539	11.3	1.7	(7.9–14.7)
Jacksonville, Florida	2,049	13.0	1.2	(10.7–15.3)
Kansas City, Missouri-Kansas	5,183	11.0	0.9	(9.2–12.7)
Kingsport-Bristol-Bristol, Tennessee-Virginia	440	17.3	3.1	(11.2–23.4)
Knoxville, Tennessee	465	15.6	2.1	(11.5–19.7)
Lansing-East Lansing, Michigan	464	7.8	1.5	(4.8–10.7)
Lexington-Fayette, Kentucky	379	13.9	2.6	(8.8–19.1)
Lincoln, Nebraska	1,109	11.4	1.1	(9.3–13.5)
Little Rock-North Little Rock-Conway, Arkansas	823	14.7	1.7	(11.4–18.0)
Logan, Utah-Idaho	362	8.0	1.5	(5.0–11.0)
Los Angeles-Long Beach-Anaheim, California	1,775	8.0	0.9	(6.2–9.9)
Louisville-Jefferson County, Kentucky-Indiana	1,569	13.2	1.5	(10.3–16.0)
Lubbock, Texas	408	8.8	1.6	(5.7–11.8)
Manhattan, Kansas	388	8.5	1.6	(5.4–11.6)
Memphis, Tennessee-Mississippi-Arkansas	843	16.9	2.3	(12.5–21.4)
Miami-Fort Lauderdale-West Palm Beach, Florida	1,589	10.8	1.3	(8.3–13.4)
Milwaukee-Waukesha-West Allis, Wisconsin	899	11.6	1.8	(8.0–15.2)
Minneapolis-St. Paul-Bloomington, Minnesota-Wisconsin	6,234	9.3	0.8	(7.7–10.9)
Minot, North Dakota	413	7.8	1.4	(5.0–10.7)
Montgomery County-Bucks County-Chester County, Pennsylvania^§^	639	9.0	1.1	(6.8–11.3)
Myrtle Beach-Conway-North Myrtle Beach, South Carolina-North Carolina	576	17.1	2.4	(12.4–21.8)
Nashville-Davidson County-Murfreesboro-Franklin, Tennessee	698	16.0	1.8	(12.4–19.6)
Nassau County-Suffolk County, New York^§^	610	12.1	1.6	(9.1–15.2)
Newark, New Jersey-Pennsylvania^§^	2,674	9.2	0.9	(7.5–10.9)
New Orleans-Metairie, Louisiana	959	12.3	1.6	(9.1–15.5)
New York-Jersey City-White Plains, New York-New Jersey^§^	5,420	11.5	0.7	(10.1–13.0)
Norfolk, Nebraska	446	17.1	2.0	(13.2–21.0)
North Platte, Nebraska	531	12.4	1.7	(9.1–15.6)
North Port-Sarasota-Bradenton, Florida	861	13.1	1.3	(10.4–15.7)
Oakland-Hayward-Berkeley, California^§^	409	6.9	1.5	(4.1–9.8)
Ogden-Clearfield, Utah	1,422	8.1	0.8	(6.5–9.7)
Oklahoma City, Oklahoma	1,809	12.6	0.9	(10.8–14.3)
Omaha-Council Bluffs, Nebraska-Iowa	2,050	10.4	0.9	(8.7–12.2)
Orlando-Kissimmee-Sanford, Florida	1,597	11.0	1.1	(8.9–13.2)
Panama City, Florida	805	16.0	1.9	(12.2–19.8)
Pensacola-Ferry Pass-Brent, Florida	889	12.4	1.3	(9.9–14.9)
Philadelphia, Pennsylvania^§^	1,181	12.0	1.2	(9.6–14.5)
Phoenix-Mesa-Scottsdale, Arizona	1,073	10.2	1.4	(7.6–12.9)
Pittsburgh, Pennsylvania	1,717	11.1	0.9	(9.3–12.8)
Ponce, Puerto Rico	342	20.9	2.8	(15.4–26.4)
Portland-South Portland, Maine	1,967	10.1	0.8	(8.4–11.8)
Portland-Vancouver-Hillsboro, Oregon-Washington	2,293	8.8	0.8	(7.2–10.4)
Port St. Lucie, Florida	831	11.1	1.3	(8.4–13.7)
Providence-Warwick, Rhode Island-Massachusetts	6,046	10.7	0.6	(9.5–12.0)
Provo-Orem, Utah	875	8.7	1.1	(6.6–10.8)
Raleigh, North Carolina	372	9.6	1.8	(6.1–13.1)
Rapid City, South Dakota	611	12.6	1.7	(9.3–15.8)
Reno, Nevada	1,219	10.8	1.1	(8.5–13.0)
Richmond, Virginia	885	10.8	1.3	(8.3–13.2)
Riverside-San Bernardino-Ontario, California	848	12.9	1.5	(9.9–15.9)
Rochester, New York	342	15.7	2.9	(10.0–21.4)
Rockingham County-Strafford County, New Hampshire^§^	1,188	10.6	1.1	(8.5–12.7)
Sacramento-Roseville-Arden-Arcade, California	586	8.0	1.3	(5.4–10.6)
St. Louis, Missouri-Illinois	1,467	13.2	1.3	(10.6–15.7)
Salem, Oregon	392	10.4	2.3	(5.9–14.9)
Salina, Kansas	377	10.8	1.7	(7.5–14.0)
Salisbury, Maryland-Delaware	1,638	13.8	1.2	(11.5–16.0)
Salt Lake City, Utah	2,716	10.2	0.7	(8.7–11.6)
San Antonio-New Braunfels, Texas	621	9.1	1.3	(6.6–11.7)
San Francisco-Redwood City-South San Francisco, California^§^	321	6.2	1.7	(2.9–9.5)
San Jose-Sunnyvale-Santa Clara, California	351	7.3	1.5	(4.3–10.3)
San Juan-Carolina-Caguas, Puerto Rico	2,422	15.1	0.9	(13.2–16.9)
Scottsbluff, Nebraska	557	13.3	1.7	(10.0–16.6)
Scranton-Wilkes-Barre-Hazleton, Pennsylvania	432	10.9	1.7	(7.6–14.2)
Seattle-Bellevue-Everett, Washington^§^	2,487	9.7	0.8	(8.1–11.2)
Shreveport-Bossier City, Louisiana	445	16.0	2.4	(11.3–20.6)
Silver Spring-Frederick-Rockville, Maryland^§^	1,706	7.1	0.9	(5.4–8.8)
Sioux City, Iowa-Nebraska-South Dakota	762	10.4	1.8	(6.8–13.9)
Sioux Falls, South Dakota	608	9.7	1.5	(6.7–12.6)
Spartanburg, South Carolina	462	12.5	2.1	(8.3–16.6)
Spokane-Spokane Valley, Washington	634	9.6	1.4	(6.8–12.4)
Springfield, Massachusetts	1,161	10.1	1.5	(7.3–13.0)
Tallahassee, Florida	1,424	9.6	1.3	(7.1–12.2)
Tampa-St. Petersburg-Clearwater, Florida	1,603	13.4	1.2	(11.0–15.7)
Toledo, Ohio	726	15.1	1.9	(11.4–18.8)
Topeka, Kansas	1,754	11.4	0.8	(9.8–13.1)
Tulsa, Oklahoma	1,462	12.2	1.0	(10.3–14.2)
Virginia Beach-Norfolk-Newport News, Virginia-North Carolina	1,137	10.1	1.1	(8.1–12.2)
Warren-Troy-Farmington Hills, Michigan^§^	1,612	12.0	1.0	(10.0–14.0)
Washington-Arlington-Alexandria, District of Columbia-Virginia-Maryland-West Virginia^§^	6,186	8.0	0.7	(6.7–9.4)
Wichita, Kansas	3,425	11.9	0.6	(10.6–13.1)
Wilmington, Delaware-Maryland-New Jersey^§^	2,268	10.8	0.8	(9.3–12.4)
Winston-Salem, North Carolina	523	9.0	1.3	(6.5–11.5)
Worcester, Massachusetts-Connecticut	1,925	9.4	0.9	(7.6–11.2)
*Median*		*11.1*		
*Range*		*6.2–20.9*		

**Abbreviations:** CI = confidence interval; MMSA = metropolitan and micropolitan statistical areas; SE = standard error.

* Age adjusted to the 2000 U.S. standard population.

^†^ Including heart attack (also known as myocardial infarction) or angina.

^§^ Metropolitan division.

**TABLE 55:** Age-adjusted* prevalence estimates of adults aged ≥45 years who have ever been told by a health professional they have had a coronary heart disease,^†^ by state/territory — Behavioral Risk Factor Surveillance System, United States, 2014

State/Territory	Sample size	%	SE	95% CI
Alabama	6,387	14.6	0.6	(13.4–15.8)
Alaska	2,854	8.9	0.8	(7.4–10.5)
Arizona	11,619	10.5	0.4	(9.7–11.2)
Arkansas	4,148	15.9	0.8	(14.4–17.5)
California	5,291	10.2	0.5	(9.1–11.2)
Colorado	9,435	8.6	0.4	(7.9–9.3)
Connecticut	5,831	9.4	0.5	(8.4–10.3)
Delaware	3,231	11.9	0.7	(10.4–13.3)
District of Columbia	3,024	8.3	0.7	(6.9–9.6)
Florida	7,520	12.1	0.5	(11.2–13.1)
Georgia	4,615	12.2	0.6	(11.0–13.5)
Hawaii	4,795	8.0	0.6	(6.9–9.2)
Idaho	3,906	9.2	0.5	(8.2–10.3)
Illinois	3,549	10.2	0.6	(9.0–11.4)
Indiana	8,550	12.5	0.4	(11.6–13.3)
Iowa	5,999	10.7	0.5	(9.7–11.6)
Kansas	9,549	10.7	0.4	(10.0–11.4)
Kentucky	8,527	15.6	0.6	(14.4–16.9)
Louisiana	4,719	15.5	0.6	(14.3–16.7)
Maine	7,063	11.8	0.5	(10.7–12.8)
Maryland	9,723	8.9	0.4	(8.0–9.7)
Massachusetts	11,616	10.9	0.5	(10.0–11.8)
Michigan	6,182	13.2	0.5	(12.1–14.2)
Minnesota	10,977	9.6	0.3	(8.9–10.2)
Mississippi	3,117	13.0	0.7	(11.5–14.4)
Missouri	5,359	12.9	0.7	(11.5–14.2)
Montana	5,653	10.0	0.5	(8.9–11.0)
Nebraska	15,937	10.0	0.3	(9.4–10.6)
Nevada	2,625	12.2	1.0	(10.3–14.2)
New Hampshire	4,688	10.1	0.6	(9.0–11.2)
New Jersey	9,223	11.2	0.5	(10.2–12.1)
New Mexico	6,462	9.8	0.5	(8.9–10.8)
New York	4,658	10.1	0.5	(9.1–11.2)
North Carolina	4,888	13.6	0.6	(12.5–14.8)
North Dakota	5,791	11.3	0.6	(10.2–12.4)
Ohio	8,314	13.2	0.6	(12.1–14.3)
Oklahoma	6,070	14.4	0.5	(13.3–15.4)
Oregon	3,856	9.9	0.6	(8.8–11.0)
Pennsylvania	8,164	11.7	0.4	(10.8–12.5)
Rhode Island	4,906	10.3	0.5	(9.3–11.4)
South Carolina	8,018	12.2	0.5	(11.2–13.1)
South Dakota	5,178	12.1	0.7	(10.6–13.5)
Tennessee	3,943	14.9	0.7	(13.4–16.3)
Texas	10,559	11.2	0.5	(10.1–12.2)
Utah	8,507	9.1	0.4	(8.3–9.8)
Vermont	4,573	9.8	0.5	(8.8–10.8)
Virginia	6,723	11.2	0.5	(10.2–12.1)
Washington	7,373	10.0	0.4	(9.2–10.9)
West Virginia	4,497	16.1	0.6	(14.8–17.3)
Wisconsin	5,057	9.5	0.6	(8.3–10.6)
Wyoming	5,138	11.0	0.6	(9.9–12.1)
Guam	1,193	10.7	1.5	(7.8–13.6)
Puerto Rico	4,075	17.1	0.8	(15.6–18.6)
*Median*		*11.0*		
*Range*		*8.0–17.1*		

**Abbreviations:** CI = confidence interval; SE = standard error.

* Age adjusted to the 2000 U.S. standard population.

^†^ Including heart attack (also known as myocardial infarction) or angina.

**TABLE 56:** Age-adjusted* prevalence estimates of adults aged ≥45 years who have ever been told by a health professional they have had a coronary heart disease,^†^ by metropolitan and micropolitan statistical area — Behavioral Risk Factor Surveillance System, United States, 2014

MMSA	Sample size	%	SE	95% CI
Aberdeen, South Dakota	457	14.2	1.9	(10.5–17.8)
Aguadilla-Isabela, Puerto Rico	360	15.9	2.6	(10.8–21.1)
Albuquerque, New Mexico	1,252	9.0	0.9	(7.1–10.8)
Allentown-Bethlehem-Easton, Pennsylvania-New Jersey	876	11.6	1.6	(8.4–14.8)
Anchorage, Alaska	1,126	8.8	1.2	(6.5–11.2)
Atlanta-Sandy Springs-Roswell, Georgia	1,893	10.4	1.0	(8.5–12.3)
Augusta-Richmond County, Georgia-South Carolina	630	8.7	1.3	(6.1–11.3)
Austin-Round Rock, Texas	1,515	9.4	0.9	(7.5–11.2)
Baltimore-Columbia-Towson, Maryland	3,483	8.0	0.6	(6.9–9.2)
Baton Rouge, Louisiana	604	14.8	1.6	(11.7–18.0)
Berlin, New Hampshire-Vermont	443	15.8	2.3	(11.2–20.3)
Billings, Montana	586	9.3	1.7	(6.0–12.6)
Birmingham-Hoover, Alabama	1,102	12.9	1.3	(10.4–15.4)
Bismarck, North Dakota	803	9.4	1.1	(7.2–11.6)
Boise City, Idaho	956	9.5	1.1	(7.3–11.6)
Boston, Massachusetts^§^	3,350	11.0	0.8	(9.4–12.6)
Burlington-South Burlington, Vermont	1,298	8.2	0.9	(6.4–10.1)
Cambridge-Newton-Framingham, Massachusetts^§^	3,821	9.7	0.7	(8.4–11.0)
Camden, New Jersey^§^	1,285	11.4	1.1	(9.2–13.7)
Cedar Rapids, Iowa	463	11.6	1.9	(7.8–15.4)
Charleston, West Virginia	621	16.8	1.7	(13.4–20.2)
Charleston-North Charleston, South Carolina	983	10.1	1.1	(7.8–12.3)
Charlotte-Concord-Gastonia, North Carolina-South Carolina	1,449	12.7	1.1	(10.5–15.0)
Chicago-Naperville-Elgin, Illinois-Indiana-Wisconsin	2,836	8.6	0.7	(7.3–9.9)
Cincinnati, Ohio-Kentucky-Indiana	1,468	12.7	1.3	(10.1–15.3)
Claremont-Lebanon, New Hampshire-Vermont	1,282	9.7	1.0	(7.7–11.7)
Cleveland-Elyria, Ohio	723	11.2	1.3	(8.7–13.8)
College Station-Bryan, Texas	436	10.3	2.3	(5.8–14.8)
Colorado Springs, Colorado	889	8.9	1.1	(6.7–11.1)
Columbia, South Carolina	817	11.5	1.3	(9.0–14.0)
Columbus, Ohio	1,173	12.8	1.3	(10.2–15.4)
Corpus Christi, Texas	500	12.3	2.8	(6.8–17.7)
Dallas-Plano-Irving, Texas^§^	902	10.3	1.7	(7.1–13.6)
Dayton, Ohio	421	17.1	2.6	(12.0–22.2)
Denver-Aurora-Lakewood, Colorado	3,838	8.1	0.5	(7.1–9.1)
Des Moines-West Des Moines, Iowa	972	9.6	1.2	(7.2–11.9)
Duluth, Minnesota-Wisconsin	675	12.2	1.7	(9.0–15.5)
El Paso, Texas	515	9.6	1.5	(6.6–12.6)
Evansville, Indiana-Kentucky	543	15.2	2.3	(10.6–19.8)
Fargo, North Dakota-Minnesota	817	10.2	1.3	(7.6–12.7)
Fayetteville-Springdale-Rogers, Arkansas-Missouri	629	13.5	1.6	(10.3–16.6)
Fort Wayne, Indiana	658	11.0	1.4	(8.2–13.7)
Fort Worth-Arlington, Texas^§^	539	11.5	1.6	(8.4–14.6)
Grand Island, Nebraska	789	10.4	1.2	(8.0–12.7)
Grand Rapids-Wyoming, Michigan	628	11.4	1.7	(8.1–14.6)
Greensboro-High Point, North Carolina	357	10.0	1.7	(6.7–13.2)
Greenville-Anderson-Mauldin, South Carolina	1,034	13.9	1.7	(10.7–17.2)
Hagerstown-Martinsburg, Maryland-West Virginia	636	14.6	1.9	(10.8–18.4)
Hartford-West Hartford-East Hartford, Connecticut	1,925	10.0	0.9	(8.3–11.7)
Hilton Head Island-Bluffton-Beaufort, South Carolina	440	11.8	2.2	(7.5–16.0)
Houston-The Woodlands-Sugar Land, Texas	1,438	10.7	1.5	(7.8–13.7)
Huntington-Ashland, West Virginia-Kentucky-Ohio	941	19.0	1.6	(15.9–22.1)
Idaho Falls, Idaho	341	9.6	1.9	(5.8–13.3)
Indianapolis-Carmel-Anderson, Indiana	2,668	12.1	0.8	(10.6–13.6)
Jacksonville, Florida	477	13.6	2.2	(9.4–17.9)
Kansas City, Missouri-Kansas	3,503	10.3	0.8	(8.8–11.8)
Kingsport-Bristol-Bristol, Tennessee-Virginia	404	15.0	2.1	(11.0–19.1)
Knoxville, Tennessee	445	14.5	2.0	(10.5–18.5)
Lafayette, Louisiana	383	16.4	2.2	(12.1–20.7)
Lexington-Fayette, Kentucky	388	16.4	2.9	(10.7–22.2)
Lincoln, Nebraska	1,217	8.3	0.9	(6.5–10.0)
Little Rock-North Little Rock-Conway, Arkansas	891	15.7	1.8	(12.2–19.1)
Logan, Utah-Idaho	333	8.4	1.5	(5.4–11.4)
Los Angeles-Long Beach-Anaheim, California	1,338	10.0	0.9	(8.1–11.8)
Louisville-Jefferson County, Kentucky-Indiana	1,949	14.0	1.2	(11.6–16.3)
Madison, Wisconsin	350	7.7	1.7	(4.3–11.1)
Memphis, Tennessee-Mississippi-Arkansas	653	12.3	1.7	(9.0–15.6)
Miami-Fort Lauderdale-West Palm Beach, Florida	1,636	10.7	1.0	(8.7–12.6)
Milwaukee-Waukesha-West Allis, Wisconsin	968	8.4	1.1	(6.1–10.6)
Minneapolis-St. Paul-Bloomington, Minnesota-Wisconsin	5,682	9.1	0.5	(8.2–10.0)
Minot, North Dakota	428	12.0	1.8	(8.4–15.6)
Montgomery, Alabama	360	14.9	2.2	(10.5–19.3)
Montgomery County-Bucks County-Chester County, Pennsylvania^§^	585	9.5	1.4	(6.7–12.3)
Myrtle Beach-Conway-North Myrtle Beach, South Carolina-North Carolina	764	14.2	1.7	(10.9–17.5)
Nashville-Davidson County-Murfreesboro-Franklin, Tennessee	563	14.7	1.8	(11.1–18.3)
Nassau County-Suffolk County, New York^§^	551	9.9	1.4	(7.1–12.7)
Newark, New Jersey-Pennsylvania^§^	2,952	11.2	0.9	(9.4–12.9)
New Orleans-Metairie, Louisiana	1,345	15.0	1.2	(12.7–17.3)
New York-Jersey City-White Plains, New York-New Jersey^§^	4,776	9.7	0.6	(8.6–10.9)
Norfolk, Nebraska	696	11.1	1.4	(8.3–13.9)
North Platte, Nebraska	706	13.1	1.4	(10.3–15.8)
North Port-Sarasota-Bradenton, Florida	440	12.5	2.1	(8.3–16.7)
Oakland-Hayward-Berkeley, California^§^	434	9.1	1.7	(5.8–12.4)
Ogden-Clearfield, Utah	1,594	8.0	0.7	(6.6–9.4)
Oklahoma City, Oklahoma	1,640	13.9	0.9	(12.1–15.8)
Omaha-Council Bluffs, Nebraska-Iowa	3,285	9.5	0.7	(8.2–10.8)
Orlando-Kissimmee-Sanford, Florida	661	14.6	1.6	(11.4–17.8)
Philadelphia, Pennsylvania^§^	1,089	11.1	1.3	(8.5–13.7)
Phoenix-Mesa-Scottsdale, Arizona	7,202	10.5	0.5	(9.6–11.4)
Pittsburgh, Pennsylvania	1,810	12.8	0.9	(10.9–14.6)
Ponce, Puerto Rico	375	19.2	2.8	(13.7–24.6)
Portland-South Portland, Maine	2,177	9.9	0.9	(8.2–11.7)
Portland-Vancouver-Hillsboro, Oregon-Washington	2,042	8.8	0.7	(7.4–10.3)
Providence-Warwick, Rhode Island-Massachusetts	6,192	11.9	0.8	(10.4–13.4)
Provo-Orem, Utah	975	7.6	0.9	(5.8–9.3)
Raleigh, North Carolina	402	12.3	1.8	(8.8–15.8)
Rapid City, South Dakota	1,035	14.7	1.7	(11.4–17.9)
Reno, Nevada	850	9.4	1.1	(7.2–11.6)
Richmond, Virginia	1,014	12.0	1.3	(9.4–14.6)
Riverside-San Bernardino-Ontario, California	573	10.7	1.6	(7.5–13.8)
Roanoke, Virginia	405	13.1	2.2	(8.8–17.4)
Rochester, Minnesota	458	8.3	1.4	(5.6–11.0)
Rockingham County-Strafford County, New Hampshire^§^	1,081	11.7	1.1	(9.5–13.8)
Sacramento-Roseville-Arden-Arcade, California	411	13.6	2.8	(8.1–19.2)
St. Cloud, Minnesota	338	8.7	1.7	(5.3–12.0)
St. Louis, Missouri-Illinois	1,403	12.7	1.3	(10.1–15.3)
Salisbury, Maryland-Delaware	1,605	13.2	1.0	(11.3–15.1)
Salt Lake City, Utah	3,000	9.2	0.6	(8.0–10.4)
San Antonio-New Braunfels, Texas	1,619	10.9	1.0	(8.8–12.9)
San Juan-Carolina-Caguas, Puerto Rico	2,562	17.4	1.0	(15.5–19.3)
Scottsbluff, Nebraska	703	14.8	1.9	(11.0–18.5)
Seattle-Bellevue-Everett, Washington^§^	2,603	9.2	0.7	(7.9–10.6)
Shreveport-Bossier City, Louisiana	378	12.1	1.9	(8.3–15.9)
Silver Spring-Frederick-Rockville, Maryland^§^	1,779	8.2	1.1	(6.0–10.4)
Sioux City, Iowa-Nebraska-South Dakota	871	10.7	2.1	(6.6–14.9)
Sioux Falls, South Dakota	905	11.2	1.2	(8.9–13.5)
Spartanburg, South Carolina	432	11.5	1.8	(8.0–15.0)
Spokane-Spokane Valley, Washington	546	10.1	1.6	(6.9–13.2)
Springfield, Massachusetts	785	13.3	1.9	(9.7–17.0)
Tampa-St. Petersburg-Clearwater, Florida	1,219	11.7	1.2	(9.4–14.1)
Toledo, Ohio	498	17.4	2.3	(12.9–21.9)
Topeka, Kansas	1,034	8.9	0.9	(7.1–10.7)
Tulsa, Oklahoma	1,462	13.9	1.1	(11.8–16.0)
Tuscaloosa, Alabama	490	12.8	1.9	(9.0–16.6)
Virginia Beach-Norfolk-Newport News, Virginia-North Carolina	1,339	11.0	1.1	(8.9–13.1)
Warren-Troy-Farmington Hills, Michigan^§^	1,538	12.7	1.0	(10.7–14.7)
Washington-Arlington-Alexandria, District of Columbia-Virginia-Maryland-West Virginia^§^	5,922	8.0	0.6	(6.8–9.2)
Wichita, Kansas	1,872	11.2	0.8	(9.6–12.7)
Wichita Falls, Texas	474	12.4	2.4	(7.7–17.1)
Wilmington, Delaware-Maryland-New Jersey^§^	2,051	12.1	1.0	(10.1–14.1)
Worcester, Massachusetts-Connecticut	1,783	10.8	1.1	(8.7–12.9)
Youngstown-Warren-Boardman, Ohio-Pennsylvania	416	11.4	1.8	(7.9–14.9)
*Median*		*11.3*		
*Range*		*7.6–19.2*		

**Abbreviations:** CI = confidence interval; MMSA = metropolitan and micropolitan statistical areas; SE = standard error.

* Age adjusted to the 2000 U.S. standard population.

^†^ Including heart attack (also known as myocardial infarction) or angina.

^§^ Metropolitan division.

#### Stroke 

Stroke was defined as respondents aged ≥45 years having ever been told by a doctor, nurse, or other health professional they have had a stroke. In 2013, age-adjusted prevalence estimates for stroke among adults aged ≥45 years ranged from 3.1% in Puerto Rico to 7.5% in Mississippi (median: 4.7%) (Table 57). Among selected MMSA, age-adjusted prevalence estimates ranged from 2.3% in Grand Island, Nebraska, to 9.4% in Spartanburg, South Carolina (median: 4.9%) (Table 58). In 2014, age-adjusted prevalence estimates ranged from 3.3% in Colorado to 8.0% in Guam (median: 4.8%) (Table 59). Among selected MMSA, age-adjusted prevalence estimates ranged from 2.3% in Sacramento-Roseville-Arden-Arcade, California, to 9.0% in Berlin, New Hampshire-Vermont (median: 5.0%) (Table 60).

**TABLE 57:** Age-adjusted* prevalence estimates of adults aged ≥45 years who have ever been told by a health professional they have had a stroke, by state/territory — Behavioral Risk Factor Surveillance System, United States, 2013

State/Territory	Sample size	%	SE	95% CI
Alabama	4,962	7.4	0.5	(6.4–8.4)
Alaska	2,798	5.0	0.6	(3.8–6.2)
Arizona	3,118	4.5	0.5	(3.5–5.5)
Arkansas	4,020	6.6	0.5	(5.6–7.6)
California	7,156	4.0	0.3	(3.5–4.6)
Colorado	9,423	3.2	0.2	(2.8–3.6)
Connecticut	5,454	3.8	0.3	(3.2–4.5)
Delaware	3,762	5.7	0.5	(4.7–6.7)
District of Columbia	3,552	5.9	0.6	(4.7–7.0)
Florida	26,625	5.3	0.3	(4.8–5.9)
Georgia	5,570	5.0	0.4	(4.3–5.7)
Hawaii	5,137	4.1	0.4	(3.4–4.9)
Idaho	3,972	3.6	0.4	(2.9–4.4)
Illinois	3,976	4.6	0.5	(3.7–5.6)
Indiana	7,517	5.2	0.3	(4.6–5.8)
Iowa	6,040	4.5	0.3	(3.9–5.2)
Kansas	16,476	4.9	0.2	(4.5–5.3)
Kentucky	8,090	6.5	0.4	(5.7–7.4)
Louisiana	3,985	6.3	0.6	(5.0–7.5)
Maine	6,142	3.7	0.3	(3.1–4.3)
Maryland	9,567	5.1	0.3	(4.5–5.8)
Massachusetts	10,675	3.8	0.3	(3.3–4.4)
Michigan	9,156	5.5	0.3	(4.9–6.2)
Minnesota	9,932	3.5	0.4	(2.8–4.3)
Mississippi	5,612	7.5	0.5	(6.5–8.5)
Missouri	5,218	5.2	0.4	(4.4–6.0)
Montana	6,849	5.1	0.4	(4.3–5.8)
Nebraska	12,454	4.1	0.2	(3.6–4.5)
Nevada	3,504	5.1	0.6	(4.0–6.3)
New Hampshire	4,832	3.5	0.3	(2.9–4.1)
New Jersey	8,751	4.3	0.3	(3.6–4.9)
New Mexico	6,463	4.0	0.3	(3.4–4.6)
New York	5,819	4.0	0.4	(3.3–4.7)
North Carolina	6,206	5.9	0.4	(5.1–6.6)
North Dakota	5,449	4.2	0.3	(3.5–4.9)
Ohio	8,620	5.9	0.4	(5.1–6.6)
Oklahoma	5,969	6.0	0.4	(5.3–6.7)
Oregon	4,323	5.3	0.4	(4.5–6.2)
Pennsylvania	8,122	5.0	0.3	(4.4–5.6)
Rhode Island	4,753	4.1	0.4	(3.3–4.8)
South Carolina	7,838	6.1	0.4	(5.3–6.9)
South Dakota	4,742	4.5	0.5	(3.6–5.4)
Tennessee	4,266	6.3	0.5	(5.3–7.4)
Texas	7,241	4.5	0.4	(3.8–5.2)
Utah	7,557	4.2	0.3	(3.6–4.8)
Vermont	4,739	4.3	0.4	(3.6–5.1)
Virginia	5,813	4.7	0.4	(4.0–5.5)
Washington	8,025	4.5	0.3	(3.9–5.1)
West Virginia	4,170	6.2	0.4	(5.4–7.0)
Wisconsin	4,701	3.8	0.4	(2.9–4.6)
Wyoming	5,011	4.7	0.4	(4.0–5.4)
Guam	815	4.9	0.9	(3.1–6.6)
Puerto Rico	4,050	3.1	0.3	(2.5–3.7)
*Median*		*4.7*		
*Range*		*3.1–7.5*		

**Abbreviations**: CI = confidence interval; SE = standard error.

* Age adjusted to the 2000 U.S. standard population.

**TABLE 58:** Age-adjusted* prevalence estimates of adults aged ≥45 years who have ever been told by a health professional they have had a stroke, by metropolitan and micropolitan statistical area — Behavioral Risk Factor Surveillance System, United States, 2013

MMSA	Sample size	%	SE	95% CI
Aguadilla-Isabela, Puerto Rico	410	N/A^§^	N/A^§^	(N/A–N/A)^§^
Akron, Ohio	539	7.3	1.4	(4.6–9.9)
Albuquerque, New Mexico	1,337	3.1	0.5	(2.1–4.1)
Allentown-Bethlehem-Easton, Pennsylvania-New Jersey	721	5.1	1.1	(2.9–7.3)
Anchorage, Alaska	924	5.3	1.0	(3.5–7.2)
Atlanta-Sandy Springs-Roswell, Georgia	2,256	4.7	0.5	(3.7–5.8)
Augusta-Richmond County, Georgia-South Carolina	675	5.7	1.4	(3.0–8.4)
Austin-Round Rock, Texas	593	6.3	1.6	(3.1–9.5)
Baltimore-Columbia-Towson, Maryland	3,431	6.1	0.6	(5.0–7.3)
Baton Rouge, Louisiana	673	6.3	1.1	(4.2–8.4)
Billings, Montana	489	7.2	1.3	(4.7–9.8)
Birmingham-Hoover, Alabama	979	6.6	0.9	(4.8–8.5)
Bismarck, North Dakota	697	4.8	1.0	(2.9–6.8)
Boise City, Idaho	990	2.9	0.6	(1.7–4.1)
Boston, Massachusetts^†^	2,801	3.8	0.5	(2.7–4.8)
Buffalo-Cheektowaga-Niagara Falls, New York	365	5.1	1.4	(2.4–7.8)
Burlington-South Burlington, Vermont	1,078	4.6	0.9	(2.9–6.3)
Cambridge-Newton-Framingham, Massachusetts^†^	3,337	3.6	0.5	(2.6–4.5)
Camden, New Jersey^†^	1,251	6.7	1.1	(4.6–8.8)
Cedar Rapids, Iowa	467	5.2	1.3	(2.6–7.8)
Charleston, West Virginia	586	7.8	1.3	(5.3–10.3)
Charleston-North Charleston, South Carolina	1,080	8.5	1.4	(5.7–11.3)
Charlotte-Concord-Gastonia, North Carolina-South Carolina	1,297	5.0	0.7	(3.6–6.4)
Chattanooga, Tennessee-Georgia	462	3.2	0.8	(1.7–4.7)
Chicago-Naperville-Elgin, Illinois-Indiana-Wisconsin	2,286	4.7	0.7	(3.4–6.1)
Cincinnati, Ohio-Kentucky-Indiana	1,848	5.4	0.7	(4.0–6.7)
Claremont-Lebanon, New Hampshire-Vermont	1,267	2.5	0.5	(1.6–3.3)
Cleveland-Elyria, Ohio	800	4.5	0.9	(2.7–6.3)
Colorado Springs, Colorado	935	3.1	0.7	(1.6–4.5)
Columbia, South Carolina	987	4.7	0.9	(2.9–6.6)
Columbus, Ohio	1,186	5.2	0.8	(3.7–6.8)
Crestview-Fort Walton Beach-Destin, Florida	787	6.1	1.2	(3.8–8.5)
Dallas-Plano-Irving, Texas^†^	525	3.0	0.8	(1.4–4.6)
Davenport-Moline-Rock Island, Iowa-Illinois	506	N/A^§^	N/A^§^	(N/A–N/A)^§^
Dayton, Ohio	628	5.8	1.2	(3.4–8.3)
Deltona-Daytona Beach-Ormond Beach, Florida	926	4.1	1.0	(2.1–6.1)
Denver-Aurora-Lakewood, Colorado	3,670	3.0	0.3	(2.3–3.6)
Des Moines-West Des Moines, Iowa	975	4.4	0.9	(2.7–6.0)
Duluth, Minnesota-Wisconsin	517	N/A^§^	N/A^§^	(N/A–N/A)^§^
Durham-Chapel Hill, North Carolina	432	5.5	1.5	(2.6–8.5)
El Paso, Texas	482	N/A^§^	N/A^§^	(N/A–N/A)^§^
Evansville, Indiana-Kentucky	439	4.0	0.9	(2.1–5.8)
Fargo, North Dakota-Minnesota	737	4.1	1.0	(2.2–6.1)
Fayetteville-Springdale-Rogers, Arkansas-Missouri	581	6.1	1.1	(3.9–8.3)
Fort Smith, Arkansas-Oklahoma	386	8.4	2.0	(4.5–12.3)
Fort Wayne, Indiana	565	3.9	0.9	(2.2–5.6)
Fort Worth-Arlington, Texas^†^	571	8.1	1.9	(4.4–11.9)
Gainesville, Florida	769	4.9	1.1	(2.7–7.1)
Grand Forks, North Dakota-Minnesota	344	N/A^§^	N/A^§^	(N/A–N/A)^§^
Grand Island, Nebraska	611	2.3	0.7	(1.0–3.7)
Grand Rapids-Wyoming, Michigan	900	4.3	0.8	(2.8–5.8)
Greensboro-High Point, North Carolina	460	5.8	1.4	(3.0–8.6)
Greenville-Anderson-Mauldin, South Carolina	941	4.9	0.8	(3.2–6.5)
Gulfport-Biloxi-Pascagoula, Mississippi	561	5.8	1.0	(3.8–7.8)
Hagerstown-Martinsburg, Maryland-West Virginia	580	3.4	0.7	(1.9–4.9)
Hartford-West Hartford-East Hartford, Connecticut	2,025	3.9	0.6	(2.8–5.0)
Hilton Head Island-Bluffton-Beaufort, South Carolina	682	3.7	1.1	(1.6–5.9)
Houston-The Woodlands-Sugar Land, Texas	774	3.0	0.9	(1.2–4.7)
Huntington-Ashland, West Virginia-Kentucky-Ohio	826	7.5	1.1	(5.3–9.6)
Idaho Falls, Idaho	336	N/A^§^	N/A^§^	(N/A–N/A)^§^
Indianapolis-Carmel-Anderson, Indiana	1,764	4.6	0.6	(3.5–5.7)
Jackson, Mississippi	545	6.5	1.5	(3.6–9.3)
Jacksonville, Florida	2,066	6.6	1.0	(4.7–8.5)
Kansas City, Missouri-Kansas	5,205	3.5	0.4	(2.7–4.3)
Kingsport-Bristol-Bristol, Tennessee-Virginia	443	N/A^§^	N/A^§^	(N/A–N/A)^§^
Knoxville, Tennessee	466	6.4	1.4	(3.6–9.1)
Lansing-East Lansing, Michigan	469	5.7	1.4	(2.9–8.5)
Lexington-Fayette, Kentucky	380	6.1	1.4	(3.3–8.8)
Lincoln, Nebraska	1,114	3.9	0.6	(2.8–5.1)
Little Rock-North Little Rock-Conway, Arkansas	839	6.3	1.0	(4.4–8.3)
Logan, Utah-Idaho	362	N/A^§^	N/A^§^	(N/A–N/A)^§^
Los Angeles-Long Beach-Anaheim, California	1,784	3.5	0.5	(2.5–4.5)
Louisville-Jefferson County, Kentucky-Indiana	1,582	7.1	1.2	(4.9–9.4)
Lubbock, Texas	406	5.3	1.3	(2.7–7.8)
Manhattan, Kansas	388	4.8	1.3	(2.3–7.3)
Memphis, Tennessee-Mississippi-Arkansas	852	6.5	1.2	(4.1–8.9)
Miami-Fort Lauderdale-West Palm Beach, Florida	1,604	3.7	0.6	(2.5–4.8)
Milwaukee-Waukesha-West Allis, Wisconsin	903	4.4	1.0	(2.3–6.4)
Minneapolis-St. Paul-Bloomington, Minnesota-Wisconsin	6,248	3.6	0.5	(2.6–4.7)
Minot, North Dakota	417	3.4	0.9	(1.6–5.1)
Montgomery County-Bucks County-Chester County, Pennsylvania^†^	642	3.1	0.7	(1.7–4.5)
Myrtle Beach-Conway-North Myrtle Beach, South Carolina-North Carolina	584	5.1	1.2	(2.7–7.5)
Nashville-Davidson County-Murfreesboro-Franklin, Tennessee	702	5.8	1.0	(3.7–7.8)
Nassau County-Suffolk County, New York^†^	613	2.7	0.7	(1.3–4.2)
Newark, New Jersey-Pennsylvania^†^	2,699	3.2	0.5	(2.2–4.3)
New Orleans-Metairie, Louisiana	962	6.6	1.8	(3.0–10.2)
New York-Jersey City-White Plains, New York-New Jersey^†^	5,471	4.1	0.4	(3.2–5.0)
Norfolk, Nebraska	454	6.6	1.6	(3.6–9.7)
North Platte, Nebraska	538	5.6	1.1	(3.5–7.7)
North Port-Sarasota-Bradenton, Florida	867	4.5	0.7	(3.1–6.0)
Oakland-Hayward-Berkeley, California^†^	409	4.5	1.2	(2.2–6.9)
Ogden-Clearfield, Utah	1,430	3.9	0.6	(2.7–5.1)
Oklahoma City, Oklahoma	1,820	5.6	0.6	(4.4–6.9)
Omaha-Council Bluffs, Nebraska-Iowa	2,064	3.8	0.5	(2.8–4.8)
Orlando-Kissimmee-Sanford, Florida	1,613	5.5	0.8	(4.0–7.1)
Panama City, Florida	810	5.3	1.0	(3.4–7.3)
Pensacola-Ferry Pass-Brent, Florida	898	5.2	0.8	(3.6–6.7)
Philadelphia, Pennsylvania^†^	1,188	6.4	0.9	(4.7–8.1)
Phoenix-Mesa-Scottsdale, Arizona	1,078	4.1	0.7	(2.6–5.5)
Pittsburgh, Pennsylvania	1,726	4.5	0.6	(3.2–5.7)
Ponce, Puerto Rico	345	3.1	0.9	(1.3–4.8)
Portland-South Portland, Maine	1,977	3.0	0.5	(2.1–4.0)
Portland-Vancouver-Hillsboro, Oregon-Washington	2,301	4.4	0.5	(3.4–5.5)
Port St. Lucie, Florida	842	5.0	1.2	(2.5–7.4)
Providence-Warwick, Rhode Island-Massachusetts	6,092	4.1	0.4	(3.3–4.9)
Provo-Orem, Utah	878	4.4	0.8	(2.9–6.0)
Raleigh, North Carolina	372	6.8	1.8	(3.3–10.2)
Rapid City, South Dakota	612	3.5	0.8	(2.0–5.1)
Reno, Nevada	1,223	3.2	0.6	(2.1–4.4)
Richmond, Virginia	889	4.5	0.9	(2.8–6.2)
Riverside-San Bernardino-Ontario, California	851	5.8	1.1	(3.5–8.0)
Rochester, New York	343	7.4	2.0	(3.4–11.3)
Rockingham County-Strafford County, New Hampshire^†^	1,196	3.7	0.6	(2.5–5.0)
Sacramento-Roseville-Arden-Arcade, California	589	2.8	0.7	(1.5–4.2)
St. Louis, Missouri-Illinois	1,477	5.5	0.8	(4.0–7.1)
Salem, Oregon	396	5.7	1.3	(3.2–8.2)
Salina, Kansas	381	8.1	1.6	(4.9–11.2)
Salisbury, Maryland-Delaware	1,641	5.2	0.7	(3.7–6.6)
Salt Lake City, Utah	2,746	4.4	0.5	(3.5–5.4)
San Antonio-New Braunfels, Texas	628	4.3	0.9	(2.4–6.1)
San Francisco-Redwood City-South San Francisco, California^†^	323	N/A^§^	N/A^§^	(N/A–N/A)^§^
San Jose-Sunnyvale-Santa Clara, California	354	N/A^§^	N/A^§^	(N/A–N/A)^§^
San Juan-Carolina-Caguas, Puerto Rico	2,430	3.2	0.4	(2.4–4.0)
Scottsbluff, Nebraska	561	3.4	0.8	(1.8–5.0)
Scranton-Wilkes-Barre-Hazleton, Pennsylvania	435	4.4	1.1	(2.4–6.5)
Seattle-Bellevue-Everett, Washington^†^	2,505	4.0	0.5	(3.1–4.9)
Shreveport-Bossier City, Louisiana	449	6.5	1.8	(3.0–10.1)
Silver Spring-Frederick-Rockville, Maryland^†^	1,712	3.3	0.6	(2.1–4.4)
Sioux City, Iowa-Nebraska-South Dakota	768	6.1	1.5	(3.2–9.0)
Sioux Falls, South Dakota	608	5.3	1.2	(2.9–7.8)
Spartanburg, South Carolina	466	9.4	2.3	(5.0–13.8)
Spokane-Spokane Valley, Washington	643	4.4	0.9	(2.6–6.2)
Springfield, Massachusetts	1,170	3.9	0.9	(2.2–5.6)
Tallahassee, Florida	1,428	5.4	1.2	(3.1–7.7)
Tampa-St. Petersburg-Clearwater, Florida	1,614	6.4	0.9	(4.6–8.1)
Toledo, Ohio	735	4.5	0.9	(2.8–6.2)
Topeka, Kansas	1,769	4.6	0.6	(3.4–5.7)
Tulsa, Oklahoma	1,469	5.3	0.7	(4.0–6.6)
Virginia Beach-Norfolk-Newport News, Virginia-North Carolina	1,144	5.4	0.8	(3.8–6.9)
Warren-Troy-Farmington Hills, Michigan^†^	1,623	6.1	0.8	(4.6–7.6)
Washington-Arlington-Alexandria, District of Columbia-Virginia-Maryland-West Virginia^†^	6,218	4.9	0.6	(3.7–6.1)
Wichita, Kansas	3,455	5.7	0.5	(4.8–6.6)
Wilmington, Delaware-Maryland-New Jersey^†^	2,283	6.1	0.6	(4.9–7.4)
Winston-Salem, North Carolina	527	5.2	1.1	(3.0–7.4)
Worcester, Massachusetts-Connecticut	1,946	4.1	0.7	(2.8–5.4)
*Median*		*4.9*		
*Range*		*2.3–9.4*		

**Abbreviations:** CI = confidence interval; MMSA = metropolitan and micropolitan statistical areas; N/A = not available; SE = standard error.

* Age adjusted to the 2000 U.S. standard population.

^†^ Metropolitan division.

^§^ Estimate not available if the unweighted sample size for the denominator was <50 or if the relative standard error is >30%.

**TABLE 59:** Age-adjusted* prevalence estimates of adults aged ≥45 years who have ever been told by a health professional they have had a stroke, by state/territory — Behavioral Risk Factor Surveillance System, United States, 2014

State/Territory	Sample size	%	SE	95% CI
Alabama	6,467	7.3	0.4	(6.5–8.2)
Alaska	2,875	5.0	0.6	(3.9–6.2)
Arizona	11,694	4.8	0.3	(4.3–5.4)
Arkansas	4,191	7.6	0.6	(6.4–8.8)
California	5,319	4.5	0.4	(3.8–5.3)
Colorado	9,495	3.3	0.2	(2.8–3.7)
Connecticut	5,870	4.1	0.4	(3.3–4.8)
Delaware	3,256	5.3	0.6	(4.2–6.4)
District of Columbia	3,045	6.0	0.6	(4.8–7.1)
Florida	7,564	4.7	0.3	(4.1–5.4)
Georgia	4,637	6.2	0.5	(5.2–7.2)
Hawaii	4,818	4.8	0.4	(3.9–5.6)
Idaho	3,922	4.6	0.4	(3.7–5.4)
Illinois	3,556	5.0	0.5	(4.1–5.9)
Indiana	8,628	5.2	0.3	(4.6–5.8)
Iowa	6,046	4.0	0.3	(3.4–4.6)
Kansas	9,651	5.0	0.3	(4.5–5.5)
Kentucky	8,605	6.4	0.5	(5.5–7.3)
Louisiana	4,759	6.6	0.5	(5.7–7.5)
Maine	7,110	4.6	0.3	(4.0–5.3)
Maryland	9,784	5.3	0.5	(4.4–6.3)
Massachusetts	11,670	4.3	0.3	(3.7–4.9)
Michigan	6,219	5.0	0.4	(4.3–5.7)
Minnesota	11,020	3.7	0.2	(3.3–4.1)
Mississippi	3,157	6.1	0.5	(5.2–7.1)
Missouri	5,400	6.3	0.5	(5.4–7.2)
Montana	5,708	4.2	0.4	(3.4–4.9)
Nebraska	16,096	4.4	0.3	(3.9–4.9)
Nevada	2,642	5.5	0.6	(4.3–6.8)
New Hampshire	4,716	3.5	0.3	(2.8–4.1)
New Jersey	9,282	4.2	0.3	(3.7–4.8)
New Mexico	6,485	4.6	0.4	(3.8–5.4)
New York	4,692	4.1	0.4	(3.4–4.8)
North Carolina	4,936	6.4	0.4	(5.6–7.2)
North Dakota	5,827	4.2	0.4	(3.4–5.0)
Ohio	8,368	5.7	0.4	(4.9–6.5)
Oklahoma	6,112	5.7	0.4	(5.0–6.4)
Oregon	3,884	5.0	0.4	(4.1–5.8)
Pennsylvania	8,217	5.1	0.3	(4.5–5.7)
Rhode Island	4,934	3.8	0.4	(3.1–4.5)
South Carolina	8,115	5.6	0.3	(4.9–6.2)
South Dakota	5,224	4.5	0.5	(3.6–5.4)
Tennessee	3,965	7.0	0.5	(6.0–8.0)
Texas	10,641	5.1	0.4	(4.3–5.8)
Utah	8,557	4.5	0.3	(3.9–5.1)
Vermont	4,593	3.6	0.3	(3.0–4.3)
Virginia	6,763	4.7	0.3	(4.1–5.4)
Washington	7,418	4.5	0.3	(3.9–5.1)
West Virginia	4,525	6.5	0.4	(5.6–7.3)
Wisconsin	5,084	4.5	0.5	(3.6–5.5)
Wyoming	5,182	4.4	0.4	(3.7–5.1)
Guam	1,202	8.0	1.1	(5.8–10.2)
Puerto Rico	4,085	3.6	0.4	(2.9–4.3)
*Median*		*4.8*		
*Range*		*3.3–8.0*		

**Abbreviations:** CI = confidence interval; SE = standard error.

* Age adjusted to the 2000 U.S. standard population.

**TABLE 60:** Age-adjusted* prevalence estimates of adults aged ≥45 years who have ever been told by a health professional they have had a stroke, by metropolitan and micropolitan statistical area — Behavioral Risk Factor Surveillance System, United States, 2014

MMSA	Sample size	%	SE	95% CI
Aberdeen, South Dakota	461	6.1	1.7	(2.8–9.4)
Aguadilla-Isabela, Puerto Rico	360	N/A^§^	N/A^§^	(N/A–N/A)^§^
Albuquerque, New Mexico	1,254	4.7	0.8	(3.1–6.3)
Allentown-Bethlehem-Easton, Pennsylvania-New Jersey	882	4.5	1.1	(2.3–6.6)
Anchorage, Alaska	1,133	4.9	1.0	(3.0–6.8)
Atlanta-Sandy Springs-Roswell, Georgia	1,899	5.0	0.7	(3.6–6.3)
Augusta-Richmond County, Georgia-South Carolina	637	3.9	0.9	(2.2–5.7)
Austin-Round Rock, Texas	1,524	3.8	0.6	(2.6–5.0)
Baltimore-Columbia-Towson, Maryland	3,509	5.8	0.8	(4.3–7.3)
Baton Rouge, Louisiana	604	7.7	1.3	(5.2–10.2)
Berlin, New Hampshire-Vermont	446	9.0	2.0	(5.0–12.9)
Billings, Montana	593	3.6	1.1	(1.5–5.7)
Birmingham-Hoover, Alabama	1,116	6.2	1.0	(4.3–8.2)
Bismarck, North Dakota	808	4.1	1.0	(2.1–6.2)
Boise City, Idaho	963	4.4	0.8	(2.8–6.0)
Boston, Massachusetts^†^	3,363	3.7	0.5	(2.7–4.8)
Burlington-South Burlington, Vermont	1,296	2.6	0.6	(1.3–3.8)
Cambridge-Newton-Framingham, Massachusetts^†^	3,839	4.2	0.5	(3.2–5.2)
Camden, New Jersey^†^	1,293	4.9	0.7	(3.5–6.4)
Cedar Rapids, Iowa	471	4.9	1.3	(2.4–7.5)
Charleston, West Virginia	622	8.4	1.4	(5.6–11.2)
Charleston-North Charleston, South Carolina	995	3.8	0.6	(2.5–5.0)
Charlotte-Concord-Gastonia, North Carolina-South Carolina	1,464	7.0	1.0	(5.0–8.9)
Chicago-Naperville-Elgin, Illinois-Indiana-Wisconsin	2,852	5.3	0.6	(4.1–6.4)
Cincinnati, Ohio-Kentucky-Indiana	1,478	4.8	0.8	(3.3–6.4)
Claremont-Lebanon, New Hampshire-Vermont	1,287	3.9	0.6	(2.7–5.0)
Cleveland-Elyria, Ohio	725	6.1	1.1	(3.9–8.2)
College Station-Bryan, Texas	441	N/A^§^	N/A^§^	(N/A–N/A)^§^
Colorado Springs, Colorado	893	2.6	0.6	(1.4–3.7)
Columbia, South Carolina	823	6.4	1.4	(3.7–9.1)
Columbus, Ohio	1,179	5.5	0.9	(3.8–7.2)
Corpus Christi, Texas	506	6.0	1.5	(3.1–8.9)
Dallas-Plano-Irving, Texas^†^	913	5.4	1.1	(3.2–7.7)
Dayton, Ohio	424	8.6	2.3	(4.1–13.2)
Denver-Aurora-Lakewood, Colorado	3,856	3.1	0.3	(2.5–3.7)
Des Moines-West Des Moines, Iowa	981	3.2	0.6	(2.1–4.4)
Duluth, Minnesota-Wisconsin	683	5.8	1.3	(3.3–8.4)
El Paso, Texas	516	3.4	0.9	(1.6–5.2)
Evansville, Indiana-Kentucky	548	6.2	1.4	(3.5–9.0)
Fargo, North Dakota-Minnesota	827	2.4	0.6	(1.3–3.5)
Fayetteville-Springdale-Rogers, Arkansas-Missouri	636	7.7	1.4	(5.0–10.4)
Fort Wayne, Indiana	658	7.0	1.5	(4.2–9.9)
Fort Worth-Arlington, Texas^†^	540	4.6	1.0	(2.6–6.6)
Grand Island, Nebraska	792	6.3	1.4	(3.6–9.0)
Grand Rapids-Wyoming, Michigan	633	N/A^§^	N/A^§^	(N/A–N/A)^§^
Greensboro-High Point, North Carolina	363	2.8	0.8	(1.3–4.3)
Greenville-Anderson-Mauldin, South Carolina	1,044	4.6	0.7	(3.1–6.0)
Hagerstown-Martinsburg, Maryland-West Virginia	639	6.1	1.3	(3.5–8.6)
Hartford-West Hartford-East Hartford, Connecticut	1,940	4.9	0.7	(3.5–6.2)
Hilton Head Island-Bluffton-Beaufort, South Carolina	445	3.3	0.9	(1.6–5.0)
Houston-The Woodlands-Sugar Land, Texas	1,446	6.5	1.2	(4.2–8.8)
Huntington-Ashland, West Virginia-Kentucky-Ohio	945	8.3	1.1	(6.1–10.5)
Idaho Falls, Idaho	341	N/A^§^	N/A^§^	(N/A–N/A)^§^
Indianapolis-Carmel-Anderson, Indiana	2,692	5.6	0.6	(4.5–6.7)
Jacksonville, Florida	481	5.9	1.3	(3.5–8.4)
Kansas City, Missouri-Kansas	3,538	6.0	0.6	(4.7–7.2)
Kingsport-Bristol-Bristol, Tennessee-Virginia	404	4.2	1.0	(2.3–6.1)
Knoxville, Tennessee	449	6.6	1.5	(3.7–9.5)
Lafayette, Louisiana	390	4.5	1.3	(2.0–7.0)
Lexington-Fayette, Kentucky	392	N/A^§^	N/A^§^	(N/A–N/A)^§^
Lincoln, Nebraska	1,233	4.1	0.6	(2.9–5.3)
Little Rock-North Little Rock-Conway, Arkansas	896	7.3	1.5	(4.4–10.2)
Logan, Utah-Idaho	334	N/A^§^	N/A^§^	(N/A–N/A)^§^
Los Angeles-Long Beach-Anaheim, California	1,343	5.0	0.8	(3.5–6.5)
Louisville-Jefferson County, Kentucky-Indiana	1,971	4.8	0.7	(3.5–6.1)
Madison, Wisconsin	352	N/A^§^	N/A^§^	(N/A–N/A)^§^
Memphis, Tennessee-Mississippi-Arkansas	654	5.6	1.4	(2.9–8.3)
Miami-Fort Lauderdale-West Palm Beach, Florida	1,646	3.0	0.5	(2.0–4.1)
Milwaukee-Waukesha-West Allis, Wisconsin	975	5.4	1.2	(3.1–7.7)
Minneapolis-St. Paul-Bloomington, Minnesota-Wisconsin	5,706	3.5	0.3	(3.0–4.1)
Minot, North Dakota	430	6.0	1.6	(2.9–9.1)
Montgomery, Alabama	368	7.8	1.8	(4.3–11.3)
Montgomery County-Bucks County-Chester County, Pennsylvania^†^	582	3.5	0.8	(1.8–5.1)
Myrtle Beach-Conway-North Myrtle Beach, South Carolina-North Carolina	771	5.2	1.2	(2.8–7.6)
Nashville-Davidson County-Murfreesboro-Franklin, Tennessee	567	5.9	1.1	(3.8–8.0)
Nassau County-Suffolk County, New York^†^	555	3.6	0.9	(1.9–5.3)
Newark, New Jersey-Pennsylvania^†^	2,973	4.2	0.5	(3.2–5.2)
New Orleans-Metairie, Louisiana	1,353	6.1	1.0	(4.2–8.0)
New York-Jersey City-White Plains, New York-New Jersey^†^	4,814	3.6	0.4	(2.9–4.4)
Norfolk, Nebraska	701	3.4	0.8	(1.8–5.0)
North Platte, Nebraska	715	5.3	0.9	(3.5–7.1)
North Port-Sarasota-Bradenton, Florida	443	4.3	1.1	(2.2–6.4)
Oakland-Hayward-Berkeley, California^†^	435	N/A^§^	N/A^§^	(N/A–N/A)^§^
Ogden-Clearfield, Utah	1,602	4.5	0.6	(3.3–5.8)
Oklahoma City, Oklahoma	1,645	5.1	0.6	(3.9–6.2)
Omaha-Council Bluffs, Nebraska-Iowa	3,304	4.4	0.4	(3.5–5.2)
Orlando-Kissimmee-Sanford, Florida	665	5.7	1.1	(3.5–7.9)
Philadelphia, Pennsylvania^†^	1,094	6.3	1.1	(4.2–8.4)
Phoenix-Mesa-Scottsdale, Arizona	7,246	4.8	0.3	(4.1–5.4)
Pittsburgh, Pennsylvania	1,825	5.5	0.6	(4.2–6.7)
Ponce, Puerto Rico	377	N/A^§^	N/A^§^	(N/A–N/A)^§^
Portland-South Portland, Maine	2,188	4.3	0.6	(3.1–5.5)
Portland-Vancouver-Hillsboro, Oregon-Washington	2,052	5.2	0.6	(4.1–6.3)
Providence-Warwick, Rhode Island-Massachusetts	6,230	4.6	0.6	(3.4–5.7)
Provo-Orem, Utah	978	4.0	0.7	(2.7–5.3)
Raleigh, North Carolina	405	4.3	1.2	(2.0–6.6)
Rapid City, South Dakota	1,043	5.8	1.0	(3.9–7.7)
Reno, Nevada	855	5.3	0.9	(3.5–7.1)
Richmond, Virginia	1,020	4.6	0.8	(3.0–6.1)
Riverside-San Bernardino-Ontario, California	578	6.2	1.2	(3.8–8.6)
Roanoke, Virginia	405	5.5	1.5	(2.5–8.4)
Rochester, Minnesota	457	N/A^§^	N/A^§^	(N/A–N/A)^§^
Rockingham County-Strafford County, New Hampshire^†^	1,088	2.7	0.6	(1.5–3.9)
Sacramento-Roseville-Arden-Arcade, California	414	2.3	0.7	(0.9–3.6)
St. Cloud, Minnesota	339	N/A^§^	N/A^§^	(N/A–N/A)^§^
St. Louis, Missouri-Illinois	1,408	5.1	0.8	(3.5–6.8)
Salisbury, Maryland-Delaware	1,625	5.0	0.6	(3.7–6.2)
Salt Lake City, Utah	3,019	4.6	0.5	(3.6–5.5)
San Antonio-New Braunfels, Texas	1,625	4.1	0.6	(3.0–5.3)
San Juan-Carolina-Caguas, Puerto Rico	2,568	4.3	0.5	(3.3–5.3)
Scottsbluff, Nebraska	709	6.1	1.4	(3.3–8.9)
Seattle-Bellevue-Everett, Washington^†^	2,621	3.7	0.4	(2.9–4.5)
Shreveport-Bossier City, Louisiana	378	7.8	1.5	(4.9–10.7)
Silver Spring-Frederick-Rockville, Maryland^†^	1,791	N/A^§^	N/A^§^	(N/A–N/A)^§^
Sioux City, Iowa-Nebraska-South Dakota	883	5.4	1.3	(2.9–7.9)
Sioux Falls, South Dakota	909	4.0	0.7	(2.7–5.3)
Spartanburg, South Carolina	439	8.7	1.7	(5.4–11.9)
Spokane-Spokane Valley, Washington	553	6.5	1.2	(4.1–8.9)
Springfield, Massachusetts	788	6.8	1.6	(3.6–10.0)
Tampa-St. Petersburg-Clearwater, Florida	1,220	4.7	0.7	(3.4–6.1)
Toledo, Ohio	499	5.5	1.2	(3.2–7.8)
Topeka, Kansas	1,045	3.7	0.6	(2.5–4.8)
Tulsa, Oklahoma	1,477	6.5	0.7	(5.0–7.9)
Tuscaloosa, Alabama	494	7.6	1.8	(4.2–11.1)
Virginia Beach-Norfolk-Newport News, Virginia-North Carolina	1,341	5.6	0.9	(3.9–7.3)
Warren-Troy-Farmington Hills, Michigan^†^	1,544	5.2	0.8	(3.7–6.8)
Washington-Arlington-Alexandria, District of Columbia-Virginia-Maryland-West Virginia^†^	5,949	4.8	0.6	(3.7–5.9)
Wichita, Kansas	1,890	5.1	0.6	(4.0–6.3)
Wichita Falls, Texas	476	4.9	1.1	(2.7–7.1)
Wilmington, Delaware-Maryland-New Jersey^†^	2,065	6.3	0.8	(4.7–7.8)
Worcester, Massachusetts-Connecticut	1,794	3.2	0.6	(2.0–4.3)
Youngstown-Warren-Boardman, Ohio-Pennsylvania	419	5.2	1.3	(2.7–7.6)
*Median*		*5.0*		
*Range*		*2.3–9.0*		

**Abbreviations:** CI = confidence interval; MMSA = metropolitan and micropolitan statistical areas; N/A = not available; SE = standard error.

* Age adjusted to the 2000 U.S. standard population.

^†^ Metropolitan division.

^§^ Estimate not available if the unweighted sample size for the denominator was <50 or if the relative standard error is >30%.

#### High Blood Pressure

High blood pressure was defined as respondents who reported having ever been told by a doctor, nurse, or other health professional they have high blood pressure. In 2013, age-adjusted prevalence estimates of high blood pressure ranged from 25.2% in Minnesota to 40.1% in Puerto Rico (median: 29.5%) (Table 61). Among selected MMSA, age-adjusted prevalence estimates ranged from 22.2% in Duluth, Minnesota-Wisconsin, to 42.2% in Fort Smith, Arkansas-Oklahoma (median: 30.4%) (Table 62).

**TABLE 61:** Age-adjusted* prevalence estimates of adults aged ≥18 years who have ever been told by a health professional they have high blood pressure,^†^ by state/territory — Behavioral Risk Factor Surveillance System, United States, 2013

State/Territory	Sample size	%	SE	95% CI
Alabama	6,492	37.5	0.8	(35.9–39.1)
Alaska	4,564	29.9	0.9	(28.2–31.6)
Arizona	4,242	28.8	1.2	(26.6–31.1)
Arkansas	5,245	36.0	0.9	(34.2–37.9)
California	11,502	28.1	0.5	(27.1–29.1)
Colorado	13,615	25.7	0.4	(24.8–26.6)
Connecticut	7,680	28.2	0.7	(27.0–29.5)
Delaware	5,191	32.4	0.8	(30.8–34.0)
District of Columbia	4,913	30.0	0.8	(28.4–31.7)
Florida	34,074	30.3	0.5	(29.3–31.3)
Georgia	8,116	34.3	0.6	(33.0–35.5)
Hawaii	7,831	26.1	0.7	(24.8–27.4)
Idaho	5,611	27.7	0.7	(26.2–29.2)
Illinois	5,603	28.6	0.8	(27.0–30.1)
Indiana	10,298	31.3	0.5	(30.3–32.4)
Iowa	8,139	28.6	0.6	(27.4–29.7)
Kansas	23,221	29.3	0.3	(28.7–29.9)
Kentucky	10,992	36.5	0.6	(35.2–37.7)
Louisiana	5,240	38.0	0.9	(36.2–39.9)
Maine	8,079	29.1	0.6	(27.9–30.4)
Maryland	12,971	30.8	0.5	(29.7–31.8)
Massachusetts	15,012	27.2	0.5	(26.2–28.2)
Michigan	12,728	31.7	0.5	(30.6–32.7)
Minnesota	14,290	25.2	0.6	(24.0–26.4)
Mississippi	7,440	38.0	0.7	(36.6–39.5)
Missouri	7,104	29.5	0.7	(28.1–31.0)
Montana	9,672	26.3	0.6	(25.2–27.3)
Nebraska	17,085	28.3	0.5	(27.3–29.3)
Nevada	5,090	29.1	1.0	(27.1–31.1)
New Hampshire	6,449	27.0	0.7	(25.7–28.3)
New Jersey	13,347	28.4	0.5	(27.4–29.4)
New Mexico	9,289	27.4	0.6	(26.2–28.5)
New York	8,942	29.5	0.6	(28.3–30.6)
North Carolina	8,841	33.2	0.6	(32.0–34.4)
North Dakota	7,788	27.7	0.6	(26.5–28.9)
Ohio	11,934	30.5	0.5	(29.4–31.5)
Oklahoma	8,224	35.4	0.6	(34.2–36.7)
Oregon	5,934	29.5	0.8	(28.0–31.1)
Pennsylvania	11,382	30.3	0.5	(29.3–31.4)
Rhode Island	6,509	31.1	0.7	(29.7–32.5)
South Carolina	10,675	35.6	0.6	(34.4–36.8)
South Dakota	6,883	27.9	0.8	(26.4–29.4)
Tennessee	5,798	36.1	0.8	(34.5–37.7)
Texas	10,865	31.0	0.6	(29.8–32.2)
Utah	12,730	25.8	0.4	(24.9–26.6)
Vermont	6,377	27.6	0.7	(26.4–28.9)
Virginia	8,443	30.9	0.6	(29.7–32.1)
Washington	11,140	28.9	0.5	(27.8–29.9)
West Virginia	5,879	36.7	0.7	(35.3–38.1)
Wisconsin	6,575	29.6	0.8	(28.0–31.2)
Wyoming	6,434	26.8	0.7	(25.5–28.1)
Guam	1,889	31.5	1.3	(28.9–34.1)
Puerto Rico	5,996	40.1	0.8	(38.6–41.6)
*Median*		*29.5*		
*Range*		*25.2–40.1*		

**Abbreviations:** CI = confidence interval; SE = standard error.

* Age adjusted to the 2000 U.S. standard population.

^†^ Excluding pregnant women.

**TABLE 62:** Age-adjusted* prevalence estimates of adults aged ≥18 years who have ever been told by a health professional they have high blood pressure,^†^ by metropolitan and micropolitan statistical area — Behavioral Risk Factor Surveillance System, United States, 2013

MMSA	Sample size	%	SE	95% CI
Aguadilla-Isabela, Puerto Rico	591	37.6	2.3	(33.1–42.0)
Akron, Ohio	691	28.3	2.2	(24.0–32.7)
Albuquerque, New Mexico	2,078	25.2	1.1	(23.1–27.2)
Allentown-Bethlehem-Easton, Pennsylvania-New Jersey	1,029	32.9	2.0	(29.0–36.7)
Anchorage, Alaska	1,521	29.8	1.3	(27.2–32.4)
Atlanta-Sandy Springs-Roswell, Georgia	3,506	30.8	0.9	(29.1–32.6)
Augusta-Richmond County, Georgia-South Carolina	908	40.2	2.4	(35.4–44.9)
Austin-Round Rock, Texas	935	27.8	1.8	(24.3–31.3)
Baltimore-Columbia-Towson, Maryland	4,751	30.8	0.8	(29.2–32.4)
Baton Rouge, Louisiana	927	39.7	2.3	(35.2–44.2)
Billings, Montana	817	26.8	1.6	(23.6–29.9)
Birmingham-Hoover, Alabama	1,352	35.6	1.5	(32.6–38.6)
Bismarck, North Dakota	1,034	27.9	1.5	(25.0–30.7)
Boise City, Idaho	1,483	28.7	1.4	(25.9–31.4)
Boston, Massachusetts^§^	4,057	27.4	0.9	(25.6–29.3)
Buffalo-Cheektowaga-Niagara Falls, New York	505	26.5	2.3	(21.9–31.0)
Burlington-South Burlington, Vermont	1,630	26.5	1.2	(24.2–28.8)
Cambridge-Newton-Framingham, Massachusetts^§^	4,880	26.8	0.9	(25.0–28.5)
Camden, New Jersey^§^	1,862	30.4	1.2	(28.1–32.6)
Cedar Rapids, Iowa	648	27.6	2.1	(23.5–31.8)
Charleston, West Virginia	817	38.6	1.9	(34.8–42.3)
Charleston-North Charleston, South Carolina	1,546	31.0	1.4	(28.3–33.7)
Charlotte-Concord-Gastonia, North Carolina-South Carolina	1,951	35.2	1.3	(32.6–37.7)
Chattanooga, Tennessee-Georgia	575	37.1	2.7	(31.8–42.3)
Chicago-Naperville-Elgin, Illinois-Indiana-Wisconsin	3,339	27.0	1.0	(25.1–29.0)
Cincinnati, Ohio-Kentucky-Indiana	2,601	31.8	1.2	(29.5–34.1)
Claremont-Lebanon, New Hampshire-Vermont	1,683	26.7	1.4	(23.9–29.5)
Cleveland-Elyria, Ohio	1,104	27.4	1.4	(24.6–30.2)
Colorado Springs, Colorado	1,373	28.9	1.4	(26.1–31.8)
Columbia, South Carolina	1,444	31.5	1.5	(28.6–34.3)
Columbus, Ohio	1,858	31.7	1.2	(29.3–34.1)
Crestview-Fort Walton Beach-Destin, Florida	1,079	34.2	2.0	(30.3–38.1)
Dallas-Plano-Irving, Texas^§^	901	29.3	1.6	(26.2–32.4)
Davenport-Moline-Rock Island, Iowa-Illinois	672	28.8	2.1	(24.7–32.9)
Dayton, Ohio	836	30.3	1.9	(26.6–34.1)
Deltona-Daytona Beach-Ormond Beach, Florida	1,105	30.9	2.2	(26.6–35.2)
Denver-Aurora-Lakewood, Colorado	5,702	25.6	0.6	(24.4–26.8)
Des Moines-West Des Moines, Iowa	1,344	30.8	1.5	(27.8–33.7)
Duluth, Minnesota-Wisconsin	701	22.2	2.1	(18.0–26.4)
Durham-Chapel Hill, North Carolina	616	28.9	2.1	(24.8–33.0)
El Paso, Texas	752	27.6	1.8	(24.0–31.2)
Evansville, Indiana-Kentucky	575	35.9	2.8	(30.3–41.5)
Fargo, North Dakota-Minnesota	1,182	30.2	1.7	(26.9–33.4)
Fayetteville-Springdale-Rogers, Arkansas-Missouri	819	30.6	2.2	(26.2–35.0)
Fort Smith, Arkansas-Oklahoma	496	42.2	3.1	(36.1–48.3)
Fort Wayne, Indiana	780	32.3	2.2	(28.0–36.6)
Fort Worth-Arlington, Texas^§^	809	32.5	2.1	(28.3–36.7)
Gainesville, Florida	1,034	30.8	2.2	(26.6–35.0)
Grand Forks, North Dakota-Minnesota	503	28.7	2.8	(23.2–34.2)
Grand Island, Nebraska	796	30.1	2.0	(26.2–34.0)
Grand Rapids-Wyoming, Michigan	1,342	31.1	1.4	(28.3–33.8)
Greensboro-High Point, North Carolina	665	34.8	2.0	(30.8–38.7)
Greenville-Anderson-Mauldin, South Carolina	1,340	33.1	1.5	(30.1–36.1)
Gulfport-Biloxi-Pascagoula, Mississippi	771	36.8	2.0	(32.9–40.7)
Hagerstown-Martinsburg, Maryland-West Virginia	767	29.8	2.5	(24.9–34.6)
Hartford-West Hartford-East Hartford, Connecticut	2,830	29.1	1.1	(26.9–31.2)
Hilton Head Island-Bluffton-Beaufort, South Carolina	824	28.8	2.1	(24.7–33.0)
Houston-The Woodlands-Sugar Land, Texas	1,382	31.6	1.6	(28.4–34.7)
Huntington-Ashland, West Virginia-Kentucky-Ohio	1,174	39.3	1.6	(36.2–42.4)
Idaho Falls, Idaho	508	28.9	2.3	(24.4–33.5)
Indianapolis-Carmel-Anderson, Indiana	2,533	30.9	1.0	(28.9–32.9)
Jackson, Mississippi	802	34.7	1.9	(31.0–38.4)
Jacksonville, Florida	2,876	31.9	1.2	(29.5–34.2)
Kansas City, Missouri-Kansas	7,406	28.3	1.0	(26.4–30.1)
Kingsport-Bristol-Bristol, Tennessee-Virginia	537	38.1	3.3	(31.6–44.6)
Knoxville, Tennessee	651	31.7	2.0	(27.8–35.6)
Lansing-East Lansing, Michigan	686	28.8	1.7	(25.4–32.2)
Lexington-Fayette, Kentucky	636	33.2	2.0	(29.3–37.0)
Lincoln, Nebraska	1,872	26.5	1.1	(24.4–28.6)
Little Rock-North Little Rock-Conway, Arkansas	1,141	34.5	1.8	(31.0–37.9)
Logan, Utah-Idaho	639	23.2	1.9	(19.5–26.9)
Los Angeles-Long Beach-Anaheim, California	3,039	28.3	0.9	(26.5–30.2)
Louisville-Jefferson County, Kentucky-Indiana	2,146	34.2	1.4	(31.5–36.9)
Lubbock, Texas	529	28.9	2.3	(24.4–33.5)
Manhattan, Kansas	662	26.8	1.7	(23.5–30.1)
Memphis, Tennessee-Mississippi-Arkansas	1,205	36.6	1.7	(33.3–40.0)
Miami-Fort Lauderdale-West Palm Beach, Florida	2,203	27.7	1.3	(25.1–30.3)
Milwaukee-Waukesha-West Allis, Wisconsin	1,266	33.1	1.9	(29.4–36.8)
Minneapolis-St. Paul-Bloomington, Minnesota-Wisconsin	9,099	23.7	0.8	(22.2–25.2)
Minot, North Dakota	652	27.2	1.9	(23.5–30.8)
Montgomery County-Bucks County-Chester County, Pennsylvania^§^	970	29.0	1.6	(25.9–32.0)
Myrtle Beach-Conway-North Myrtle Beach, South Carolina-North Carolina	772	33.0	2.0	(29.0–37.0)
Nashville-Davidson County-Murfreesboro-Franklin, Tennessee	1,062	35.6	1.7	(32.1–39.0)
Nassau County-Suffolk County, New York^§^	938	29.0	1.8	(25.4–32.5)
Newark, New Jersey-Pennsylvania^§^	4,108	28.0	0.9	(26.3–29.8)
New Orleans-Metairie, Louisiana	1,284	35.6	1.9	(31.8–39.3)
New York-Jersey City-White Plains, New York-New Jersey^§^	8,924	29.3	0.6	(28.1–30.5)
Norfolk, Nebraska	670	30.3	2.0	(26.5–34.2)
North Platte, Nebraska	719	30.5	2.0	(26.7–34.3)
North Port-Sarasota-Bradenton, Florida	1,090	27.5	1.8	(23.9–31.1)
Oakland-Hayward-Berkeley, California^§^	699	25.6	2.0	(21.6–29.5)
Ogden-Clearfield, Utah	2,447	28.7	0.9	(26.8–30.5)
Oklahoma City, Oklahoma	2,641	33.8	1.0	(31.8–35.9)
Omaha-Council Bluffs, Nebraska-Iowa	3,118	28.3	1.0	(26.4–30.2)
Orlando-Kissimmee-Sanford, Florida	2,273	31.4	1.4	(28.8–34.1)
Panama City, Florida	1,025	35.7	2.2	(31.4–40.0)
Pensacola-Ferry Pass-Brent, Florida	1,316	31.6	1.4	(28.8–34.4)
Philadelphia, Pennsylvania^§^	1,767	32.2	1.4	(29.4–35.1)
Phoenix-Mesa-Scottsdale, Arizona	1,546	28.1	1.6	(24.9–31.3)
Pittsburgh, Pennsylvania	2,359	28.9	1.1	(26.7–31.1)
Ponce, Puerto Rico	530	39.0	2.5	(34.2–43.9)
Portland-South Portland, Maine	2,633	27.7	1.1	(25.6–29.9)
Portland-Vancouver-Hillsboro, Oregon-Washington	3,238	27.2	1.0	(25.3–29.1)
Port St. Lucie, Florida	1,021	29.8	2.3	(25.2–34.4)
Providence-Warwick, Rhode Island-Massachusetts	8,280	29.9	0.8	(28.4–31.5)
Provo-Orem, Utah	1,854	23.4	1.0	(21.4–25.4)
Raleigh, North Carolina	675	29.8	1.8	(26.2–33.4)
Rapid City, South Dakota	871	30.8	1.9	(27.0–34.6)
Reno, Nevada	1,817	26.3	1.1	(24.2–28.5)
Richmond, Virginia	1,310	35.4	1.6	(32.2–38.6)
Riverside-San Bernardino-Ontario, California	1,369	30.6	1.5	(27.7–33.5)
Rochester, New York	508	30.7	2.2	(26.4–34.9)
Rockingham County-Strafford County, New Hampshire^§^	1,660	27.9	1.3	(25.3–30.5)
Sacramento-Roseville-Arden-Arcade, California	889	28.4	1.7	(25.0–31.7)
St. Louis, Missouri-Illinois	2,059	30.2	1.3	(27.7–32.7)
Salem, Oregon	527	31.2	2.9	(25.6–36.9)
Salina, Kansas	524	32.2	2.3	(27.7–36.7)
Salisbury, Maryland-Delaware	2,062	32.3	1.4	(29.6–35.0)
Salt Lake City, Utah	4,666	25.8	0.7	(24.4–27.2)
San Antonio-New Braunfels, Texas	937	31.9	1.6	(28.7–35.1)
San Francisco-Redwood City-South San Francisco, California^§^	540	24.2	2.2	(20.0–28.5)
San Jose-Sunnyvale-Santa Clara, California	624	26.2	2.4	(21.5–30.9)
San Juan-Carolina-Caguas, Puerto Rico	3,647	40.6	1.0	(38.7–42.5)
Scottsbluff, Nebraska	713	33.1	2.4	(28.4–37.9)
Scranton-Wilkes-Barre-Hazleton, Pennsylvania	566	34.0	2.6	(29.0–39.1)
Seattle-Bellevue-Everett, Washington^§^	3,769	27.9	0.9	(26.3–29.6)
Shreveport-Bossier City, Louisiana	571	40.5	3.0	(34.6–46.4)
Silver Spring-Frederick-Rockville, Maryland^§^	2,417	25.0	1.0	(23.1–27.0)
Sioux City, Iowa-Nebraska-South Dakota	1,054	33.7	2.7	(28.4–39.0)
Sioux Falls, South Dakota	1,007	27.4	1.6	(24.2–30.6)
Spartanburg, South Carolina	595	34.4	2.6	(29.4–39.4)
Spokane-Spokane Valley, Washington	862	29.1	1.9	(25.3–32.9)
Springfield, Massachusetts	1,566	31.6	2.0	(27.6–35.6)
Tallahassee, Florida	1,850	30.8	1.5	(27.8–33.8)
Tampa-St. Petersburg-Clearwater, Florida	2,208	32.4	1.3	(29.9–34.8)
Toledo, Ohio	998	29.9	1.8	(26.4–33.5)
Topeka, Kansas	2,401	30.7	1.0	(28.7–32.7)
Tulsa, Oklahoma	1,995	35.7	1.3	(33.1–38.2)
Virginia Beach-Norfolk-Newport News, Virginia-North Carolina	1,676	31.1	1.4	(28.4–33.8)
Warren-Troy-Farmington Hills, Michigan^§^	2,254	29.5	1.1	(27.3–31.6)
Washington-Arlington-Alexandria, District of Columbia-Virginia-Maryland-West Virginia^§^	9,000	29.1	0.8	(27.6–30.6)
Wichita, Kansas	4,923	31.1	0.7	(29.7–32.6)
Wilmington, Delaware-Maryland-New Jersey^§^	3,272	32.2	0.9	(30.4–34.1)
Winston-Salem, North Carolina	695	33.2	2.4	(28.4–37.9)
Worcester, Massachusetts-Connecticut	2,756	25.7	1.1	(23.6–27.8)
*Median*		*30.4*		
*Range*		*22.2–42.2*		

**Abbreviations:** CI = confidence interval; MMSA = metropolitan and micropolitan statistical areas; SE = standard error.

* Age adjusted to the 2000 U.S. standard population.

^†^ Excluding pregnant women.

^§^ Metropolitan division.

#### High Blood Cholesterol

Respondents were categorized as having high blood cholesterol if, after having their cholesterol checked, they had ever been told by a doctor, nurse, or other health professional it was high. In 2013, age-adjusted prevalence estimates of high blood cholesterol ranged from 28.8% in Vermont to 38.4% in Alabama (median: 33.9%) (Table 63). Among selected MMSA, age-adjusted prevalence estimates ranged from 26.3% in Burlington-South Burlington, Vermont, to 39.6% in Baton Rouge, Louisiana (median: 33.5%) (Table 64).

**TABLE 63:** Age-adjusted* prevalence estimates of adults aged ≥18 years who have ever been told by a health professional they have high blood cholesterol, by state/territory — Behavioral Risk Factor Surveillance System, United States, 2013

State/Territory	Sample size	%	SE	95% CI
Alabama	5,632	38.4	1.0	(36.4–40.4)
Alaska	3,616	35.0	1.1	(32.8–37.2)
Arizona	3,553	35.0	1.5	(32.1–38.0)
Arkansas	4,466	36.1	1.1	(33.9–38.3)
California	9,466	34.1	0.7	(32.8–35.4)
Colorado	11,709	31.4	0.6	(30.3–32.5)
Connecticut	6,823	32.8	0.8	(31.2–34.5)
Delaware	4,547	35.0	1.0	(33.1–36.9)
District of Columbia	4,481	33.3	1.1	(31.2–35.4)
Florida	30,216	34.3	0.6	(33.1–35.6)
Georgia	6,953	34.5	0.8	(33.1–36.0)
Hawaii	6,338	30.4	0.8	(28.7–32.0)
Idaho	4,555	32.9	1.1	(30.8–34.9)
Illinois	4,821	32.0	1.0	(30.1–33.9)
Indiana	8,848	34.7	0.7	(33.3–36.1)
Iowa	7,018	35.2	0.8	(33.6–36.8)
Kansas	19,337	33.0	0.4	(32.2–33.9)
Kentucky	9,563	38.0	0.8	(36.5–39.5)
Louisiana	4,585	36.4	1.2	(34.1–38.7)
Maine	7,221	33.1	0.8	(31.6–34.6)
Maryland	11,613	33.1	0.7	(31.7–34.4)
Massachusetts	13,375	32.5	0.6	(31.2–33.8)
Michigan	11,093	34.8	0.6	(33.6–36.0)
Minnesota	12,421	29.5	0.8	(28.0–31.0)
Mississippi	6,309	37.1	0.9	(35.2–38.9)
Missouri	6,020	33.0	1.0	(31.1–34.9)
Montana	7,945	29.2	0.7	(27.8–30.6)
Nebraska	14,365	32.2	0.6	(31.0–33.5)
Nevada	4,237	34.3	1.4	(31.6–37.0)
New Hampshire	5,809	31.9	0.9	(30.2–33.5)
New Jersey	11,482	34.8	0.7	(33.5–36.2)
New Mexico	7,515	31.8	0.8	(30.2–33.4)
New York	7,752	34.9	0.7	(33.5–36.4)
North Carolina	7,611	36.2	0.8	(34.7–37.7)
North Dakota	6,433	31.3	0.8	(29.7–32.9)
Ohio	10,252	32.3	0.7	(31.0–33.7)
Oklahoma	6,981	35.8	0.8	(34.2–37.3)
Oregon	5,040	31.7	0.9	(29.9–33.6)
Pennsylvania	9,752	33.1	0.7	(31.7–34.4)
Rhode Island	5,895	33.9	0.9	(32.1–35.6)
South Carolina	9,378	37.1	0.8	(35.6–38.6)
South Dakota	5,644	30.5	1.0	(28.5–32.5)
Tennessee	5,012	34.0	1.0	(32.1–35.9)
Texas	9,026	34.6	0.8	(33.0–36.1)
Utah	10,134	31.2	0.5	(30.2–32.3)
Vermont	5,650	28.8	0.8	(27.3–30.3)
Virginia	7,410	34.7	0.8	(33.2–36.2)
Washington	9,524	32.2	0.7	(30.9–33.5)
West Virginia	5,031	36.7	0.9	(35.0–38.4)
Wisconsin	5,663	31.2	1.0	(29.3–33.2)
Wyoming	5,629	30.5	0.9	(28.7–32.3)
Guam	1,341	36.0	1.6	(32.8–39.2)
Puerto Rico	5,092	34.5	0.9	(32.8–36.2)
*Median*		*33.9*		
*Range*		*28.8–38.4*		

**Abbreviations:** CI = confidence interval; SE = standard error.

* Age adjusted to the 2000 U.S. standard population.

**TABLE 64:** Age-adjusted* prevalence estimates of adults aged ≥18 years who have ever been told by a health professional they have high blood cholesterol, by metropolitan and micropolitan statistical area — Behavioral Risk Factor Surveillance System, United States, 2013

MMSA	Sample size	%	SE	95% CI
Aguadilla-Isabela, Puerto Rico	517	31.4	2.7	(26.2–36.6)
Akron, Ohio	600	32.6	3.1	(26.5–38.7)
Albuquerque, New Mexico	1,663	32.1	1.5	(29.1–35.1)
Allentown-Bethlehem-Easton, Pennsylvania-New Jersey	876	35.0	2.5	(30.0–39.9)
Anchorage, Alaska	1,246	34.6	1.7	(31.3–37.8)
Atlanta-Sandy Springs-Roswell, Georgia	2,991	33.4	1.1	(31.3–35.5)
Augusta-Richmond County, Georgia-South Carolina	816	34.5	2.4	(29.7–39.2)
Austin-Round Rock, Texas	784	32.4	1.9	(28.7–36.1)
Baltimore-Columbia-Towson, Maryland	4,273	31.4	1.0	(29.4–33.3)
Baton Rouge, Louisiana	806	39.6	2.9	(33.9–45.4)
Billings, Montana	655	28.4	1.8	(24.8–32.0)
Birmingham-Hoover, Alabama	1,175	37.5	2.0	(33.6–41.4)
Bismarck, North Dakota	883	31.5	1.9	(27.7–35.3)
Boise City, Idaho	1,235	31.9	1.8	(28.4–35.4)
Boston, Massachusetts^†^	3,597	30.9	1.1	(28.7–33.2)
Buffalo-Cheektowaga-Niagara Falls, New York	442	28.9	2.6	(23.7–34.0)
Burlington-South Burlington, Vermont	1,430	26.3	1.3	(23.7–28.8)
Cambridge-Newton-Framingham, Massachusetts^†^	4,339	31.7	1.1	(29.6–33.9)
Camden, New Jersey^†^	1,633	33.7	1.5	(30.7–36.6)
Cedar Rapids, Iowa	568	36.5	2.9	(30.8–42.2)
Charleston, West Virginia	728	35.2	2.0	(31.3–39.1)
Charleston-North Charleston, South Carolina	1,370	33.9	1.6	(30.8–37.0)
Charlotte-Concord-Gastonia, North Carolina-South Carolina	1,671	38.7	1.6	(35.6–41.8)
Chattanooga, Tennessee-Georgia	516	32.9	2.8	(27.4–38.3)
Chicago-Naperville-Elgin, Illinois-Indiana-Wisconsin	2,864	30.5	1.2	(28.2–32.8)
Cincinnati, Ohio-Kentucky-Indiana	2,246	32.5	1.4	(29.9–35.2)
Claremont-Lebanon, New Hampshire-Vermont	1,484	29.4	2.2	(25.1–33.6)
Cleveland-Elyria, Ohio	969	29.7	1.9	(25.9–33.5)
Colorado Springs, Colorado	1,193	33.5	1.7	(30.2–36.8)
Columbia, South Carolina	1,266	34.6	1.9	(30.8–38.4)
Columbus, Ohio	1,550	35.4	1.6	(32.4–38.5)
Crestview-Fort Walton Beach-Destin, Florida	909	33.3	2.4	(28.6–38.0)
Dallas-Plano-Irving, Texas^†^	735	34.3	2.1	(30.1–38.5)
Davenport-Moline-Rock Island, Iowa-Illinois	589	35.0	2.7	(29.7–40.3)
Dayton, Ohio	744	33.4	2.8	(28.0–38.9)
Deltona-Daytona Beach-Ormond Beach, Florida	1,002	38.6	3.2	(32.3–44.9)
Denver-Aurora-Lakewood, Colorado	4,911	31.5	0.8	(30.0–33.0)
Des Moines-West Des Moines, Iowa	1,188	36.4	1.8	(32.9–40.0)
Duluth, Minnesota-Wisconsin	610	28.0	2.3	(23.5–32.6)
Durham-Chapel Hill, North Carolina	540	30.0	2.4	(25.3–34.8)
El Paso, Texas	606	31.9	2.5	(27.1–36.7)
Evansville, Indiana-Kentucky	511	38.2	3.5	(31.4–44.9)
Fargo, North Dakota-Minnesota	987	32.8	2.8	(27.3–38.3)
Fayetteville-Springdale-Rogers, Arkansas-Missouri	662	30.3	2.4	(25.5–35.1)
Fort Smith, Arkansas-Oklahoma	420	34.5	3.4	(27.8–41.3)
Fort Wayne, Indiana	655	36.6	2.8	(31.1–42.1)
Fort Worth-Arlington, Texas^†^	721	32.8	2.2	(28.4–37.2)
Gainesville, Florida	918	31.7	2.5	(26.8–36.7)
Grand Forks, North Dakota-Minnesota	416	29.8	3.5	(22.9–36.7)
Grand Island, Nebraska	685	33.7	2.8	(28.2–39.2)
Grand Rapids-Wyoming, Michigan	1,180	33.5	1.7	(30.3–36.7)
Greensboro-High Point, North Carolina	580	37.6	2.7	(32.4–42.8)
Greenville-Anderson-Mauldin, South Carolina	1,165	35.8	1.9	(32.1–39.4)
Gulfport-Biloxi-Pascagoula, Mississippi	647	36.9	2.6	(31.7–42.1)
Hagerstown-Martinsburg, Maryland-West Virginia	678	34.8	2.8	(29.3–40.3)
Hartford-West Hartford-East Hartford, Connecticut	2,536	34.1	1.3	(31.5–36.6)
Hilton Head Island-Bluffton-Beaufort, South Carolina	750	34.8	2.7	(29.6–40.0)
Houston-The Woodlands-Sugar Land, Texas	1,171	34.2	1.9	(30.4–38.0)
Huntington-Ashland, West Virginia-Kentucky-Ohio	992	36.2	1.9	(32.5–40.0)
Idaho Falls, Idaho	390	36.3	3.6	(29.1–43.4)
Indianapolis-Carmel-Anderson, Indiana	2,176	34.7	1.4	(32.0–37.4)
Jackson, Mississippi	675	36.6	2.6	(31.6–41.7)
Jacksonville, Florida	2,532	33.1	1.4	(30.4–35.7)
Kansas City, Missouri-Kansas	6,383	34.8	1.2	(32.3–37.2)
Kingsport-Bristol-Bristol, Tennessee-Virginia	486	32.6	3.2	(26.3–38.8)
Knoxville, Tennessee	558	33.3	2.6	(28.2–38.4)
Lansing-East Lansing, Michigan	588	34.6	2.4	(29.9–39.3)
Lexington-Fayette, Kentucky	513	38.7	2.9	(33.1–44.3)
Lincoln, Nebraska	1,528	32.0	1.3	(29.4–34.5)
Little Rock-North Little Rock-Conway, Arkansas	995	39.1	2.3	(34.6–43.6)
Logan, Utah-Idaho	472	30.7	2.5	(25.8–35.6)
Los Angeles-Long Beach-Anaheim, California	2,511	36.4	1.3	(33.9–38.9)
Louisville-Jefferson County, Kentucky-Indiana	1,881	36.2	1.6	(33.0–39.4)
Lubbock, Texas	455	36.2	3.8	(28.7–43.6)
Manhattan, Kansas	514	30.2	2.1	(26.0–34.4)
Memphis, Tennessee-Mississippi-Arkansas	1,052	33.8	1.8	(30.3–37.3)
Miami-Fort Lauderdale-West Palm Beach, Florida	1,936	31.6	1.6	(28.5–34.8)
Milwaukee-Waukesha-West Allis, Wisconsin	1,083	31.5	2.2	(27.2–35.8)
Minneapolis-St. Paul-Bloomington, Minnesota-Wisconsin	8,025	28.9	1.0	(27.0–30.8)
Minot, North Dakota	533	29.8	2.3	(25.2–34.4)
Montgomery County-Bucks County-Chester County, Pennsylvania^†^	846	32.5	1.9	(28.7–36.2)
Myrtle Beach-Conway-North Myrtle Beach, South Carolina-North Carolina	665	38.4	3.0	(32.6–44.3)
Nashville-Davidson County-Murfreesboro-Franklin, Tennessee	901	33.2	1.9	(29.4–37.0)
Nassau County-Suffolk County, New York^†^	828	37.4	2.3	(32.9–41.9)
Newark, New Jersey-Pennsylvania^†^	3,550	34.5	1.3	(32.0–36.9)
New Orleans-Metairie, Louisiana	1,127	33.9	2.3	(29.3–38.5)
New York-Jersey City-White Plains, New York-New Jersey^†^	7,644	35.1	0.8	(33.5–36.7)
Norfolk, Nebraska	562	36.0	2.6	(30.9–41.2)
North Platte, Nebraska	608	37.4	3.1	(31.3–43.4)
North Port-Sarasota-Bradenton, Florida	958	38.0	2.6	(32.8–43.2)
Oakland-Hayward-Berkeley, California^†^	568	32.7	2.5	(27.8–37.6)
Ogden-Clearfield, Utah	2,008	31.9	1.1	(29.7–34.1)
Oklahoma City, Oklahoma	2,246	36.6	1.4	(33.9–39.4)
Omaha-Council Bluffs, Nebraska-Iowa	2,660	32.9	1.2	(30.6–35.3)
Orlando-Kissimmee-Sanford, Florida	2,013	36.0	1.6	(32.9–39.1)
Panama City, Florida	908	35.4	2.4	(30.6–40.1)
Pensacola-Ferry Pass-Brent, Florida	1,103	32.2	1.8	(28.7–35.6)
Philadelphia, Pennsylvania^†^	1,503	33.4	1.8	(29.9–36.8)
Phoenix-Mesa-Scottsdale, Arizona	1,293	36.1	2.1	(32.0–40.3)
Pittsburgh, Pennsylvania	2,044	30.7	1.5	(27.8–33.6)
Ponce, Puerto Rico	445	31.0	2.6	(25.9–36.1)
Portland-South Portland, Maine	2,351	32.1	1.4	(29.4–34.7)
Portland-Vancouver-Hillsboro, Oregon-Washington	2,769	31.3	1.1	(29.1–33.6)
Port St. Lucie, Florida	938	33.8	2.9	(28.1–39.4)
Providence-Warwick, Rhode Island-Massachusetts	7,502	35.6	1.1	(33.4–37.8)
Provo-Orem, Utah	1,379	30.0	1.4	(27.3–32.6)
Raleigh, North Carolina	550	31.6	2.1	(27.5–35.8)
Rapid City, South Dakota	734	30.9	2.4	(26.3–35.5)
Reno, Nevada	1,518	31.6	1.5	(28.7–34.6)
Richmond, Virginia	1,154	33.3	1.8	(29.7–36.9)
Riverside-San Bernardino-Ontario, California	1,108	33.4	1.8	(30.0–36.9)
Rochester, New York	448	29.6	2.5	(24.8–34.5)
Rockingham County-Strafford County, New Hampshire^†^	1,505	32.2	1.5	(29.1–35.2)
Sacramento-Roseville-Arden-Arcade, California	745	30.4	2.2	(26.1–34.6)
St. Louis, Missouri-Illinois	1,776	31.6	1.6	(28.4–34.8)
Salem, Oregon	450	33.7	4.0	(25.9–41.5)
Salina, Kansas	428	33.2	3.0	(27.2–39.1)
Salisbury, Maryland-Delaware	1,846	36.6	2.0	(32.7–40.5)
Salt Lake City, Utah	3,755	31.5	0.9	(29.7–33.3)
San Antonio-New Braunfels, Texas	786	34.6	2.2	(30.2–39.0)
San Francisco-Redwood City-South San Francisco, California^†^	449	36.2	3.1	(30.1–42.2)
San Jose-Sunnyvale-Santa Clara, California	517	31.6	2.6	(26.5–36.7)
San Juan-Carolina-Caguas, Puerto Rico	3,069	35.3	1.1	(33.0–37.5)
Scottsbluff, Nebraska	597	30.5	2.5	(25.7–35.4)
Scranton-Wilkes-Barre-Hazleton, Pennsylvania	491	33.5	3.2	(27.2–39.8)
Seattle-Bellevue-Everett, Washington^†^	3,224	31.2	1.0	(29.2–33.3)
Shreveport-Bossier City, Louisiana	499	38.7	3.7	(31.5–45.8)
Silver Spring-Frederick-Rockville, Maryland^†^	2,156	34.3	1.5	(31.4–37.3)
Sioux City, Iowa-Nebraska-South Dakota	845	31.4	3.0	(25.5–37.2)
Sioux Falls, South Dakota	826	29.8	2.0	(25.8–33.8)
Spartanburg, South Carolina	508	37.4	3.7	(30.2–44.7)
Spokane-Spokane Valley, Washington	732	33.6	2.7	(28.4–38.9)
Springfield, Massachusetts	1,397	32.2	2.1	(28.0–36.3)
Tallahassee, Florida	1,664	34.0	1.9	(30.2–37.8)
Tampa-St. Petersburg-Clearwater, Florida	1,944	37.1	1.7	(33.9–40.4)
Toledo, Ohio	864	30.4	2.2	(26.2–34.6)
Topeka, Kansas	2,044	34.4	1.4	(31.7–37.0)
Tulsa, Oklahoma	1,707	35.9	1.6	(32.9–39.0)
Virginia Beach-Norfolk-Newport News, Virginia-North Carolina	1,481	31.5	1.4	(28.7–34.4)
Warren-Troy-Farmington Hills, Michigan^†^	2,016	34.3	1.3	(31.7–36.9)
Washington-Arlington-Alexandria, District of Columbia-Virginia-Maryland-West Virginia^†^	8,058	34.5	1.1	(32.5–36.6)
Wichita, Kansas	4,104	34.5	1.0	(32.6–36.4)
Wilmington, Delaware-Maryland-New Jersey^†^	2,835	34.8	1.2	(32.3–37.2)
Winston-Salem, North Carolina	617	38.3	2.7	(33.1–43.6)
Worcester, Massachusetts-Connecticut	2,419	35.9	1.6	(32.8–39.0)
*Median*		*33.5*		
*Range*		*26.3–39.6*		

**Abbreviations:** CI = confidence interval; MMSA = metropolitan and micropolitan statistical areas; SE = standard error.

* Age adjusted to the 2000 U.S. standard population.

^†^ Metropolitan division.

## Discussion

Considerable variation exists at the levels of state, territory, and MMSA in age-adjusted prevalence estimates of health status, health care access, health risk behaviors, use of preventive practices, and chronic diseases and conditions among U.S. adults. The variations might reflect differences in demographic factors of respondents, including race and sex distribution of the population; socioeconomic conditions, including education level, income level, and employment status; state laws and local ordinances relating to health policy; availability of and access to health care services; use of preventive health services; and patterns of reimbursement for preventive services.

### Health Status Indicators

Use of a single question to measure self-rated health status is complex because it includes a person’s physical health, mental health, and functional capacity (*18*). Health status is a measure of the perceived effects of acute and chronic health conditions (*19*). In this report, variations of prevalence estimates of good or better health across states, territories, and MMSA suggest differences in patterns of chronic disease, health care access, and health behaviors.

Frequent mental distress assesses both the effects of chronic disease and self-reported mental distress (*20*). Persons with frequent mental distress are at a higher risk for certain health risk behaviors (e.g., physical inactivity, inadequate sleep, smoking, and drinking) and chronic diseases and conditions (e.g., diabetes, high blood pressure, heart disease, stroke, asthma, and arthritis) (*21*,*22*). Similarly, frequent physical distress is a measure of physical symptoms related to chronic diseases and conditions (e.g., cancer, diabetes, obesity, and arthritis) and health risk factors (e.g., body mass index, physical inactivity, and smoking status) (*19*). The questions related to frequent physical distress have demonstrated validity and reliability for population health surveillance (*23*). Both frequent mental distress and frequent physical distress are measured and tracked by the health-related quality of life question (*19*). The wide variation in frequent mental distress and frequent physical distress indicates the continued need for surveillance of symptoms related to mental and physical unhealthy days at state and local levels (*23*).

### Health Care Access and Preventive Practices

Health care coverage is associated with access to preventive health care, and lack of health insurance can often lead to adverse health outcomes (*24*). In 2010, one in four adults did not have health care coverage, and those who had a chronic illness and did not have health insurance were more likely to skip or delay medical care because of cost (*25*).

In the United States, cancer is a major public health problem and is the second-leading cause of death (*26*). In 2014, having a Pap test among women aged 21–65 years varied among states. This difference might be associated with lack of access to health care and lack of health insurance (*27*). Evidence-based public health approaches can improve cervical cancer screening among women in this age group. Colorectal cancer is the second leading cause of death from cancers that affect both men and women (*28*). Screening is key to finding precancerous polyps, and early detection makes colorectal cancer easier to treat. USPSTF recommends colorectal cancer screening for adults aged 50–75 years. The 2014 estimates suggest the need for continued population-level efforts to identify groups that are not receiving colorectal cancer screening.

### Health Risk Behaviors

Staying physically active is an important part of improving health; it helps maintain a healthy weight, improves cardiorespiratory efficiency, strengthens muscles and bones, lowers stress, and can improve one’s mental health and mood (*29*). Physical inactivity is a risk factor for chronic diseases and conditions (e.g., diabetes, heart disease, and arthritis) (*30*). BRFSS measured physical inactivity or lack of exercise as no leisure-time activity during the past 30 days. The varying prevalence of no leisure-time physical activity among states and MMSA indicates the need to implement strategies outlined in the CDC *Guide to Strategies to Increase Physical Activity in the Community* (*31*).

Good sleep is critical for good health and overall quality of life (*32*). The American Academy of Sleep Medicine and Sleep Research Society recommends that adults aged ≥18 years get at least 7 hours of sleep each night (*33*). Inadequate sleep (<7 hours) is associated with high blood pressure, asthma, arthritis, obesity, diabetes, coronary heart disease, stroke, depression, and other chronic diseases and conditions (*34*). The range of prevalence estimates for 2013 and 2014 shows greater efforts are needed to develop and implement interventions that address multiple health risk factors and conditions associated with insufficient sleep.

Tobacco use continues to be the single most preventable cause of morbidity and mortality worldwide; it is responsible for approximately 6 million deaths per year (*35*). Smoking causes various types of cancer, cardiovascular and pulmonary diseases, and reproductive and developmental disorders (*36*). Nicotine, found in tobacco products, is acutely toxic, and smoking has been linked to diseases of nearly every organ of the body (*37*). Implementing comprehensive tobacco control programs, which can include a combination of smoke-free laws, cigarette price increases, access to proven smoking cessation treatments and services, and direct media campaigns, can help reduce current smoking prevalence (*38*).

Binge drinking is the most common pattern of excessive alcohol use in the United States (*39*). It is a major risk factor for morbidity and mortality and other societal costs (*40*,*41*). Health-related risks extend beyond those stemming from alcohol abuse; binge drinking can lead to risky sexual activity, unintentional injuries, violence, fetal alcohol disorders, and suicide (*42*). In this report, estimated prevalence of binge drinking varies across the United States and might be associated with socioeconomic and demographic factors and alcohol-related policies in states and MMSA (*43*).

### Chronic Diseases and Conditions

Obesity is a national epidemic and a contributing factor in many health problems, including heart disease, stroke, diabetes, and certain types of cancer (*44*). It lowers quality of life and results in higher medical costs (*45*). A 2013 study on obesity reported that prevalence had increased during 1999–2002 and 2007–2010 among both men and women and substantial disparity persisted among certain population groups (*45*). Access to healthy food and regular physical activity, knowledge about healthy servings and portions, community-based social capital, and guidance from health care providers can help persons maintain a healthy weight (*46*).

Arthritis affects 20% (53 million) of the adult population in the United States (*47*), and it is the major contributor to falls among elderly persons (*48*). Physical activity and self-management education interventions can reduce pain and improve function and quality of life for adults with arthritis and for adults with other chronic conditions who might be limited by their arthritis (*49*). In 2013 and 2014, approximately 40% of adults aged ≥45 years had some form of arthritis in each year.

Depression is one of the top five causes of disability; it can cause fatigue, decrease one’s ability to work or attend school, and increase risk for suicide (*50*). The Global Burden of Disease Study 2010 identified depression as a leading cause of morbidity and mortality worldwide (*51*). Varying prevalence estimates among states and MMSA underscore the need for prevention and intervention efforts at state and local levels.

### Cardiovascular Conditions

Heart disease is the leading cause of death in the United States, accounting for 23.4% of all deaths in 2014; stroke is the fifth-leading cause of death, accounting for 5.1% of all deaths in 2014 (*52*). Both heart disease and stroke are major causes of disability among adults (*52*,*53*).

High blood pressure and high cholesterol are primary contributors to heart disease and stroke (*54*). In addition, adults aged ≥45 years with frequent mental distress have been found to have a higher likelihood of heart disease (*55*). High blood pressure and high cholesterol often can be controlled or prevented with medication, regular exercise, and a healthy diet, as well as by quitting smoking, reducing alcohol use, and monitoring blood pressure and cholesterol. Because high blood pressure and high cholesterol are contributors to stroke and heart disease, strategies for prevention and control can help prevent cardiovascular complications.

## Limitations

The findings of this report are subject to at least five limitations. First, because it is a household telephone survey BRFSS excludes information from persons in institutions, military installations, nursing homes, long-term care facilities, and correctional institutions. Second, the questionnaire is administered only to persons who speak English, Spanish, Mandarin, or Portuguese. Persons who do not speak these languages would not be able to participate in the survey. Third, the BRFSS survey collects self-reported data that are subject to recall bias and social desirability effects. Fourth, because of small sample size or unreliable estimates, certain estimates could not be obtained for some MMSA. Finally, persons without a landline or cellular telephone are not able to participate.

Overall, BRFSS is a cost-effective, timely, and flexible surveillance system that provides state health departments and local communities with reliable estimates to monitor and track health status, health risk behaviors, chronic diseases and conditions, and access to preventive health care. Crude estimates obtained using BRFSS are comparable and consistent with other U.S. survey estimates (*56*). BRFSS questions have been shown to be valid and reliable (*6*).

## Conclusion

Although chronic diseases and conditions are a challenge to the overall health of the U.S. population, prevalence of morbidity and mortality can be estimated and reduced by monitoring trends, promoting healthy behaviors, identifying emerging diseases, and building effective and sustainable public health community interventions. Results from this report reflect variations in health status, health care access, health behaviors, and chronic diseases and conditions at state and MMSA levels. Identifying areas with populations at risk can help public health officials address health needs and use limited resources more effectively. BRFSS results can be used to identify emerging health problems, support health-related legislative efforts, and develop and evaluate public health policies and programs at state and local levels. CDC will continue to work with states and territories to collect data, identify populations that are underserved and at risk, monitor chronic diseases and conditions and access to health care, and encourage the U.S. population to adopt healthy behaviors.
